# Non-invasive brain stimulation augmentation therapy for treatment-resistant schizophrenia: a systematic review and network meta-analysis

**DOI:** 10.1016/j.eclinm.2025.103583

**Published:** 2025-10-22

**Authors:** Yaohui Wei, Carolin Lorenz, Spyridon Siafis, Johannes Schneider-Thoma, David D. Kim, Hui Wu, Nobuyuki Nomura, Shimeng Dong, Yuki Furukawa, Yikang Zhu, Irene Bighelli, Wulf-Peter Hansen, Ulrike Vogelmann, Wolfgang Strube, Chunbo Li, John M. Davis, Robert C. Smith, Georgia Salanti, Frank Padberg, Stefan Leucht

**Affiliations:** a Technical University of Munich, TUM School of Medicine and Health, Department of Psychiatry and Psychotherapy, Munich, Germany; b Shanghai Key Laboratory of Psychotic Disorders, Shanghai Mental Health Centre, Shanghai Jiao Tong University School of Medicine, Shanghai, China; c Department of Neuropsychiatry, Keio University School of Medicine, Tokyo, Japan; d Department of Psychiatry and Psychotherapy, LMU University Hospital Munich, Munich, Germany; e Department of Neuropsychiatry, University of Tokyo, Tokyo, Japan; f BASTA Das Bündnis Für Psychisch Erkrankte Menschen, Munich, Germany; g Department of Psychiatry, Psychotherapy and Psychosomatics, Faculty of Medicine, University of Augsburg, Augsburg, Germany; h Institute of Psychology and Behavioral Science, Shanghai Jiao Tong University, Shanghai, China; i Psychiatric Institute, University of Illinois at Chicago, Chicago, IL, USA; j Nathan Kline Institute for Psychiatric Research; Department of Psychiatry, NYU Medical School, USA; k Institute of Social and Preventive Medicine (ISPM), University of Bern, Bern, Switzerland; l DZPG (German Center for Mental Health), Partner Site Munich/Augsburg, Munich, Germany; m DZPG (German Center for Mental Health), Partner Site Munich/Augsburg, Augsburg, Germany; n Maryland Psychiatric Research Center, Baltimore, MD, USA

**Keywords:** Electroconvulsive therapy, Magnetic seizure therapy, Psychosis, Transcranial electric stimulation, Transcranial magnetic stimulation, Treatment-resistance

## Abstract

**Background:**

Non-invasive brain stimulation (NIBS) provides adjunctive therapeutic options for individuals with schizophrenia if medications are insufficient to produce clinical response, but guidelines remain controversial on whether NIBS is effective, and which NIBS methods and targets are preferred. We aimed to compare the efficacy and safety of NIBS for treatment-resistant schizophrenia (TRS).

**Methods:**

This systematic review and network meta-analysis of randomised controlled trials investigated NIBS interventions, including electroconvulsive therapy, magnetic seizure therapy (MST), repetitive transcranial magnetic stimulation (rTMS), and transcranial electric stimulation (tES), as adjunctive treatment for TRS. We searched the Cochrane Schizophrenia Group's specialised register from inception to 2025.07.13, and three Chinese databases from inception to 2024.10.30. The primary outcome was overall symptoms, and adverse events were analysed as secondary outcomes. We synthesized the data using random-effects network meta-analysis. Sensitivity analyses examined the robustness of the findings and the effects of the detailed NIBS protocols. The protocol was pre-registered with PROSPERO (CRD42023410645) and published in a scientific journal.

**Findings:**

We identified 21710 references and included 78 trials with a total of 3416 participants (1216 women, 1733 men; mean age 37.06 years, range 25.55–48.38; ethnicity data were not recorded). Compared with sham stimulation, rTMS (SMD −0.47, 95% CI [−0.62; −0.31]) was more efficacious in improving overall symptoms; but not in a sensitivity analysis excluding studies from Chinese mainland (−0.19, [−0.38; 0.01]). No clear differences between rTMS specific protocols in terms of stimulation targets and protocols were detected. No clear differences were found for electroconvulsive therapy (−0.20, [−0.78; 0.37]), tES (−0.08, [−0.38; 0.22]), and MST (−0.30, [−1.73; 1.13]) compared to sham stimulation. Treatment as usual might be less efficacious than sham (1.13, [−0.13; 2.38]), based on indirect evidence. NIBS was generally safe (rTMS produced headaches and local reactions), but information about adverse events was rarely reported.

**Interpretation:**

rTMS may be efficacious in individuals with TRS, but this finding was driven mainly by studies from Chinese mainland. No clear differences were observed for electroconvulsive therapy, tES, and MST, but the findings were imprecise and inconclusive.

**Funding:**

German 10.13039/501100002347Federal Ministry of Education and Research (Bundesministerium für Bildung und Forschung/BMBF; 01KG2206).

**Research in context**

**Evidence before this study**

We searched PubMed from inception to 2025.04.27, for previously published articles on non-invasive brain stimulation (NIBS) for schizophrenia, using search terms including “schizophrenia” and “treatment resistant”, and the filter “Article type: Meta-analysis”, with no language restrictions. Ping-Tao Tseng et al. (2022) conducted a network meta-analysis comparing NIBS interventions, focusing on the negative symptoms of schizophrenia. Michel Sabé et al. (2024) compared repetitive transcranial magnetic stimulation (rTMS) and transcranial electric stimulation (tES) across mental disorders, without comparing interventions with each other. Two meta-analyses examined augmentation strategies for clozapine-refractory schizophrenia, but only electroconvulsive therapy and rTMS were assessed among NIBS. Eleven separate pairwise meta-analyses focused on a specific intervention (four on electroconvulsive therapy, seven on rTMS, and one on tES). Overall, we found no network meta-analyses comparing different NIBS interventions for treatment-resistant schizophrenia.

**Added value of this study**

This study is the first network meta-analysis of randomised controlled trials (RCTs) comparing NIBS for the treatment of treatment-resistant schizophrenia. We included 78 RCTs of 3416 individuals with positive symptoms of schizophrenia or schizoaffective disorder. Our findings indicate that rTMS showed a medium effect size in improving overall symptoms in treatment-resistant schizophrenia. Furthermore, we noted that such effect size decreased to small and became insignificant in the sensitivity analysis excluding studies from Chinese mainland. While low-frequency rTMS targeting temporoparietal or dorsolateral prefrontal cortex regions, high-frequency rTMS targeting dorsolateral prefrontal cortex regions, and continuous theta-burst stimulation targeting the temporoparietal region have the potential to improve overall symptoms, our study did not find clear differences among different rTMS targets and protocols. Sham stimulation might be more efficacious than treatment as usual in improving overall symptoms, highlighting potential placebo effects of NIBS in treating schizophrenia.

**Implications of all the available evidence**

For individuals with treatment-resistant schizophrenia, rTMS may offer benefits, but the conclusion is tempered by the exclusion of studies from Chinese mainland, which drove the effect because they yielded approximately 2.5 times higher effect sizes than the remaining studies. Given the placebo effects of NIBS, future studies should consider using sham stimulation rather than treatment-as-usual as the control and include blinding checks to better manage these effects. Finally, we advocate for future trials evaluating the efficacy and safety of NIBS to implement blinding methodologies (at least rater-blinded RCTs), in particular on electroconvulsive therapy, which is considered an ultima ratio treatment in many guidelines.

## Introduction

Antipsychotic medications have been the cornerstone of schizophrenia treatment since their introduction in the 1950s. However, a substantial proportion of individuals exhibit inadequate response to these drugs, a condition termed treatment-resistant schizophrenia.^1^^,^^2^ Estimates suggest that approximately a third of the individuals with schizophrenia do not respond adequately to antipsychotics.^3^, ^4^, ^5^

Non-invasive brain stimulation (NIBS) techniques, modulating brain activity using electrical currents or magnetic fields without surgical intervention, are potential adjunctive treatments for treatment-resistant schizophrenia.^6^ Among them, repetitive transcranial magnetic stimulation (rTMS) and magnetic seizure therapy (MST) utilise magnetic fields to induce electric currents in the brain, while transcranial electrical stimulation (tES)—primarily transcranial direct current stimulation (tDCS)—and electroconvulsive therapy apply direct or alternating electrical currents to stimulate neural activity. NIBS applications have been guided by symptom-specific targets implicated in the pathophysiology of schizophrenia. Temporoparietal cortex regions are commonly stimulated with so-called inhibitory protocols to reduce auditory hallucinations, while the dorsolateral prefrontal cortex (DLPFC) is targeted with ‘excitatory’ protocols to improve negative symptoms, though the terms ‘excitatory’ and ‘inhibitory’ parameters are not fully valid in terms of their complex neurophysiology and individual differences.^7^ Although concerns persist about their efficacy and safety, electroconvulsive therapy and rTMS are recommended by some guidelines due to high non-response rates to antipsychotics and limited alternative interventions.^8^, ^9^, ^10^

It is unclear whether different NIBS techniques are associated with symptom improvement in treatment-resistant schizophrenia. Previous systematic reviews and meta-analyses are out of date, limited to single interventions (e.g., electroconvulsive therapy or rTMS), did not use network meta-analysis, and lacked focus on treatment-resistant populations.^11^, ^12^, ^13^, ^14^ Additionally, most trials have only compared NIBS techniques against sham stimulation and few against other NIBS techniques. Multiple Chinese studies have explored the efficacy of NIBS in treatment-resistant schizophrenia, but their findings have not been systematically integrated into the global evidence base. Therefore, employing a network meta-analysis, which combines both direct and indirect comparisons of treatments in a single analysis, could provide more comprehensive insights.

To our knowledge, no network meta-analysis has been performed comparing NIBS techniques for the treatment of treatment-resistant schizophrenia. Therefore, we conducted a systematic review and network meta-analysis to evaluate the comparative efficacy and safety of different NIBS techniques.

## Methods

### Search strategy and selection criteria

The systematic review was reported according to the PRISMA extension for network meta-analysis. The protocol was pre-registered with PROSPERO (CRD42023410645) and published in a peer-reviewed journal.^15^ Deviations from the protocol are reported below and in more detail in the appendix.

We searched the EMBASE, PubMed, MEDLINE, PsycINFO, the clinical trials registers of the Cochrane Central Register of Controlled Trials (CENTRAL), ClinicalTrials.gov, and WHO International Clinical Trials Registry Platform published up to 2025.7.13. Chinese databases represent a unique and valuable resource for NIBS studies and should not be overlooked, given the large number of available trials and the widespread use of electroconvulsive therapy for schizophrenia in China compared to other countries.^16^ Therefore, we also searched three main Chinese databases of Wanfang Database, China National Knowledge Infrastructure (CNKI), and China Biology Medicine disc up to 2024.10.30. No language restrictions were applied. A detailed search strategy is available in the appendix (eAppendix 3).

We included single- and double-blind randomised controlled trials (RCTs) investigating NIBS techniques as an adjunct to antipsychotics in adults with treatment-resistant schizophrenia, schizoaffective disorder, or schizophreniform disorder. We accepted any study definition of treatment-resistance and classified the definition into low, intermediate, and high stringency levels for subgroup analyses, consistent with consensus papers.^2^^,^^6^ We included studies where participants exhibited treatment-resistant overall or positive symptoms, such as auditory hallucinations, due to the central role these symptoms play in treatment-resistant schizophrenia.^17^

We included all NIBS techniques and predefined their grouping in the main analysis based on their principal mechanisms: electroconvulsive therapy (electric stimulation inducing a generalized seizure), MST (magnetic stimulation inducing a seizure), rTMS (magnetic pulses without inducing a seizure), and tES (electric current without inducing a seizure). In a sensitivity analysis, we further examined each technique in more detail, considering stimulus type and target location (Fig. 5, eAppendix 11.1). Studies investigating NIBS as monotherapy or single-session interventions were excluded. Additionally, maintenance studies in which participants were stabilized with NIBS before randomization, and studies in predominant negative symptoms, cognitive impairment, or comorbidities were not eligible.

**Fig. 1:**
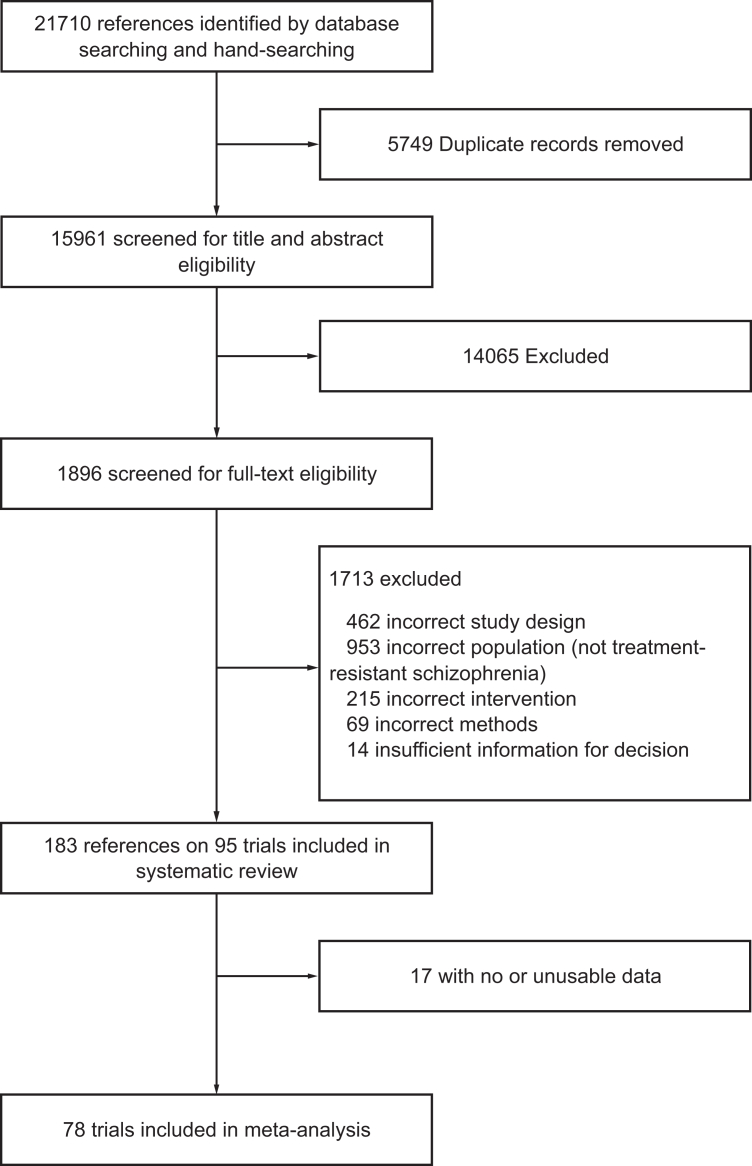
Study selection.

**Fig. 2:**
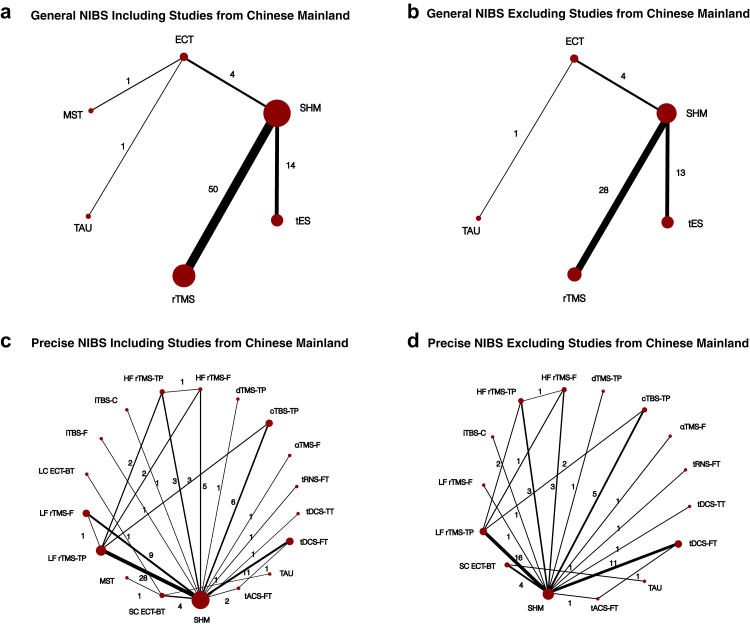
Network plot of the primary outcome overall symptoms (2a, general NIBS including studies from Chinese mainland; 2b, general NIBS excluding studies from Chinese mainland; 2c, precise NIBS protocols with consideration of frequency and targets, including studies from Chinese mainland; 2d, precise NIBS protocols with consideration of frequency and targets, excluding studies from Chinese mainland). αTMS-F: α-peak-frequency–guided transcranial magnetic stimulation, frontal; cTBS-TP: continuous theta burst stimulation, temporoparietal; dTMS-TP: deep transcranial magnetic stimulation, temporoparietal; ECT: electroconvulsive therapy; HF rTMS-F: high frequency rTMS, frontal; HF rTMS-TP: high frequency rTMS, temporoparietal; iTBS-C: intermittent theta burst stimulation, cerebellum; iTBS-F: intermittent theta burst stimulation, frontal; LC ECT-BT: low-charge ECT, bitemporal; LF rTMS-F: low frequency rTMS, frontal; LF rTMS-TP: low frequency rTMS, temporoparietal; MST: magnetic seizure therapy; rTMS: repetitive transcranial magnetic stimulation; SC ECT-BT: standard-charge ECT, bitemporal; SHM: sham therapy; TAU: treatment as usual; tACS-FT: alpha transcranial alternating current stimulation, frontal-temporal; tDCS-FT: transcranial direct current stimulation, frontal-temporal; tES: transcranial electrical stimulation; tRNS-FT: transcranial random noise stimulation, frontal-temporal.

**Fig. 3:**
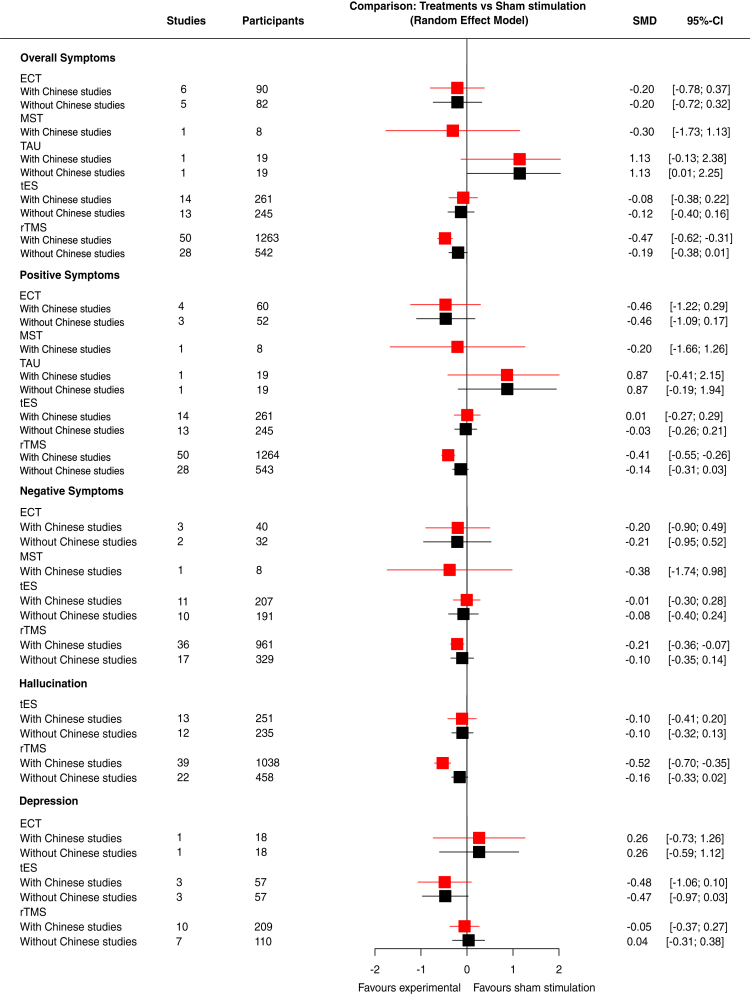
Forest plot of NIBS interventions versus sham stimulation for the outcomes. ECT: electroconvulsive therapy; MST: magnetic seizure therapy; rTMS: repetitive transcranial magnetic stimulation; SHM: sham therapy; TAU: treatment as usual; tES: transcranial electrical stimulation.

**Fig. 4:**
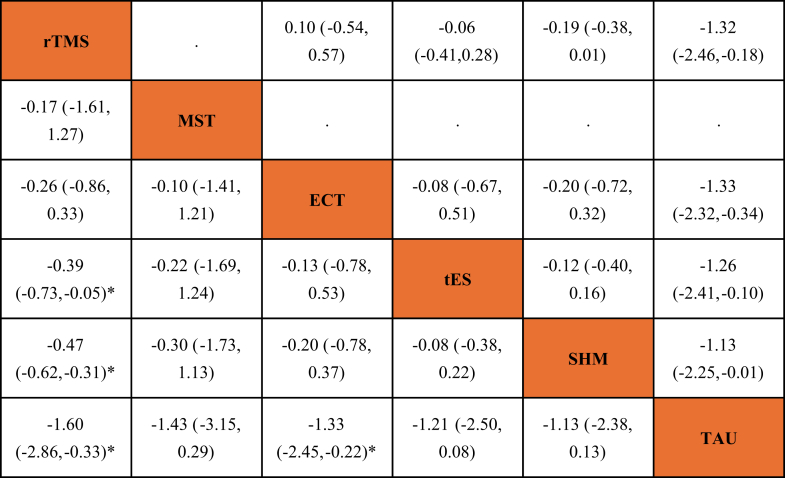
League table of the primary outcome overall symptoms. Order of treatments is according to the mean effect size. Results of the network meta-analysis including studies from Chinese mainland are presented in the left lower half and excluding studies from Chinese mainland in the right upper half. Relative treatment effects are measured by standardised mean differences along with their 95% CIs. The confidence in the evidence was very low for all comparisons. ECT: electroconvulsive therapy; MST: magnetic seizure therapy; rTMS: repetitive transcranial magnetic stimulation; SHM: sham therapy; TAU: treatment as usual; tES: transcranial electrical stimulation. ∗p < 0.05.

**Fig. 5:**
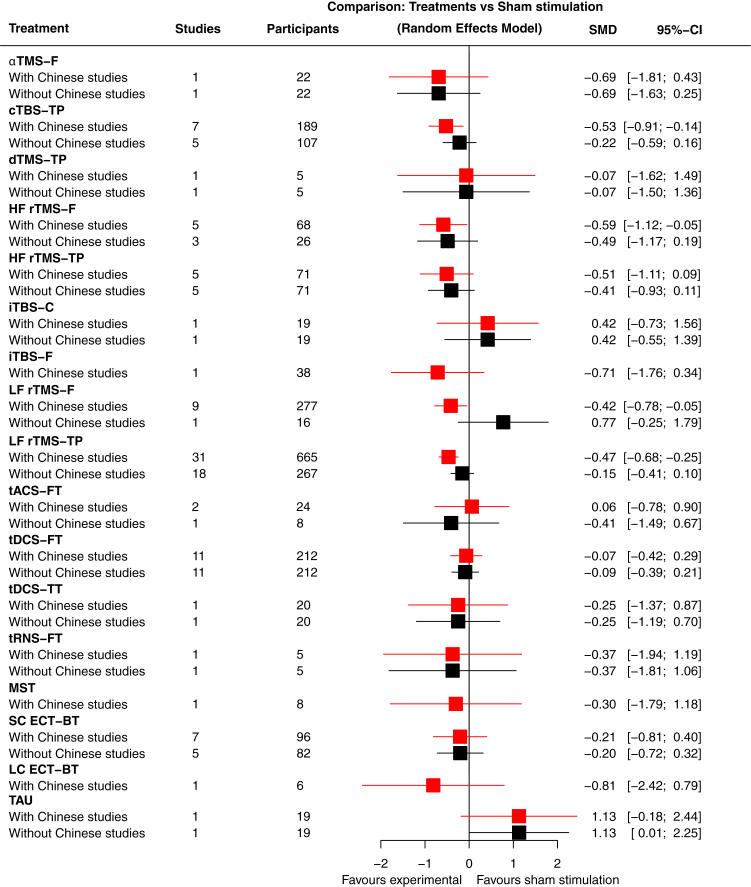
Forest plot of precise NIBS protocols versus sham stimulation for the primary outcome overall symptoms. αTMS-F: α-peak-frequency–guided transcranial magnetic stimulation, frontal; cTBS-TP: continuous theta burst stimulation, temporoparietal; dTMS-TP: deep transcranial magnetic stimulation, temporoparietal; HF rTMS-F: high frequency rTMS, frontal; HF rTMS-TP: high frequency rTMS, temporoparietal; iTBS-C: intermittent theta burst stimulation, cerebellum; iTBS-F: intermittent theta burst stimulation, frontal; LC ECT-BT: low-charge ECT, bitemporal; LF rTMS-F: low frequency rTMS, frontal; LF rTMS-TP: low frequency rTMS, temporoparietal; MST: magnetic seizure therapy; SC ECT-BT: standard-charge ECT, bitemporal; TAU: treatment as usual; tACS-FT: alpha transcranial alternating current stimulation, frontal-temporal; tDCS-FT: transcranial direct current stimulation, frontal-temporal; tDCS-TT, transcranial direct current stimulation, temporal–temporal; tRNS-FT: transcranial random noise stimulation, frontal-temporal.

Two independent reviewers (pairs from CL, NN, YW, HW, YZ, DK, SD, or YF) conducted the study selection in two steps (title/abstract and full-text), extracted the data using a Microsoft Access database specifically developed for this project to ensure consistency, and assessed the risk of bias using the Cochrane Risk of Bias tool 2 (RoB2).^18^ Disagreements were resolved by discussion or by consulting SL, JST, and SS. Where necessary, study authors were contacted to clarify trial details or obtain missing data. An online questionnaire was sent to authors of studies from Chinese databases to verify the randomization and blinding procedures of studies from Chinese mainland.^19^^,^^20^

### Data analysis

The primary outcome was the change in overall symptoms measured with validated scales, preferably the Positive and Negative Syndrome Scale (PANSS).^21^ If not used, validated scales for positive symptoms were employed, including those for auditory hallucinations, as these are a core target of NIBS. A sensitivity analysis excluding such scales was conducted. As secondary outcomes, we included the change in positive and negative symptoms, depressive symptoms, response (number of participants responding to treatment, defined by the original authors, preferably a ≥20% reduction of PANSS total scores),^22^ functioning, quality of life, cognition (classified into global composite scores and scores for the seven domains of MATRICS),^23^ dropouts due to any reason, and side effects known to occur with NIBS, including neurological, cognitive, cardiovascular, and musculoskeletal events. Follow-up assessments of the primary outcomes were conducted after the end of treatment to examine the maintenance of treatment effects over time.

We conducted pairwise and network meta-analyses in a frequentist framework for all outcomes using a random-effects model, except for rare event outcomes where a fixed-effects Mantel–Haenszel method was used. The effect sizes were standardised mean differences (SMDs) for continuous variables and odds ratios (ORs) for dichotomous variables, each reported with 95% CIs, with details on the classification of outcomes provided in eAppendix 2. Heterogeneity was quantified by estimating the between-study variance (τ2) and the I^2^ value.^24^ In the network meta-analysis, a common between-study variance was assumed across treatment comparisons.

Transitivity assumption is a prerequisite for conducting a network meta-analysis, and for this we designed the eligibility criteria accordingly and explored the distribution of potential effect modifiers across treatment comparisons by visual exploration of box plots. For baseline severity,^25^ illness duration,^26^ publication year,^27^ sex,^26^ and sample size,^28^ there is evidence that they may act as effect modifiers. Stringency of treatment-resistant definition as defined above and treatment duration were considered obvious potential effect modifiers by us. We also assessed incoherence (agreement between direct and indirect evidence) using local (SIDE test) and global methods (design-by-treatment interaction test).^29^^,^^30^

To investigate potential sources of heterogeneity or incoherence, we performed prespecified subgroup analyses and meta-regressions examining the above-mentioned potential effect-modifiers of the primary outcome for the pairwise comparison of NIBS with sham stimulation.

The robustness of the findings was assessed in predefined sensitivity analyses of the primary outcome (eAppendix 11), including studies at overall low risk of bias, studies in which randomisation was explicitly mentioned and not just assumed because they were blinded, studies with original SDs needing no imputation, studies not from Chinese mainland,^19^ and studies without predominantly positive symptoms subjects or not using positive symptoms as the primary outcome. Different types of stimulus frequency and of target sites were examined. The aim was to evaluate whether the main results remained under various study characteristics and statistical assumptions. When we observed that studies from Chinese mainland consistently showed higher effect sizes, we post-hoc extended this sensitivity analysis to all secondary outcomes.

We assessed the confidence in the evidence using the Confidence in network meta-analysis (CINeMA) framework.^31^ Data analysis was conducted in R statistical software version 4.2.1 using the packages meta version 8.0–1 and netmeta version 2.9–0. A more detailed explanation of the analytical approach is provided in eAppendix 2.

People with lived experience of mental disorders were involved in identifying and shaping the research question, choosing outcome measures and potential effect modifiers, and interpreting the results.

### Ethics

This study used publicly available data and no ethical approval was required.

### Role of the funding source

The sponsor had no role in the entire process of this study, including study design, data collection, data analysis, data interpretation, or writing the report.

## Results

We identified 21,710 records through our search, in which 1896 potentially eligible articles were assessed in full text. Of these studies, 78 trials comprising 3416 participants were available for inclusion in our meta-analysis, including 27 trials from Chinese mainland (Fig. 1). Of the included trials, 70 provided data for the primary outcome: 50 compared rTMS with sham stimulation, 14 compared tES with sham stimulation (including one tRNS trial, one tACS trial, 11 tDCS trials, and one trial comparing both tACS and tDCS with sham stimulation), four compared electroconvulsive therapy with sham stimulation, one with treatment as usual, and one compared electroconvulsive therapy with MST (Fig. 2). Among the 2949 participants with gender indicated, 1216 (41.0%) were women and 1733 (59.0%) were men. Mean age of participants was 37.06 (range 25.55–48.38) years, and the mean illness duration was 11.01 (range 0.89–25.44) years.

Risk of bias is presented in the appendix (eAppendix 6). Briefly, among the 70 included trials with primary outcome, three (4%) were considered low risk, 36 (51%) were moderate risk, and 31 (44%) were high risk. We found no clear evidence of violations of the transitivity assumption when comparing potential effect modifiers; however, most outcomes had a small number of studies per comparison.

Fig. 2a shows the network plot for the primary outcome of overall symptoms, with available data from 70 studies and 3014 participants. Compared to sham stimulation, rTMS (50 RCTs included in the network meta-analysis; SMD −0.47, 95% CI −0.62 to −0.31) was more efficacious, while no clear differences were observed for electroconvulsive therapy (6 RCTs included; SMD −0.20, 95% CI −0.78 to 0.37), tES (14 RCTs included; SMD −0.08, 95% CI −0.38 to 0.22) and MST (1 RCT included; SMD −0.30, 95% CI −1.73 to 1.13) (Fig. 3). No clear differences were observed in comparisons between NIBS, with some indications that rTMS is more efficacious than tES (SMD −0.39, 95% CI −0.73 to −0.05), but this is mainly based on indirect evidence (Fig. 4). Treatment as usual appeared to be less efficacious compared to sham stimulation (SMD 1.13, 95% CI −0.13 to 2.38), and all active interventions, but based on indirect evidence from one small study with a large difference for the comparison between electroconvulsive therapy and treatment as usual (SMD −1.33, 95% CI −2.45 to −0.22). The confidence in the evidence was very low for all comparisons, mainly due to imprecision (as most comparisons were based on only a few studies with small sample sizes) and incoherence (since inconsistency could not be statistically assessed in a star-shaped network) (Fig. 4, eAppendix 13).

The major finding of the sensitivity analyses of the primary outcome was that, when excluding studies from Chinese mainland, the effect size for rTMS versus sham stimulation was 2.5 times smaller compared to the primary analysis (SMD −0.19, 95% CI −0.38 to 0.01) (see network plot in Fig. 2b). Removing studies from Chinese mainland also reduced heterogeneity from I^2^ = 66.3% to 51.2%. The other sensitivity analyses did not substantially change the results. In particular, we did not find clear differences between targeting the temporoparietal or DLPFC regions, or between different types of stimulation (e.g., continuous theta burst versus standard rTMS stimulation). Moreover, the few significant findings were no longer significant after excluding studies from Chinese mainland (Fig. 2c and d, Fig. 5, eAppendix 11.1).

In subgroup analyses and meta-regressions of the primary outcome, in the comparison between NIBS and sham stimulation, smaller effect sizes were observed in studies involving participants with a longer duration of illness (by 0.34 every additional 10 years, 95% CI: 0.04, 0.64, p-value = 0.029). No clear differences were found for other variables. In particular, there were no clear differences in the subgroups based on treatment-resistance stringency (eAppendix 10).

With regard to the secondary outcomes, the results for positive symptoms, negative symptoms, and hallucinations were similar to those for the primary outcome (i.e., superiority of rTMS over sham stimulation; but again such effects disappeared when excluding studies from the Chinese mainland post hoc, see Fig. 3). There was a signal based on 3 small studies that tES might improve depressive symptoms compared to sham stimulation (3 RCTs with 115 participants included; SMD −0.48, 95% CI −1.06 to 0.10). The data on quality of life and functioning were too limited to be informative. During the follow-ups at the first, third and sixth months, none of the interventions showed significant superiority over sham stimulation in reducing overall symptoms. Some cognitive differences were noted in pairwise comparisons with tES more efficacious to sham stimulation for working memory in one study (SMD −1.20, 95% CI −2.29 to −0.11), and less efficacious for speed of processing based on three studies (SMD 0.41, 95% CI 0.06–0.76) (eAppendix 9.9); however, the available data were too limited to draw definitive conclusions.

In terms of acceptability, participants dropping out of the study early for any reason did not differ between active and sham treatment groups. When analysing adverse events, we found that rTMS was associated with increased rates of headaches and local reactions compared with sham treatment, with no clear differences for the other comparisons. Information on adverse events was generally rarely reported and insufficient to draw firm conclusions.

## Discussion

To our knowledge, this is the first network meta-analysis comparing different NIBS interventions for treatment-resistant schizophrenia. This study is based on 78 trials, which included 3416 participants randomly assigned to one of four NIBS interventions or controls. The main finding was that rTMS was more efficacious than sham stimulation, but the effect size was reduced to a small (SMD −0.19) and imprecise effect when studies from Chinese mainland were excluded from the analysis.

Among NIBS methods, rTMS is the most extensively studied NIBS technique in the context of treatment-resistant schizophrenia. In this network analysis, rTMS was beneficial for reducing overall symptoms of treatment-resistant schizophrenia, but the effects were not consistent when studies from Chinese mainland were excluded. This finding is consistent with a smaller review based on pairwise meta-analysis focusing on auditory hallucinations.^32^ Other pairwise^33^ and network meta-analyses^34^ reported medium effect sizes for rTMS in schizophrenia with negative symptoms. Nevertheless, the authors did not account for the influence of studies from Chinese mainland, despite the fact that many such studies contributed to these meta-analyses. The currently largest meta-analysis,^35^ including 4122 participants with all types of patients, examined only side effects and thus is not directly comparable to our work. Excluding studies from Chinese mainland reduced heterogeneity. Our findings may reflect previously raised concerns regarding reporting and randomization in studies from the Chinese mainland.^19^^,^^20^^,^^36^^,^^37^ Therefore, we made efforts to develop an online questionnaire explaining randomisation, allocation concealment, and blinding, but the response rate was low.

Despite the absence of conclusive evidence for rTMS in our findings, our exploratory analyses offer some insights for future investigation. First, although the effect size of rTMS was only −0.19 after excluding studies from the Chinese mainland and the 95% CI included a small possibility of no effect, more rTMS studies are warranted. It is possible that such studies could increase the effect size to above 0.20, a small but clinically important effect according to Cohen.^38^ Second, as in meta-analyses on antipsychotics,^26^ less chronic patients responded better in meta-regression. Thus, RCTs of NIBS in younger patients are warranted. In terms of stimulus location, there was an unexpected finding that inhibitory rTMS was effective not only over the temporoparietal cortex, but also over the DLPFC. However, the latter analysis was based mostly on studies from Chinese mainland, and replication in other settings is needed. At least theoretically, excitatory stimulation of the DLPFC may be more appropriate for negative symptoms.^7^ As some guidelines, such as those of the American Psychiatric Association (APA),^39^ the Royal Australian and New Zealand College of Psychiatrists,^10^ or the German Psychiatric Association,^9^ recommend electroconvulsive therapy for treatment-resistant schizophrenia, it was somewhat surprising that electroconvulsive therapy did not show clear superiority over sham. Of the six included electroconvulsive therapy studies, only Petrides et al.^40^ showed statistical superiority, but this study did not use a sham control. The same study was also the only electroconvulsive therapy trial included in an network meta-analysis^41^ of various pharmacological and non-pharmacological strategies for clozapine-resistant schizophrenia. The meta-analysis by Lally et al.^42^ concluded in favor of electroconvulsive therapy, but out of eight included studies, Petrides et al.^40^ was the only randomized one. In the meta-analysis by Zheng et al.,^43^ results were not significant after excluding Chinese studies. Our findings are consistent with a Cochrane meta-analysis including open-label RCTs,^11^ which found insufficient evidence to establish electroconvulsive therapy's superiority over sham stimulation as an adjunct to standard care in treatment-resistant schizophrenia. In the growing field of NIBS for schizophrenia, high-quality RCTs on electroconvulsive therapy are urgently needed, as current guidelines rely mainly on the study by Petrides et al.^40^

Depression is a common comorbidity in schizophrenia, affecting approximately one-fourth of individuals across the course of the illness.^44^^,^^45^ Our findings indicated that tDCS might improve depressive symptoms in treatment-resistant schizophrenia compared to sham stimulation, while other NIBS treatments did not show clear effects. However, only three trials comprising 115 participants in total (57 receiving tDCS) evaluated its antidepressant efficacy. Similar effects of tDCS have also been observed in individuals with predominant negative symptoms and in those in the stable phase.^34^^,^^46^ tDCS is better-established as a treatment for major depressive episodes; by contrast, its effects in schizophrenia are less explored.^47^^,^^48^ Nevertheless, alleviating depressive symptoms in schizophrenia with tDCS may be a worthwhile route of further investigation.

Due to limited data, firm conclusions could not be drawn for some secondary outcomes, such as quality of life, functioning, and cognition. In one study tDCS showed better efficacy than sham stimulation on working memory, consistent with findings from a meta-analysis of NIBS across mental disorders in which tDCS but not rTMS was effective,^49^ as well as several positive trials that did not meet our inclusion criteria. Smith et al.,^50^ Lesoni et al.,^51^ and Jeon et al.^52^ examined cognition in stable patients, and the study by Narita et al.^53^ was an uncontrolled trial. In contrast, Bulubas et al.^54^ did not find significant effects on cognition in patients with negative symptoms. We encourage future studies with larger samples and standardized cognitive assessments to further investigate the cognitive effects of tDCS and other brain stimulation techniques in treatment-resistant schizophrenia. Moreover, studies in stable, non-treatment-resistant patients seem promising.^50^

Our findings indicated a placebo effect in the use of NIBS, because sham stimulation outperformed treatment as usual. Recent meta-analyses also point to a placebo response in NIBS treatments for certain mental disorders.^55^, ^56^, ^57^ Given the ubiquity of placebo effects in medical practice, future NIBS studies should consider assessing subjects' expectations about stimulation efficacy and include a questionnaire in which participants are asked to guess their group allocation. This also highlights the importance of using sham controls instead of treatment as usual as comparison groups in these trials.

Our study has several limitations. First, most trials compared NIBS technology with sham stimulation, with few studies comparing active interventions head-to-head, resulting in a mainly star-shaped network. This means that comparisons between NIBS techniques were mainly indirect. Further direct comparisons may reveal differences between interventions. For the same reason, local and global inconsistency tests could not be applied due to the absence of closed loops. Therefore, our findings need to be cautiously interpreted, and the confidence level expressed by CINeMA downgraded accordingly. Nevertheless, pairwise meta-analyses showed essentially the same findings for comparisons of the various NIBS interventions versus sham stimulation (eAppendix 8, 9).

Second, NIBS studies are distributed disproportionately. There is a meaningful number of rTMS trials and a lower number of tES studies, but the number of RCTs with electroconvulsive therapy and MST data is very small and insufficient. Therefore, these NIBS techniques should not be given up prematurely. Rather, more non-open-label RCTs are needed. electroconvulsive therapy studies comparing electroconvulsive therapy with sham are particularly needed to understand their effects more clearly. Yet sham stimulation in electroconvulsive therapy studies might be challenging due to the requirement of general anaesthesia. Moreover, although there are many rTMS studies, many of them had relatively small sample sizes, which can exaggerate the reported effect sizes (eAppendix 12).^58^

Third, we used a broad definition of NIBS which included MST and electroconvulsive therapy. Despite the induction of seizures and anesthesia, both electroconvulsive therapy and MST are often classified as NIBS techniques (i.e., ‘convulsive’ techniques) in many studies and in treatment guidelines,^9^^,^^59^^,^^60^ as they are non-surgical and transcranially applied.

Fourth, definitions of treatment-resistant schizophrenia varied across studies, ranging from partial nonresponse to clozapine resistance. We categorized studies by definition stringency to reflect this variation and examined them in a subgroup analysis. No clear differences were identified, but underlying clinical profiles could still be heterogeneous. For example, some electroconvulsive therapy studies focused on clozapine-resistant individuals, unlike most TMS and tES trials. Nevertheless, the main definition of treatment-resistant schizophrenia was as a lack of response to at least two antipsychotics, which aligns with clinical practice and a recent consensus.^2^ We encourage future trials to adopt standardized treatment-resistant schizophrenia criteria to improve comparability and transitivity, as recently proposed by the Treatment Response and Resistance in Psychosis working group.^2^

Fifth, we used a broad grouping method for both rTMS and tES protocols. While this may introduce some heterogeneity, more fine-grained categorization was limited by the small number of studies available for specific protocols. Furthermore, sensitivity analyses using precise NIBS protocols yielded results consistent with our primary findings, supporting the validity of our overall conclusions.

In summary, our study suggests that, from the main analysis, individuals with treatment-resistant schizophrenia may benefit from rTMS, but the effect size was small when studies from Chinese mainland were excluded. The low level of certainty in the evidence together with insights from secondary analyses call for more evidence to further clarify the role of rTMS. Other NIBS methods should not be given up prematurely. This may be most urgently needed for electroconvulsive therapy, because electroconvulsive therapy is still widely used and recommended in guidelines, but firm evidence for its effectiveness is lacking.

## Contributors

SL obtained the funding and supervised the study. SS, CL, HW, YZ, JS-T, IB, CL, W-P H, FP, GS and SL designed the systematic review and meta-analysis. CL, HW, YW, YZ, DK, and YF screened the literature searches, CL and YW contacted study authors. CL, NN, YW, DK and SD extracted data from included studies and assessed risk of bias. YW conducted the network meta-analyses under the supervision of JS-T and SS. W-PH provided the patients’ perspective when designing the study and interpreting the results. YW, SS, JS-T, UV, WS, FB, JMD, RCS and SL interpreted the results. YW drafted the manuscript. YW and DK have accessed and verified the underlying data. All authors critically reviewed the report for important intellectual content, approved the final submitted version, and accepted responsibility to submit it for publication.

## Data sharing statements

The appendix (available with publication) contains the aggregate outcome data per study, the meta-analytic results per comparison, and most descriptive variables used for our analysis. Additional variables can be shared upon reasonable written request.

## Declaration of interests

SL has in the last 3 years received honoraria for advising or consulting, or for lectures or educational material from Angelini, Apsen, Boehringer Ingelheim, Janssen, Karuna, Kynexis, Lundbeck, Orionpharma, Otsuka, Neurotorium, Novo Nordisk, Recordati, Rovi, Roche, and TEVA Angelini. HW is a senior editor at The Lancet Public Health, but was not involved in any of the editorial process of this manuscript. Her contribution happened before she was employed by Elsevier. WS has received paid speakership reimbursements from ROVI. NN has received grant from the German Center for Mental Health (DZPG) (grant: 01EE2303A, 01EE2303F) and has received manuscript fees from Sumitomo Pharma. RCS is a fellow in The American College of Neuropsychopharmacology (ACNP) committee. FP has received grant from the German Center for Mental Health (DZPG) (grant: 01EE2303), German Federal Ministry of Education and Research (BMBF) (grant: FKZ01EE1403G, FKZ01EW1903), German Research Foundation (DFG) (grant: BR4264/6-1), and has received honoraria for advising or consulting, or for lectures or educational material from Mag&More, NeuroCare Group, Brainsway Inc., Sooma, and European Innovation Council Pathfinder Project CITRUS.

All other authors declare no competing interests.
