# Supplementary material for: Non-invasive brain stimulation augmentation therapy for treatment-resistant schizophrenia: a systematic review and network meta-analysis
Source: eClinicalMedicine. 2025 Oct 22;89:103583. doi: 10.1016/j.eclinm.2025.103583 (PMC12589960; doi:10.1016/j.eclinm.2025.103583)
Supplement: Supplementary Files [file mmc1.docx]

**Non-invasive brain stimulation augmentation therapy for treatment-resistant schizophrenia: a systematic review and network meta-analysis**

**Supplementary material**

**Funding statement**

This project has received funding from the German Federal Ministry of Education and Research (Bundesministerium für Bildung und Forschung/BMBF; 01KG2206)

Indix

[1. PRISMA checklist 5](#_Toc209621354)

[2. Analytical approach and descriptions 9](#_Toc209621355)

[3. Database search strategy 14](#_Toc209621356)

[3.1 Systematic searches 14](#_Toc209621357)

[3.2 Cochrane Schizophrenia Group searches 21](#_Toc209621358)

[3.3 Chinese databases systematic searches 22](#_Toc209621359)

[4. Study selection 25](#_Toc209621360)

[4.1 Criteria for Treatment-Resistant Categorization 25](#_Toc209621361)

[4.2 Communication with authors 26](#_Toc209621362)

[5. Description and references of included studies 29](#_Toc209621363)

[6. Risk of Bias 43](#_Toc209621364)

[7. Transitivity assessments 47](#_Toc209621365)

[7.1 Baseline severity of symptoms 47](#_Toc209621366)

[7.2 Duration of illness 48](#_Toc209621367)

[7.3 Publication year 48](#_Toc209621368)

[7.4 Sample size 49](#_Toc209621369)

[7.5 Treatment duration 49](#_Toc209621370)

[7.6 Mean age of participants 50](#_Toc209621371)

[7.7 Women proportion of participants 50](#_Toc209621372)

[7.8 Definition of treatment-resistance 51](#_Toc209621373)

[7.9 Diagnosis (schizophrenia or schizoaffective disorder) 51](#_Toc209621374)

[7.10 Use of PANSS for rating scales 51](#_Toc209621375)

[7.11 Blinding status 51](#_Toc209621376)

[7.12 Sponsorship 52](#_Toc209621377)

[8. Results of the primary outcome: overall symptoms 53](#_Toc209621378)

[9. Results of network and pairwise meta-analyses of secondary outcomes 60](#_Toc209621379)

[9.1 Positive symptoms 61](#_Toc209621380)

[9.2 Negative symptoms 67](#_Toc209621381)

[9.3 Hallucination 72](#_Toc209621382)

[9.4 Depression 77](#_Toc209621383)

[9.5 Functioning 82](#_Toc209621384)

[9.6 Quality of life 82](#_Toc209621385)

[9.7 Response 83](#_Toc209621386)

[9.8 Dropouts 88](#_Toc209621387)

[9.9 Cognition 93](#_Toc209621388)

[9.9.1 Attention vigilance 93](#_Toc209621389)

[9.9.2 Composite Cognition 94](#_Toc209621390)

[9.9.3 Reasoning and solving 95](#_Toc209621391)

[9.9.4 Social cognition 96](#_Toc209621392)

[9.9.5 Verbal learning 96](#_Toc209621393)

[9.9.6 Visual learning 96](#_Toc209621394)

[9.9.7 Speed of processing 97](#_Toc209621395)

[9.9.8 Working memory 98](#_Toc209621396)

[9.10 Follow-up 99](#_Toc209621397)

[9.10.1 Follow-up in 1 month 99](#_Toc209621398)

[9.10.2 Follow-up in 2 months 104](#_Toc209621399)

[9.10.3 Follow-up in 3 months 104](#_Toc209621400)

[9.10.4 Follow-up in 6 months 104](#_Toc209621401)

[9.11 Side effects 105](#_Toc209621402)

[9.11.1 Headache 105](#_Toc209621403)

[9.11.2 Nausea 110](#_Toc209621404)

[9.11.3 Dizziness 110](#_Toc209621405)

[9.11.4 Sedation 111](#_Toc209621406)

[9.11.5 Depression 112](#_Toc209621407)

[9.11.6 Dystonia 112](#_Toc209621408)

[9.11.7 Mania 113](#_Toc209621409)

[9.11.8 Cognitive problems 113](#_Toc209621410)

[9.11.9 Local reaction 114](#_Toc209621411)

[9.11.10 Hearing Problems 119](#_Toc209621412)

[9.11.11 Myalgia 120](#_Toc209621413)

[9.11.12 Seizures 121](#_Toc209621414)

[10. Subgroup analyses and meta-regressions 123](#_Toc209621415)

[10.1 Baseline severity 124](#_Toc209621416)

[10.2 Definition of treatment-resistance 125](#_Toc209621417)

[10.3 Illness Duration 127](#_Toc209621418)

[10.4 Publication year 128](#_Toc209621419)

[10.5 Sample size 129](#_Toc209621420)

[10.6 Treatment duration 130](#_Toc209621421)

[10.7 Sessions number 131](#_Toc209621422)

[10.8 Clozapine resistance 132](#_Toc209621423)

[11. Sensitivity analyses 134](#_Toc209621424)

[11.1 Different nodes for precise NIBS protocols 135](#_Toc209621425)

[11.1.1 Splitting NIBS into precise protocols, with consideration of frequency 135](#_Toc209621426)

[11.1.2 Splitting NIBS into precise protocols, with consideration of targets 145](#_Toc209621427)

[11.1.3 Splitting NIBS into precise protocols, with consideration of both frequency and targets 155](#_Toc209621428)

[11.2 Different nodes for precise NIBS protocols (excluding atypical protocols) 165](#_Toc209621429)

[11.2.1 Splitting NIBS into precise protocols, with consideration of frequency 165](#_Toc209621430)

[11.2.2 Splitting NIBS into precise protocols, with consideration of targets 175](#_Toc209621431)

[11.2.3 Splitting NIBS into precise protocols, with consideration of both frequency and targets 185](#_Toc209621432)

[11.3 Double blind studies only 195](#_Toc209621433)

[11.4 Excluding studies at high risk of bias 198](#_Toc209621434)

[11.5 Excluding studies with implied randomization 201](#_Toc209621435)

[11.6 Excluding studies that did not use operationalized diagnostic criteria 204](#_Toc209621436)

[11.7 Excluding studies that assumed the administration of antipsychotics 207](#_Toc209621437)

[11.8 Studies in treatment-resistant positive symptom domains 210](#_Toc209621438)

[11.9 Studies with rating scales of positive symptoms used for the primary outcome 213](#_Toc209621439)

[11.10 Studies excluding imputed values 216](#_Toc209621440)

[11.11 Excluding studies from mainland China 219](#_Toc209621441)

[11.12 Excluding studies in treatment-resistant positive symptom domains 222](#_Toc209621442)

[12. Investigation of small study effect and publication bias 225](#_Toc209621443)

[13. Evaluating the Confidence in NMA: CINeMA 226](#_Toc209621444)

[14. Description of NIBS treatment 229](#_Toc209621445)

[14.1 Convulsive NIBS treatment 229](#_Toc209621446)

[14.2 Non-convulsive NIBS treatment 229](#_Toc209621447)

# **PRISMA checklist**

**PRISMA NMA Checklist of Items to Include When Reporting A Systematic Review Involving a Network Meta-analysis**

| **Section/Topic** | **Item #** | **Checklist Item** | **Reported on Page #** |
| --- | --- | --- | --- |
| **TITLE** |  |  |  |
| **Title** | 1 | Identify the report as a systematic review *incorporating a network meta-analysis (or related form of meta-analysis).* | 1 |
|  |  |  |  |
| **ABSTRACT** |  |  |  |
| **Structured summary** | 2 | Provide a structured summary including, as applicable:  Background: main objectives  Methods: data sources; study eligibility criteria, participants, and interventions; study appraisal; and *synthesis methods, such as network meta-analysis.*  Results: number of studies and participants identified; summary estimates with corresponding confidence/credible intervals; *treatment rankings may also be discussed. Authors may choose to summarize pairwise comparisons against a chosen treatment included in their analyses for brevity.*  Discussion/Conclusions: limitations; conclusions and implications of findings.  Other: primary source of funding; systematic review registration number with registry name. | 2 |
|  |  |  |  |
| **INTRODUCTION** |  |  |  |
| **Rationale** | 3 | Describe the rationale for the review in the context of what is already known*, including mention of why a network meta-analysis has been conducted.* | 4 |
| **Objectives** | 4 | Provide an explicit statement of questions being addressed, with reference to participants, interventions, comparisons, outcomes, and study design (PICOS). | 4 |
|  |  |  |  |
| **METHODS** |  |  |  |
| **Protocol and registration** | 5 | Indicate whether a review protocol exists and if and where it can be accessed (e.g., Web address); and, if available, provide registration information, including registration number. | 5 |
| **Eligibility criteria** | 6 | Specify study characteristics (e.g., PICOS, length of follow-up) and report characteristics (e.g., years considered, language, publication status) used as criteria for eligibility, giving rationale. *Clearly describe eligible treatments included in the treatment network, and note whether any have been clustered or merged into the same node (with justification).* | 5 |
| **Information sources** | 7 | Describe all information sources (e.g., databases with dates of coverage, contact with study authors to identify additional studies) in the search and date last searched. | 5 |
| **Search** | 8 | Present full electronic search strategy for at least one database, including any limits used, such that it could be repeated. | eAppendix 3 |
| **Study selection** | 9 | State the process for selecting studies (i.e., screening, eligibility, included in systematic review, and, if applicable, included in the meta-analysis). | 5 |
| **Data collection process** | 10 | Describe method of data extraction from reports (e.g., piloted forms, independently, in duplicate) and any processes for obtaining and confirming data from investigators. | 5 |
| **Data items** | 11 | List and define all variables for which data were sought (e.g., PICOS, funding sources) and any assumptions and simplifications made. | 5 |
| **Geometry of the network** | S1 | Describe methods used to explore the geometry of the treatment network under study and potential biases related to it. This should include how the evidence base has been graphically summarized for presentation, and what characteristics were compiled and used to describe the evidence base to readers. | 5,6 |
| **Risk of bias within individual studies** | 12 | Describe methods used for assessing risk of bias of individual studies (including specification of whether this was done at the study or outcome level), and how this information is to be used in any data synthesis. | 5,6 |
| **Summary measures** | 13 | State the principal summary measures (e.g., risk ratio, difference in means). *Also describe the use of additional summary measures assessed, such as treatment rankings and surface under the cumulative ranking curve (SUCRA) values, as well as modified approaches used to present summary findings from meta-analyses.* | 6 |
| **Planned methods of analysis** | 14 | Describe the methods of handling data and combining results of studies for each network meta-analysis. This should include, but not be limited to:   - *Handling of multi-arm trials;* - *Selection of variance structure;* - *Selection of prior distributions in Bayesian analyses; and* - *Assessment of model fit.* | 6 |
| **Assessment of Inconsistency** | S2 | Describe the statistical methods used to evaluate the agreement of direct and indirect evidence in the treatment network(s) studied. Describe efforts taken to address its presence when found. | 6 |
| **Risk of bias across studies** | 15 | Specify any assessment of risk of bias that may affect the cumulative evidence (e.g., publication bias, selective reporting within studies). | 6 |
| **Additional analyses** | 16 | Describe methods of additional analyses if done, indicating which were pre-specified. This may include, but not be limited to, the following:   - Sensitivity or subgroup analyses; - Meta-regression analyses; - *Alternative formulations of the treatment network; and* - *Use of alternative prior distributions for Bayesian analyses (if applicable).* | 6 |
|  |  |  |  |
| **RESULTS†** |  |  |  |
| **Study selection** | 17 | Give numbers of studies screened, assessed for eligibility, and included in the review, with reasons for exclusions at each stage, ideally with a flow diagram. | 8, Figure 1 |
| **Presentation of network structure** | S3 | Provide a network graph of the included studies to enable visualization of the geometry of the treatment network. | Figure 2 |
| **Summary of network geometry** | S4 | Provide a brief overview of characteristics of the treatment network. This may include commentary on the abundance of trials and randomized patients for the different interventions and pairwise comparisons in the network, gaps of evidence in the treatment network, and potential biases reflected by the network structure. | 8 |
| **Study characteristics** | 18 | For each study, present characteristics for which data were extracted (e.g., study size, PICOS, follow-up period) and provide the citations. | 8, eAppendix 4 |
| **Risk of bias within studies** | 19 | Present data on risk of bias of each study and, if available, any outcome level assessment. | 8, eAppendix 6 |
| **Results of individual studies** | 20 | For all outcomes considered (benefits or harms), present, for each study: 1) simple summary data for each intervention group, and 2) effect estimates and confidence intervals. *Modified approaches may be needed to deal with information from larger networks.* | 8,9  eAppendix 8, eAppendix 9 |
| **Synthesis of results** | 21 | Present results of each meta-analysis done, including confidence/credible intervals. *In larger networks, authors may focus on comparisons versus a particular comparator (e.g. placebo or standard care), with full findings presented in an appendix. League tables and forest plots may be considered to summarize pairwise comparisons.* If additional summary measures were explored (such as treatment rankings), these should also be presented. | 8,9  eAppendix 8, eAppendix 9, Figure 3, Figure 5 |
| **Exploration for inconsistency** | S5 | Describe results from investigations of inconsistency. This may include such information as measures of model fit to compare consistency and inconsistency models, *P* values from statistical tests, or summary of inconsistency estimates from different parts of the treatment network. | 11 |
| **Risk of bias across studies** | 22 | Present results of any assessment of risk of bias across studies for the evidence base being studied. | 8 |
| **Results of additional analyses** | 23 | Give results of additional analyses, if done (e.g., sensitivity or subgroup analyses, meta-regression analyses*, alternative network geometries studied, alternative choice of prior distributions for Bayesian analyses,* and so forth). | 8,9 |
|  |  |  |  |
| **DISCUSSION** |  |  |  |
| **Summary of evidence** | 24 | Summarize the main findings, including the strength of evidence for each main outcome; consider their relevance to key groups (e.g., healthcare providers, users, and policy-makers). | 10-11 |
| **Limitations** | 25 | Discuss limitations at study and outcome level (e.g., risk of bias), and at review level (e.g., incomplete retrieval of identified research, reporting bias). *Comment on the validity of the assumptions, such as transitivity and consistency. Comment on any concerns regarding network geometry (e.g., avoidance of certain comparisons).* | 11-12 |
| **Conclusions** | 26 | Provide a general interpretation of the results in the context of other evidence, and implications for future research. | 12 |
|  |  |  |  |
| **FUNDING** |  |  |  |
| **Funding** | 27 | Describe sources of funding for the systematic review and other support (e.g., supply of data); role of funders for the systematic review. This should also include information regarding whether funding has been received from manufacturers of treatments in the network and/or whether some of the authors are content experts with professional conflicts of interest that could affect use of treatments in the network. | 2 |

PICOS = population, intervention, comparators, outcomes, study design.

* Text in italics indicates wording specific to reporting of network meta-analyses that has been added to guidance from the PRISMA statement.

† Authors may wish to plan for use of appendices to present all relevant information in full detail for items in this section.

# **Analytical approach and descriptions**

| **Analysis Category** | **Description** | |
| --- | --- | --- |
| Transitivity assessments | Transitivity assumption is required for valid indirect comparisons. Since we only included trials involving TRS individuals who received NIBS as an add-on to antipsychotics, we assumed that individuals in the included trials had an equal likelihood of being randomized to any intervention. Nonetheless, it was further explored by examining the distribution of potential effect modifiers across treatment comparisons. We explored the distribution of potential effect modifiers by visual exploration of box plots such as baseline symptom severity, illness duration, publication year, sample size, treatment duration, average participant age and proportion of female participants. We summarized and examined the number of comparisons within each category for categorical variables such as definition of treatment resistance, schizophrenia diagnosis, use of PANSS rating scales, blinding status, and sponsorship. | |
| Risk of Bias assessment | The risk of bias of the primary outcome was evaluated using RoB-2, which considers the domains of the randomization process, deviations of indented interventions, missing outcome data, measurement of the outcome and selection of the reported result. Within-study reporting bias was additionally evaluated with the RoB-MEN tool. | |
| Outcome variables | Continuous Variables (SMD) | Overall symptoms (primary outcome), Positive symptoms, Negative symptoms, Hallucination, Depression, Functioning, Quality of life, Cognition (Attention vigilance, Composite cognition, Reasoning and solving, Social cognition, Verbal learning, Visual learning, Speed of processing, Working memory) |
|  | Dichotomous Variables (OR) | Response (number of participants responding to treatment, defined by the original authors, preferably a ≥20% reduction of PANSS total scores), Dropouts (due to any reason), Side effects (Headache, Nausea, Dizziness, Sedation, Depression, Dystonia, Mania, Cognitive problems, Local reaction, Hearing problems, Myalgia, Seizures) |
| Pairwise meta-analyses | As the first step of our two-step analytical procedure, pairwise meta-analyses examined RCTs that directly compared two interventions. We conducted a random-effects meta-analysis using restricted maximum likelihood estimation of tau-squared, implemented in the meta package. | |
| Network meta-analyses | When the assumptions required for NMA were met, we conducted the NMA using a frequentist framework. NMA allows for a more comprehensive synthesis of the evidence by combining both direct and indirect comparisons across multiple interventions, thereby increasing statistical power and enabling ranking of treatments even when head-to-head trials are limited. We conducted random-effects network meta-analyses within a frequentist framework and performed fixed-effect analyses using the netmeta package. For rare events, we conducted common-effect Mantel–Haenszel network meta-analyses using netmeta’s netmetabin function. Network meta-analyses were only performed when more than ten studies were included. | |
| Assessment of heterogeneity | Between-study variance (τ²) and the I² value were presented for all NMA outcomes to facilitate a clearer evaluation of heterogeneity. Heterogeneity was further accounted for in the assessment of confidence in the evidence using the CINeMA framework. | |
| Assessment of incoherence | The consistency assumption—referring to the agreement between direct and indirect evidence—was intended to be evaluated using both local and global approaches. However, due to the lack of closed loops in the network, the planned assessment of consistency using local (SIDE test) and global methods (design-by-treatment interaction test) was largely infeasible. | |
| Subgroup analyses and meta-regressions  (They were conducted using the SMD of any NIBS versus sham as the dependent variable, and the potential effect modifier as the independent variable, as described in more detail in the meta package) | Baseline severity | Baseline symptom severity was examined as a potential effect modifier because greater severity at the start of treatment may be associated with poorer response to NIBS in TRS. Differences in baseline severity across studies may partly explain variability in treatment effects. Therefore, this variable was included in meta-regressions to explore its potential influence on outcomes. Coding of the variable: Baseline severity was considered a continuous variable, using PANSS total scores. If only BPRS total scores were reported, they were converted to PANSS scores based on an established equipercentile linking method. Studies without PANSS or BPRS scores were excluded from this analysis. |
|  | Definition of treatment-resistance | Definition of treatment-resistance was examined in subgroup analyses as more stringent definitions may reflect more severely resistant populations, which is often associated with reduced responsiveness to interventions. Analysis: Treatement-resistance definitons were grouped into three categories (see appendix 4.1). |
|  | Illness Duration | Longer duration of schizophrenia may be linked to greater treatment resistance and reduced responsiveness to NIBS. Chronic illness may be associated with more pronounced neurobiological changes, such as reduced cortical plasticity, which could affect the efficacy of NIBS interventions. Coding of the variable: Illness duration was considered a continuous variable. |
|  | Publication year | Publication year was examined as a potential effect modifier to explore whether changes over time in study design and treatment protocols influenced the reported effectiveness of NIBS. Coding of the variable: publication year was considered a continuous variable. |
|  | Sample size | Smaller studies are more prone to random error and may overestimate treatment effects due to publication bias or selective reporting. Coding of the variable: sample size was considered a continuous variable. |
|  | Treatment duration | Treatment duration was considered a potential effect modifier because longer exposure to NIBS may lead to more pronounced or sustained therapeutic effects. Coding of the variable: treatment duration was considered a continuous variable. |
|  | Sessions number | A higher number of stimulation sessions may enhance therapeutic efficacy. This is conceptually distinct from treatment duration, as treatment frequency can vary across studies. Coding of the variable: Sessions number was considered a continuous variable. |
|  | Clozapine resistance | Clozapine resistance was examined in subgroup analyses to explore whether patients who failed to respond to clozapine—a marker of more severe treatment resistance—respond differently to NIBS. Clozapine-resistant schizophrenia is often associated with greater symptom severity, longer illness duration, and poorer functional outcomes, which may affect responsiveness to NIBS interventions. Analysis: Treatement-resistance was grouped into two categories (clozapine resistance and non clozapine resistance). |
| Sensitivity analyses | Different nodes for precise NIBS protocols | A sensitivity analysis was conducted using more precise definitions of NIBS protocols, including stimulation frequency, target regions, and their combinations. It also helps to identify which specific protocols may be more effective and clinically relevant for TRS. |
|  | Double blind studies only | This sensitivity analysis tested whether the main findings remained consistent when including only double-blind studies, which are generally considered more reliable due to reduced risk of bias related to expectations or outcome assessment. |
|  | Excluding studies at high risk of bias | This sensitivity analysis examined whether the results were affected by the inclusion of studies rated as high risk of bias. By excluding these studies, the analysis focused on evidence from trials with more reliable methodological quality. |
|  | Excluding studies with implied randomization | This sensitivity analysis assessed the impact of excluding studies where randomization was not clearly described. This helped ensure that only studies with clearly reported and verifiable randomization procedures were included. |
|  | Excluding studies that did not use operationalized diagnostic criteria | This sensitivity analysis evaluated whether the results were influenced by including studies that did not apply formal diagnostic systems (e.g., DSM or ICD) to define schizophrenia. Excluding these studies ensured greater diagnostic consistency across the included trials. |
|  | Excluding studies that assumed the administration of antipsychotics | This sensitivity analysis examined the impact of excluding studies that did not explicitly confirm antipsychotic use during the trial but rather assumed it based on clinical context. |
|  | Studies in treatment-resistant positive symptom domains | This sensitivity analysis included only studies that specifically targeted treatment-resistant positive symptoms (e.g., hallucinations). This approach aimed to reduce clinical heterogeneity and ensure that the observed effects were relevant to the core symptoms defining treatment resistance in schizophrenia. |
|  | Studies with rating scales of positive symptoms used for the primary outcome | This sensitivity analysis included only studies that used validated rating scales for positive symptoms (e.g., PANSS positive subscale) as the primary outcome. This helped ensure that treatment effects were directly measured based on the symptom domain most relevant to treatment resistance. |
|  | Studies excluding imputed values | This sensitivity analysis excluded studies that relied on imputed data for the primary outcome. This was done to assess whether the main results were robust when based solely on directly reported values. |
|  | Excluding studies from mainland China | This sensitivity analysis excluded studies conducted in mainland China to assess whether the main findings were influenced by concerns about methodological quality or reporting standards raised in previous reviews. |
|  | Excluding studies in treatment-resistant positive symptom domains | This sensitivity analysis excluded studies that specifically targeted treatment-resistant positive symptoms. The goal was to assess whether the overall findings were driven by this subgroup and to examine the generalizability of the results beyond this clinical domain. |
| Small-study effects and publication bias | Small-study effects and publication bias were assessed using comparison-adjusted funnel plots. These methods help identify whether smaller studies tend to report larger treatment effects, which may suggest selective publication or reporting bias. | |
| Confidence in the evidence | Confidence in the evidence was assessed using the CINeMA framework, which considers six domains: within-study bias, across-studies bias, indirectness, imprecision, heterogeneity, and incoherence. This approach allows for a structured and transparent evaluation of how much trust can be placed in each network estimate. Ratings were based on the judgment across all six domains for each comparison. | |

# **Database search strategy**

1. Systematic searches were conducted through February 2023 across multiple databases including CENTRAL, ClinicalTrials.gov, Embase, MEDLINE, PsycINFO, PubMed, and WHO ICTRP, without limitations on date/time, language, document type, or publication status, utilizing the search strategy that is detailed subsequently.

2. Subsequent updates to the search were executed within the study-specific registry maintained by the Cochrane Schizophrenia Group, extending up to July 2025.

3. Searches were conducted in the Chinese databases CNKI, Wanfang, and CBM starting in March 2023. These searches were updated in October 2024. Detailed information regarding the search strategy is provided below.

## **3.1 Systematic searches**

**CENTRAL**

#1 ([mh "Schizophrenia Spectrum and Other Psychotic Disorders"] OR [mh Schizophrenia] OR [mh ^"Psychotic Disorders"] OR [mh "Schizotypal Personality Disorder"] OR (Schizophreni* OR Schizoaffective* OR Schizo-Affective* OR Schizotyp* OR Psychotic* OR Psychosis OR Psychoses):ti,ab) AND ([mh ^"Convulsive Therapy"] OR [mh "Electroconvulsive Therapy"] OR [mh ^"Electric Stimulation Therapy"] OR [mh "Transcranial Direct Current Stimulation"] OR [mh Electroshock] OR [mh "Magnetic Field Therapy"] OR [mh "Transcranial Magnetic Stimulation"] OR [mh "Deep Brain Stimulation"] OR (Brain Depth Stimulation* OR Brain Stimulation* OR Convulsive Therap* OR Current Stimulation OR Electric Convuls* OR Electric Field Stimulation* OR Electric Stimulation* OR Electrical Stimulation* OR Electro Stimulation* OR Electroconvuls* OR Electromagnetic Therap* OR Electroshock* OR Electrostimul* OR Electrotherap* OR Magnetic Field Therap* OR Magnetic Seizure* OR Magnetic Stimulation* OR Magnetic Therap* OR Magnetotherap* OR Random Noise Stimulation* OR Shock* OR Theta Burst Stimulation* OR TMS OR aTMS OR dTMS OR pTMS OR rTMS OR sTMS OR tRNS OR tDCS OR tACS OR ECT OR tES OR TBS OR cTBS OR iTBS OR LCE OR MST OR ECS Therap*):ti,ab)

**ClinicalTrials.gov**

*Condition or disease:* Schizophrenia OR Schizotypal OR Schizoaffective OR Psychotic OR Psychosis OR Psychoses
*Study type:* Interventional Studies (Clinical Trials)
*Other terms:* Randomized OR Randomised OR RCT

*Intervention/treatment:* Electroconvulsive OR Stimulation OR Electromagnetic OR Magnetic OR TMS OR dTMS OR rTMS OR tDCS OR tACS OR ECT OR tES OR TBS OR iTBS OR MST

**Embase**

1 Randomized controlled trial/ or Controlled clinical study/ or randomization/ or intermethod comparison/ or double blind procedure/ or human experiment/ or (random$ or placebo or (open adj label) or ((double or single or doubly or singly) adj (blind or blinded or blindly)) or parallel group$1 or crossover or cross over or ((assign$ or match or matched or allocation) adj5 (alternate or group$1 or intervention$1 or patient$1 or subject$1 or participant$1)) or assigned or allocated or (controlled adj7 (study or design or trial)) or volunteer or volunteers).ti,ab. or (compare or compared or comparison or trial).ti. or ((evaluated or evaluate or evaluating or assessed or assess) and (compare or compared or comparing or comparison)).ab. (6211404)

2 (random$ adj sampl$ adj7 ("cross section$" or questionnaire$1 or survey$ or database$1)).ti,ab. not (comparative study/ or controlled study/ or randomi?ed controlled.ti,ab. or randomly assigned.ti,ab.) (9365)

3 Cross-sectional study/ not (randomized controlled trial/ or controlled clinical study/ or controlled study/ or (randomi?ed controlled or control group$1).ti,ab.) (338931)

4 (((case adj control$) and random$) not randomi?ed controlled).ti,ab. (21251)

5 (Systematic review not (trial or study)).ti. (251368)

6 (nonrandom$ not random$).ti,ab. (18731)

7 ("Random field$" or (random cluster adj3 sampl$)).ti,ab. (4426)

8 (review.ab. and review.pt.) not trial.ti. (1091007)

9 "we searched".ab. and (review.ti. or review.pt.) (48390)

10 ("update review" or (databases adj4 searched)).ab. (60348)

11 (rat or rats or mouse or mice or swine or porcine or murine or sheep or lambs or pigs or

piglets or rabbit or rabbits or cat or cats or dog or dogs or cattle or bovine or monkey or monkeys or trout or marmoset$1).ti. and animal experiment/ (1214398)

12 Animal experiment/ not (human experiment/ or human/) (2550295)

13 or/2-12 (4262395)

14 1 not 13 (5485863)

1. xp schizophrenia spectrum disorder/ or paranoid psychosis/ or exp Schizophrenia/ or

schizoaffective psychosis/ or schizotypal personality disorder/ or brief psychotic disorder/ or (Schizophreni* or Schizoaffective* or Schizo-Affective* or Schizotyp* or Psychotic* or Psychosis or Psychoses).ti,ab. (296055)

16 Electroconvulsive Therapy/ or Electrotherapy/ or Brain Depth Stimulation/ or exp Transcranial Electrical Stimulation/ or Transcranial Alternating Current Stimulation/ or Transcranial Direct Current Stimulation/ or Transcranial Random Noise Stimulation/ or exp Transcranial Magnetic Stimulation/ or Magnetotherapy/ or Electrostimulation/ or (Brain Depth Stimulation* or Brain Stimulation* or Convulsive Therap* or Current Stimulation or Electric Convuls* or Electric Field Stimulation* or Electric Stimulation* or Electrical Stimulation* or Electro Stimulation* or Electroconvuls* or Electromagnetic Therap* or Electroshock* or Electrostimul* or Electrotherap* or Magnetic Field Therap* or Magnetic Seizure* or Magnetic Stimulation* or Magnetic Therap* or Magnetotherap* or Random Noise Stimulation* or Shock* or Theta Burst Stimulation* or TMS or aTMS or dTMS or pTMS or rTMS or sTMS or tRNS or tDCS or tACS or ECT or tES or TBS or cTBS or iTBS or LCE or MST or ECS Therap*).

**MEDLINE**

1 "Schizophrenia Spectrum and Other Psychotic Disorders"/ or exp Schizophrenia/ or Psychotic Disorders/ or Schizotypal Personality Disorder/ or (Schizophreni* or Schizoaffective* or Schizo- Affective* or Schizotyp* or Psychotic* or Psychosis or Psychoses).ti,ab. (221380)
2 Convulsive Therapy/ or exp Electroshock/ or Transcranial Direct Current Stimulation/ or Electroconvulsive Therapy/ or Electric Stimulation Therapy/ or exp Magnetic Field Therapy/ or Transcranial Magnetic Stimulation/ or Deep Brain Stimulation/ or (Brain Depth Stimulation* or Brain Stimulation* or Convulsive Therap* or Current Stimulation or Electric Convuls* or Electric Field Stimulation* or Electric Stimulation* or Electrical Stimulation* or Electro Stimulation* or Electroconvuls* or Electromagnetic Therap* or Electroshock* or Electrostimul* or Electrotherap* or Magnetic Field Therap* or Magnetic Seizure* or Magnetic Stimulation* or Magnetic Therap* or Magnetotherap* or Random Noise Stimulation* or Shock* or Theta Burst Stimulation* or TMS or aTMS or dTMS or pTMS or rTMS or sTMS or tRNS or tDCS or tACS or ECT or tES or TBS or cTBS or iTBS or LCE or MST or ECS Therap*).ti,ab. (387113)
3 ((Randomized Controlled Trial or Controlled Clinical Trial).pt. or (Randomi?ed or Placebo or Randomly or Trial or Groups).ab. or Drug Therapy.fs.) not (exp Animals/ not Humans.sh.) (4893081)

**PsycINFO**

1 exp Schizophrenia/ or Psychosis/ or Schizotypal Personality Disorder/ or Schizotypy/ or Schizophreniform Disorder/ or Schizoaffective Disorder/ or (Schizophreni* or Schizoaffective* or Schizo-Affective* or Schizotyp* or Psychotic* or Psychosis or Psychoses).ti,ab. (187305)
2 Brain Stimulation/ or Electrical Stimulation/ or exp Electrical Brain Stimulation/ or Deep Brain Stimulation/ or Transcranial Direct Current Stimulation/ or exp Electroconvulsive Shock/ or Electroconvulsive Shock Therapy/ or Shock Therapy/ or Transcranial Magnetic Stimulation/ or Transcranial Magnetic Stimulation/ or (Brain Depth Stimulation* or Brain Stimulation* or Convulsive Therap* or Current Stimulation or Electric Convuls* or Electric Field Stimulation* or Electric Stimulation* or Electrical Stimulation* or Electro Stimulation* or Electroconvuls* or Electromagnetic Therap* or Electroshock* or Electrostimul* or Electrotherap* or Magnetic Field Therap* or Magnetic Seizure* or Magnetic Stimulation* or Magnetic Therap* or Magnetotherap* or Random Noise Stimulation* or Shock* or Theta Burst Stimulation* or TMS or aTMS or dTMS or pTMS or rTMS or sTMS or tRNS or tDCS or tACS or ECT or tES or TBS or cTBS or iTBS or LCE or MST or ECS Therap*).ti,ab. (75564)
3 exp Clinical Trials/ or Placebo/ or (random* or sham or placebo* or ((singl* or doubl*) adj (blind* or dumm* or mask*)) or ((tripl* or trebl*) adj (blind* or dumm* or mask*)) or (control* adj3 (study or studies or trial* or group*)) or Nonrandom* or non random* or non-random* or quasi-random* or quasirandom* or allocated or ((open label or open-label) adj5 (study or studies or trial*)) or ((equivalence or superiority or non-inferiority or noninferiority) adj3 (study or studies or trial*)) or ((pragmatic or practical) adj3 trial*) or ((quasiexperimental or quasi- experimental) adj3 (study or studies or trial*)) or (phase adj3 (III or "3") adj3 (study or studies or trial*))).ti,ab,hw. (396089)

**PubMed**

("Schizophrenia Spectrum and Other Psychotic Disorders"[MH] OR Schizophrenia[MH] OR Psychotic Disorders[MH:NoExp] OR Schizotypal Personality Disorder[MH] OR Schizophreni*[TIAB] OR Schizoaffective[TIAB] OR Schizo-Affective[TIAB] OR Schizotyp*[TIAB] OR Psychotic*[TIAB] OR Psychosis[TIAB] OR Psychoses[TIAB] OR Chronic Psychiatric Illness*[TIAB] OR Chronic Psychiatric Disorder*[TIAB] OR Chronic Mental Illness*[TIAB] OR Chronic Mental Disorder*[TIAB] OR Severe Psychiatric Illness*[TIAB] OR Severe Psychiatric Disorder*[TIAB] OR Severe Mental Illness*[TIAB] OR Severe Mental Disorder*[TIAB] OR Serious Psychiatric Illness*[TIAB] OR Serious Psychiatric Disorder*[TIAB] OR Serious Mental Illness*[TIAB] OR Serious Mental Disorder*[TIAB]) AND (Convulsive Therapy[MH:NoExp] OR Electroconvulsive Therapy[MH] OR Electric Stimulation Therapy[MH:NoExp] OR Transcranial Direct Current Stimulation[MH] OR Electroshock[MH] OR Magnetic Field Therapy[MH] OR Transcranial Magnetic Stimulation[MH] OR Deep Brain Stimulation[MH] OR Brain Depth Stimulation*[TIAB] OR Brain Stimulation*[TIAB] OR Convulsive Therap*[TIAB] OR Current Stimulation[TIAB] OR Electric Convuls*[TIAB] OR Electric Field Stimulation*[TIAB] OR Electric Stimulation*[TIAB] OR Electrical Stimulation*[TIAB] OR Electro Stimulation*[TIAB] OR Electroconvuls*[TIAB] OR Electromagnetic Therap*[TIAB] OR Electroshock*[TIAB] OR Electrostimul*[TIAB] OR Electrotherap*[TIAB] OR Magnetic Field Therap*[TIAB] OR Magnetic Seizure*[TIAB] OR Magnetic Stimulation*[TIAB] OR Magnetic Therap*[TIAB] OR Magnetotherap*[TIAB] OR Random Noise Stimulation*[TIAB] OR Shock*[TIAB] OR Theta Burst Stimulation*[TIAB] OR TMS[TIAB] OR aTMS[TIAB] OR dTMS[TIAB] OR pTMS[TIAB] OR rTMS[TIAB] OR sTMS[TIAB] OR tRNS[TIAB] OR tDCS[TIAB] OR tACS[TIAB] OR ECT[TIAB] OR tES[TIAB] OR TBS[TIAB] OR cTBS[TIAB] OR iTBS[TIAB] OR LCE[TIAB] OR MST[TIAB] OR ECS Therap*[TIAB]) AND (Randomized Controlled Trial[PT] OR Controlled Clinical Trial[PT] OR Pragmatic Clinical Trial[PT] OR Randomized[TIAB] OR Randomised[TIAB] OR Placebo[TIAB] OR Randomly[TIAB] OR Trial[TIAB] OR Groups[TIAB]) NOT MEDLINE[SB]

**WHO ICTRP**

(Schizophrenia OR Schizotypal OR Schizoaffective Psychotic or Psychosis or Psychoses) AND (Stimulation OR Electroconvulsive OR Shock OR Convulsive OR Electromagnetic OR Electroshock OR Electrostimulation OR Electrotherapy OR Magnetic OR Magnetotherapy OR TMS OR aTMS OR dTMS OR pTMS OR rTMS OR sTMS OR tRNS OR tDCS OR tACS OR ECT OR tES OR TBS OR cTBS OR iTBS OR LCE OR MST OR ECS)

## **3.2 Cochrane Schizophrenia Group searches**

We searched the Cochrane Schizophrenia Group register on 10.06.2024 (from the inception of the register to the last update of the register on 24.01.2024), on 11.03.2025 (last update of the register on 13.01.2025) and on 20.08.2025 (last update of the register on 13.07.2025) for studies investigating the following interventions:

Convulsive Photoshock Therapy {XXX}

ECT: Electroconvulsive Therapy {XXX}

Electrosleep Therapy {XXX} {Stopped}

Theta Burst Repetitive Transcranial Magnetic Stimulation (Intermittent) {STIM}

rTMS: Repetitive Transcranial Magnetic Stimulation {STIM}

Sub-Convulsive Photoshock {XXX}

Subliminal Symbiotic Stimulation {STIM}

tDCS: Transcranial Direct Current Stimulation {STIM}

Ultrasound Detector {DEV}

Stimulation {STIM}

t-VNS: Transcutaneous Vagus Nerve Stimulation {STIM}

Theta Burst Repetitive Transcranial Magnetic Stimulation {STIM}

Theta Burst Repetitive Transcranial Magnetic Stimulation (Continuous) {STIM}

Deep Brain Stimulation {STIM}

tRNS: Transcranial Random Noise Stimulation {STIM}

Theta Burst Repetitive Transcranial Magnetic Stimulation Protocols (Guidelines) {STIM}

nTMS: Navigated Transcranial Magnetic Stimulation {STIM}

Deep Transcranial Magnetic Stimulation {STIM}

Sensory Caloric Vestibular Simulation {STIM}

tACS: Transcranial Alternating Current Stimulation {STIM}

Magnetic Seizure Therapy {RPOCEDURE}

Sub-Convulsive Therapy {XXX}

tGACS: Transcranial Gamma Alternating Current Stimulation {STIM}

Gamma Transcranial Direct Current Stimulation (tDCS) {STIM}

Non-Convulsively Electroconvulsive Therapy {ECT}

TMS: Transcranial Magnetic Stimulation {STIM}

VNS: Vagal Transcutaneous Nerve Stimulation {STIM}

ECT: Electroconvulsive Therapy {XXX} (Hybrid)

Ultrasound

dTMS: Deep Transcranial Magnetic Stimulation {STIM}

Brainstem Neuromodulation

Pulsed Low-Intensity Focused Ultrasound

Auditory Stimulation (Closed-Loop) {STIM}

Non-Invasive Neuromodulation {XXX}

rTUS: Repetitive Transcranial Ultrasound Stimulation {STIM}

## **3.3 Chinese databases systematic searches**

**CNKI**

(SU%=精神分裂症+精神分裂+偏执型障碍+分裂型障碍+分裂型精神障碍+分裂情感性障碍+精神病+精神疾病 OR TKA=精神分裂症+精神分裂+偏执型障碍+分裂型障碍+分裂型精神障碍+分裂情感性障碍+精神病+精神疾病) AND (SU%=侵入性脑刺激+ NIBS+非侵入脑刺激+无创脑刺激+无创性脑刺激+非侵入性神经刺激+非侵入性神经调节+无创神经调节+经颅磁刺激+ Rtms+ aTMS+ pTMS+ sTMS+ dTMS+经颅直流电刺激+经颅电刺激+tDCS+经颅交流电刺激+经颅随机电刺激+tACS+经颅微电流刺激+经颅超声刺激+电刺激+磁疗+电磁疗法+磁刺激+磁休克+磁痉挛+磁抽搐+电休克+电惊厥+惊厥疗法+电抽搐+MECT+低电荷电疗+低电荷电疗法+低电量治疗+低电量电抽搐+经颅随机噪声+经颅随机噪音+ tRNS+经颅磁治疗+ Theta节律刺激+Theta波刺激+ theta脉冲刺激+iTBS+ cTBS+惊厥疗法+模式化刺激+θ短阵快速脉冲刺激+θ突发刺激+θ脉冲刺激+爆发式磁刺激+爆发式刺激+θ爆发刺激 OR TKA=侵入性脑刺激+ NIBS+非侵入脑刺激+无创脑刺激+无创性脑刺激+非侵入性神经刺激+非侵入性神经调节+无创神经调节+经颅磁刺激+ Rtms+ aTMS+ pTMS+ sTMS+ dTMS+经颅直流电刺激+经颅电刺激+tDCS+经颅交流电刺激+经颅随机电刺激+tACS+经颅微电流刺激+经颅超声刺激+电刺激+磁疗+电磁疗法+磁刺激+磁休克+磁痉挛+磁抽搐+电休克+电惊厥+惊厥疗法+电抽搐+MECT+低电荷电疗+低电荷电疗法+低电量治疗+低电量电抽搐+经颅随机噪声+经颅随机噪音+ tRNS+经颅磁治疗+ Theta节律刺激+Theta波刺激+ theta脉冲刺激+iTBS+ cTBS+惊厥疗法+模式化刺激+θ短阵快速脉冲刺激+θ突发刺激+θ脉冲刺激+爆发式磁刺激+爆发式刺激+θ爆发刺激) AND (SU%=随机+盲法+双盲+单盲+三盲+交叉+RCT OR TKA=随机+盲法+双盲+单盲+三盲+交叉+RCT)

**Wanfang**

主题:(精神分裂 OR 偏执型障碍 OR 分裂型障碍 OR 分裂情感性障碍 OR "精神病" OR "精神疾病") and 主题:(侵入性脑刺激 OR NIBS OR 非侵入脑刺激 OR 无创脑刺激 OR 非侵入性神经刺激 OR 非侵入性神经调节 OR 无创神经调节 OR Rtms OR aTMS OR pTMS OR sTMS OR dTMS OR tDCS OR tACS OR "经颅微电流刺激" OR "经颅超声刺激" OR "电刺激" OR "磁疗" OR "电磁疗法" OR "磁刺激" OR "磁休克" OR "磁痉挛" OR "磁抽搐" OR "电休克" OR "电惊厥" OR "惊厥疗法" OR "电抽搐" OR MECT OR "低电荷电疗" OR "低电量治疗" OR "低电量电抽搐" OR "经颅随机噪声" OR "经颅随机噪音" OR tRNS OR "经颅磁治疗" OR "Theta节律刺激" OR "Theta波刺激" OR "theta脉冲刺激" OR iTBS OR cTBS OR "惊厥疗法" OR "模式化刺激" OR "θ短阵快速脉冲刺激" OR "θ突发刺激" OR "θ脉冲刺激" OR "爆发式磁刺激" OR "爆发式刺激" OR "θ爆发刺激") and 主题:("随机" OR "盲法" OR 双盲 OR 单盲 OR 三盲 OR 交叉 OR RCT)

**CBM**

( "精神分裂症"[常用字段:智能] OR "精神分裂"[常用字段:智能] OR "偏执型障碍"[常用字段:智能] OR "分裂型障碍"[常用字段:智能] OR "分裂型精神障碍"[常用字段:智能] OR "分裂情感性障碍"[常用字段:智能] OR "精神病"[常用字段:智能] OR "精神疾病"[常用字段:智能]) AND ( "侵入性脑刺激"[常用字段:智能] OR " NIBS"[常用字段:智能] OR "非侵入脑刺激"[常用字段:智能] OR "非侵入脑刺激"[常用字段:智能] OR "无创脑刺激"[常用字段:智能] OR "无创性脑刺激"[常用字段:智能] OR "非侵入性神经刺激"[常用字段:智能] OR "非侵入性神经调节"[常用字段:智能] OR "无创神经调节"[常用字段:智能] OR "经颅磁刺激"[常用字段:智能] OR "Rtms"[常用字段:智能] OR "aTMS"[常用字段:智能] OR "pTMS"[常用字段:智能] OR "sTMS"[常用字段:智能] OR "dTMS"[常用字段:智能] OR "经颅直流电刺激"[常用字段:智能] OR "经颅电刺激"[常用字段:智能] OR "tDCS"[常用字段:智能] OR "经颅交流电刺激"[常用字段:智能] OR "经颅随机电刺激"[常用字段:智能] OR "tACS"[常用字段:智能] OR "经颅微电流刺激"[常用字段:智能] OR "经颅超声刺激"[常用字段:智能] OR "电刺激"[常用字段:智能] OR "磁疗"[常用字段:智能] OR "电磁疗法"[常用字段:智能] OR "磁刺激"[常用字段:智能] OR "磁休克"[常用字段:智能] OR "磁痉挛"[常用字段:智能] OR "磁抽搐"[常用字段:智能] OR "电休克"[常用字段:智能] OR "电惊厥"[常用字段:智能] OR "惊厥疗法"[常用字段:智能] OR "电抽搐"[常用字段:智能] OR "MECT"[常用字段:智能] OR "低电荷电疗"[常用字段:智能] OR "低电荷电疗法"[常用字段:智能] OR "低电量治疗"[常用字段:智能] OR "低电量电抽搐"[常用字段:智能] OR "经颅随机噪声"[常用字段:智能] OR "经颅随机噪音"[常用字段:智能] OR "tRNS"[常用字段:智能] OR "经颅磁治疗"[常用字段:智能] OR " Theta节律刺激"[常用字段:智能] OR "Theta波刺激"[常用字段:智能] OR " theta脉冲刺激"[常用字段:智能] OR "iTBS"[常用字段:智能] OR "cTBS"[常用字段:智能] OR "惊厥疗法"[常用字段:智能] OR "模式化刺激"[常用字段:智能] OR "θ短阵快速脉冲刺激"[常用字段:智能] OR "θ突发刺激"[常用字段:智能] OR "θ脉冲刺激"[常用字段:智能] OR "爆发式磁刺激"[常用字段:智能] OR "爆发式刺激"[常用字段:智能] OR "θ爆发刺激"[常用字段:智能]) AND ("随机对照试验"[不加权:扩展] OR "随机对照试验(主题)"[不加权:扩展] OR "非随机对照试验(主题)"[不加权:扩展] OR "随机"[常用字段:智能] OR "盲法"[常用字段:智能] OR "双盲"[常用字段:智能] OR "单盲"[常用字段:智能] OR "三盲"[常用字段:智能] OR "交叉"[常用字段:智能] OR "RCT"[常用字段:智能])

# **4. Study selection**

## **4.1 Criteria for Treatment-Resistant Categorization**

| **Resistant Treatment-resistant Category** | **Category Description** | **Studies within the category** |
| --- | --- | --- |
| **Minimum cut-off: 1** | Lack of response to antipsychotics without a specification and studies that do not fit into the other two specified categories. | Argawal 1985, Koops 2016, Klein 1999, Bose 2018, Brunelin 2006, Brunelin 2012, Chang 2018, Hajak 2004, Holi 2004, Rosenberg 2012, Li 2019, Mao 2023, Mahato 2025, McIntosh 2004, Weijer 2014, Dollfus 2018, Parlikar 2025, Plewnia 2014, Plewnia 2025, Potapov 2022, Suonmaa 2021, Tuppurainen 2020, Hu 2019, Jiang 2017, Kantrowitz 2019, Li 2015, Lu 2014, Pu 2013, Quan 2012, Ren 2010, Tyagi 2022, Wang 2021, Xu 2011, Yang 2022, Yue 2013, Zhang 2014, Zhang 2016, Zhu 2013, Donde 2023, Parlikar 2023, Liu 2017, Hua 2024 |
| **Moderate cut-off: 2** | Lack of response despite at least two antipsychotics. | Bais 2014, Blumberger 2012, Fitzgerald 2005, Fitzgerald 2014a, Fitzgerald 2014b, Fröhlich 2015, Gornerova 2023, Hoffman 2005, Jandl 2006, Jesus 2011, Kim 2014, Kimura 2016, Kindler 2013a, Klirova 2013, Goswami 2003, Jandl 2006, Cheng 2013, Gao 2009, Koops 2018, Laurin 2020, Lee 2005, Lindenmayer 2019, Marquardt 2022, Mellin 2018, Melzer-Ribeiro 2017, Paillere-Martinot 2017, Petrides 2015, Rosa 2007, Slotema 2011, Wang 2025, Xie 2023, An 2011, Cheng 2013, Gao 2009, Xie 2024 |
| **Optimum cut-off: 3** | Lack of response despite at least three antipsychotics, or treatment resistance per Kane's criteria, meaning a lack of response despite adequate doses and adequate duration of two antipsychotics, with at least one of them being prospective. It also involves persistant moderate symptoms, measured with rating scales at the end of the trial. | Chauhan 2021, Xu 2020, Sun 2018, Wang 2016, Melzer 2023, Chen 2024 |

## **4.2 Communication with authors**

| **Study_name** | **contacted** | **Replied** | **Provided data** | **Notes** |
| --- | --- | --- | --- | --- |
| Argawal 1985 | no | no | no | No valid email address found |
| Bais 2014 | yes | yes | no |  |
| Blumberger 2012 | yes | yes | yes |  |
| Bose 2018 | yes | yes | yes |  |
| Chauhan 2021 | yes | no | no reply |  |
| Fitzgerald 2005 | yes | no | no reply |  |
| Fitzgerald 2014a | yes | no | no reply |  |
| Fitzgerald 2014b | yes | no | no reply |  |
| Fröhlich 2015 | yes | yes | no |  |
| Gornerova 2023 | yes | yes | no |  |
| Hoffman 2005 | yes | yes | no |  |
| Jandl 2006 | yes | yes | yes |  |
| Jesus 2011 | yes | no | no reply |  |
| Kantrowitz 2019 | yes | yes | no |  |
| Kim 2014 | yes | yes | yes |  |
| Kimura 2016 | yes | no | no reply |  |
| Kindler 2013a | yes | yes | no |  |
| Klirova 2013 | yes | yes | yes |  |
| Koops 2016 | yes | yes | yes |  |
| Brunelin 2006 | yes | yes | no |  |
| Brunelin 2012 | yes | yes | yes |  |
| Chang 2018 | yes | yes | yes |  |
| Goswami 2003 | yes | no | no reply |  |
| Holi 2004 | no | no | no | No valid email address found |
| Jandl 2006 | yes | yes | yes |  |
| Rosenberg 2012 | yes | no | no reply |  |
| Cheng 2013 | yes | no | no reply | Chinese database study |
| Gao 2009 | yes | no | no reply | Chinese database study |
| Koops 2018 | yes | yes | yes |  |
| Lee 2005 | yes | no | no reply |  |
| Li 2019 | yes | yes | yes | Chinese study |
| Lindenmayer 2019 | yes | yes | no |  |
| Marquardt 2022 | yes | no | no reply |  |
| McIntosh 2004 | yes | yes | yes |  |
| Mellin 2018 | yes | yes | no |  |
| Melzer-Ribeiro 2017 | yes | yes | no |  |
| Paillere-Martinot 2017 | yes | yes | yes |  |
| Petrides 2015 | yes | yes | no | The authors replied that they don't have access to data |
| Rosa 2007 | yes | no | no |  |
| Slotema 2011 | yes | yes | yes |  |
| Tyagi 2022 | yes | yes | no |  |
| Weijer 2014 | yes | no | no reply |  |
| Xie 2023 | yes | yes | yes | Chinese study |
| Dollfus 2018 | yes | yes | yes |  |
| Laurin 2020 | yes | yes | yes |  |
| Plewnia 2014 | yes | no | no reply |  |
| Potapov 2022 | yes | yes | yes |  |
| Suonmaa 2021 | no | no | no | No valid email address found |
| Tuppurainen 2020 | yes | yes | yes |  |
| Hua 2024 | yes | yes | no | Chinese study |
| Xie 2024 | yes | no | no | Chinese study |
| An 2011 | yes | no | no reply | Chinese dataset study |
| Cheng 2013 | yes | no | no reply | Chinese dataset study |
| Gao 2009 | yes | no | no reply | Chinese dataset study |
| Hu 2019 | yes | no | no reply | Chinese dataset study |
| Jiang 2017 | no | no | no | No valid email address found, Chinese dataset study |
| Li 2015 | yes | no | no reply | Chinese dataset study |
| Lu 2014 | yes | no | no reply | Chinese dataset study |
| Pu 2013 | yes | no | no reply | Chinese dataset study |
| Quan 2012 | yes | no | no reply | Chinese dataset study |
| Ren 2010 | yes | no | no reply | Chinese dataset study |
| Sun 2018 | yes | no | no reply | Chinese dataset study |
| Wang 2016 | yes | no | no reply | Chinese dataset study |
| Wang 2021 | yes | no | no reply | Chinese dataset study |
| Xu 2011 | yes | no | no reply | Chinese dataset study |
| Xu 2020 | yes | no | no reply | Chinese dataset study |
| Yang 2022 | yes | no | no reply | Chinese dataset study |
| Yue 2013 | no | no | no | No valid email address found, Chinese dataset study |
| Zhang 2014 | yes | no | no reply | Chinese dataset study |
| Zhang 2016 | no | no | no | No valid email address found, Chinese dataset study |
| Zhu 2013 | yes | no | no reply | Chinese dataset study |
| Donde 2023 | yes | yes | yes |  |
| Melzer 2023 | yes | yes | yes |  |
| Parlikar 2023 | yes | no | no reply |  |
| Chen 2024 | no | no | no | No valid email address found, Chinese dataset study |
| Liu 2017 | no | no | no | No valid email address found, Chinese dataset study. Due to the implausibility of the data presented in the study, we attempted all methods to contact the authors and the journal editors. However, we were unable to establish communication to verify the authenticity of the data. Therefore, we have decided to exclude this study from our analysis. |

# **5. Description and references of included studies**

| **TMS studies** | | | | | | |
| --- | --- | --- | --- | --- | --- | --- |
| **rTMS studies** | | | | | | |
| **Study name** | **Study site** | **Intervention comparision** | **Study design** | **Trial duration (weeks)** | **Treatment-resistant definition level*** | **Stimulation paradigms** |
| An 2011^1^ | Chinese mainland | rTMS (n=26), SHM (n=10) | DB | 1.7 | 2 | Low frenquency (1 Hz) rTMS was applied to the dualdorsolateral prefrontal, or the left temporoparietal. |
| Bais 2014^2^ | Netherlands | rTMS (n=35), SHM (n=16) | DB | 1.14 | 2 | Low frenquency (1 Hz) rTMS was applied to the left or bilateral temporo-parietal junctionarea. |
| Blumberger 2012^3^ | Canada | rTMS (n=34), SHM (n=17) | DB | 4 | 2 | Low frenquency (1 Hz) rTMS was applied to the Heschl’s gyrus. |
| Brunelin 2006^4^ | France | rTMS (n=14), SHM (n=10) | DB | 0.7 | 1 | Low frenquency (1 Hz) rTMS was applied to the left temporoparietal cortex. |
| Cheng 2013^5^ | Chinese mainland | rTMS (n=23), SHM (n=24) | DB | 6 | 2 | Low frenquency (1 Hz) rTMS was applied to the left temporoparietal cortex. |
| Dollfus 2018^6^ | France | rTMS (n=35), SHM (n=39) | DB | 0.29 | 1 | High frenquency (20 Hz) rTMS was applied to the left temporal region using neuronavigation. |
| Fitzgerald 2005^7^ | Australia | rTMS (n=17), SHM (n=16) | DB | 2 | 2 | Low frenquency (1 Hz) rTMS was applied to the left temporoparietal cortex. |
| Gao 2009^8^ | Chinese mainland | rTMS (n=23), SHM (n=23) | DB | 2 | 2 | Low frenquency (1 Hz) rTMS was applied to the left temporoparietal cortex. |
| Gornerova 2023^9^ | Czech Republic | rTMS (n=10), SHM (n=9) | DB | 2 | 2 | Low frenquency (0.9 Hz) rTMS was applied to the left temporo-parietal region. |
| Hajak 2004^10^ | Germany | rTMS (n=10), SHM (n=10) | SB | 2 | 1 | High frenquency (10 Hz) rTMS was applied to the left dorsolateral prefrontal cortex. |
| Hoffman 2005^11^ | USA | rTMS (n=27), SHM (n=23) | DB | 1.3 | 2 | Low frenquency (1 Hz) rTMS was applied to the left temporoparietal cortex. |
| Holi 2004^12^ | Finland | rTMS (n=11), SHM (n=11) | DB | 2 | 1 | High frenquency (10 Hz) rTMS was applied to the left prefrontal cortex. |
| Hu 2019^13^ | Chinese mainland | rTMS (n=25), SHM (n=25) | DB | 4 | 1 | Low frenquency (1 Hz) rTMS was applied to the left temporoparietal cortex. |
| Jandl 2006^14^ | Germany | rTMS (n=6), SHM (n=8) | DB | 0.7 | 2 | Low frenquency (1 Hz) rTMS was applied to the left and right temporo-parietal cortex. |
| Jesus 2011^15^ | Brazil | rTMS (n=8), SHM (n=9) | DB | 4 | 2 | Low frenquency (1 Hz) rTMS was applied to the left temporal-parietal cortex. |
| Jiang 2017^16^ | Chinese mainland | rTMS (n=30), SHM (n=30) | DB | 6 | 1 | Low frenquency (1 Hz) rTMS was applied to the left dorsolateral prefrontal cortex. |
| Kim 2014^17^ | South Korea | rTMS (n=16), SHM (n=6) | DB | 1 | 2 | Low frenquency (1 Hz) or high frenquency (20 Hz) rTMS was applied to the temporoparietal area, or high frequency (20 Hz) rTMS was applied to the Broca’s area. |
| Kimura 2016^18^ | Japan | rTMS (n=16), SHM (n=14) | DB | 0.3 | 2 | High frenquency (20 Hz) rTMS was applied to the left temporoparietal cortex. |
| Klirova 2013^19^ | Czech Republic | rTMS (n=6), SHM (n=9) | DB | 1.43 | 2 | Low frenquency (0.9 Hz) rTMS was applied to the left temporo-parietal region. |
| Klein 1999^20^ | Israel | rTMS (n=16), SHM (n=15) | DB | 2 | 1 | Low frenquency (1 Hz) rTMS was applied to the right prefrontal cortex. |
| Laurin 2020^21^ | France | rTMS (n=7), SHM (n=8) | DB | 0.7 | 1 | Low frenquency (1 Hz) rTMS was applied to the left superior temporal sulcus. |
| Lee 2005^22^ | South Korea | rTMS (n=25), SHM (n=14) | DB | 1.43 | 2 | Low frenquency (1 Hz) rTMS was applied to the left or right temporoparietal cortex. |
| Li 2015^23^ | Chinese mainland | rTMS (n=26), SHM (n=24) | DB | 2 | 1 | Low frenquency (1 Hz) rTMS was applied to the left temporoparietal cortex. |
| Lu 2014^24^ | Chinese mainland | rTMS (n=16), SHM (n=16) | DB | 2 | 1 | High frenquency (20 Hz) rTMS was applied to the bilateral dorsolateral prefrontal cortex. |
| Mao 2023^25^ | Chinese mainland | rTMS (n=29), SHM (n=24) | DB | 4 | 1 | High frenquency (10 Hz) rTMS was applied to the left dorsolateral prefrontal cortex. |
| McIntosh 2004^26^ | UK | rTMS (n=8), SHM (n=8) | DB | 0.57 | 1 | Low frenquency (1 Hz) rTMS was applied to the left temporoparietal cortex. |
| Paillere-Martinot 2017^27^ | France | rTMS (n=15), SHM (n=13) | DB | 2 | 2 | Low frenquency (1 Hz) rTMS was applied to a targeted a language perception area individually determined using functional magnetic resonance imaging and a language recognition task. |
| Pu 2013^28^ | Chinese mainland | rTMS (n=40), SHM (n=40) | DB | 1.4 | 1 | Low frenquency (1 Hz) rTMS was applied to the left temporoparietal cortex and the right temporoparietal cortex. |
| Quan 2012^29^ | Chinese mainland | rTMS (n=88), SHM (n=34) | DB | 6 | 1 | Low frenquency (1 Hz) rTMS was applied to the left dorsolateral prefrontal cortex. |
| Ren 2010^30^ | Chinese mainland | rTMS (n=12), SHM (n=13) | DB | 2 | 1 | Low frenquency (1 Hz) rTMS was applied to the bilateral dorsolateral prefrontal cortex. |
| Rosa 2007^31^ | Brazil | rTMS (n=6), SHM (n=5) | DB | 1.43 | 2 | Low frenquency (1 Hz) rTMS was applied to the left temporoparietal cortex. |
| Slotema 2011^32^ | Netherlands | rTMS (n=42), SHM (n=20) | DB | 3 | 2 | Low frenquency (1 Hz) rTMS was applied to the left temporoparietal area or the area of maximal hallucinatory activation calculated from individual fMRI scans during auditory verbal hallucinations. |
| Sun 2018^33^ | Chinese mainland | rTMS (n=27), SHM (n=23) | DB | 4 | 2 | Low frenquency (1 Hz) rTMS was applied to the left temporoparietal cortex, while high frenquency (10 Hz) rTMS was applied to the left dorsolateral prefrontal cortex. |
| Tuppurainen 2020^34^ | Finland | rTMS (n=22), SHM (n=22) | DB | 3 | 1 | Individual α-peak-frequency-guided rTMS was applied to the left dorsolateral prefrontal cortex. |
| Wang 2016^35^ | Chinese mainland | rTMS (n=26), SHM (n=26) | DB | 6 | 2 | Low frenquency (1 Hz) rTMS was applied to the bilateral dorsolateral prefrontal cortex. |
| Weijer 2014^36^ | Netherlands | 1 Hz rTMS (n=10), 20Hz rTMS (n=8) | DB | 1 | 1 | Low frenquency (1 Hz) or high frenquency (20 Hz) rTMS was applied to the area based on individual auditory verbal hallucinations related activation patterns identified with functional magnetic resonance imaging. |
| Xie 2023^37^ | Chinese mainland | rTMS (n=32), SHM (n=26) | SB | 2.14 | 2 | Low frenquency (1 Hz) rTMS was applied to the left temporoparietal junction. |
| Xie 2024^38^ | Chinese mainland | rTMS (n=30), SHM (n=25) | DB | 2.14 | 2 | Low frenquency (1 Hz) rTMS was applied to the left temporoparietal junction. |
| Xu 2011^39^ | Chinese mainland | rTMS (n=18), SHM (n=17) | DB | 2 | 1 | Low frenquency (1 Hz) rTMS was applied to the left temporoparietal cortex. |
| Yang 2022^40^ | Chinese mainland | rTMS (n=69), SHM (n=69) | DB | 6 | 1 | Low frenquency (1 Hz) rTMS was applied to the left temporoparietal cortex. |
| Yue 2013^41^ | Chinese mainland | rTMS (n=40), SHM (n=40) | DB | 6 | 1 | Low frenquency (1 Hz) rTMS was applied to the left dorsolateral prefrontal cortex. |
| Zhang 2014^42^ | Chinese mainland | rTMS (n=40), SHM (n=40) | DB | 4 | 1 | Low frenquency (1 Hz) rTMS was applied to the left temporoparietal cortex. |
| Zhang 2016^43^ | Chinese mainland | rTMS (n=46), SHM (n=46) | DB | 2 | 1 | Low frenquency (1 Hz) rTMS was applied to the left dorsolateral prefrontal cortex. |
| Zhu 2013^44^ | Chinese mainland | rTMS (n=20), SHM (n=20) | DB | 1.4 | 1 | Low frenquency (1 Hz) rTMS was applied to the bilateral dorsolateral prefrontal cortex. |
| **iTBS studies** | | | | | | |
| Chauhan 2021^45^ | India | iTBS (n=19), SHM (n=17) | DB | 0.7 | 3 | iTBS coil was centered over midline cerebellum. |
| Wang 2021^46^ | Chinese mainland | iTBS (n=40), SHM (n=40) | DB | 2 | 1 | iTBS was applied to the bilateral temporoparietal cortex. |
| **cTBS studies** | | | | | | |
| Chen 2024^47^ | Chinese mainland | rTMS (n=50), cTBS (n=50) | DB | 4 | 3 | Low frenquency (1 Hz) rTMS or cTBS was applied to the bilateral superior temporal gyrus. |
| Hua 2024^48^ | Chinese mainland | cTBS (n=32), SHM (n=30) | DB | 2 | 1 | cTBS was applied to the left temporoparietal junction. |
| Kindler 2013a^49^ | Switzerland | rTMS (n=12), cTBS (n=12) | SB | 1.43 | 2 | Low frenquency (1 Hz) rTMS or cTBS was applied to the Area Sylvian parietotemporal. |
| Koops 2016^50^ | Netherlands | cTBS (n=37), SHM (n=34) | DB | 0.7 | 1 | cTBS was applied to the left temporoparietal cortex. |
| Plewnia 2014^51^ | Germany | cTBS (n=8), SHM (n=8) | SB | 3 | 1 | cTBS was applied to the temporoparietal cortices. |
| Plewnia 2025^52^ | Germany | cTBS (n=66), SHM (n=64) | DB | 3 | 1 | cTBS was applied to the temporoparietal cortices. |
| Potapov 2022^53^ | Russia | rTMS (n=30), cTBS (n=25), SHM (n=21) | SB | 3 | 1 | Low frenquency (1 Hz) rTMS or cTBS was applied to the left temporo-parietal cortex. |
| Tyagi 2022^54^ | India | cTBS (n=30), SHM (n=29) | DB | 2 | 2 | cTBS was applied to the temporoparietal cortices. |
| **dTMS studies** | | | | | | |
| Rosenberg 2012^55^ | Israel | dTMS (n=9), SHM (n=9) | DB | 1.43 | 1 | Low frenquency (1 Hz) dTMS was applied to the left temporoparietal cortex. |
| **tES studies** | | | | | | |
| **tDCS studies** | | | | | | |
| Bose 2018^56^ | India | tDCS (n=12), SHM (n=14) | DB | 0.7 | 2 | Anode was placed over the left dorsolateral prefrontal cortex and cathode over the left temporo-parietal junction. |
| Brunelin 2012^57^ | France | tDCS (n=15), SHM (n=15) | SB | 0.7 | 2 | Anode was placed over the left dorsolateral prefrontal cortex and cathode over the left temporo-parietal cortex. |
| Chang 2018^58^ | Taiwan region | tDCS (n=30), SHM (n=30) | DB | 0.7 | 2 | Anode was placed between the left prefrontal cortex and dorsolateral prefrontal cortex, while cathode over the left temporo-parietal junction. |
| Fitzgerald 2014a^59^ | Australia | tDCS (n=13), SHM (n=13) | DB | 3 | 2 | Anodal was placed over the prefrontal cortex and cathodal over the temporoparietal junction. |
| Fitzgerald 2014b^59^ | Australia | tDCS (n=11), SHM (n=11) | DB | 3 | 2 | Anodal was placed over the prefrontal cortex and cathodal over the temporoparietal junction. |
| Fröhlich 2015^60^ | USA | tDCS (n=13), SHM (n=13) | DB | 0.7 | 2 | Anodal was placed over the left-dorsolateral prefrontal cortex and cathodal over the left temporo-parietal junction. |
| Kantrowitz 2019^61^ | USA | tDCS (n=47), SHM (n=42) | DB | 0.7 | 2 | Stimulation was targeted at the fronto-temporal region. |
| Koops 2018^62^ | Netherlands | tDCS (n=28), SHM (n=26) | DB | 0.7 | 2 | Anode was placed over the left dorsolateral prefrontal cortex and cathode over the left temporo-parietal junction. |
| Lindenmayer 2019^63^ | USA | tDCS (n=15), SHM (n=13) | DB | 4 | 2 | Anode was placed over the left dorsolateral prefrontal cortex and cathode over the left temporo-parietal junction. |
| Marquardt 2022^64^ | Norway | tDCS (n=13), SHM (n=11) | DB | 0.7 | 2 | Anode was placed over the left dorsolateral prefrontal cortex and cathode over the left temporo-parietal cortex. |
| Mahato2025^65^ | India | tDCS (n=20), SHM (n=20) | DB | 0.7 | 1 | Anodal was placed over the left temporo-parietal junction according to the EEG system, electrode was surrounded by four return electrodes positioned at C3, T7, P3, and P7. |
| Parlikar 2025^66^ | India | tDCS (n=17), SHM (n=17) | DB | 0.7 | 1 | Cathode was placed at the temporo-parietal junction area. |
| Parlikar 2023^67^ | India | tDCS (n=17), SHM (n=17) | DB | 0.7 | 1 | tDCS stimulated the left temporo-parietal junction using subject-specific neuro-navigation. |
| **tRNS studies** | | | | | | |
| Donde 2023^68^ | France | tRNS (n=5), SHM (n=5) | DB | 0.7 | 1 | Anode was placed over the left prefrontal cortex and cathode over the left temporoparietal junction. |
| **tACS studies** | | | | | | |
| Wang 2025^69^ | Chinese mainland | tACS (n=16), SHM (n=16) | DB | 4 | 2 | Electrode was placed at the dorsolateral prefrontal cortex; another electrode was placed at the area of the left temporo-parietal junction area. |
| Mellin 2018^70^ | USA | tACS (n=8), tDCS (n=7), SHM (n=7) | DB | 0.7 | 2 | tACS stimulates between the frontal site and Cz and between the temporo-parietal site and Cz, respectively. The stimulation paradigm for tDCS was +2 mA at the frontal site and -2 mA at the temporo-parietal site. |
| **ECT and MST studies** | | | | | | |
| Argawal 1985^71^ | India | ECT (n=15), SHM (n=15) | SB | 2.3 | 1 | ECT electrodes were placed bilaterally on the temporal regions. |
| Goswami 2003^72^ | India | ECT (n=15), SHM (n=10) | DB | 4 | 2 | ECT electrodes placed bilaterally with unipolar frontal EEG lead. |
| Li 2019^73^ | Chinese mainland | ECT (n=6), ECT (n=6) | DB | 4 | 1 | Low-charge or standard ECT electrodes were placed bilaterally on the temporal regions. |
| Melzer-Ribeiro 2017^74^ | Brazil | ECT (n=13), SHM (n=10) | SB | 4 | 2 | ECT electrodes were placed bilaterally on the temporal regions. |
| Melzer-Ribeiro 2023^75^ | Brazil | ECT (n=21), SHM (n=19) | DB | 10 | 3 | ECT electrodes were placed bilaterally on the temporal regions. |
| Petrides 2015^76^ | USA | ECT (n=20), SHM (n=19) | SB | 8 | 2 | ECT electrodes were placed bilaterally. |
| Xu 2020^77^ | Chinese mainland | ECT (n=11), MST (n=8) | SB | 4 | 3 | ECT electrodes were placed bilaterally on the temporal regions, while the center of the MST coil is aligned with the F3/F4 position. |

*Categories of the Treatment-Resistant Levels: 1 = Minimum cut-off, 2 = Moderate cut-off, 3 = Optimum cut-off; see Appendix ‘Criteria for Treatment-Resistant Categorization’ above for a detailed description of the categories.

cTBS: continuous theta-burst stimulation; DB: Double Blind; dTMS: Deep transcranial magnetic stimulation; ECT: Electroconvulsive therapy; iTBS: intermittent theta burst stimulation; MST: Magnetic seizure therapy; rTMS: Repetitive transcranial magnetic stimulation; SB: Single Blind; SHM: Sham therapy; tACS: Transcranial alternating current stimulation; TAU: Treatment as Usual; tDCS: Transcranial direct current stimulation; tRNS: Transcranial random noise stimulation.

**References**

1. 安翠霞, 董玲, 于鲁璐, 刘艳菊, 任会鹏, 王学义. 不同部位低频重复经颅磁刺激治疗顽固性幻听疗效和安全性的双盲对照研究. *中华精神科杂志* 2011; **44**(4): 256-.
2. Bais L, Vercammen A, Stewart R, et al. Short and long term effects of left and bilateral repetitive transcranial magnetic stimulation in schizophrenia patients with auditory verbal hallucinations: a randomized controlled trial. PloS one 2014; 9(10): e108828.
3. Blumberger DM, Christensen BK, Zipursky RB, et al. MRI-targeted repetitive transcranial magnetic stimulation of Heschl’s gyrus for refractory auditory hallucinations. Brain stimulation 2012; 5(4): 577-85.
4. Brunelin J, Poulet E, Bediou B, et al. Low frequency repetitive transcranial magnetic stimulation improves source monitoring deficit in hallucinating patients with schizophrenia. Schizophrenia research 2006; 81(1): 41-5.
5. 成军, 李红, 石玉中. 氯氮平联合重复经颅磁刺激治疗难治性精神分裂症的临床疗效. 中国康复医学杂志 2013; 28(8): 754-7.
6. Dollfus S, Jaafari N, Guillin O, et al. High-frequency neuronavigated rTMS in auditory verbal hallucinations: a pilot double-blind controlled study in patients with schizophrenia. Schizophrenia Bulletin 2018; 44(3): 505-14.
7. Fitzgerald PB, Benitez J, Daskalakis JZ, et al. A double-blind sham-controlled trial of repetitive transcranial magnetic stimulation in the treatment of refractory auditory hallucinations. Journal of Clinical Psychopharmacology 2005; 25(4): 358-62.
8. 高志勤, 余海鹰, 崔雪莲, 金梅, 邱旭萍. 低频重复经颅磁刺激治疗精神分裂症慢性幻听的疗效及随访研究. 精神医学杂志 2009; 22(4): 257-8.
9. Gornerova N, Brunovsky M, Klirova M, et al. The effect of low-frequency rTMS on auditory hallucinations, EEG source localization and functional connectivity in schizophrenia. Neuroscience letters 2023; 794: 136977.
10. Hajak G, Marienhagen J, Langguth B, Werner S, Binder H, Eichhammer P. High-frequency repetitive transcranial magnetic stimulation in schizophrenia: a combined treatment and neuroimaging study. Psychol Med. 2004 Oct;34(7):1157-63.
11. Hoffman RE, Gueorguieva R, Hawkins KA, et al. Temporoparietal transcranial magnetic stimulation for auditory hallucinations: safety, efficacy and moderators in a fifty patient sample. Biological psychiatry 2005; 58(2): 97-104.
12. Holi MM, Eronen M, Toivonen K, Toivonen P, Marttunen M, Naukkarinen H. Left prefrontal repetitive transcranial magnetic stimulation in schizophrenia. Schizophrenia bulletin 2004; 30(2): 429-34.
13. 胡浩浩, 陈红英, 王璎. 重复经颅磁刺激治疗精神分裂症顽固性幻听的临床效果. 中国民康医学 2019; 31(1): 88-90.
14. Jandl M, Steyer J, Weber M, et al. Treating auditory hallucinations by transcranial magnetic stimulation: a randomized controlled cross-over trial. Neuropsychobiology 2006; 53(2): 63-9.
15. de Jesus DR, Gil A, Barbosa L, et al. A pilot double-blind sham-controlled trial of repetitive transcranial magnetic stimulation for patients with refractory schizophrenia treated with clozapine. Psychiatry research 2011; 188(2): 203-7.
16. 姜洪亮. 低频重复经颅磁刺激治疗精神分裂症顽固性幻听的随机双盲对照研究. 心理医生 2017; 23(12): 60-1.
17. Kim E-J, Yeo S, Hwang I, et al. Bilateral repetitive transcranial magnetic stimulation for auditory hallucinations in patients with schizophrenia: a randomized controlled, cross-over study. Clinical Psychopharmacology and Neuroscience 2014; 12(3): 222.
18. Kimura H, Kanahara N, Takase M, Yoshida T, Watanabe H, Iyo M. A randomized, sham-controlled study of high frequency rTMS for auditory hallucination in schizophrenia. Psychiatry research 2016; 241: 190-4.
19. Klirova M, Horacek J, Novak T, et al. Individualized rTMS neuronavigated according to regional brain metabolism (18 FGD PET) has better treatment effects on auditory hallucinations than standard positioning of rTMS: a double-blind, sham-controlled study. European archives of psychiatry and clinical neuroscience 2013; 263: 475-84.
20. Klein E, Kolsky Y, Puyerovsky M, Koren D, Chistyakov A, Feinsod M. Right prefrontal slow repetitive transcranial magnetic stimulation in schizophrenia: a double-blind sham-controlled pilot study. Biol Psychiatry. 1999 Nov 15;46(10):1451-4.
21. Laurin A, Le Strat Y, Marinescu M, Tébéka S, Dubertret C. P. 551 Effects of the transcranial magnetic stimulation on the sense of agency and body ownership impairments in schizophrenia with first-rank symptoms. European Neuropsychopharmacology 2020; 40: S312-S3.
22. Lee S-H, Kim W, Chung Y-C, et al. A double blind study showing that two weeks of daily repetitive TMS over the left or right temporoparietal cortex reduces symptoms in patients with schizophrenia who are having treatment-refractory auditory hallucinations. Neuroscience letters 2005; 376(3): 177-81.
23. 李艳红, 吴强, 王敬巍, 石玉中. 低频重复经颅磁刺激治疗精神分裂症顽固性幻听疗效观察. 临床心身疾病杂志 2015; 21(4): 131-2.
24. 路亚洲, 姜玮, 任艳萍, 刘志宏, 周丹娜, 马辛. 20Hz 重复经颅磁刺激治疗改善难治性精神分裂症注意和执行功能的损害. 中国健康心理学杂志 2014; 22(10): 1448-50.
25. Mao J, Fan K, Zhang Y, et al. 10 Hz repetitive transcranial magnetic stimulation (rTMS) may improve cognitive function: An exploratory study of schizophrenia patients with auditory hallucinations. Heliyon. 2023;9(9):e19912.
26. McIntosh AM, Semple D, Tasker K, et al. Transcranial magnetic stimulation for auditory hallucinations in schizophrenia. Psychiatry research 2004; 127(1-2): 9-17.
27. Paillère‐Martinot ML, Galinowski A, Plaze M, et al. Active and placebo transcranial magnetic stimulation effects on external and internal auditory hallucinations of schizophrenia. Acta Psychiatrica Scandinavica 2017; 135(3): 228-38.
28. 蒲绮霞, 张春萍, 黄雄, et al. 低频重复经颅磁刺激治疗顽固性幻听的疗效及对脑源性神经营养因子的影响. 广东医学 2013; 34(16): 2500-2.
29. 权文香, 乔宏, 赵志宇, et al. 低频重复经颅磁刺激治疗精神分裂症患者的顽固性幻听. 中国心理卫生杂志 2012; 26(3): 204-8.
30. 任艳萍, 周东丰, 蔡焯基, 黄青, 卢苓, 陈琦. 低频重复经颅磁刺激治疗精神分裂症顽固性幻听的随机双盲对照研究. 中国心理卫生杂志 2010; 24(3): 195-7.
31. Rosa MO, Gattaz WF, Rosa MA, et al. Effects of repetitive transcranial magnetic stimulation on auditory hallucinations refractory to clozapine. Journal of Clinical Psychiatry 2007; 68(10): 1528-32.
32. Slotema CW, Blom JD, de Weijer AD, et al. Can low-frequency repetitive transcranial magnetic stimulation really relieve medication-resistant auditory verbal hallucinations? Negative results from a large randomized controlled trial. Biological psychiatry 2011; 69(5): 450-6.
33. 孙丛丛, 周丹娜, 宁芳平, et al. 重复经颅磁刺激治疗难治性精神分裂症的增效作用研究. 首都医科大学学报 2018; 39(5): 641-5.
34. Tuppurainen H, Määttä S, Könönen M, et al. Navigated and individual α-peak-frequency–guided transcranial magnetic stimulation in male patients with treatment-refractory schizophrenia. Journal of Psychiatry and Neuroscience 2024; 49(2): E87-E95.
35. 王雪, 罗炯, 李晓虹, 任艳萍. 低频重复经颅磁刺激对难治性精神分裂症的增效作用. 四川精神卫生 2016; (1): 41-5.
36. de Weijer AD, Sommer IE, Meijering AL, et al. High frequency rTMS; a more effective treatment for auditory verbal hallucinations? Psychiatry Research: Neuroimaging 2014; 224(3): 204-10.
37. Xie Y, Guan M, He Y, et al. The Static and dynamic functional connectivity characteristics of the left temporoparietal junction region in schizophrenia patients with auditory verbal hallucinations during low-frequency rTMS treatment. Front Psychiatry 2023; 14: 1071769.
38. Xie Y, Li C, Guan M, et al. Low-frequency rTMS induces modifications in cortical structural connectivity - functional connectivity coupling in schizophrenia patients with auditory verbal hallucinations. Hum Brain Mapp 2024; 45(3): e26614.
39. 徐亚秋, 谢世平, 杜经纶. 低频重复经颅磁刺激治疗精神分裂症顽固性幻听的临床研究. 临床精神医学杂志 2011; 21(3): 177-80.
40. 杨菊, 罗兴刚, 李清均. 利培酮联合低频重复经颅磁刺激治疗精神分裂症顽固性幻听的效果及对事件相关电位的影响. 实用医院临床杂志 2022; 19(4): 169-73.
41. 岳莉莉, 柏光泽. 低频重复经颅磁刺激对精神分裂症患者顽固性幻听的疗效分析. 临床军医杂志 2013; (3): 275-7.
42. 张翠红, 张岿, 张洁, 于志红, 杨国辉. 低频重复经颅磁刺激对精神分裂症顽固性幻听的随机双盲对照研究. 中国民康医学 2014; 26(16): 20-1.
43. 张少霞, 李一凡, 郑丽华, 谢益潮. 抗精神病药物联合低频重复经颅磁刺激治疗对精神分裂症顽固性幻听的临床对照研究. 中外医疗 2016; 35(30): 4-6.
44. 朱琳, 刘少华. 低频重复经颅磁刺激治疗精神分裂症难治性幻听的疗效. 赣南医学院学报 2013; 33(4): 534-5.
45. Chauhan P, Garg S, Tikka SK, Khattri S. Efficacy of Intensive Cerebellar Intermittent Theta Burst Stimulation (iCiTBS) in Treatment-Resistant Schizophrenia: a Randomized Placebo-Controlled Study. Cerebellum. 2021;20(1):116-123.
46. 王璐. 难治性精神分裂症的 rTMS 精准干预及其神经机制的研究: 安徽医科大学; 2021.
47. 陈基娜, 戴伯坚, 于明荣, 刘超群, 朱晏卉. 不同rTMS模式对精神分裂症幻听患者的应用效果研究. 浙江临床医学 2024; 26(2): 169-71.
48. Hua Q, Wang L, He K, et al. Repetitive Transcranial Magnetic Stimulation for Auditory Verbal Hallucinations in Schizophrenia: A Randomized Clinical Trial. JAMA Netw Open 2024; 7(11): e2444215.
49. Kindler J, Homan P, Flury R, Strik W, Dierks T, Hubl D. Theta burst transcranial magnetic stimulation for the treatment of auditory verbal hallucinations: results of a randomized controlled study. Psychiatry research 2013; 209(1): 114-7.
50. Koops S, Dellen Ev, Schutte MJ, Nieuwdorp W, Neggers SF, Sommer IE. Theta burst transcranial magnetic stimulation for auditory verbal hallucinations: negative findings from a double-blind-randomized trial. Schizophrenia bulletin 2016; 42(1): 250-7.
51. Plewnia C, Zwissler B, Wasserka B, Fallgatter AJ, Klingberg S. Treatment of auditory hallucinations with bilateral theta burst stimulation: a randomized controlled pilot trial. Brain Stimulation: Basic, Translational, and Clinical Research in Neuromodulation 2014; 7(2): 340-1.
52. Plewnia C, Brendel B, Schwippel T, et al. Theta burst stimulation of temporo-parietal cortex regions for the treatment of persistent auditory hallucinations: a multicentre, randomised, sham-controlled, triple-blind phase 3 trial in Germany. Lancet Psychiatry. 2025;12(9):638-649.
53. Potapov I, Maslenikov N, Tsukarzi E, Mosolov S. Randomized comparative study of 1-Hz transcranial magnetic stimulation (TMS), continuous theta-burst stimulation (cTBS) and sham-TMS for treatment-refractory auditory hallucinations (AH) in schizophrenia. European Psychiatry 2022; 65(S1): S737-S.
54. Tyagi P, Dhyani M, Khattri S, Tejan V, Tikka SK, Garg S. “Efficacy of intensive bilateral Temporo-Parietal Continuous theta-burst Stimulation for Auditory VErbal hallucinations (TPC-SAVE) in schizophrenia: A randomized sham-controlled trial”☆. Asian Journal of Psychiatry 2022; 74: 103176.
55. Rosenberg O, Gersner R, Klein LD, Kotler M, Zangen A, Dannon P. Deep transcranial magnetic stimulation add-on for the treatment of auditory hallucinations: a double-blind study. Annals of General Psychiatry 2012; 11: 1-6.
56. Bose A, Shivakumar V, Agarwal SM, et al. Efficacy of fronto-temporal transcranial direct current stimulation for refractory auditory verbal hallucinations in schizophrenia: a randomized, double-blind, sham-controlled study. Schizophrenia research 2018; 195: 475-80.
57. Brunelin J, Mondino M, Gassab L, et al. Examining transcranial direct-current stimulation (tDCS) as a treatment for hallucinations in schizophrenia. American Journal of Psychiatry 2012; 169(7): 719-24.
58. Chang C-C, Tzeng N-S, Chao C-Y, Yeh C-B, Chang H-A. The effects of add-on fronto-temporal transcranial direct current stimulation (tDCS) on auditory verbal hallucinations, other psychopathological symptoms, and insight in schizophrenia: a randomized, double-blind, sham-controlled trial. International Journal of Neuropsychopharmacology 2018; 21(11): 979-87.
59. Fitzgerald PB, McQueen S, Daskalakis ZJ, Hoy KE. A negative pilot study of daily bimodal transcranial direct current stimulation in schizophrenia. Brain stimulation 2014; 7(6): 813-6.
60. Fröhlich F, Burrello TN, Mellin JM, et al. Exploratory study of once-daily transcranial direct current stimulation (tDCS) as a treatment for auditory hallucinations in schizophrenia. European Psychiatry 2016; 33(1): 54-60.
61. Kantrowitz JT, Sehatpour P, Avissar M, et al. Significant improvement in treatment resistant auditory verbal hallucinations after 5 days of double-blind, randomized, sham controlled, fronto-temporal, transcranial direct current stimulation (tDCS): a replication/extension study. Brain stimulation 2019; 12(4): 981-91.
62. Koops S, Blom JD, Bouachmir O, Slot MI, Neggers B, Sommer IE. Treating auditory hallucinations with transcranial direct current stimulation in a double-blind, randomized trial. Schizophrenia research 2018; 201: 329-36.
63. Lindenmayer J, Kulsa MKC, Sultana T, et al. Transcranial direct-current stimulation in ultra-treatment-resistant schizophrenia. Brain stimulation 2019; 12(1): 54-61.
64. Marquardt L, Craven AR, Hugdahl K, et al. Pilot-RCT finds no evidence for modulation of neuronal networks of auditory hallucinations by transcranial direct current stimulation. Brain Sciences 2022; 12(10): 1382.
65. Mahato AN, Munda SK, Pratap A, Mudaliyar S, Patel N. Adjunctive High-Definition Transcranial Direct Current Stimulation in Treatment of Resistant Auditory Verbal Hallucinations in Schizophrenia: A Randomized Sham-Controlled Trial. J ECT.
66. Parlikar R, Chhabra H, Selvaraj S, et al. Effects of high-definition transcranial direct current stimulation (HD-tDCS) on resting brain functional connectivity in schizophrenia patients with persistent auditory verbal hallucinations. Asian J Psychiatr. 2025;110:104571.
67. Parlikar R, Chhabra H, Selvaraj S, et al. Neurobiological and clinical effects of High-Definition tDCS on persistent auditory hallucinations in schizophrenia: A randomized controlled trial. medRxiv 2023: 2023.05. 10.23289796.
68. Dondé C, Fivel L, Haesebaert F, Poulet E, Mondino M, Brunelin J. Mechanistic account of the left auditory cortex for tone-matching in schizophrenia: A pilot transcranial random noise stimulation (tRNS) sham-controlled study. Asian Journal of Psychiatry 2024; 92: 103879.
69. Wang X, Zhang X, Chang Y, Liao J, Liu S, Ming D. Double-blind, randomized, placebo-controlled pilot clinical trial with gamma-band transcranial alternating current stimulation for the treatment of schizophrenia refractory auditory hallucinations. Transl Psychiatry. 2025;15(1):36.
70. Mellin JM, Alagapan S, Lustenberger C, et al. Randomized trial of transcranial alternating current stimulation for treatment of auditory hallucinations in schizophrenia. European Psychiatry 2018; 51: 25-33.
71. Agarwal A, Winny G. Role of ECT phenothiazine combination in schizophrenia. Indian Journal of psychiatry 1985; 27(3): 233-6.
72. Goswami U, Kumar U, Singh B. Efficacy of electroconvulsive therapy in treatment resistant schizophreinia: a double-blind study. Indian journal of psychiatry 2003; 45(1): 26-9.
73. Li M-z, Chen L-c, Rong H, et al. Low-charge electrotherapy for patients with schizophrenia: a double-blind, randomised controlled pilot clinical trial. Psychiatry Research 2019; 272: 676-81.
74. Melzer-Ribeiro DL, Rigonatti SP, Kayo M, et al. Efficacy of electroconvulsive therapy augmentation for partial response to clozapine: a pilot randomized ECT–sham controlled trial. Archives of Clinical Psychiatry (São Paulo) 2017; 44: 45-50.
75. Melzer-Ribeiro D, Napolitano I, Leite S, et al. Randomized, double-blind, sham-controlled trial to evaluate the efficacy and tolerability of electroconvulsive therapy in patients with clozapine-resistant schizophrenia. Schizophrenia research 2024; 268: 252-60.
76. Petrides G, Malur C, Braga RJ, et al. Electroconvulsive therapy augmentation in clozapine-resistant schizophrenia: a prospective, randomized study. American Journal of Psychiatry 2015; 172(1): 52-8.
77. 许珮玮. 超难治性精神分裂症的MST与MECT平行对照探索性研究: 上海交通大学; 2020.

# **6. Risk of Bias**

Risk of bias was assessed by two reviewers using the RoB-2 tool. This tool evaluates potential bias across five domains: the randomization process, deviations from intended interventions, missing outcome data, measurement of the outcome, and selection of the reported result.

During the assessment, two studies (Melzer-Ribeiro 2017 and Wang 2021) were considered to be at a high risk of bias in the randomization process - Melzer-Ribeiro 2017 due to baseline differences, and Wang 2021 because of a suboptimal randomisation procedure. However, this does not necessarily indicate intentional manipulation, and there is no clear evidence suggesting deliberate bias in the randomization process. Therefore, we decided to include both studies in the analysis.

Due to the small sample sizes, limited head-to-head comparisons, and the star-shaped network of NIBS studies, we focused on the risk of bias for the primary outcome (i.e., overall symptoms) rather than on dropouts.

**
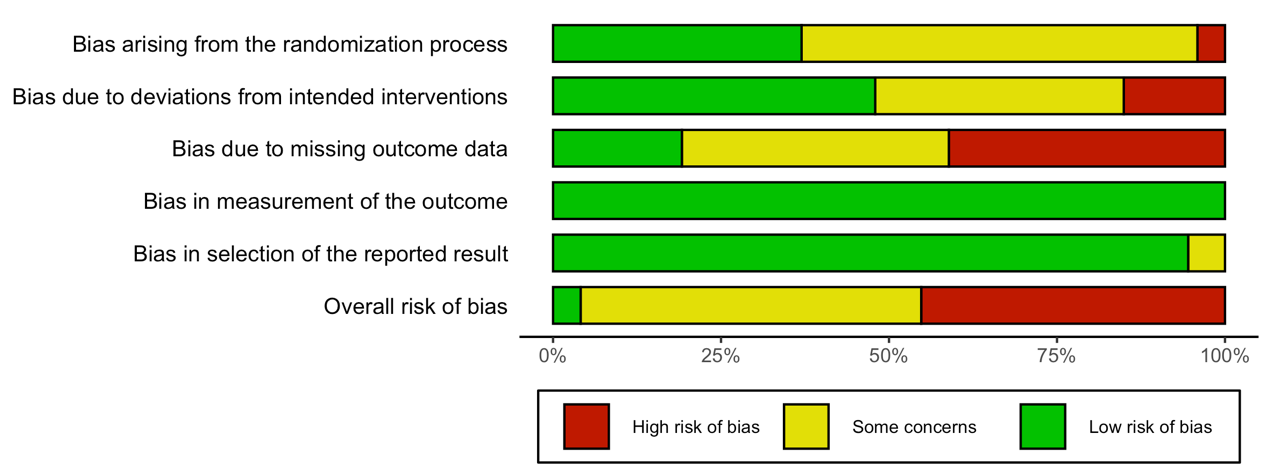
**

| **Study** | **Randomisation** | **Deviations from intended interventions** | **Missing outcome data** | **Measurement of the outcome** | **Selection of the reported results** | **Overall judgement** |
| --- | --- | --- | --- | --- | --- | --- |
| An 2011 | Some concerns | Some concerns | High | Low | Low | High |
| Argawal 1985 | Some concerns | High | High | Low | Low | High |
| Bais 2014 | Low | Some concerns | Some concerns | Low | Low | Some concerns |
| Blumberger 2012 | Some concerns | Low | Some concerns | Low | Low | Some concerns |
| Bose 2018 | Low | Some concerns | Some concerns | Low | Low | Some concerns |
| Brunelin 2006 | Some concerns | Low | High | Low | Low | High |
| Brunelin 2012 | Some concerns | Low | Low | Low | Low | Some concerns |
| Chang 2018 | Some concerns | Low | Low | Low | Low | Some concerns |
| Chauhan 2021 | Low | Low | Some concerns | Low | Low | Some concerns |
| Cheng 2013 | Some concerns | Some concerns | Some concerns | Low | Low | Some concerns |
| Dollfus 2018 | Low | High | High | Low | Low | High |
| Donde 2023 | Low | Low | Low | Low | Low | Low |
| Fitzgerald 2005 | Low | Low | Some concerns | Low | Low | Some concerns |
| Fröhlich 2015 | Some concerns | Low | High | Low | Low | High |
| Gao 2009 | Some concerns | Low | Low | Low | Some concerns | Some concerns |
| Gornerova 2023 | Some concerns | Low | High | Low | Low | High |
| Goswami 2003 | Low | High | High | Low | Low | High |
| Hajak 2004 | Some concerns | Some concerns | High | Low | Low | High |
| Holi 2004 | Low | Low | Some concerns | Low | Low | Some concerns |
| Hu 2019 | Some concerns | Some concerns | High | Low | Low | High |
| Hua 2024 | Some concerns | Low | High | Low | Low | High |
| Jandl 2006 | Low | Low | High | Low | Low | High |
| Jesus 2011 | Low | Some concerns | High | Low | Low | High |
| Jiang 2017 | Some concerns | Low | Low | Low | Low | Some concerns |
| Kantrowitz 2019 | Low | Low | High | Low | Low | High |
| Kim 2014 | Some concerns | High | High | Low | Low | High |
| Kimura 2016 | Some concerns | Low | High | Low | Low | High |
| Klein 1999 | High | Some concerns | Some concerns | Low | Low | High |
| Klirova 2013 | Some concerns | Low | Some concerns | Low | Low | Some concerns |
| Koops 2016 | Some concerns | Some concerns | Some concerns | Low | Low | Some concerns |
| Koops 2018 | Low | High | High | Low | Low | High |
| Laurin 2020 | Low | Low | Low | Low | Low | Low |
| Lee 2005 | Some concerns | Low | High | Low | Some concerns | High |
| Li 2015 | Some concerns | Low | Low | Low | Low | Some concerns |
| Lindenmayer 2019 | Some concerns | Low | High | Low | Low | High |
| Mahato 2025 | Low | Some concerns | Some concerns | Low | Low | Some concerns |
| Mao 2023 | Some concerns | Some concerns | Some concerns | Low | Low | Some concerns |
| Marquardt 2022 | Low | Some concerns | Some concerns | Low | Low | Some concerns |
| McIntosh 2004 | Low | Low | Low | Low | Low | Low |
| Mellin 2018 | Low | Some concerns | Some concerns | Low | Low | Some concerns |
| Melzer 2023 | Low | Some concerns | Some concerns | Low | Low | Some concerns |
| Melzer-Ribeiro 2017 | High | Some concerns | High | Low | Low | High |
| Paillere-Martinot 2017 | Low | Low | Some concerns | Low | Low | Some concerns |
| Parlikar 2023 | Low | Low | High | Low | Low | High |
| Parlikar 2025 | Low | Low | High | Low | Low | High |
| Petrides 2015 | Some concerns | Low | Some concerns | Low | Low | Some concerns |
| Plewnia 2014 | Some concerns | Low | Some concerns | Low | Low | Some concerns |
| Plewnia 2025 | Low | Low | Some concerns | Low | Low | Some concerns |
| Potapov 2022 | Some concerns | Some concerns | Low | Low | Low | Some concerns |
| Pu 2013 | Some concerns | Low | Low | Low | Low | Some concerns |
| Quan 2012 | Some concerns | Some concerns | Some concerns | Low | Low | Some concerns |
| Ren 2010 | Some concerns | Low | Low | Low | Low | Some concerns |
| Rosa 2007 | Some concerns | Some concerns | High | Low | Some concerns | High |
| Rosenberg 2012 | Low | High | Some concerns | Low | Low | High |
| Slotema 2011 | Low | Low | High | Low | Low | High |
| Sun 2018 | Some concerns | Some concerns | Some concerns | Low | Low | Some concerns |
| Tuppurainen 2020 | Some concerns | Low | Some concerns | Low | Low | Some concerns |
| Tyagi 2022 | Low | Low | Some concerns | Low | Low | Some concerns |
| Wang 2016 | Some concerns | Some concerns | Some concerns | Low | Low | Some concerns |
| Wang 2021 | High | Some concerns | Some concerns | Low | Low | High |
| Wang 2025 | Some concerns | High | High | Low | Low | High |
| Xie 2023 | Some concerns | Some concerns | Some concerns | Low | Low | Some concerns |
| Xie 2024 | Some concerns | High | High | Low | Low | High |
| Xu 2011 | Some concerns | Low | Low | Low | Low | Some concerns |
| Xu 2020 | Some concerns | Some concerns | Some concerns | Low | Low | Some concerns |
| Yang 2022 | Some concerns | High | High | Low | Low | High |
| Yue 2013 | Some concerns | Low | Low | Low | Low | Some concerns |
| Zhang 2014 | Some concerns | Some concerns | Low | Low | Low | Some concerns |
| Zhang 2016 | Some concerns | High | High | Low | Low | High |
| Zhu 2013 | Some concerns | High | High | Low | Low | High |

# **7. Transitivity assessments**

We only included trials involving individuals with treatment-resistant schizophrenia who received NIBS as an add-on to antipsychotics. We excluded trials focusing on specific populations, such as those with comorbidities, cognitive impairment, or predominantly negative symptoms. Therefore, we assumed that individuals in the included trials had an equal likelihood of being randomized to any intervention.

Additionally, we inspected the transitivity assumption by assessing the distribution of potential effect modifiers across treatment comparisons. These included baseline symptom severity, illness duration, publication year, sample size, treatment duration and frequency, average participant age, proportion of female participants, definition of treatment resistance, schizophrenia diagnosis, use of PANSS rating scales, blinding status, and sponsorship. We did not consider the dose and type of antipsychotics, as these are rarely reported in the included trials.

In the following boxplots, the black line in the box indicates the median value, the margins of the box indicate the 25% and 75% quantiles, the whiskers extend to the largest value no further than 1.5 times the interquartile range from the hinges and dots outside the whiskers indicate outliers.

##
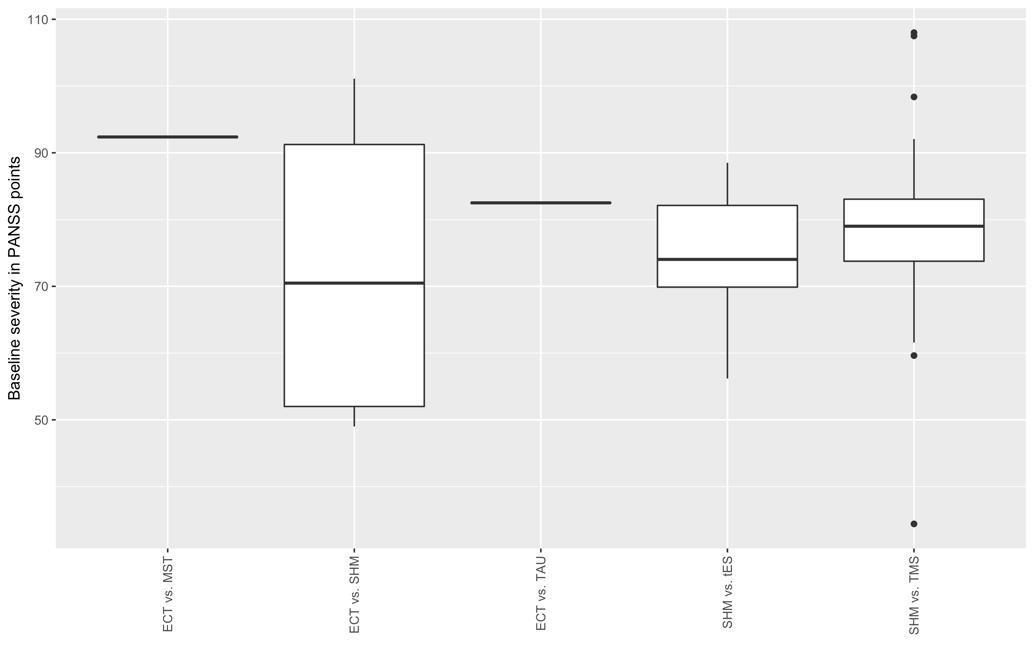
**7.1 Baseline severity of symptoms**

For baseline severity, we converted BPRS-18 baseline scores of studies to PANSS equivalents. The boxplot shows variability in baseline severity (PANSS scores) across treatment comparisons. ECT vs. SHM studies show a wide range, while ECT vs. MST and ECT vs. TAU each stem from a single study. Overall, the comparisons exhibit moderate and overlapping PANSS distributions, with most studies including patients with PANSS scores around 70 to 80, corresponding to approximately “moderately ill”.^1^ An apparent outlier is the ECT vs. MST comparison, but this is based on only one study.

## **7.2 Duration of illness**

**
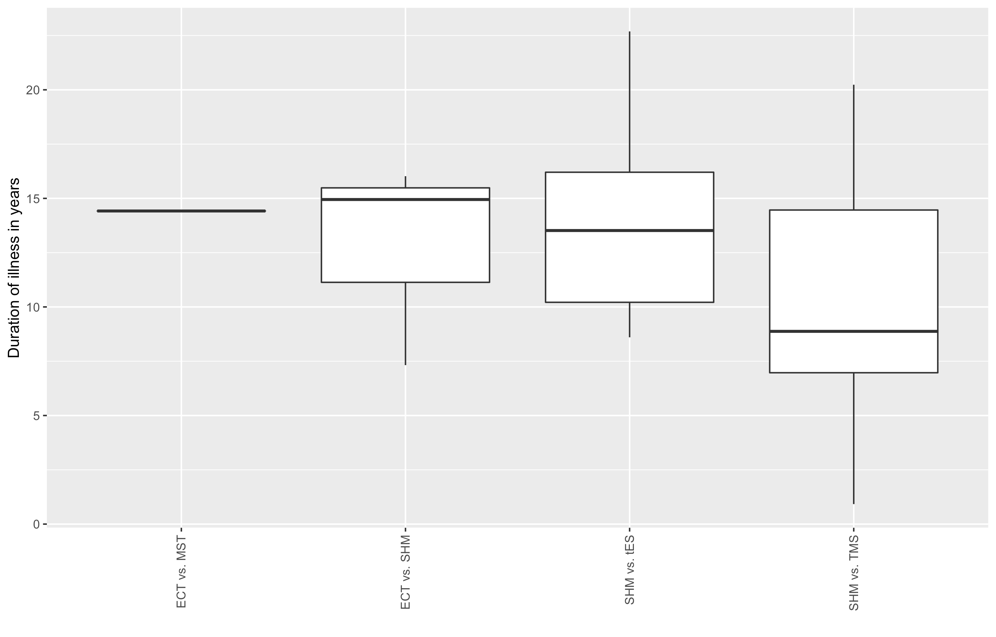
**

The boxplot shows the distribution of illness duration across treatment comparisons. Most comparisons cluster around a median of 10–15 years which we interpreted as no clear evidence of violations of the transitivity assumption.

## **
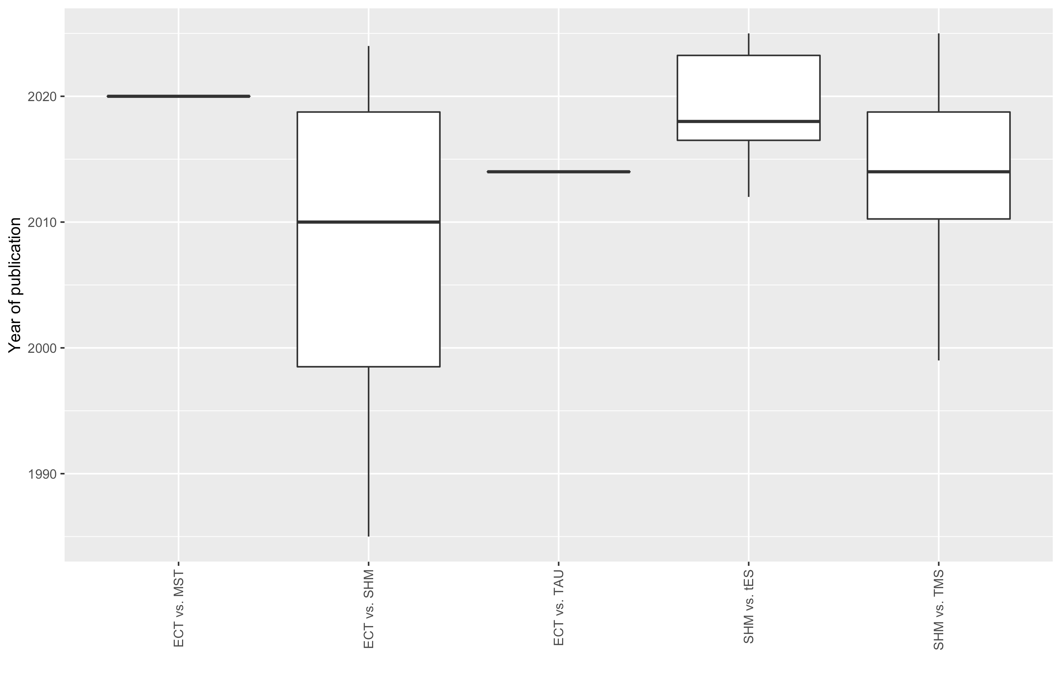
7.3 Publication year**

The boxplot displays the distribution of publication years across treatment comparisons. Most studies were published between 2005 and 2022, with ECT vs. SHM showing the widest range. ECT vs. MST came from a single, recent study which still lay in a similar range as the other ones. Overall, we found no clear evidence of violations of the transitivity assumption.

## **
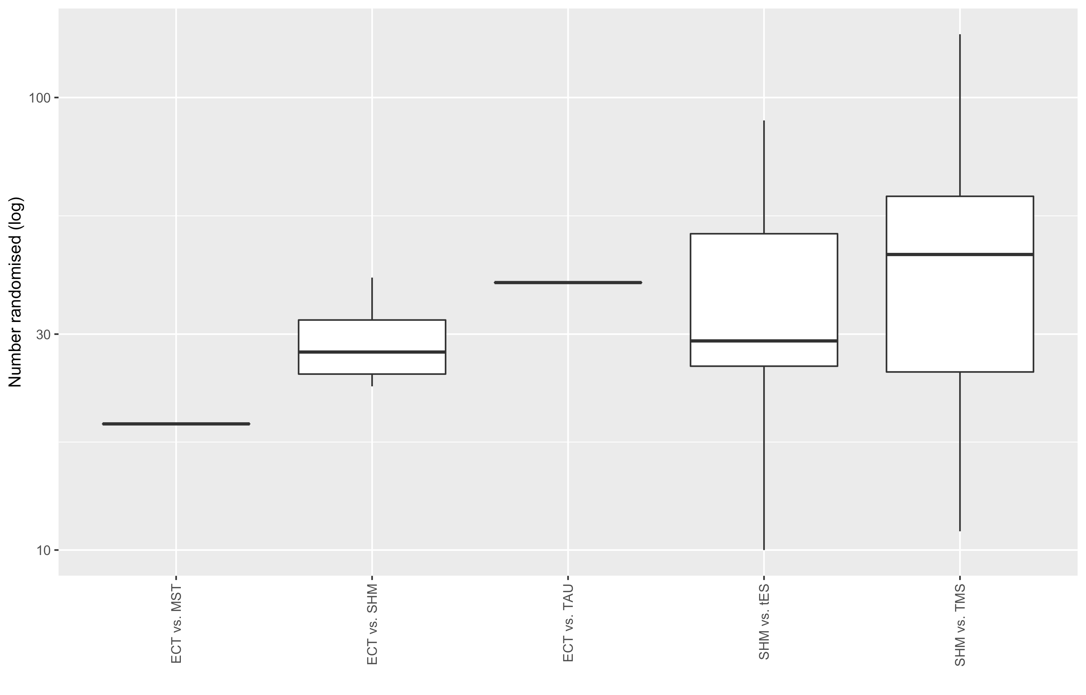
7.4 Sample size**

The boxplot shows the distribution of sample sizes (log scale) across treatment comparisons. Most studies randomized between 20 and 100 participants, with SHM vs. rTMS showing the largest variation. The sample size of ECT vs MST was 19, based on a single study. We interpreted these findings as no clear evidence of violations of the transitivity assumption.

## **
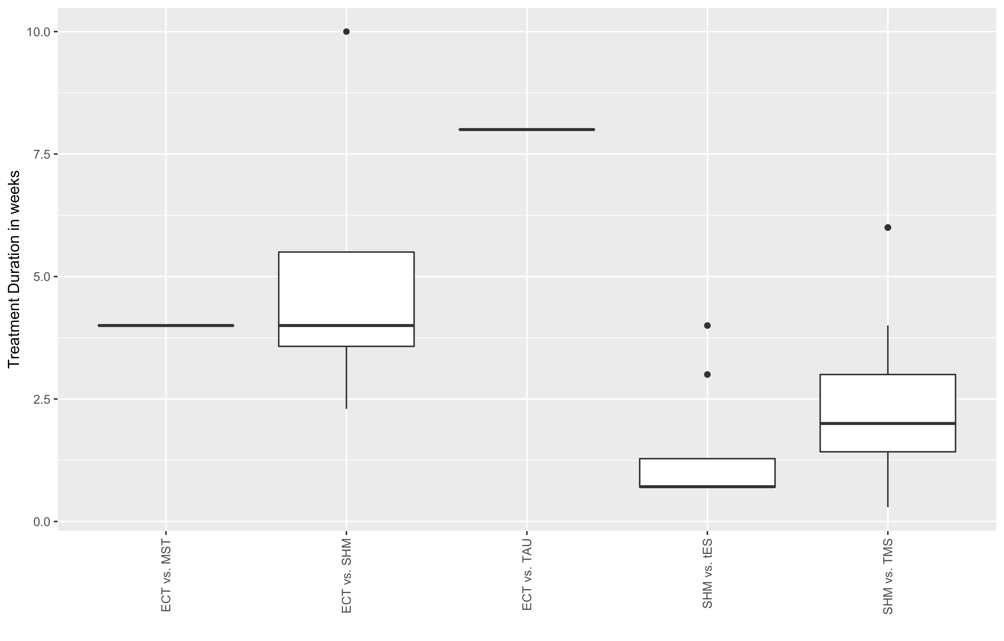
7.5 Treatment duration**

The boxplot illustrates variability in treatment duration across comparisons. ECT trials generally had longer durations because ECT cannot be administered more frequently than 2–3 times per week. SHM vs. tES and SHM vs. rTMS had shorter and more variable durations. Despite this variation, a meta-regression on treatment duration did not reveal any significant moderating effect. Our interpretation is no clear evidence of violations of the transitivity assumption.

## **
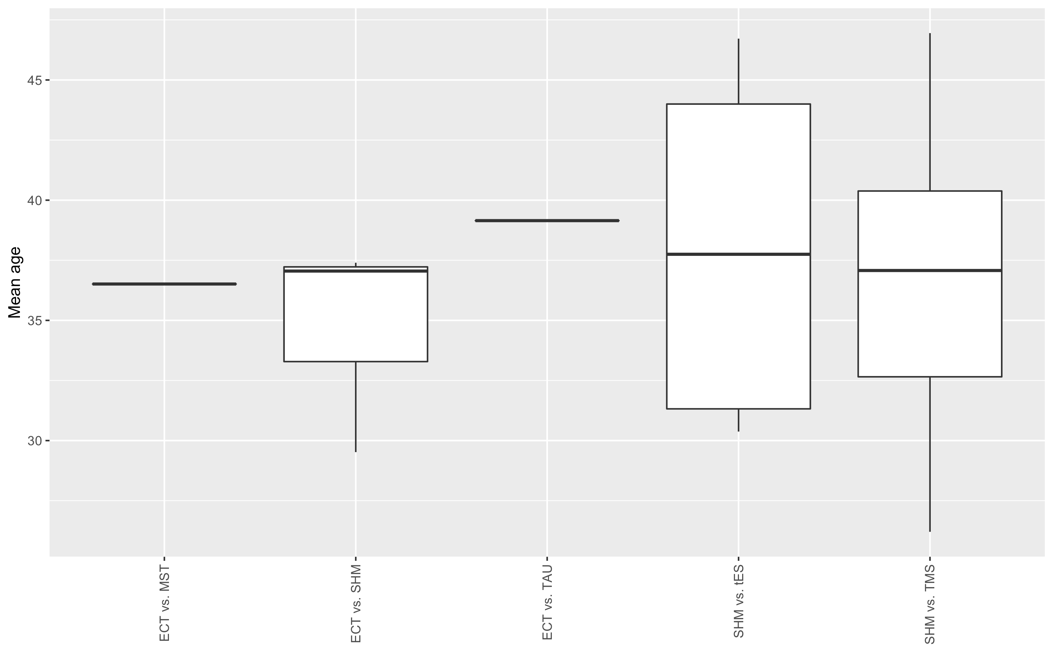
7.6 Mean age of participants**

The boxplot shows the distribution of mean age across treatment comparisons. Most comparisons cluster around a mean age of 35–40 years. We did not find any clear evidence of violations of the transitivity assumption.

## **7.7 Women proportion of participants**

**
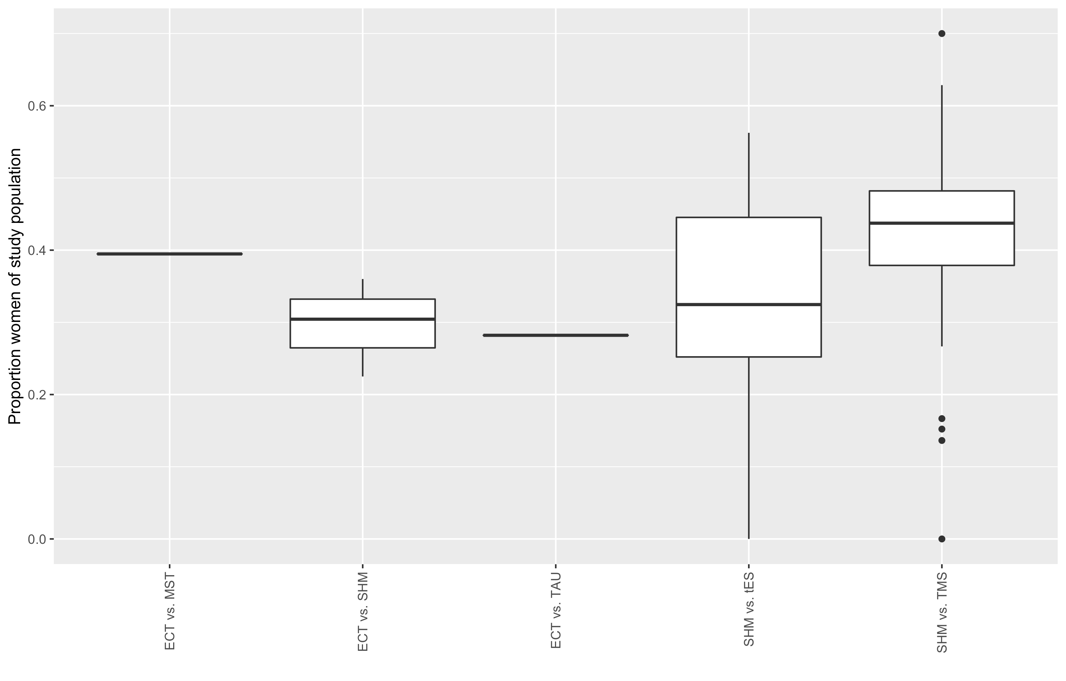
**

The boxplot shows the proportion of women across treatment comparisons. Despite some variability, most comparisons report proportions between 0.2 and 0.5. Whether women respond better or worse than men to NIBS is unclear, thus, sex is not a clear effect modifier. Our interpretation is no clear evidence of violations of the transitivity assumption.

## **7.8 Definition of treatment-resistance**

For the definition of treatment resistance, we accepted any study definition and categorized these definitions into minimum, moderate, and optimum cutoffs.

Within the minimum cutoff category, there were 40 comparisons, including 29 between TMS and sham stimulation, 8 between tES and sham stimulation, and 1 each for ECT and sham stimulation, low-charge vs. standard ECT, and low vs. high frequency rTMS.

The moderate cutoff category included 33 comparisons: 21 between TMS and sham stimulation, 8 between tES and sham stimulation, 2 between ECT and sham stimulation, 1 between ECT and treatment as usual (TAU), and 1 between rTMS and cTBS.

In the optimum cutoff category, there were 6 comparisons, which included 3 for TMS and sham stimulation, 1 each for ECT and sham stimulation, ECT and MST, and rTMS vs. cTBS.

## **7.9 Diagnosis (schizophrenia or schizoaffective disorder)**

For diagnosis, we included studies that involved individuals with treatment-resistant schizophrenia, schizoaffective disorder, or schizophreniform disorder. Among these, 50 comparisons involved the diagnosis of schizophrenia. These included 3 comparisons between ECT and sham stimulation, 1 between ECT and MST, 1 between ECT and TAU, 37 between TMS and sham stimulation, 6 between tES and sham stimulation, 1 between low-charge or standard ECT, and 1 between rTMS or cTBS.

Additionally, 28 comparisons included diagnoses other than schizophrenia. These comprised 15 TMS and sham stimulation comparisons, 10 tES and sham stimulation comparisons, 1 ECT and sham stimulation comparison, as well as one comparison between rTMS or cTBS, and one comparison between low or high frequency rTMS.

## **7.10 Use of PANSS for rating scales**

For the rating of overall symptoms, we accepted validated scales such as the Positive and Negative Syndrome Scale (PANSS), the Brief Psychiatric Rating Scale (BPRS), and others. Among the studies, 53 comparisons used the PANSS, which included 35 comparisons between TMS and sham stimulation, 12 between tES and sham stimulation, 2 between ECT and sham stimulation, 1 between ECT and MST, and one each for comparisons between rTMS or cTBS, low-charge versus standard ECT, low versus high frequency rTMS, and rTMS versus cTBS. Additionally, 7 comparisons used other validated scales besides the PANSS, including 2 between ECT and sham stimulation, 2 between tES and sham stimulation, 1 between ECT and treatment as usual (TAU), and 2 between TMS and sham stimulation.

## **7.11 Blinding status**

For blinding, we accepted at least single-blind conditions where assessors were unaware of the treatment allocations. A total of 68 comparisons were conducted under a double-blinded design, which included 48 TMS and sham stimulation comparisons, 15 tES and sham stimulation comparisons, 2 ECT and sham stimulation comparisons, and one each for comparisons between low-charge or standard ECT, rTMS or cTBS, and low versus high frequency rTMS.

Additionally, 10 comparisons were under a single-blinded design, including 4 TMS and sham stimulation comparisons, 1 tES and sham stimulation comparison, 2 ECT and sham stimulation comparisons, 1 ECT and treatment as usual (TAU) comparison, 1 ECT and MST comparison, and one comparison between rTMS or cTBS.

## **7.12 Sponsorship**

For sponsorship, we examined whether the studies were funded by pharmaceutical or brain stimulation companies. A total of 3 comparisons were conducted under such sponsorship, which included 1 ECT and sham stimulation comparison, 1 tES and sham stimulation comparison, and 1 TMS and sham stimulation.

**References**

1. Leucht S, Kane JM, Kissling W, Hamann J, Etschel E, Engel RR. What does the PANSS mean?. Schizophr Res. 2005;79(2-3):231-238.

# **8. Results of the primary outcome: overall symptoms**

For the primary outcome overall symptoms, we present below:

-  Network plot

-  Forest-plot of results of network-meta-analysis (reference sham stimulation)

-  League-table of results of the network meta-analysis

-  Forest-plot of results of pairwise meta-analysis

To assess the potential impact of including studies from Chinese mainland, we presented the results both with and without data from these studies.

Legend for network plots: lines link treatments with direct comparisons in trials; thickness of lines corresponds to the number of trials evaluating the comparison; size of the nodes corresponds to the number of trials investigating the treatment.

Legend for forest-plots of results of network-meta-analysis: effect sizes are from the network-meta-analysis. The type of effect size measure is standardised mean difference (SMD). Order of treatments is according to the mean effect size. Reference is sham stimulation. The direction of the effect is indicated below the x-axis. In general, effect estimates to the left are in favor of NIBS treatment.

Legend for league tables: Order of treatments is according to the mean effect size. Results of the network-meta-analysis are presented in the left lower half and results of pairwise meta-analyses in the right upper half. Results in bold indicate 95% CI excluding no effect.

Legend for forest-plots of results of pairwise meta-analysis: Studies are ordered by comparison investigated (in alphabetical order) and a summary effect size is calculated by pairwise meta-analyses of all studies of a specific comparison. The type of effect size measure is standardised mean difference (SMD). Both fixed- and random-effects estimates were presented for sensitivity comparison. < 0 means better outcome with the first intervention in the comparison.

Abbreviations: ECT: Electroconvulsive therapy; MST: Magnetic seizure therapy; SHM: Sham therapy; TAU: Treatment as Usual; tES: Transcranial electrical stimulation; TMS: transcranial magnetic stimulation.


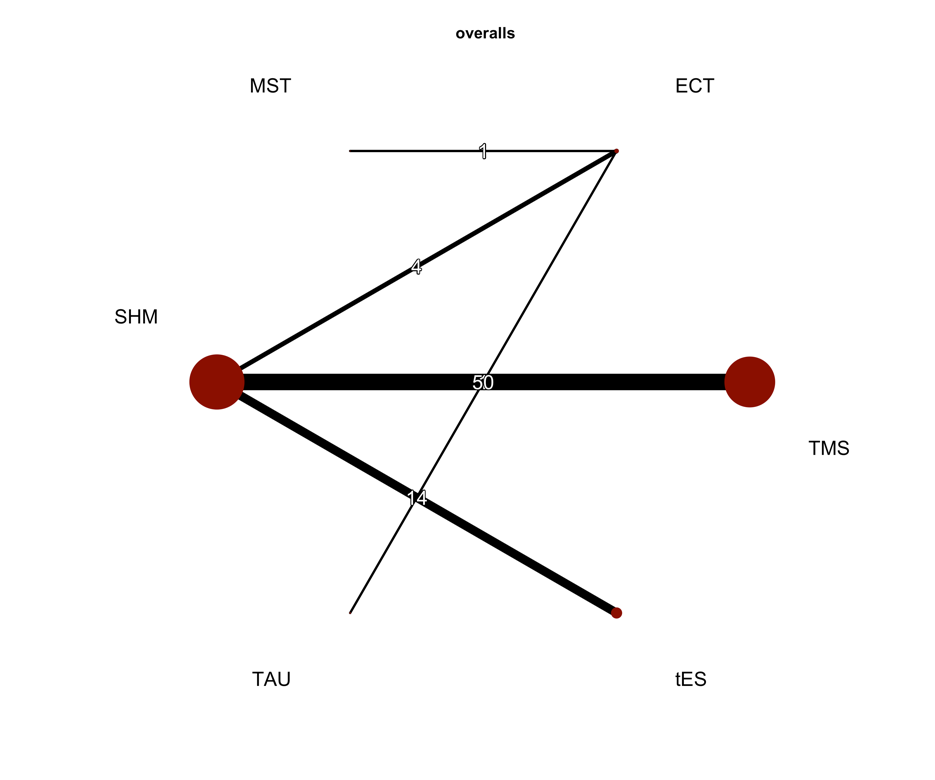


Network plot including studies from Chinese mainland.


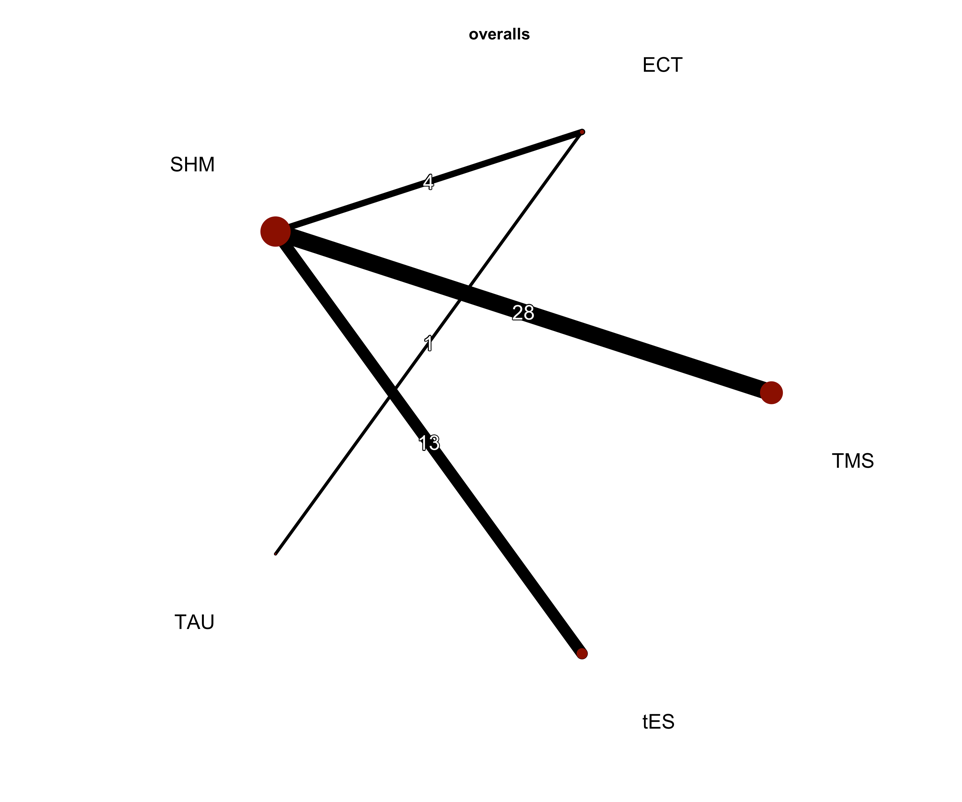


Network plot excluding studies from Chinese mainland.


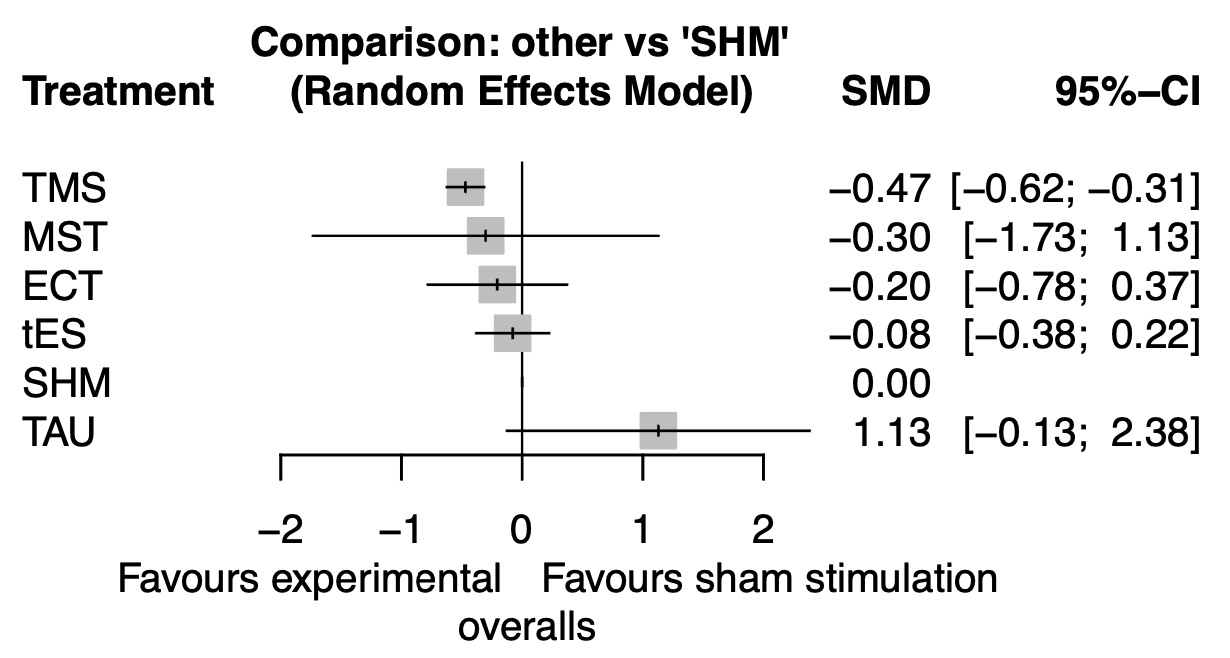


Forest-plot of results of network-meta-analysis including studies from Chinese mainland.

Quantifying heterogeneity / inconsistency:

tau^2 = 0.1971; tau = 0.4439; I^2 = 66.3% [56.4%; 74.0%]


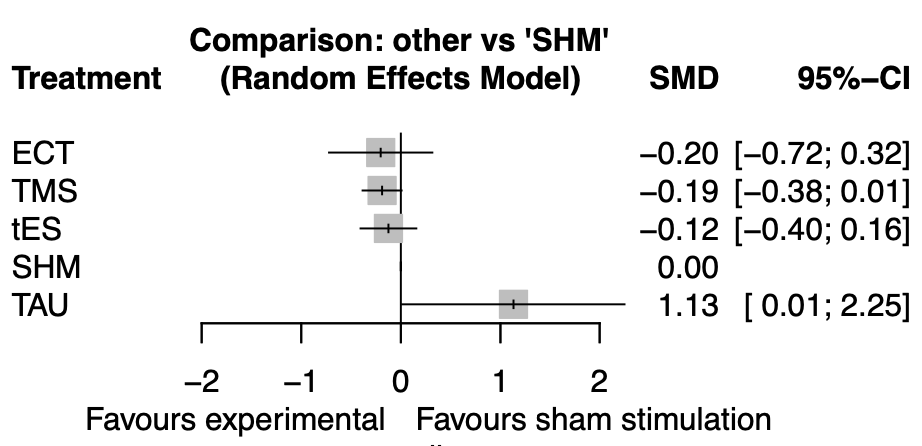


Forest-plot of results of network-meta-analysis excluding studies from Chinese mainland.

Quantifying heterogeneity / inconsistency:

tau^2 = 0.1303; tau = 0.3610; I^2 = 51.2% [30.8%; 65.7%]

| TMS | . | . | . | -0.47 (-0.62,-0.31) | . |
| --- | --- | --- | --- | --- | --- |
| -0.17 (-1.61, 1.27) | MST | -0.10 (-1.41, 1.21) | . | . | . |
| -0.26 (-0.86, 0.33) | -0.10 (-1.41, 1.21) | ECT | . | -0.20 (-0.78, 0.37) | -1.33 (-2.45,-0.22) |
| -0.39 (-0.73,-0.05) | -0.22 (-1.69, 1.24) | -0.13 (-0.78, 0.53) | tES | -0.08 (-0.38, 0.22) | . |
| -0.47 (-0.62,-0.31) | -0.30 (-1.73, 1.13) | -0.20 (-0.78, 0.37) | -0.08 (-0.38, 0.22) | SHM | . |
| -1.60 (-2.86,-0.33) | -1.43 (-3.15, 0.29) | -1.33 (-2.45,-0.22) | -1.21 (-2.50, 0.08) | -1.13 (-2.38, 0.13) | TAU |

League-table of results of the network meta-analysis including studies from Chinese mainland.

| ECT | . | . | -0.20 (-0.72, 0.32) | -1.33 (-2.32,-0.34) |
| --- | --- | --- | --- | --- |
| -0.01 (-0.57, 0.54) | TMS | . | -0.19 (-0.38, 0.01) | . |
| -0.08 (-0.67, 0.51) | -0.06 (-0.41, 0.28) | tES | -0.12 (-0.40, 0.16) | . |
| -0.20 (-0.72, 0.32) | -0.19 (-0.38, 0.01) | -0.12 (-0.40, 0.16) | SHM | . |
| -1.33 (-2.32,-0.34) | -1.32 (-2.46,-0.18) | -1.26 (-2.41,-0.10) | -1.13 (-2.25,-0.01) | TAU |

League-table of results of the network meta-analysis excluding studies from Chinese mainland.


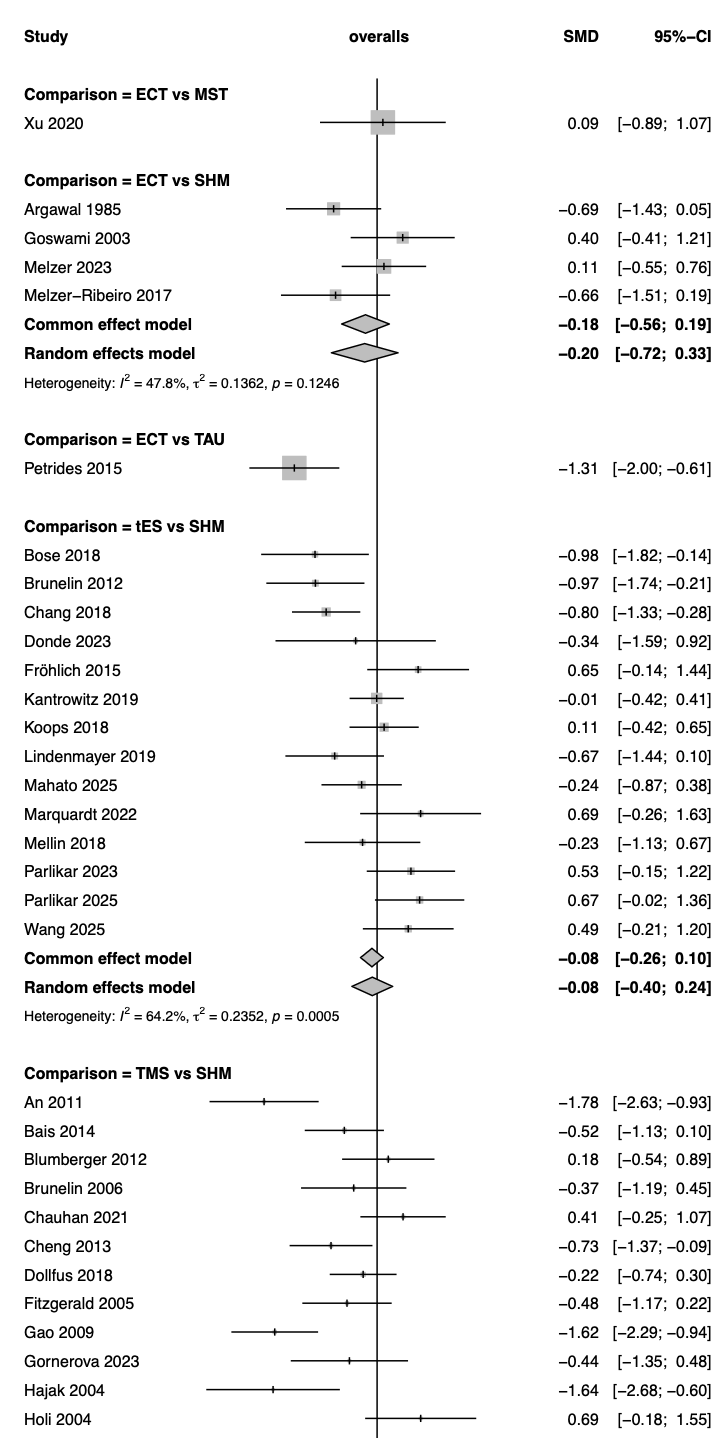


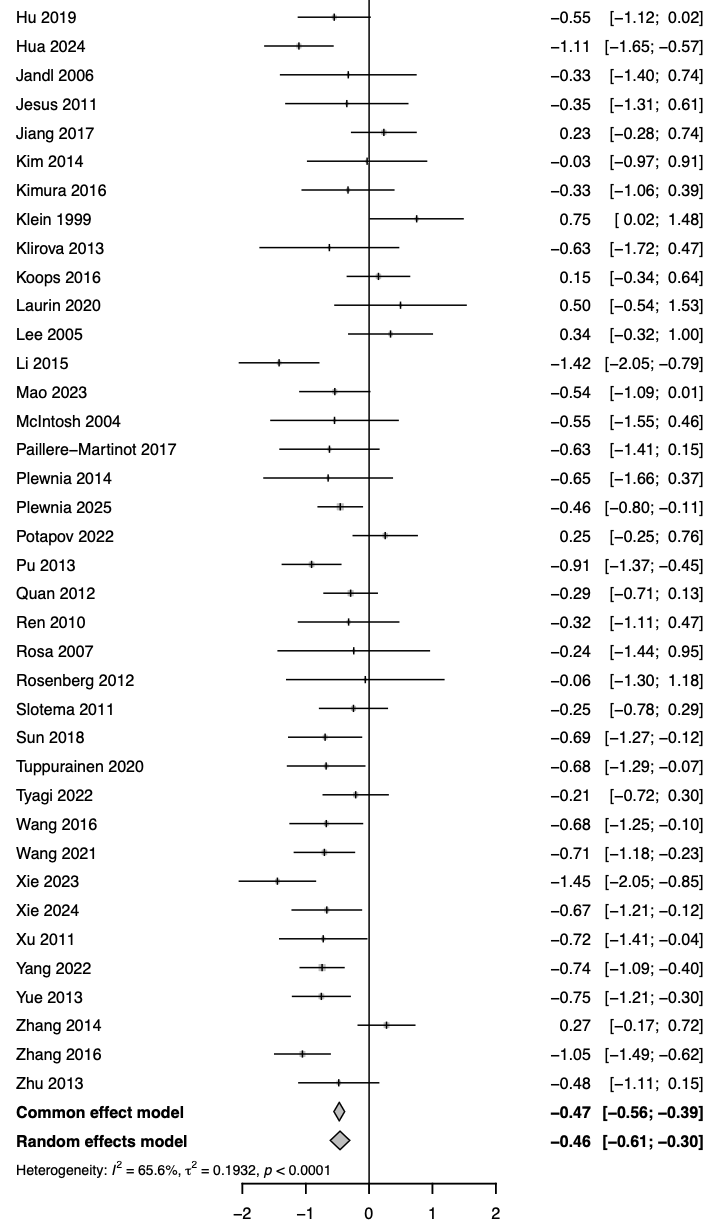


Forest-plot of results of pairwise meta-analyses including studies from Chinese mainland.


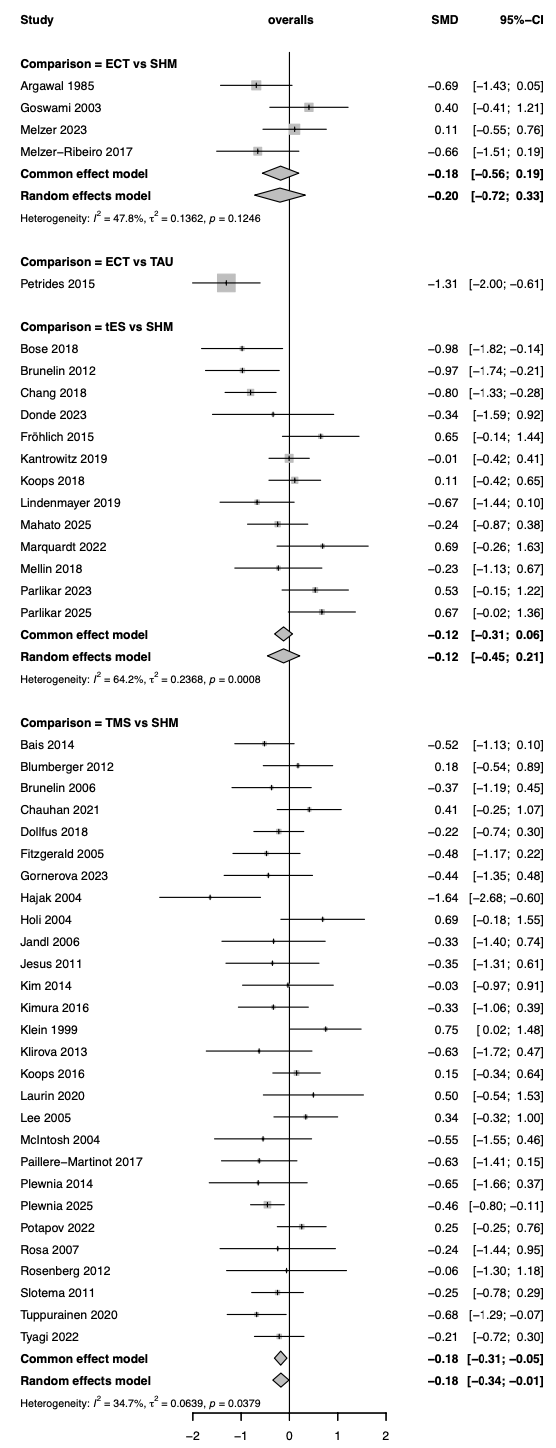


Forest-plot of results of pairwise meta-analyses excluding studies from Chinese mainland.

# **9. Results of network and pairwise meta-analyses of secondary outcomes**

For each outcome we present below:

-  Network plot

-  Forest-plot of results of network-meta-analysis (reference sham stimulation)

-  League-table of results of the network meta-analysis

-  Forest-plot of results of pairwise meta-analyses

To assess the potential impact of including studies from Chinese mainland, we presented the results both with and without data from these studies.

When fewer than 10 studies reported on the secondary outcomes, we presented only the forest plots from the pairwise meta-analyses.

Legend for network plots: lines link treatments with direct comparisons in trials; thickness of lines corresponds to the number of trials evaluating the comparison; size of the nodes corresponds to the number of trials investigating the treatment.

Legend for forest-plots of results of network-meta-analysis: effect sizes are from the network-meta-analysis. The type of effect size measure is standardised mean difference (SMD) or odds ratio (OR). Order of treatments is according to the mean effect size. Reference is sham stimulation. The direction of the effect is indicated below the x-axis. In general, effect estimates to the left are in favor of NIBS treatment.

Legend for league tables: Order of treatments is according to the mean effect size. Results of the network-meta-analysis are presented in the left lower half and results of pairwise meta-analyses in the right upper half. Results in bold indicate 95% CI excluding no effect.

Legend for forest-plots of results of pairwise meta-analysis: Studies are ordered by comparison investigated (in alphabetical order) and a summary effect size is calculated by pairwise meta-analyses of all studies of a specific comparison. The type of effect size measure is standardised mean difference (SMD) or odds ratio (OR). Both fixed- and random-effects estimates were presented for sensitivity comparison. For rare event data, we used the standard inverse variance method instead of Mantel–Haenszel with continuity correction of 0.5 to ensure that studies with zero events in one or both arms were included. This was done intentionally and is acknowledged here to make all the data visible. For continuous data, < 0 means better outcome with the first intervention in the comparison; For dichotomous data, < 1 means less events with the first intervention in the comparison.

Abbreviations: ECT: Electroconvulsive therapy; MST: Magnetic seizure therapy; SHM: Sham therapy; TAU: Treatment as Usual; tES: Transcranial electrical stimulation; TMS: transcranial magnetic stimulation.

##
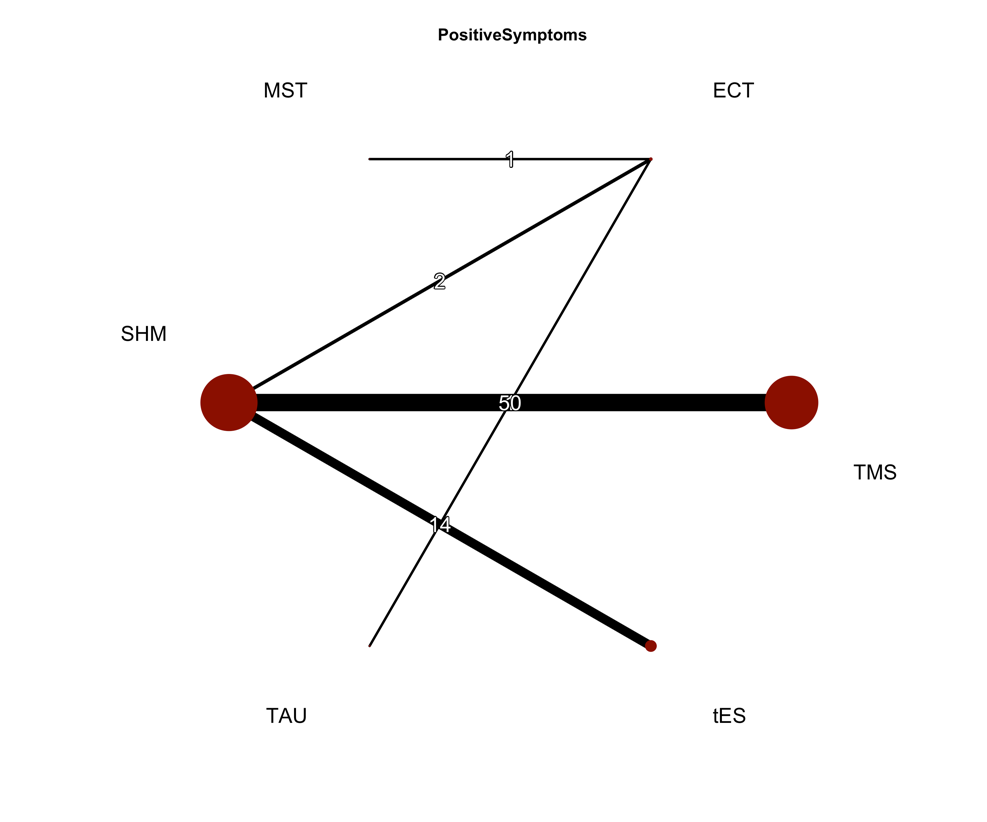
**9.1 Positive symptoms**

Network plot including studies from Chinese mainland.


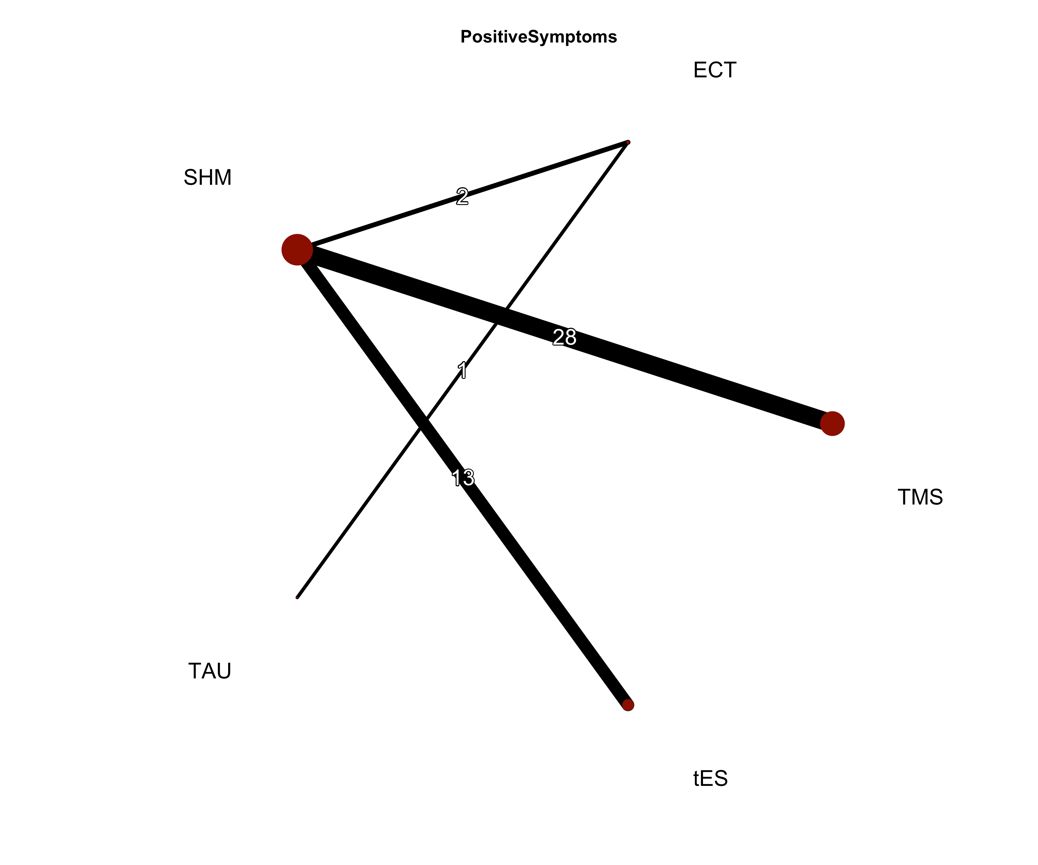


Network plot excluding studies from Chinese mainland.


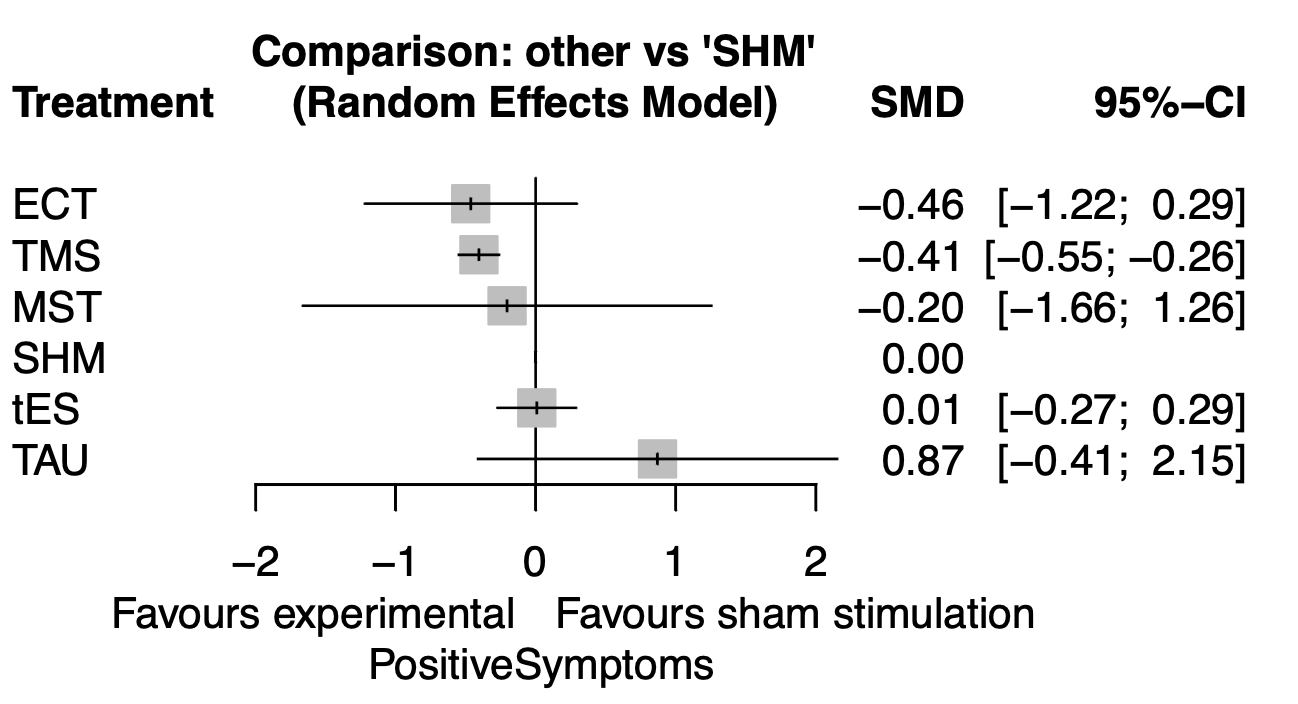


Forest-plot of results of network-meta-analysis including studies from Chinese mainland.

Quantifying heterogeneity / inconsistency:

tau^2 = 0.1534; tau = 0.3917; I^2 = 61% [48.8%; 70.3%]


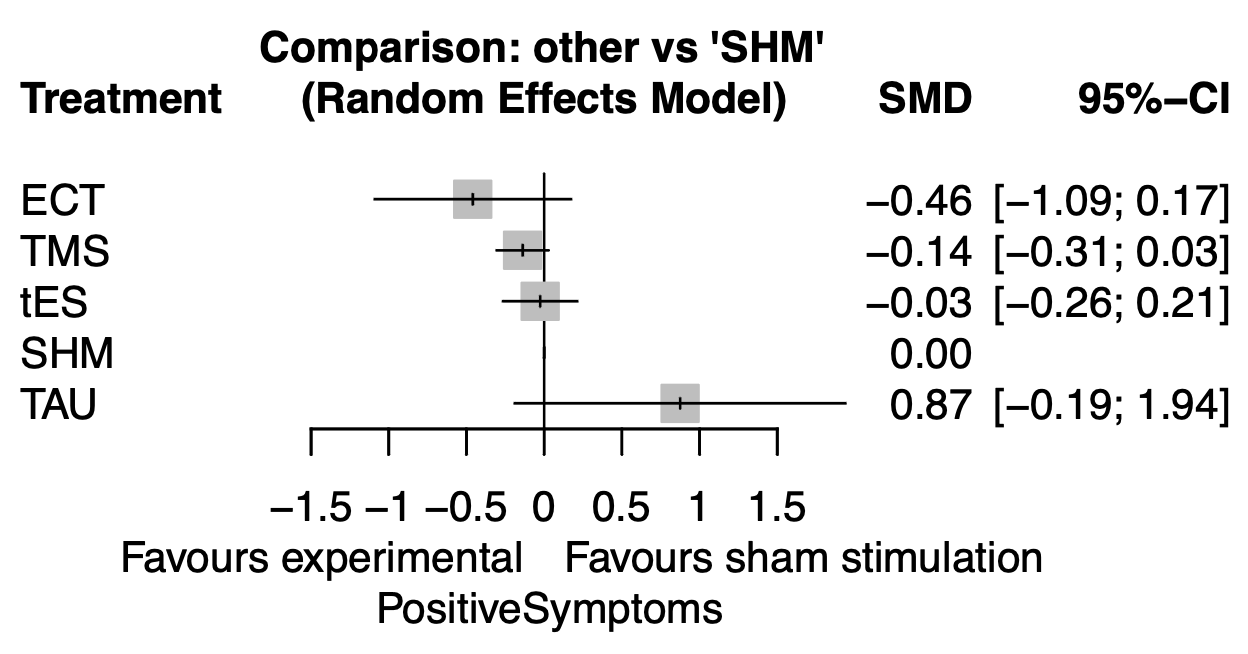


Forest-plot of results of network-meta-analysis excluding studies from Chinese mainland.

Quantifying heterogeneity / inconsistency:

tau^2 = 0.0652; tau = 0.2553; I^2 = 34.8% [4.4%; 55.6%]

| ECT | . | -0.26 (-1.51, 0.99) | -0.46 (-1.22, 0.29) | . | -1.33 (-2.37,-0.30) |
| --- | --- | --- | --- | --- | --- |
| -0.06 (-0.83, 0.71) | TMS | . | -0.41 (-0.55,-0.26) | . | . |
| -0.26 (-1.51, 0.99) | -0.20 (-1.67, 1.27) | MST | . | . | . |
| -0.46 (-1.22, 0.29) | -0.41 (-0.55,-0.26) | -0.20 (-1.66, 1.26) | SHM | -0.01 (-0.29, 0.27) | . |
| -0.47 (-1.28, 0.34) | -0.41 (-0.73,-0.10) | -0.21 (-1.70, 1.27) | -0.01 (-0.29, 0.27) | tES | . |
| -1.33 (-2.37,-0.30) | -1.28 (-2.57, 0.01) | -1.07 (-2.70, 0.55) | -0.87 (-2.15, 0.41) | -0.86 (-2.17, 0.45) | TAU |

League-table of results of the network meta-analysis including studies from Chinese mainland.

| ECT | . | . | -0.46 (-1.09, 0.17) | -1.33 (-2.19,-0.48) |
| --- | --- | --- | --- | --- |
| -0.32 (-0.97, 0.33) | TMS | . | -0.14 (-0.31, 0.03) | . |
| -0.43 (-1.11, 0.24) | -0.11 (-0.40, 0.18) | tES | -0.03 (-0.26, 0.21) | . |
| -0.46 (-1.09, 0.17) | -0.14 (-0.31, 0.03) | -0.03 (-0.26, 0.21) | SHM | . |
| -1.33 (-2.19,-0.48) | -1.01 (-2.09, 0.06) | -0.90 (-1.99, 0.19) | -0.87 (-1.94, 0.19) | TAU |

League-table of results of the network meta-analysis excluding studies from Chinese mainland.


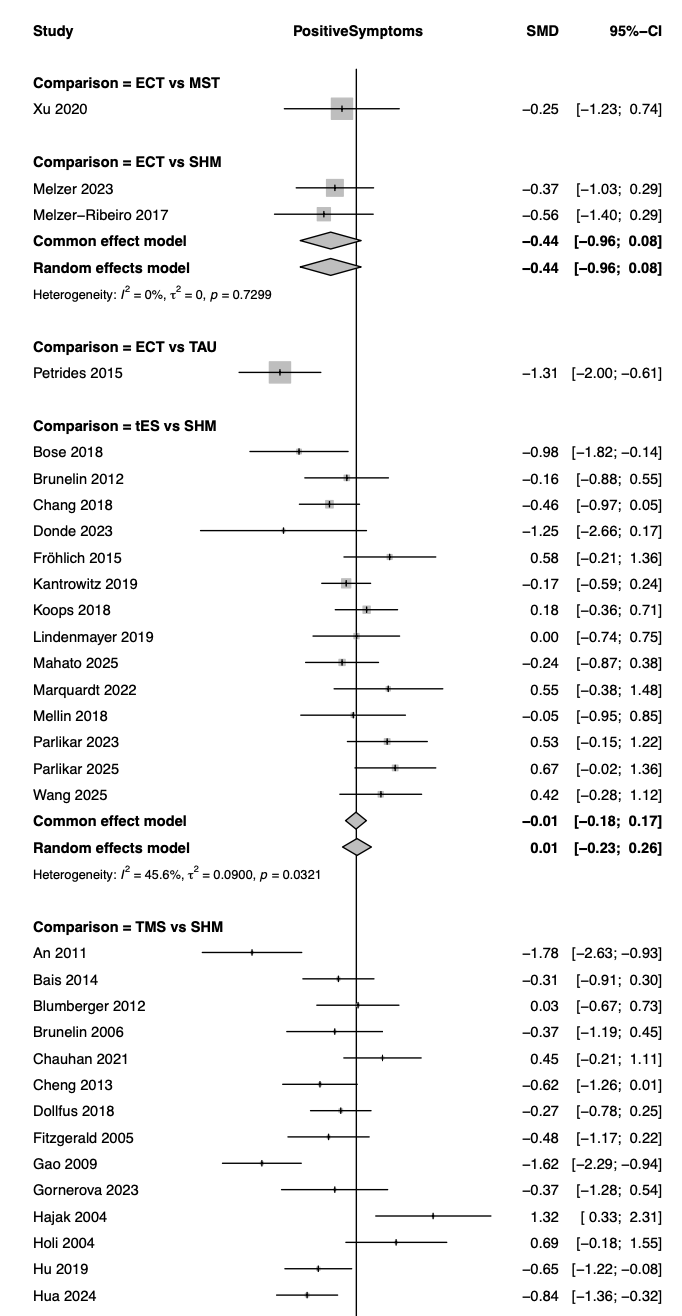


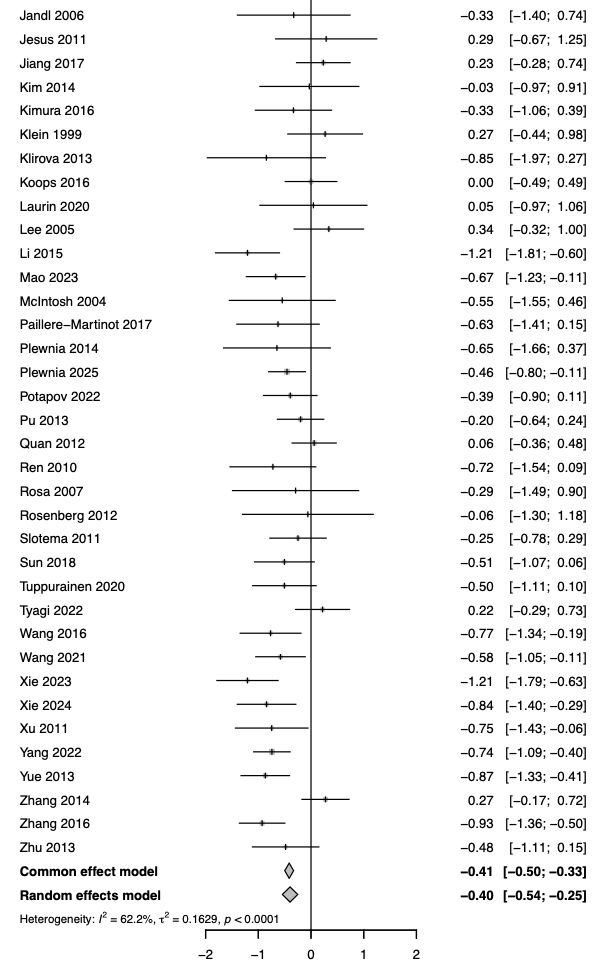


Forest-plot of results of pairwise meta-analyses including studies from Chinese mainland.


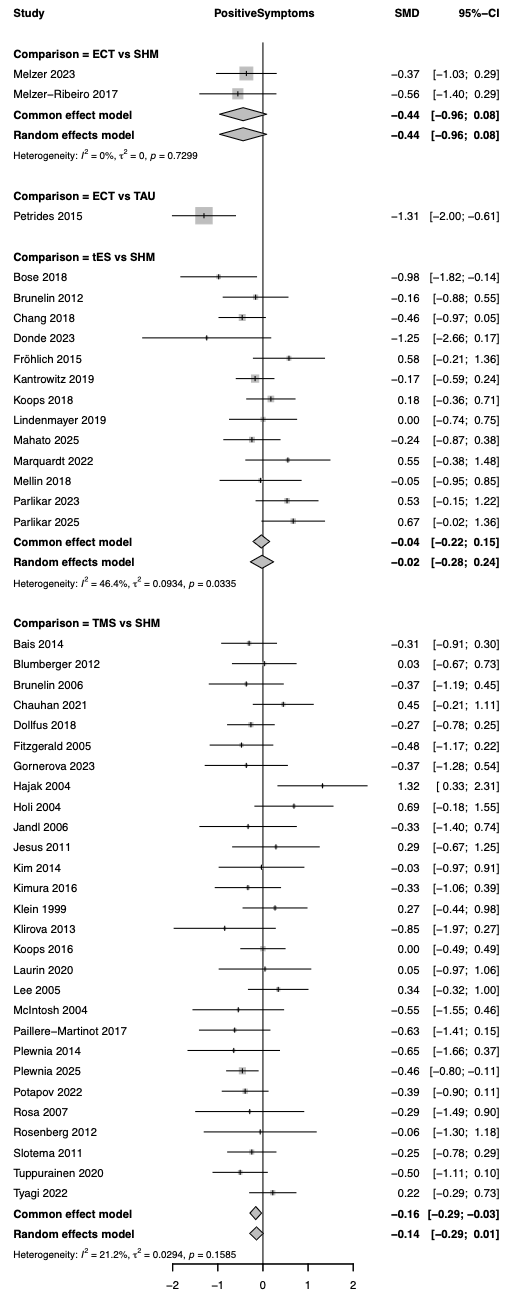


Forest-plot of results of pairwise meta-analyses excluding studies from Chinese mainland.

## **9.2 Negative symptoms**


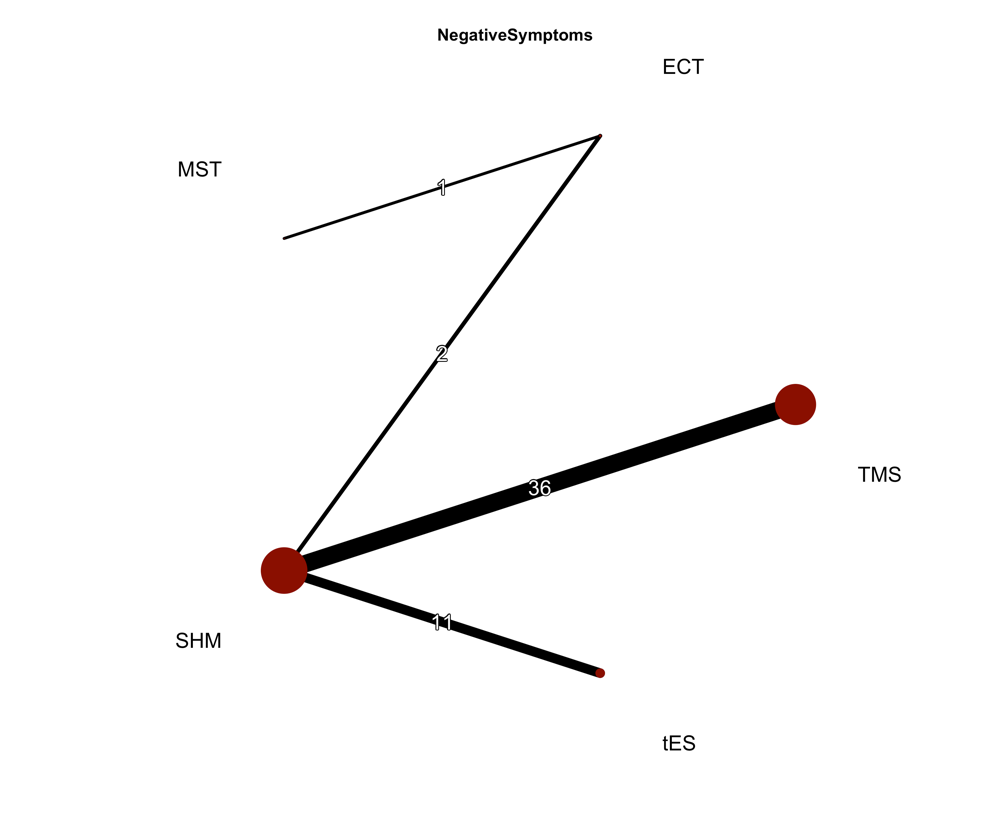


Network plot including studies from Chinese mainland.


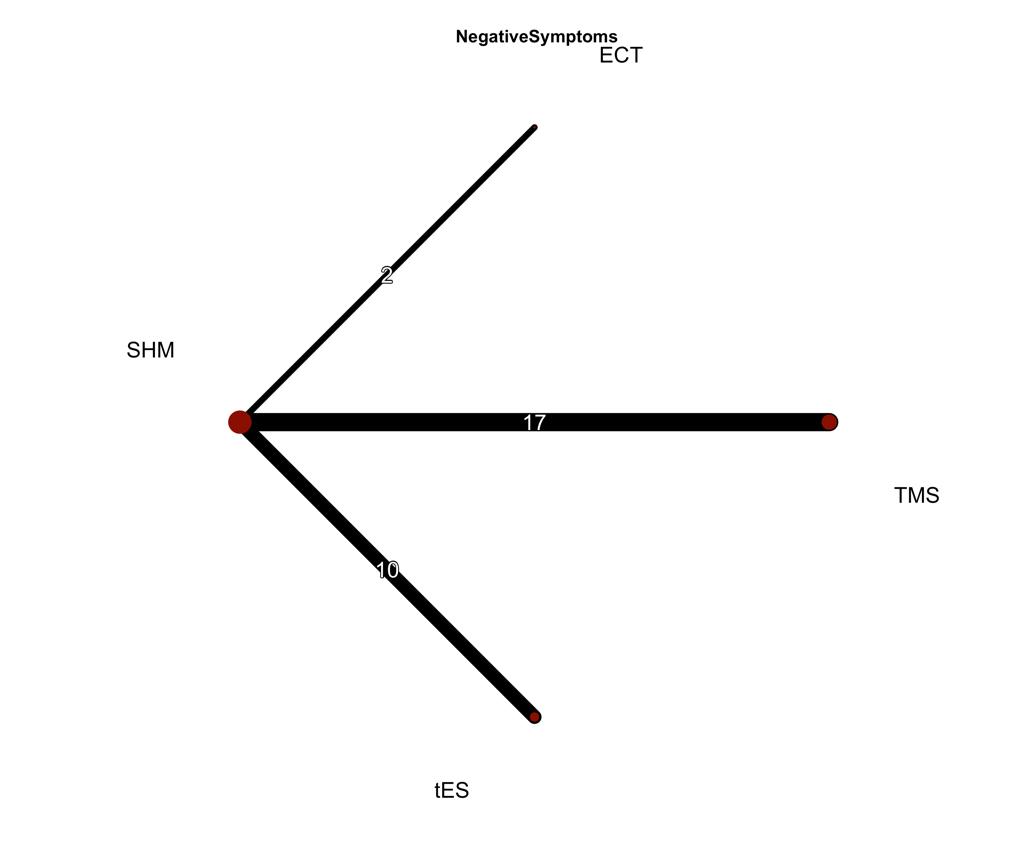


Network plot excluding studies from Chinese mainland.


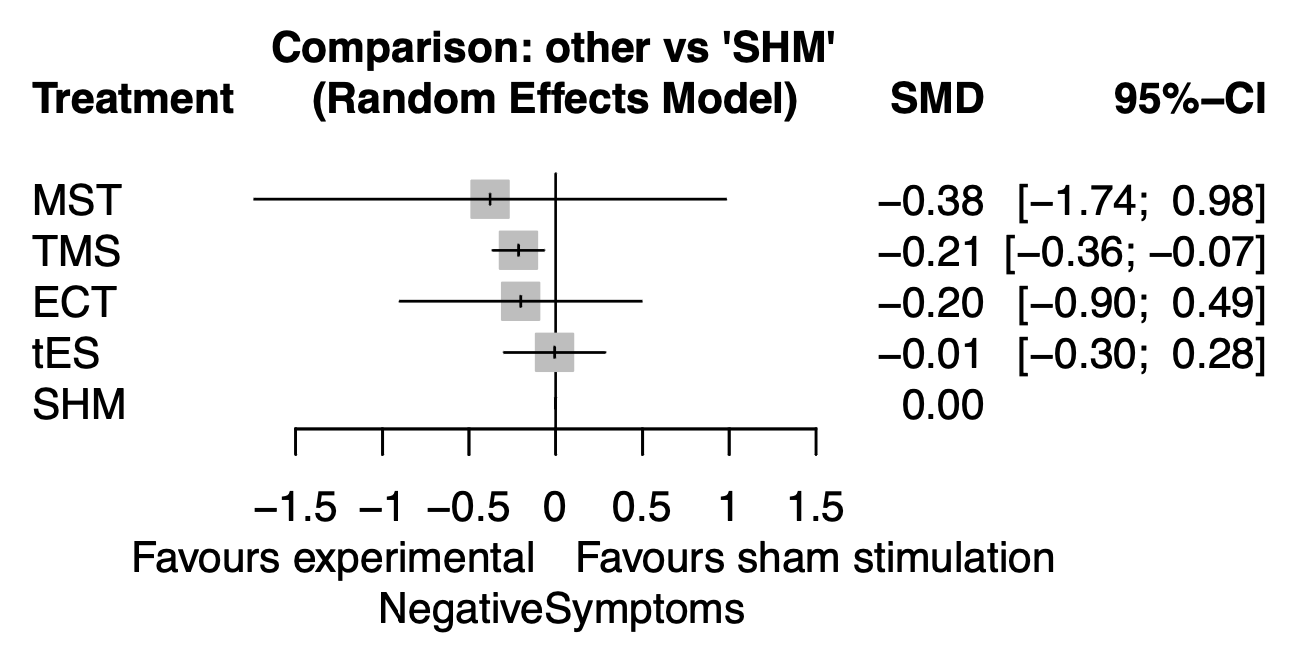


Forest-plot of results of network-meta-analysis including studies from Chinese mainland.

Quantifying heterogeneity / inconsistency:

tau^2 = 0.1037; tau = 0.3220; I^2 = 53.6% [35.4%; 66.7%]


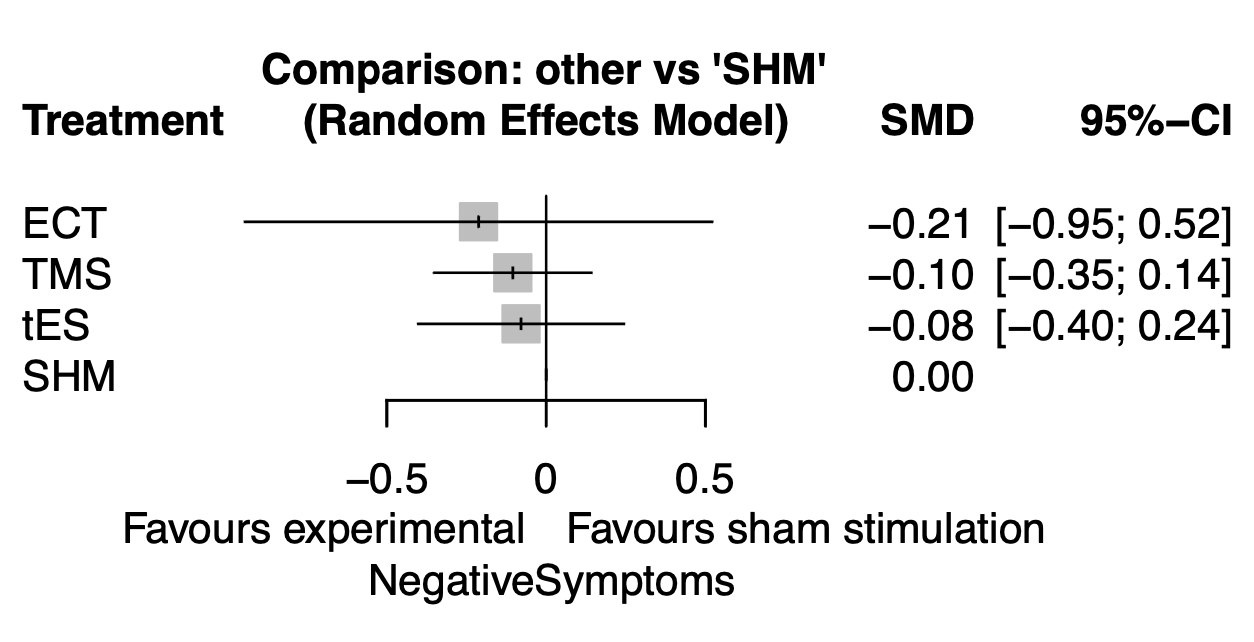


Forest-plot of results of network-meta-analysis excluding studies from Chinese mainland.

Quantifying heterogeneity / inconsistency:

tau^2 = 0.1313; tau = 0.3623; I^2 = 52.8% [26.8%; 69.6%]

| MST | . | -0.18 (-1.34, 0.99) | . | . |
| --- | --- | --- | --- | --- |
| -0.16 (-1.53, 1.20) | TMS | . | . | -0.21 (-0.36,-0.07) |
| -0.18 (-1.34, 0.99) | -0.01 (-0.72, 0.70) | ECT | . | -0.20 (-0.90, 0.49) |
| -0.37 (-1.76, 1.02) | -0.21 (-0.53, 0.12) | -0.20 (-0.95, 0.56) | tES | -0.01 (-0.30, 0.28) |
| -0.38 (-1.74, 0.98) | -0.21 (-0.36,-0.07) | -0.20 (-0.90, 0.49) | -0.01 (-0.30, 0.28) | SHM |

League-table of results of the network meta-analysis including studies from Chinese mainland.

| ECT | . | . | -0.21 (-0.95,0.52) |
| --- | --- | --- | --- |
| -0.11 (-0.88,0.67) | TMS | . | -0.10 (-0.35,0.14) |
| -0.13 (-0.93,0.67) | -0.03 (-0.43,0.38) | tES | -0.08 (-0.40,0.24) |
| -0.21 (-0.95,0.52) | -0.10 (-0.35,0.14) | -0.08 (-0.40,0.24) | SHM |

League-table of results of the network meta-analysis excluding studies from Chinese mainland.


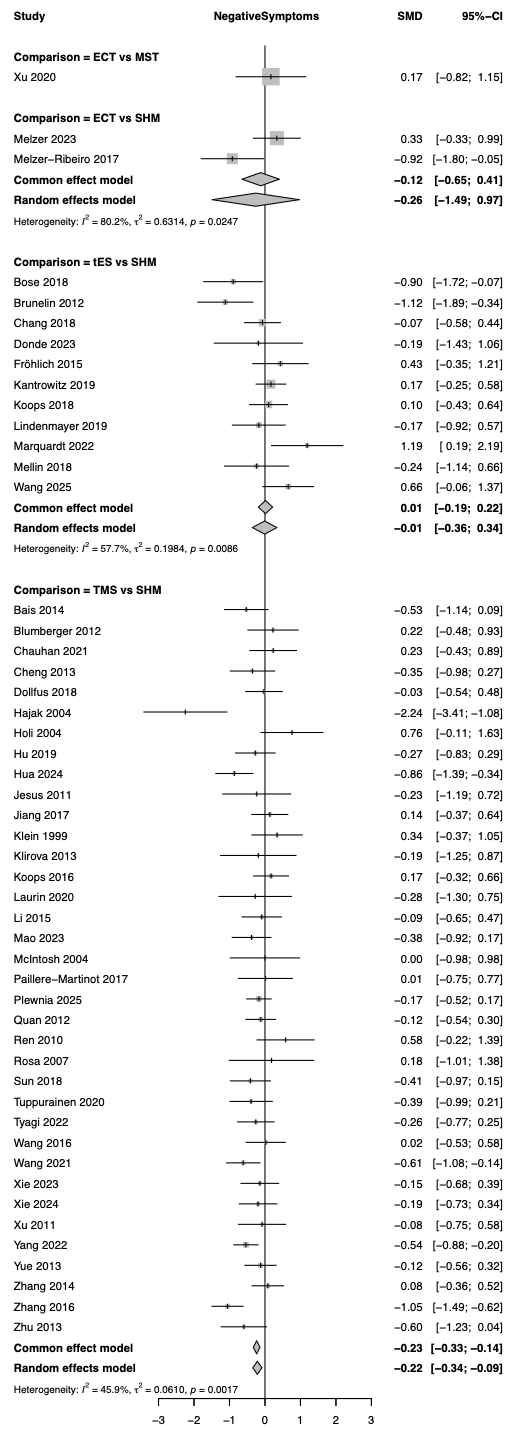
Forest-plot of results of pairwise meta-analyses including studies from Chinese mainland.


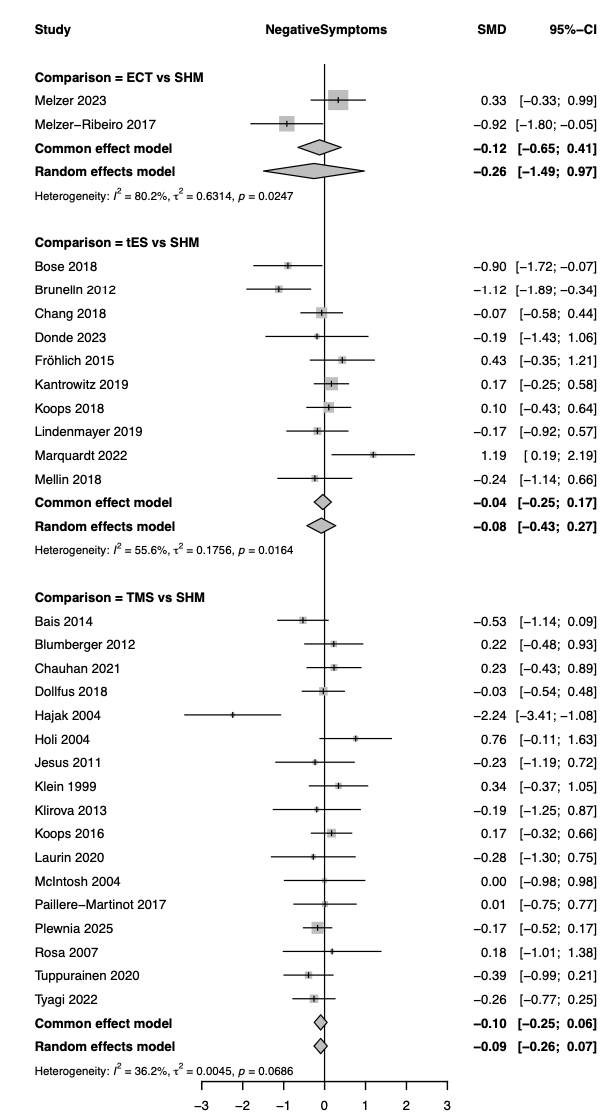


Forest-plot of results of pairwise meta-analyses excluding studies from Chinese mainland.

## **9.3 Hallucination**


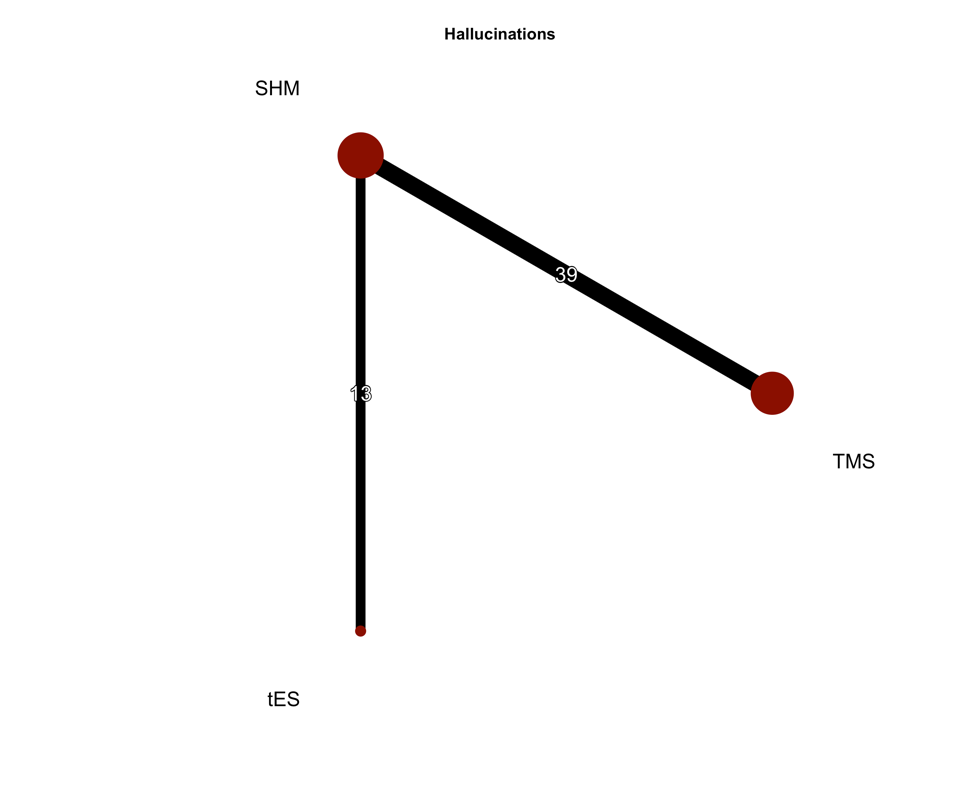


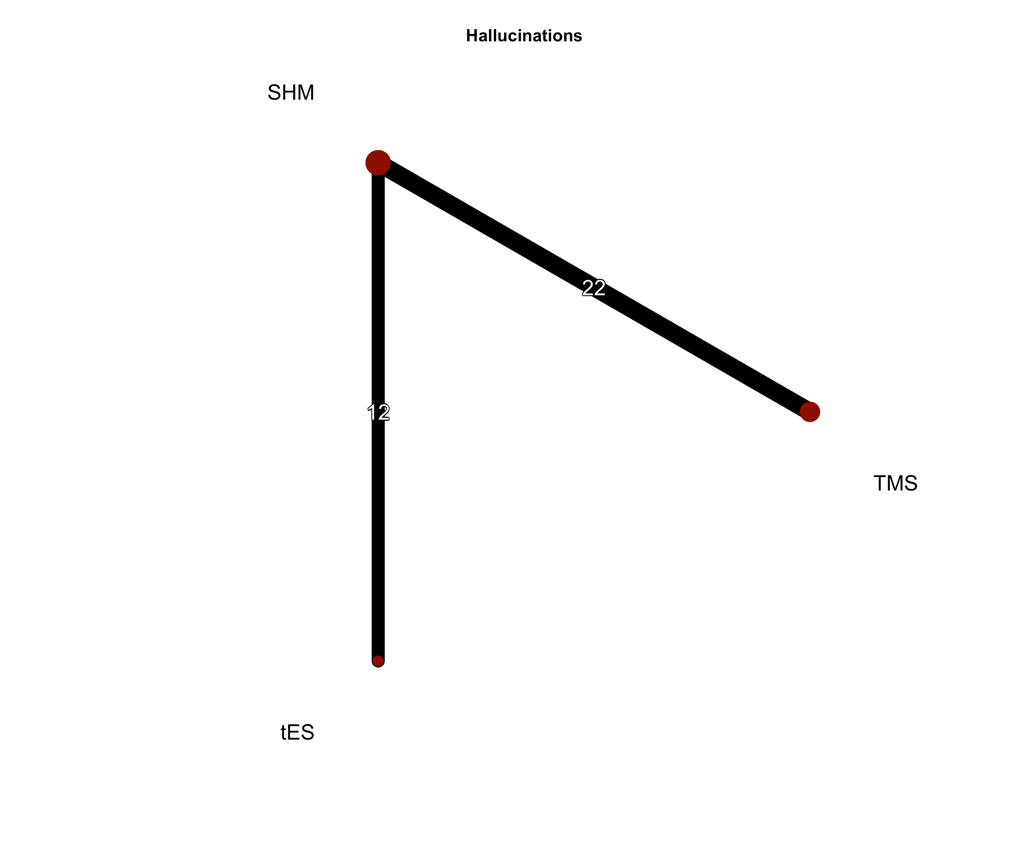
Network plot including studies from Chinese mainland.

Network plot excluding studies from Chinese mainland.


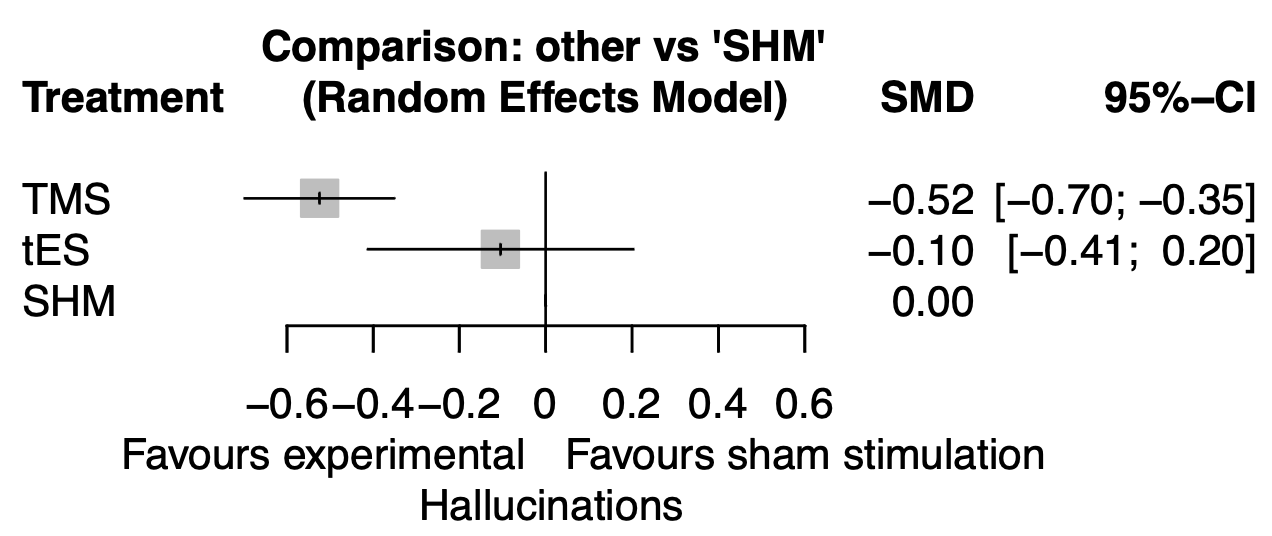


Forest-plot of results of network-meta-analysis including studies from Chinese mainland.

Quantifying heterogeneity / inconsistency:

tau^2 = 0.1923; tau = 0.4385; I^2 = 66.8% [55.6%; 75.2%]


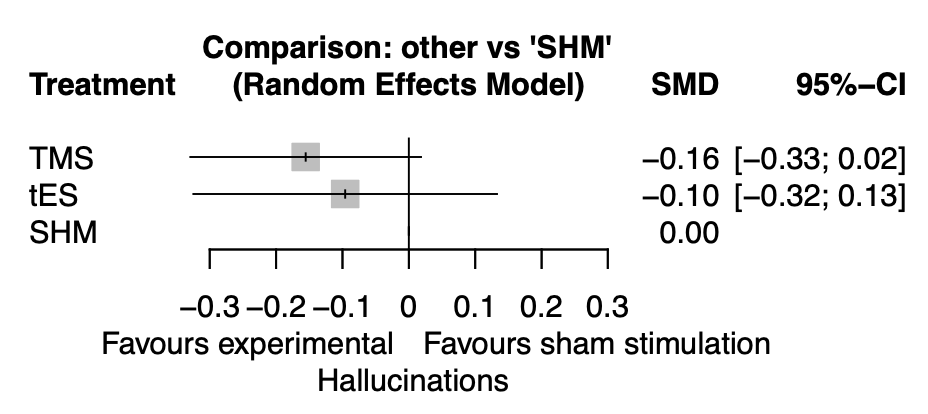


Forest-plot of results of network-meta-analysis excluding studies from Chinese mainland.

Quantifying heterogeneity / inconsistency:

tau^2 = 0.0435; tau = 0.2085; I^2 = 27.2% [0.0%; 52.8%]

| TMS | . | -0.52 (-0.70,-0.35) |
| --- | --- | --- |
| -0.42 (-0.77,-0.07) | tES | -0.10 (-0.41, 0.20) |
| -0.52 (-0.70,-0.35) | -0.10 (-0.41, 0.20) | SHM |

League-table of results of the network meta-analysis including studies from Chinese mainland.

| TMS | . | -0.16 (-0.33,0.02) |
| --- | --- | --- |
| -0.06 (-0.35,0.23) | tES | -0.10 (-0.32,0.13) |
| -0.16 (-0.33,0.02) | -0.10 (-0.32,0.13) | SHM |

League-table of results of the network meta-analysis excluding studies from Chinese mainland.


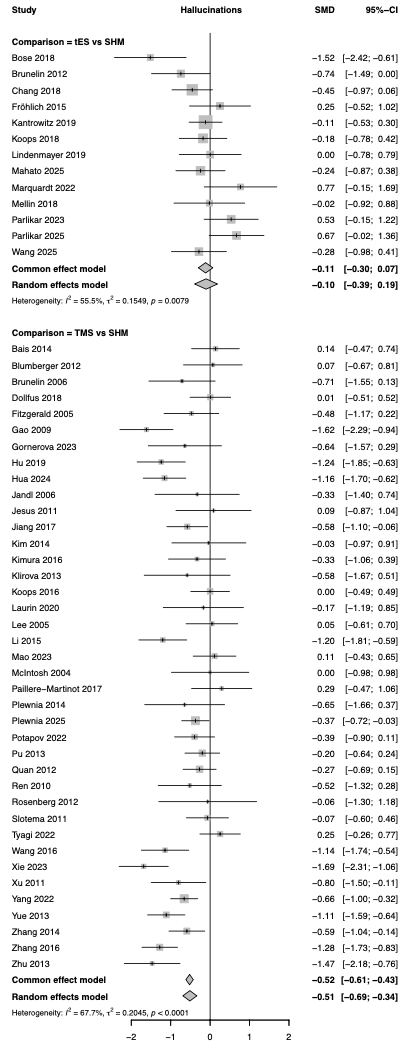


Forest-plot of results of pairwise meta-analyses including studies from Chinese mainland.


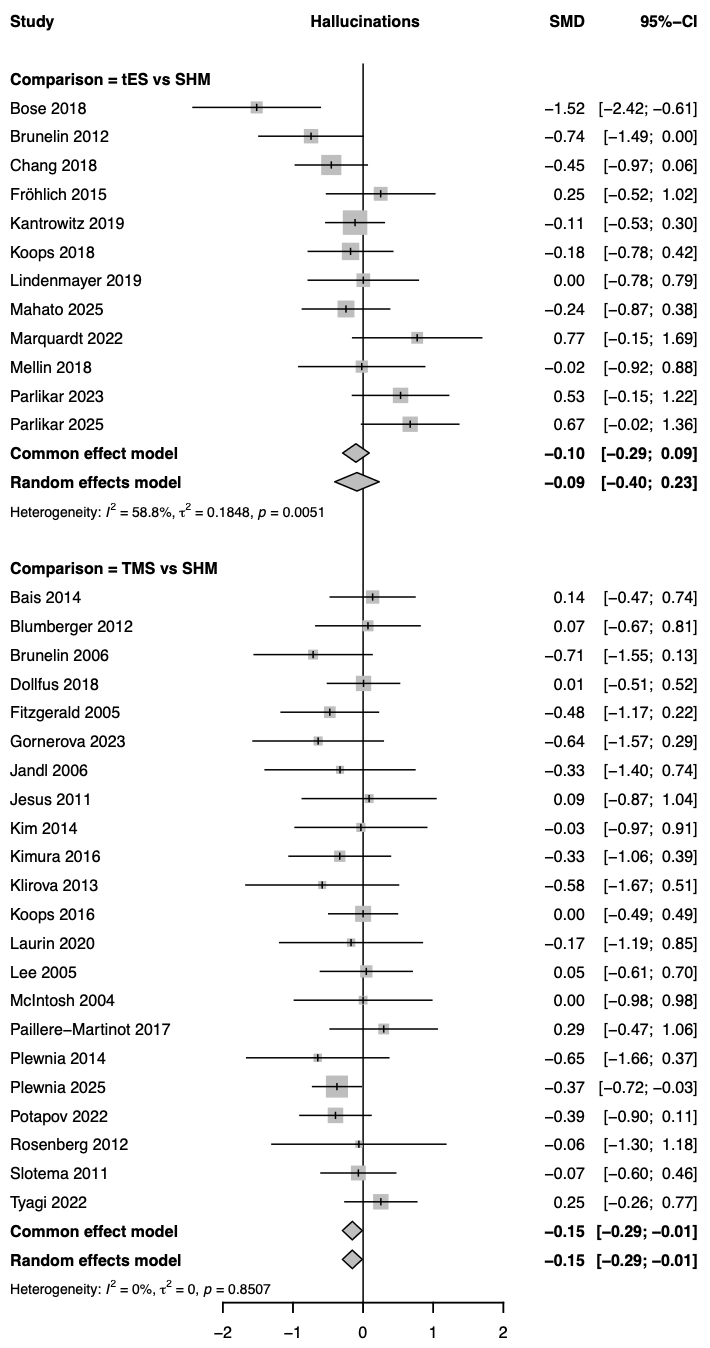


Forest-plot of results of pairwise meta-analyses excluding studies from Chinese mainland.

## **9.4 Depression**


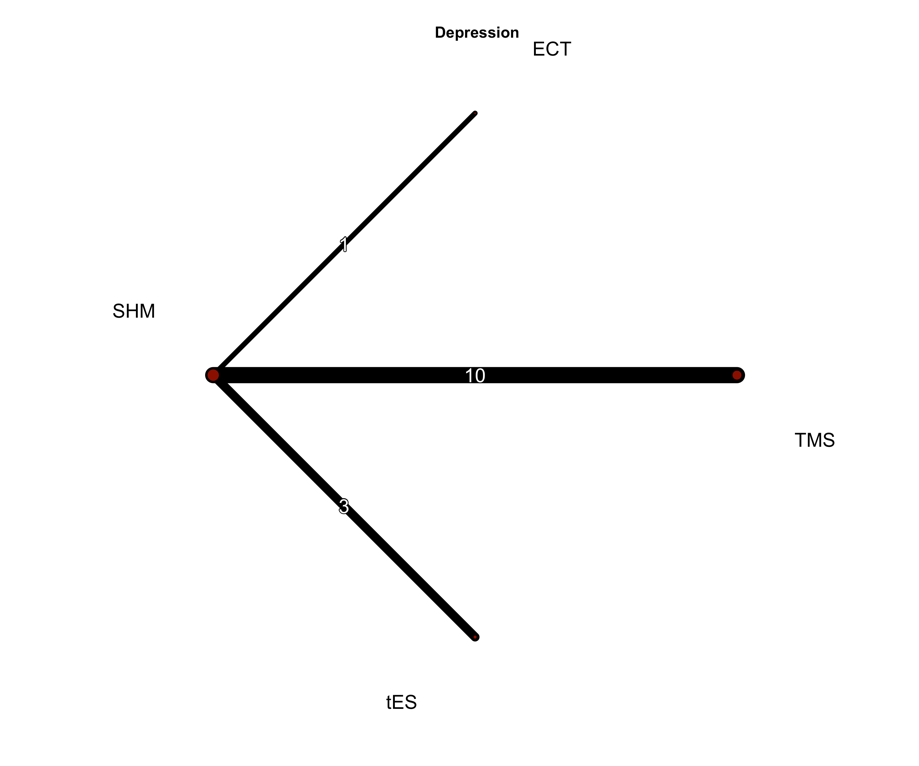


Network plot including studies from Chinese mainland.


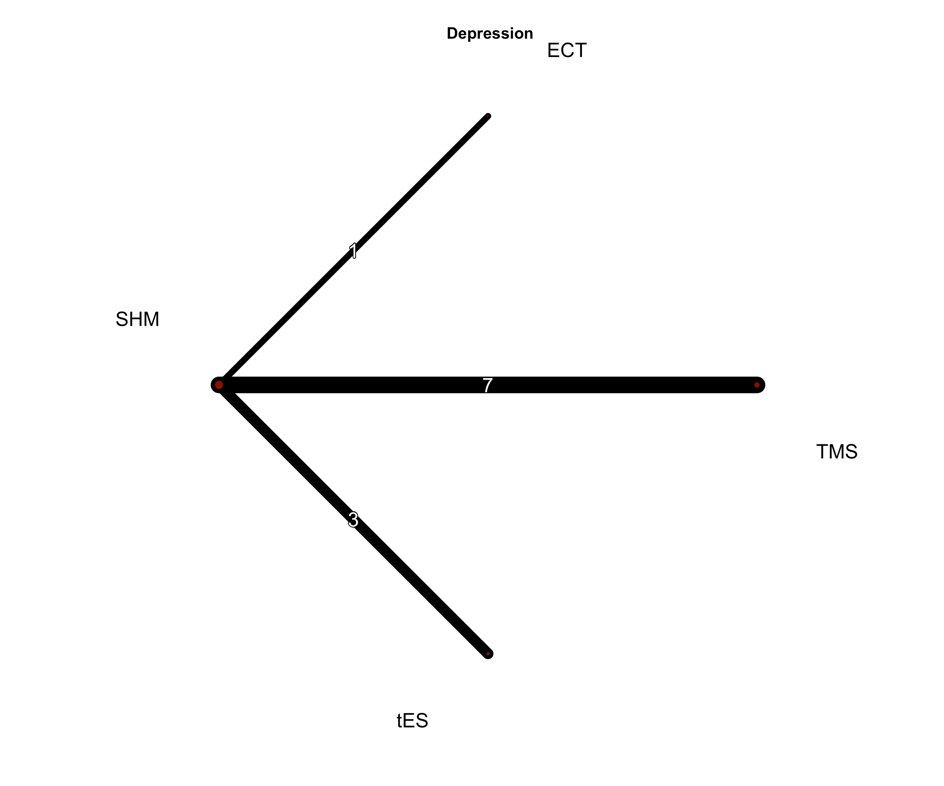


Network plot excluding studies from Chinese mainland.


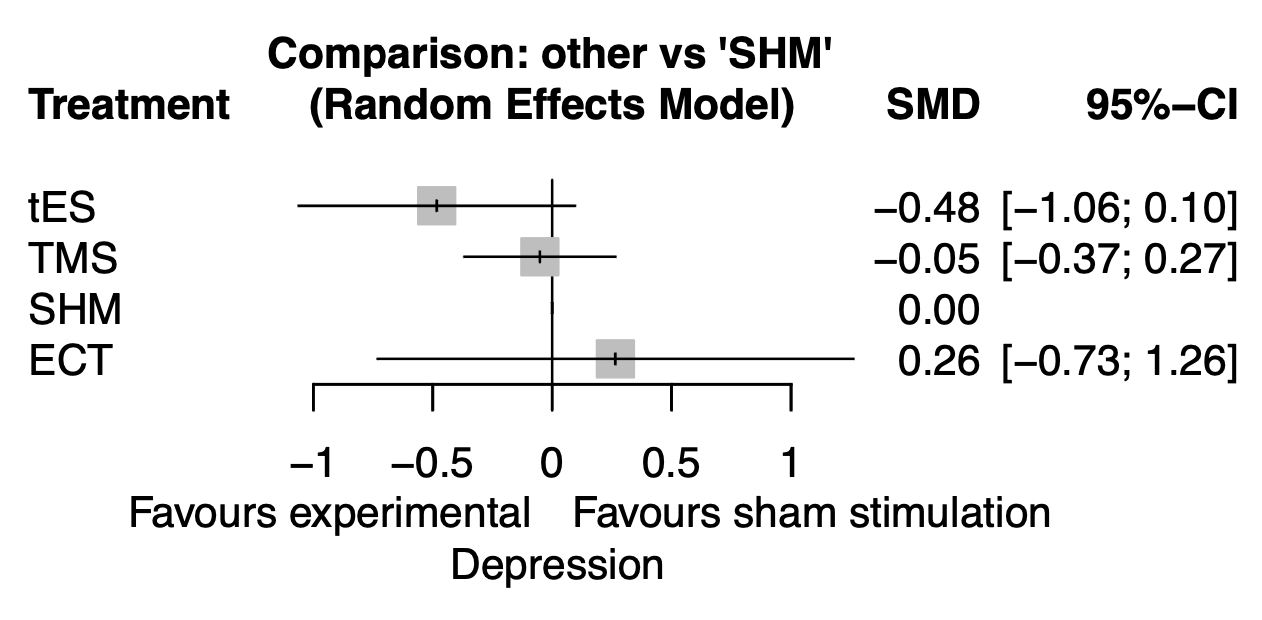


Forest-plot of results of network-meta-analysis including studies from Chinese mainland.

Quantifying heterogeneity / inconsistency:

tau^2 = 0.1431; tau = 0.3783; I^2 = 57.3% [18.7%; 77.5%]


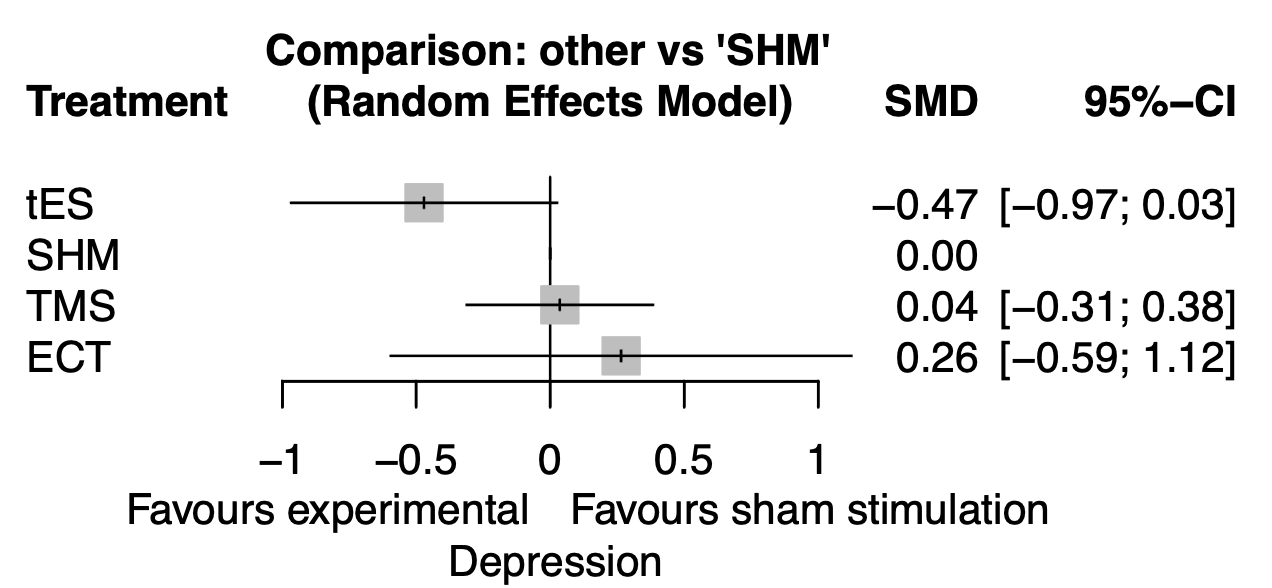


Forest-plot of results of network-meta-analysis excluding studies from Chinese mainland.

Quantifying heterogeneity / inconsistency:

tau^2 = 0.0769; tau = 0.2774; I^2 = 36.8% [0.0%; 70.9%]

| tES | . | -0.48 (-1.06,0.10) | . |
| --- | --- | --- | --- |
| -0.43 (-1.09,0.23) | TMS | -0.05 (-0.37,0.27) | . |
| -0.48 (-1.06,0.10) | -0.05 (-0.37,0.27) | SHM | -0.26 (-1.26,0.73) |
| -0.75 (-1.90,0.40) | -0.32 (-1.36,0.73) | -0.26 (-1.26,0.73) | ECT |

League-table of results of the network meta-analysis including studies from Chinese mainland.

| tES | -0.47 (-0.97,0.03) | . | . |
| --- | --- | --- | --- |
| -0.47 (-0.97,0.03) | SHM | -0.04 (-0.38,0.31) | -0.26 (-1.12,0.59) |
| -0.51 (-1.11,0.10) | -0.04 (-0.38,0.31) | TMS | . |
| -0.74 (-1.73,0.26) | -0.26 (-1.12,0.59) | -0.23 (-1.16,0.70) | ECT |

League-table of results of the network meta-analysis excluding studies from Chinese mainland.


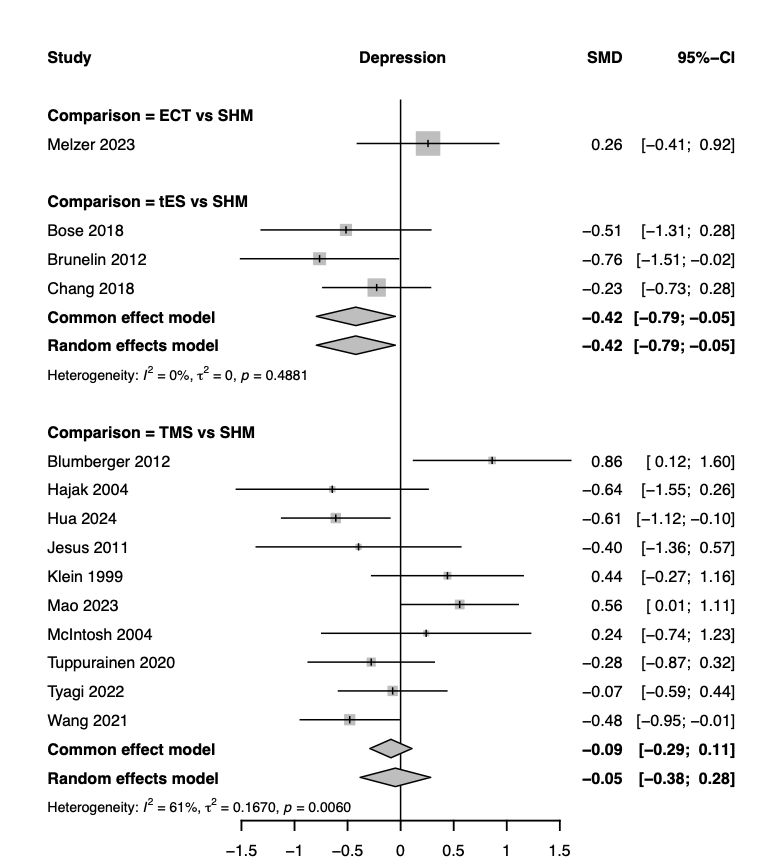


Forest-plot of results of pairwise meta-analyses including studies from Chinese mainland.


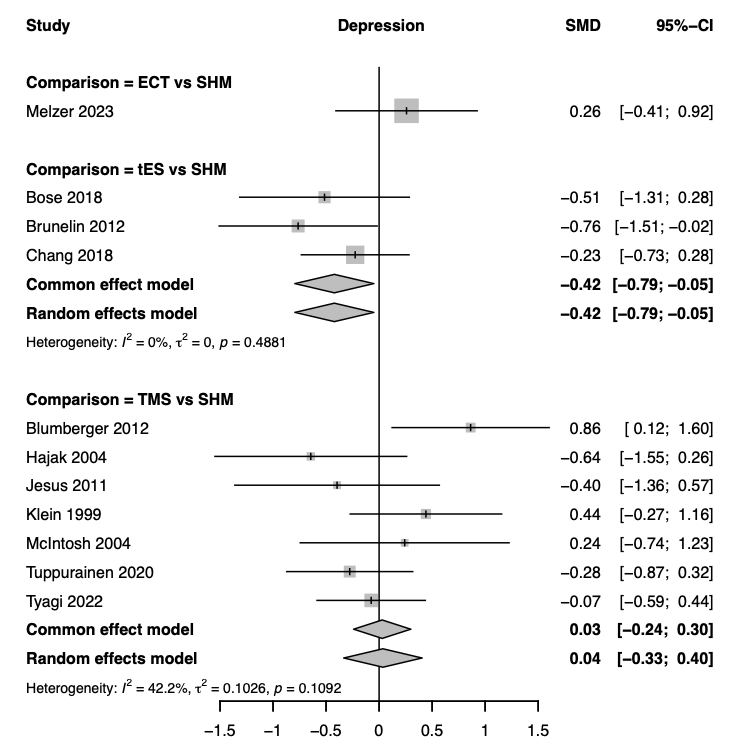


Forest-plot of results of pairwise meta-analyses excluding studies from Chinese mainland.

## **9.5 Functioning**


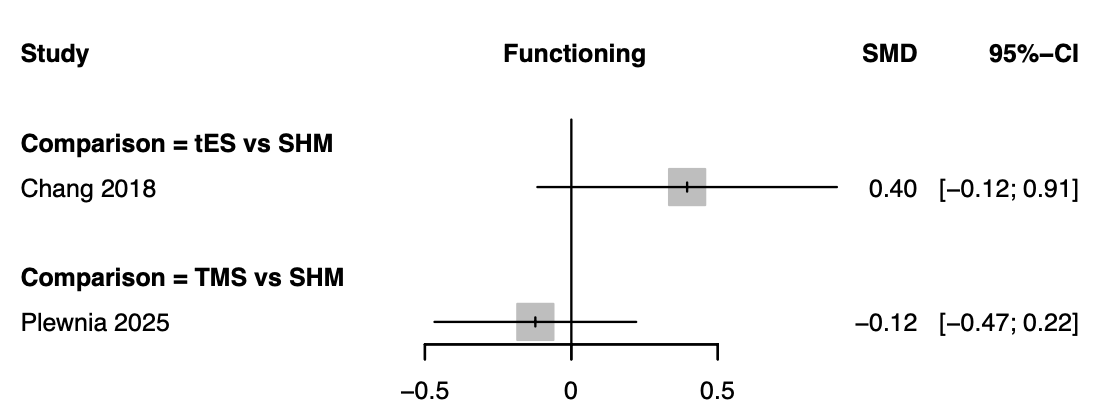


Due to the limited number of included studies, the findings presented here should be interpreted with caution.

## **9.6 Quality of life**


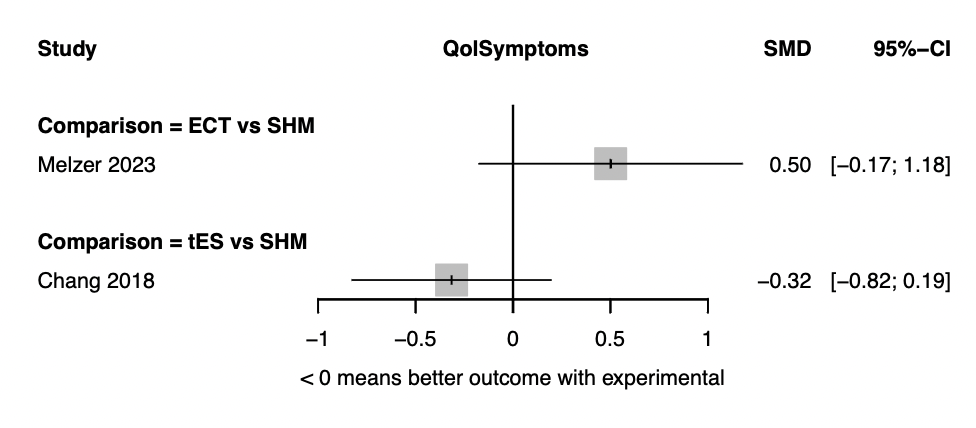


Due to the limited number of included studies, the findings presented here should be interpreted with caution.

##
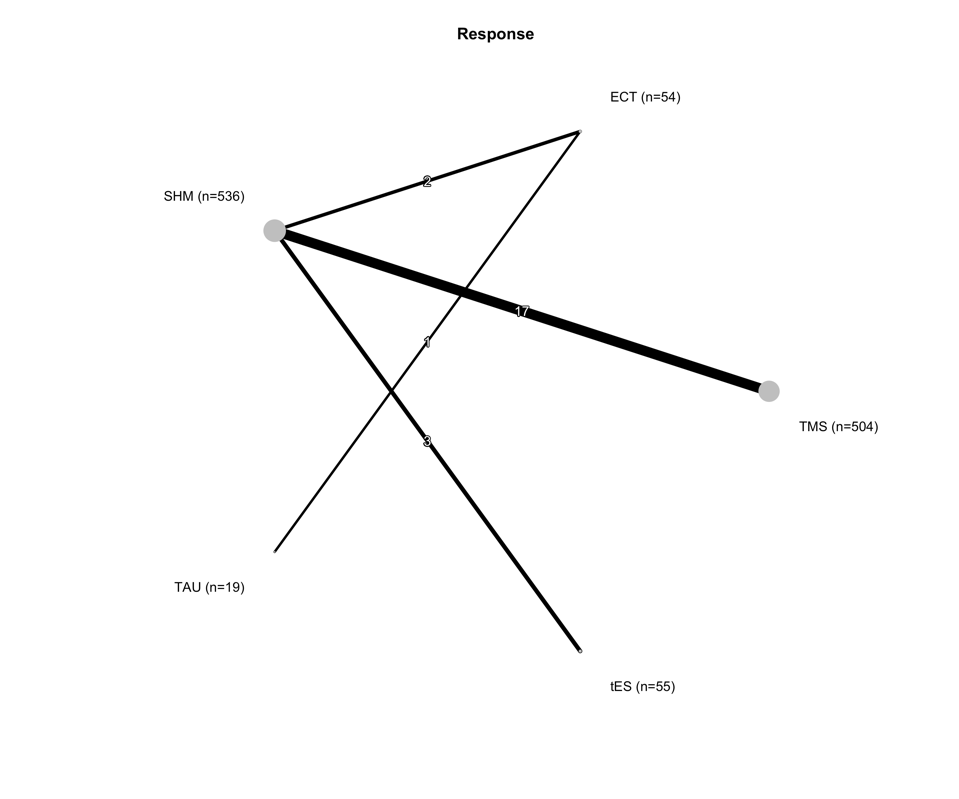
**9.7 Response**

Network plot including studies from Chinese mainland.


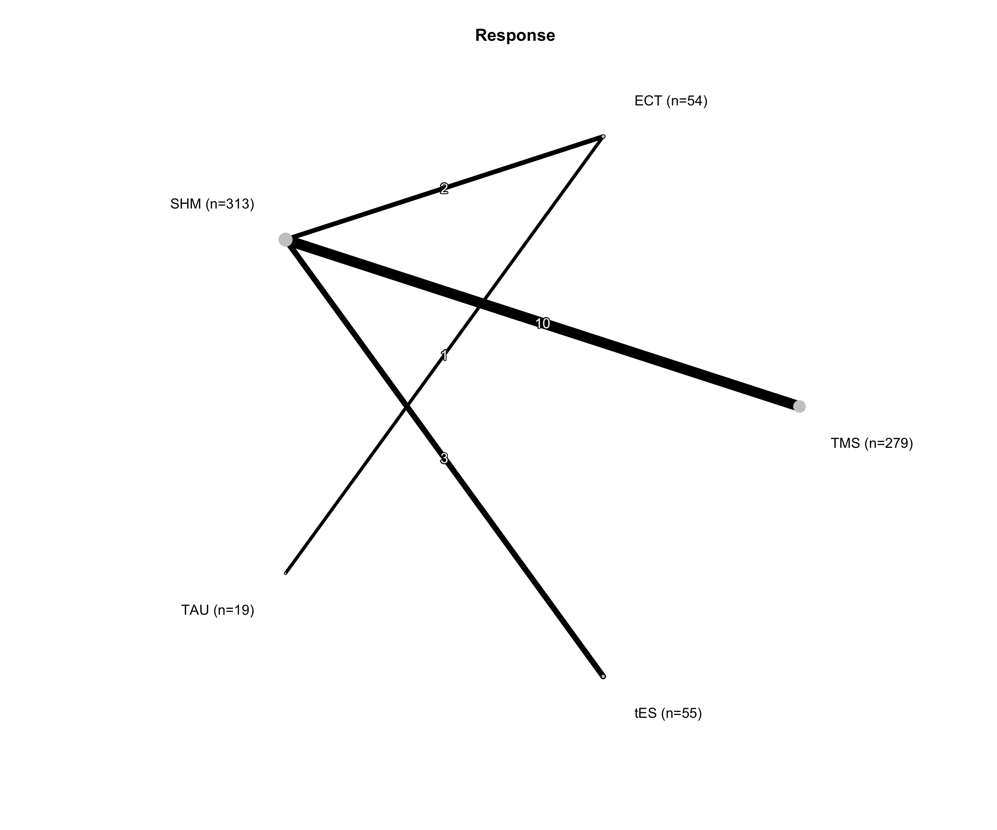


Network plot excluding studies from Chinese mainland.


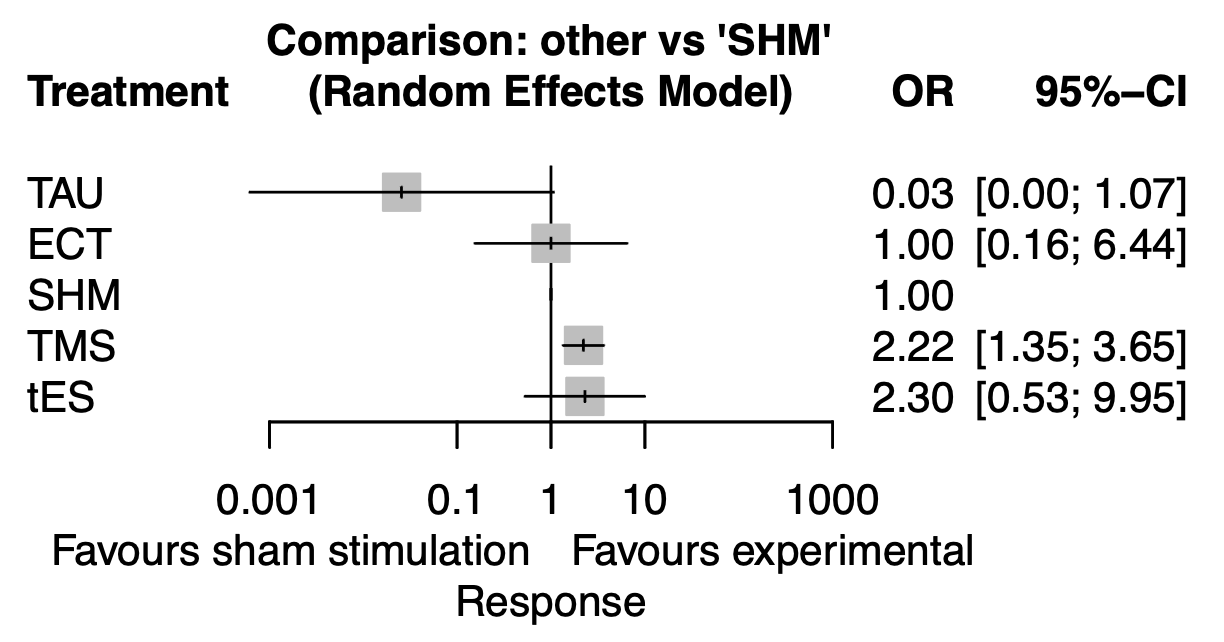


Forest-plot of results of network-meta-analysis including studies from Chinese mainland.

Quantifying heterogeneity / inconsistency:

tau^2 = 0.4751; tau = 0.6893; I^2 = 45.7% [8.3%; 67.9%]


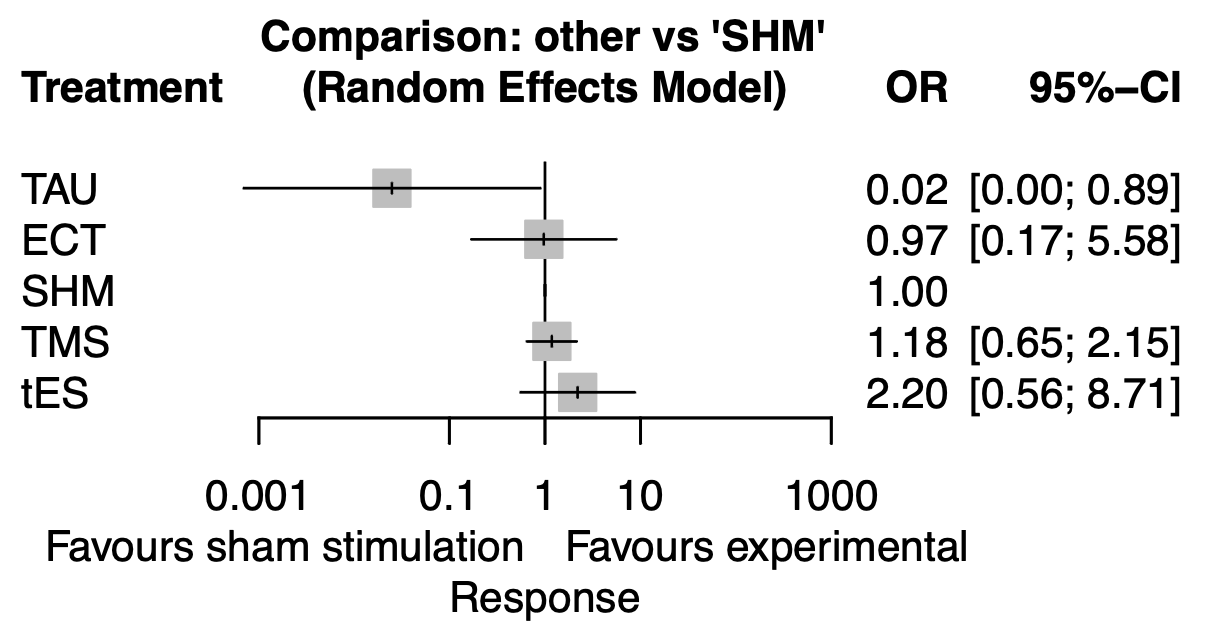


Forest-plot of results of network-meta-analysis excluding studies from Chinese mainland.

Quantifying heterogeneity / inconsistency:

tau^2 = 0.2963; tau = 0.5443; I^2 = 32% [0.0%; 64.8%]

| TAU | 0.03 (0.00, 0.65) | **.** | . | . |
| --- | --- | --- | --- | --- |
| 0.03 (0.00, 0.65) | ECT | 1.00 (0.16, 6.44) | . | . |
| 0.03 (0.00, 1.07) | 1.00 (0.16, 6.44) | SHM | 0.45 (0.27, 0.74) | 0.43 (0.10, 1.88) |
| 0.01 (0.00, 0.50) | 0.45 (0.07, 3.09) | 0.45 (0.27, 0.74) | TMS | . |
| 0.01 (0.00, 0.61) | 0.43 (0.04, 4.65) | 0.43 (0.10, 1.88) | 0.97 (0.21, 4.54) | tES |

League-table of results of the network meta-analysis including studies from Chinese mainland.

| TAU | 0.03 (0.00, 0.58) | **.** | . | . |
| --- | --- | --- | --- | --- |
| 0.03 (0.00, 0.58) | ECT | 0.97 (0.17, 5.58) | . | . |
| 0.02 (0.00, 0.89) | 0.97 (0.17, 5.58) | SHM | 0.85 (0.47, 1.54) | 0.45 (0.11, 1.80) |
| 0.02 (0.00, 0.79) | 0.82 (0.13, 5.23) | 0.85 (0.47, 1.54) | TMS | . |
| 0.01 (0.00, 0.52) | 0.44 (0.05, 4.09) | 0.45 (0.11, 1.80) | 0.54 (0.12, 2.40) | tES |

League-table of results of the network meta-analysis excluding studies from Chinese mainland.


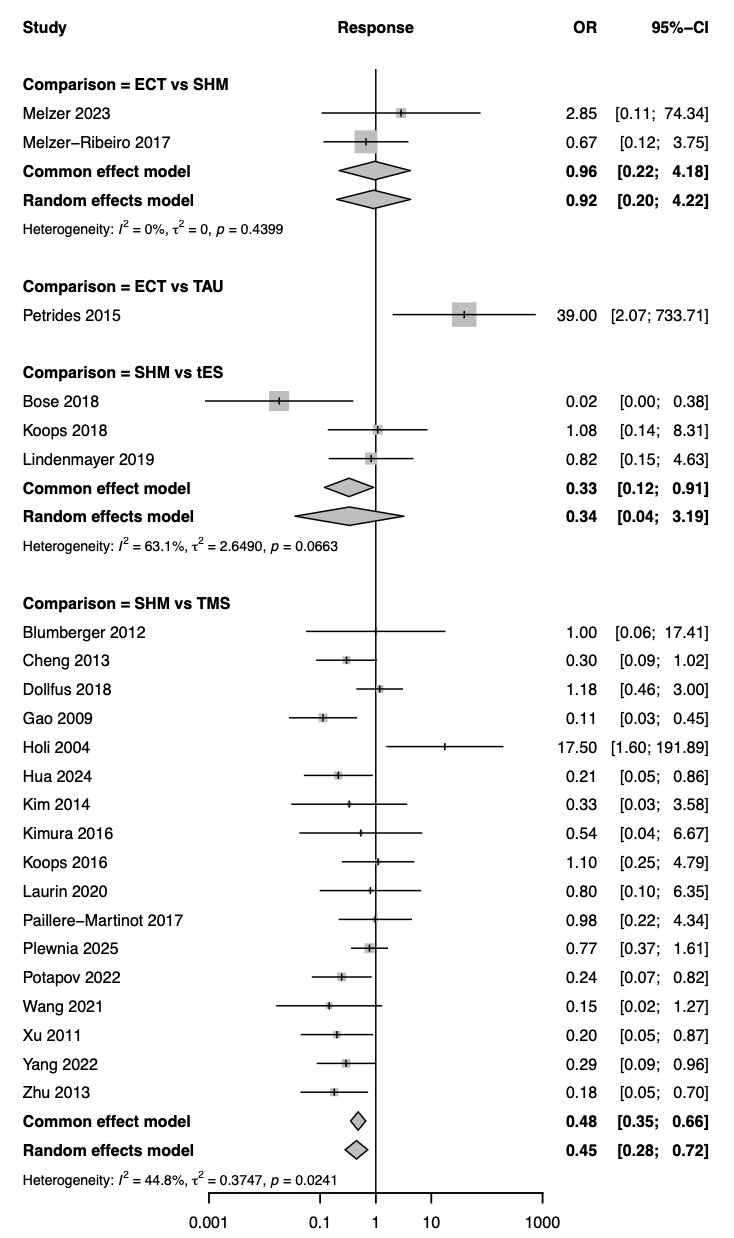


Forest-plot of results of pairwise meta-analyses including studies from Chinese mainland.


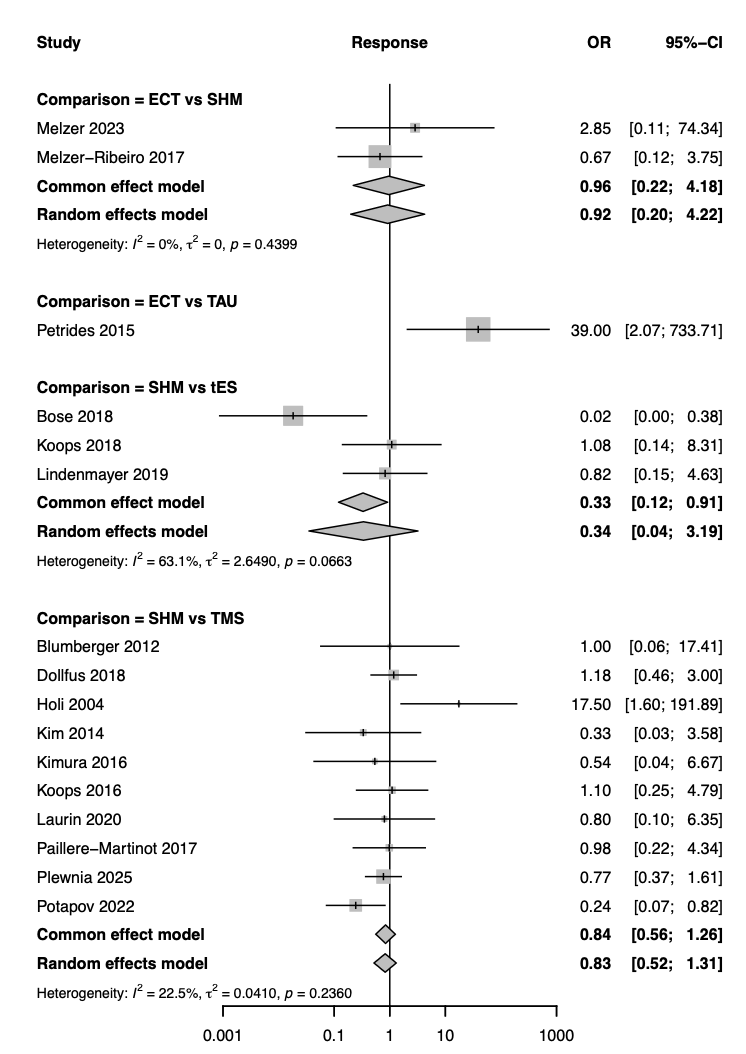


Forest-plot of results of pairwise meta-analyses excluding studies from Chinese mainland.

##
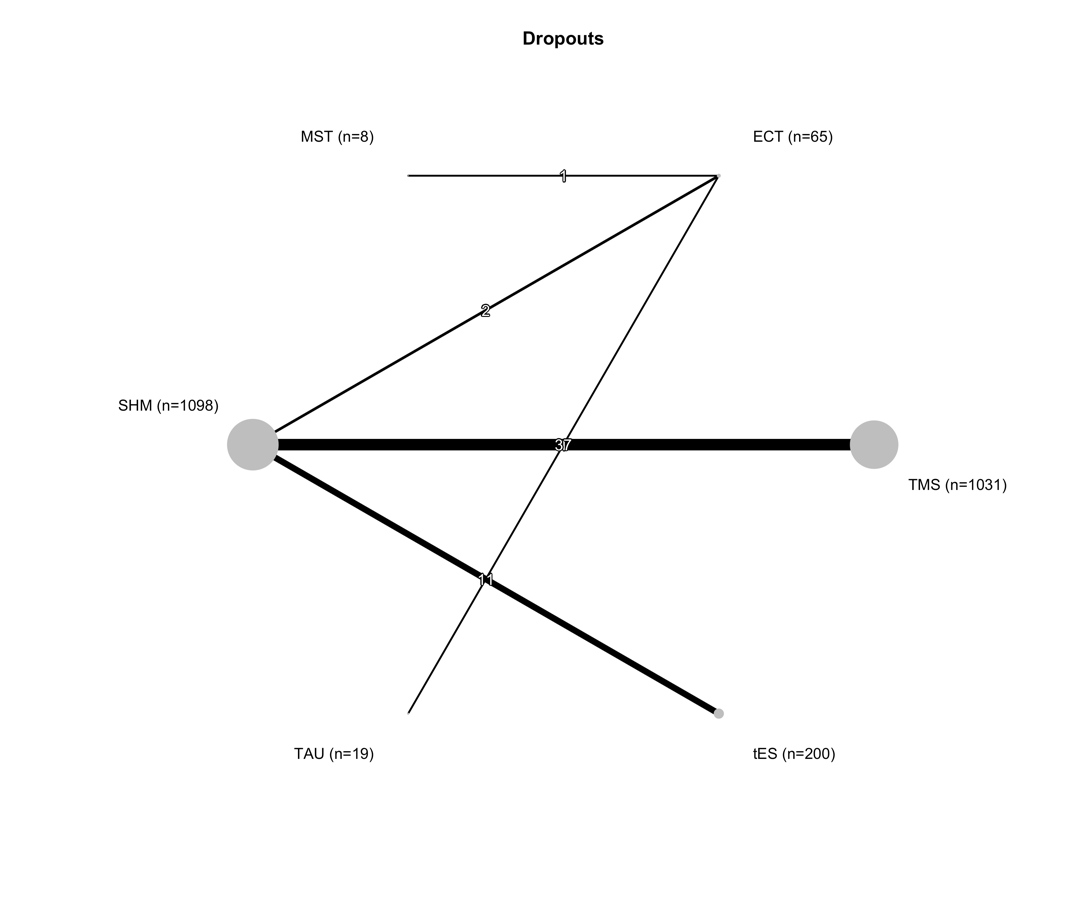
**9.8 Dropouts**

Network plot including studies from Chinese mainland.


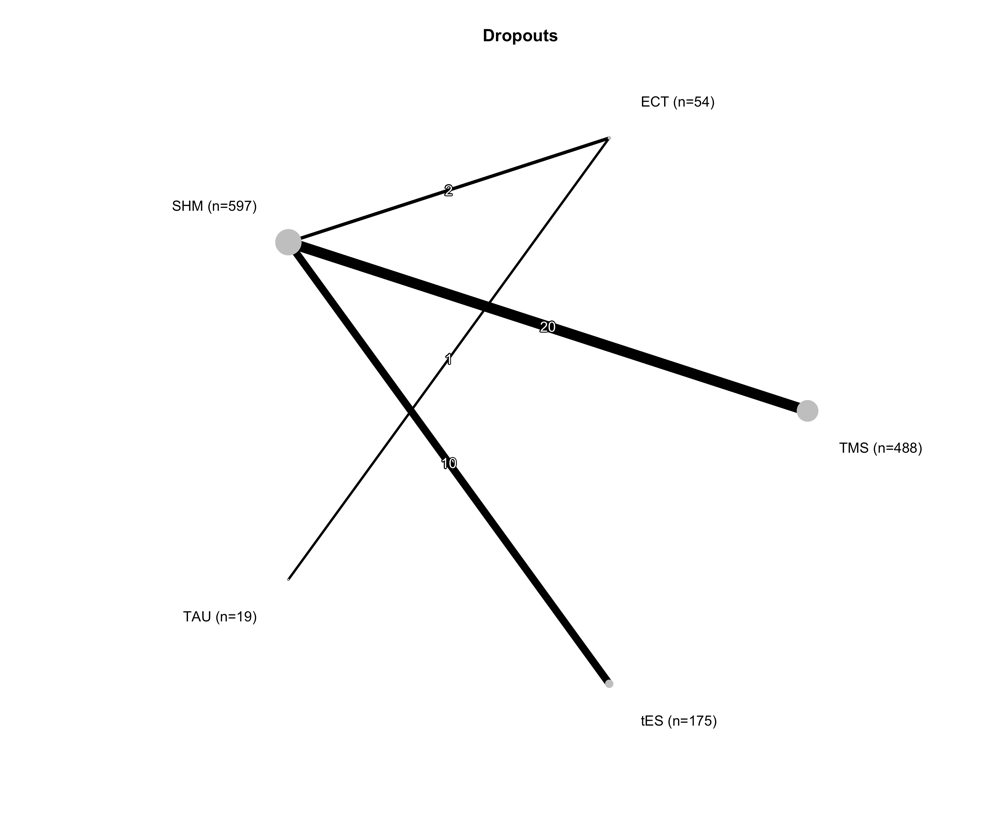


Network plot excluding studies from Chinese mainland.


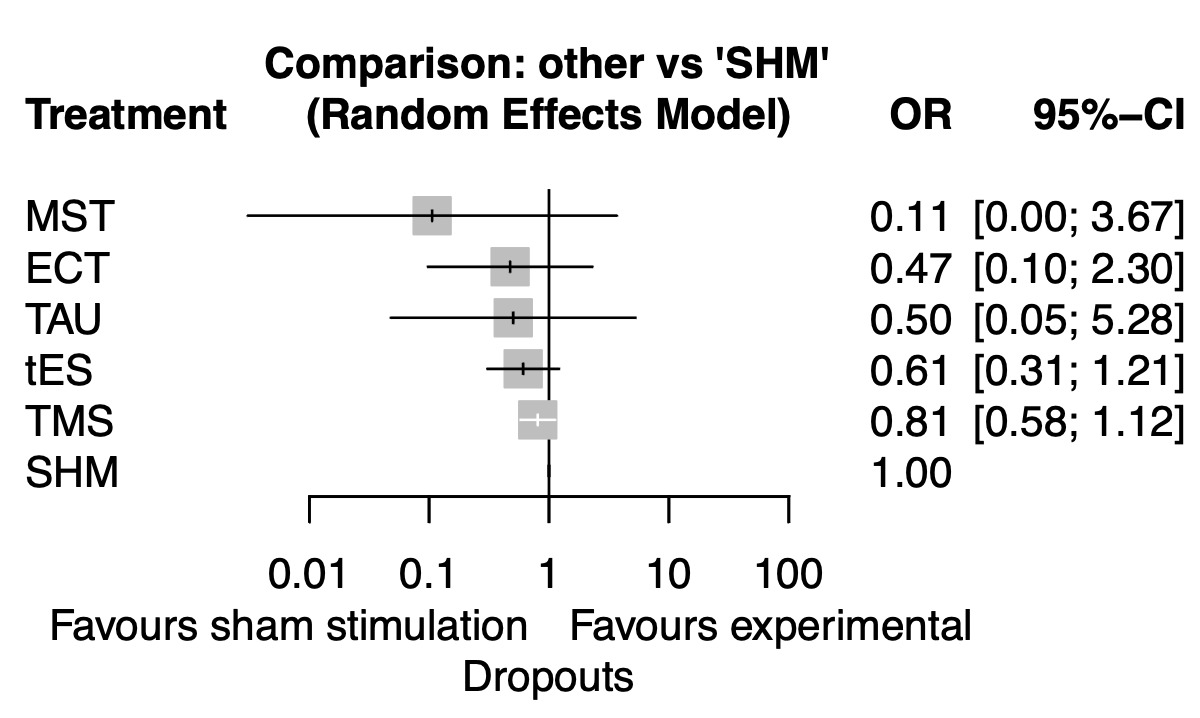


Forest-plot of results of network-meta-analysis including studies from Chinese mainland.

Quantifying heterogeneity / inconsistency:

tau^2 = 0; tau = 0; I^2 = 0% [0.0%; 33.5%]


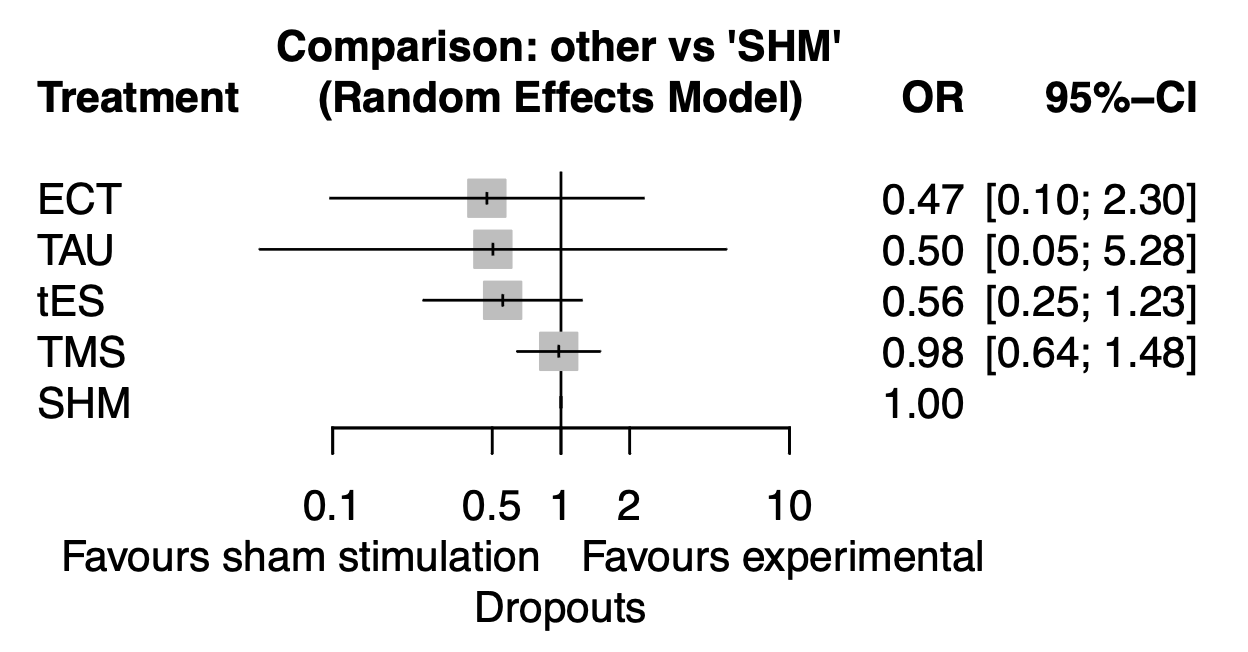


Forest-plot of results of network-meta-analysis excluding studies from Chinese mainland.

Quantifying heterogeneity / inconsistency:

tau^2 = 0; tau = 0; I^2 = 0% [0.0%; 40.8%]

| MST | 0.22 (0.01, 5.34) | . | . | . | . |
| --- | --- | --- | --- | --- | --- |
| 0.22 (0.01, 5.34) | ECT | 0.94 (0.17, 5.36) | . | . | 0.47 (0.10, 2.30) |
| 0.21 (0.01, 7.85) | 0.94 (0.17, 5.36) | TAU | . | . | . |
| 0.17 (0.00, 6.43) | 0.78 (0.14, 4.35) | 0.83 (0.07, 9.55) | tES | . | 0.61 (0.31, 1.21) |
| 0.13 (0.00, 4.62) | 0.59 (0.12, 2.95) | 0.62 (0.06, 6.70) | 0.76 (0.35, 1.62) | TMS | 0.81 (0.58, 1.12) |
| 0.11 (0.00, 3.67) | 0.47 (0.10, 2.30) | 0.50 (0.05, 5.28) | 0.61 (0.31, 1.21) | 0.81 (0.58, 1.12) | SHM |

League-table of results of the network meta-analysis including studies from Chinese mainland.

| ECT | 0.94 (0.17, 5.36) | . | . | 0.47 (0.10, 2.30) |
| --- | --- | --- | --- | --- |
| 0.94 (0.17, 5.36) | TAU | . | . | . |
| 0.85 (0.15, 5.00) | 0.91 (0.08,10.83) | tES | . | 0.56 (0.25, 1.23) |
| 0.49 (0.09, 2.48) | 0.52 (0.05, 5.60) | 0.57 (0.23, 1.40) | TMS | 0.98 (0.64, 1.48) |
| 0.47 (0.10, 2.30) | 0.50 (0.05, 5.28) | 0.56 (0.25, 1.23) | 0.98 (0.64, 1.48) | SHM |

League-table of results of the network meta-analysis excluding studies from Chinese mainland.


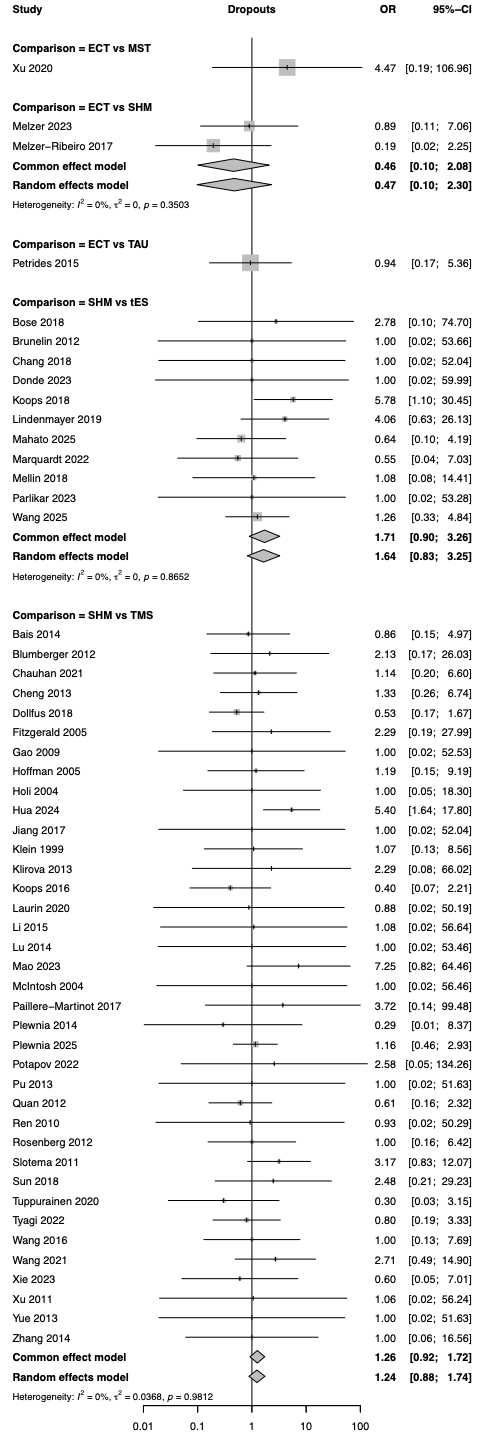


Forest-plot of results of pairwise meta-analyses including studies from Chinese mainland.


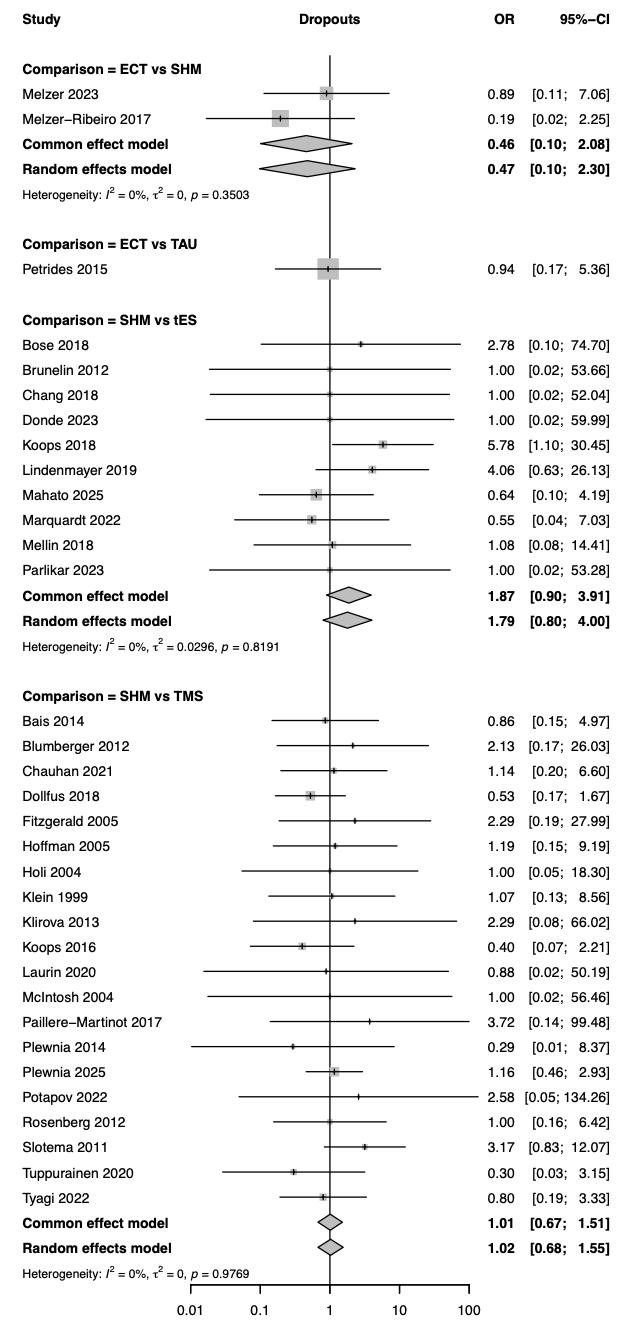


Forest-plot of results of pairwise meta-analyses excluding studies from Chinese mainland.

## **9.9 Cognition**

### **9.9.1 Attention vigilance**

**
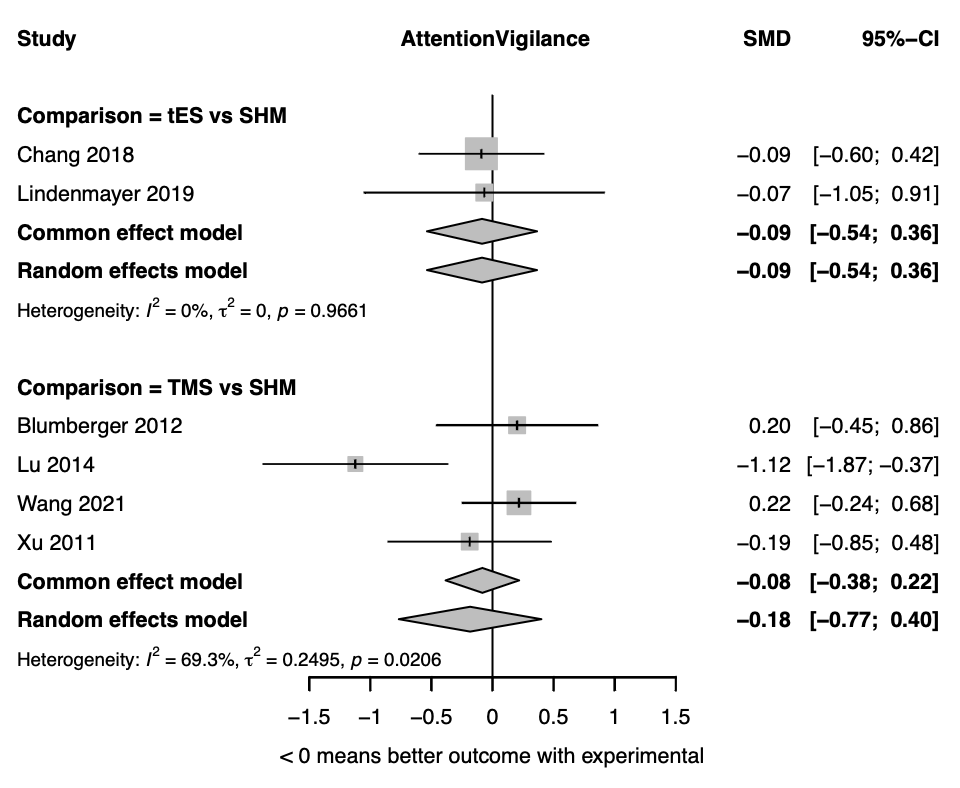
**

Forest-plot of results of pairwise meta-analyses including studies from Chinese mainland.


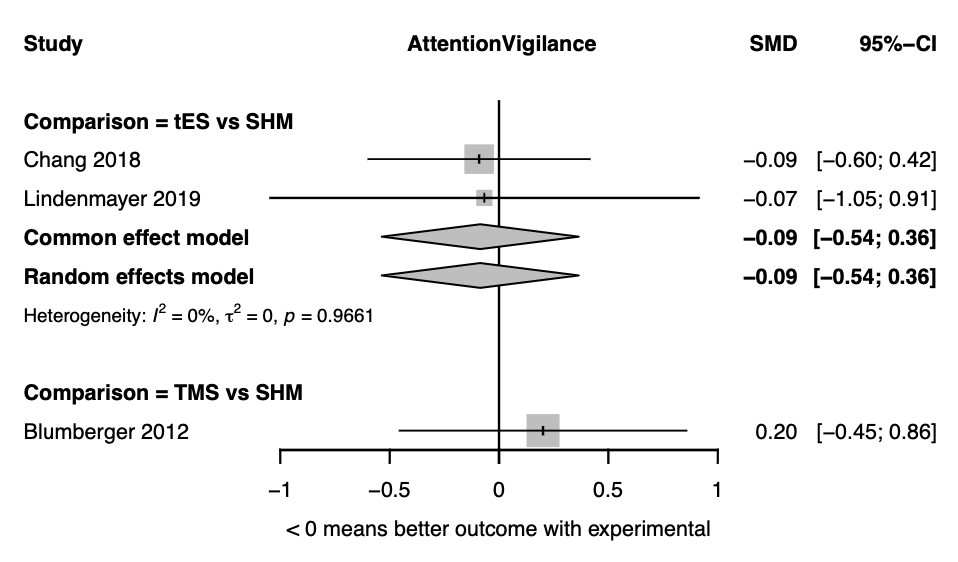


Forest-plot of results of pairwise meta-analyses excluding studies from Chinese mainland.

Due to the limited number of included studies, the findings presented here should be interpreted with caution.

### **9.9.2 Composite Cognition**


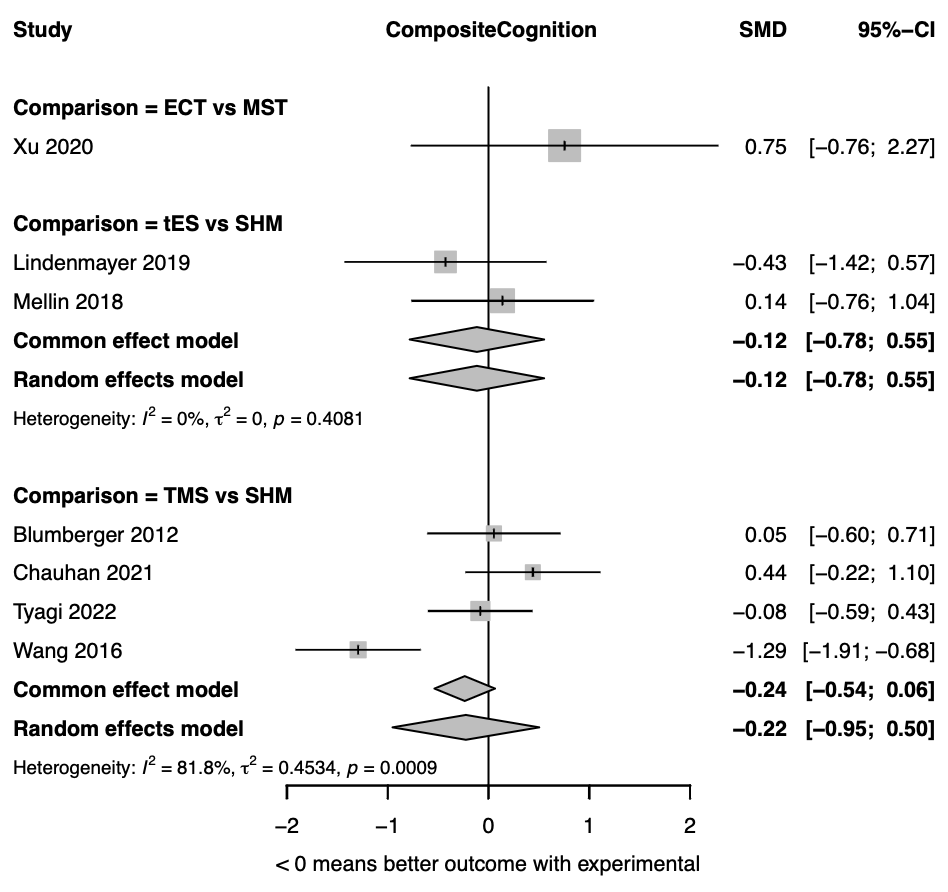


Forest-plot of results of pairwise meta-analyses including studies from Chinese mainland.


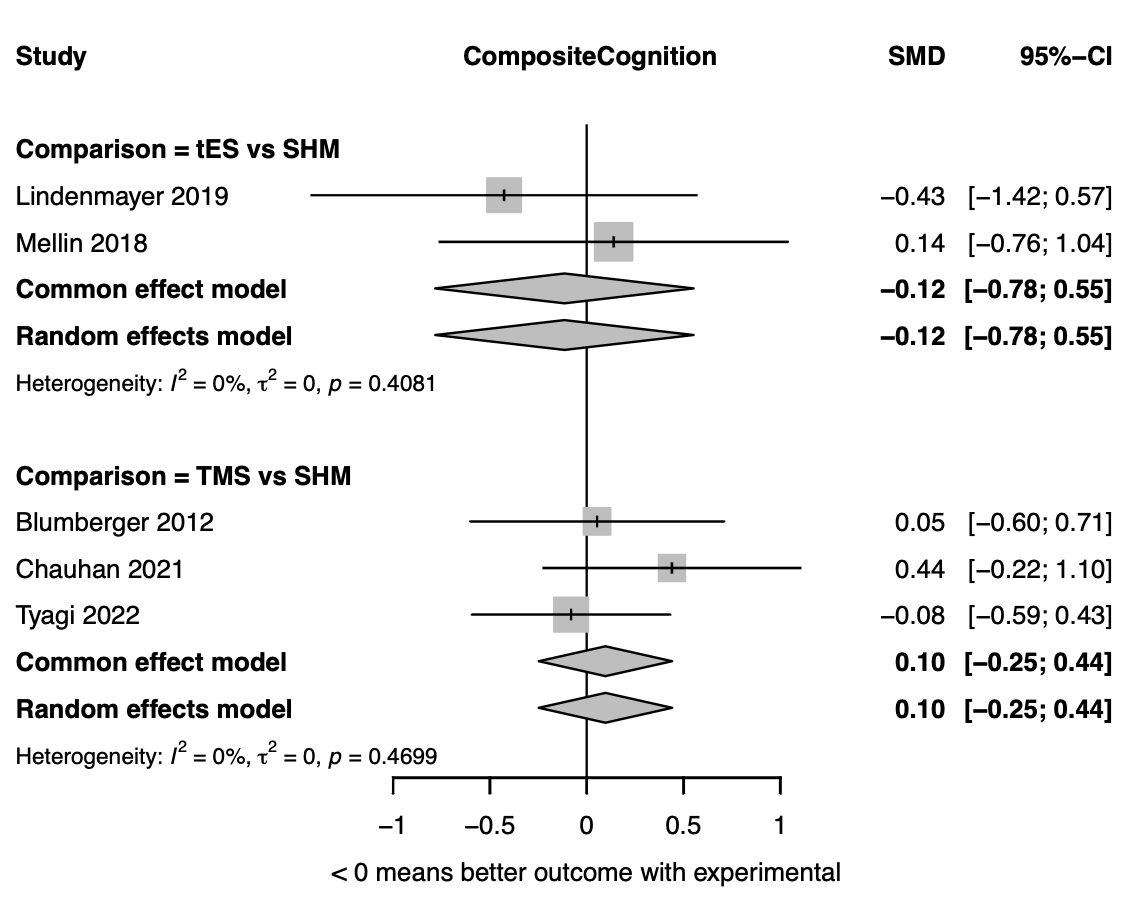


Forest-plot of results of pairwise meta-analyses excluding studies from Chinese mainland.

### **9.9.3 Reasoning and solving**


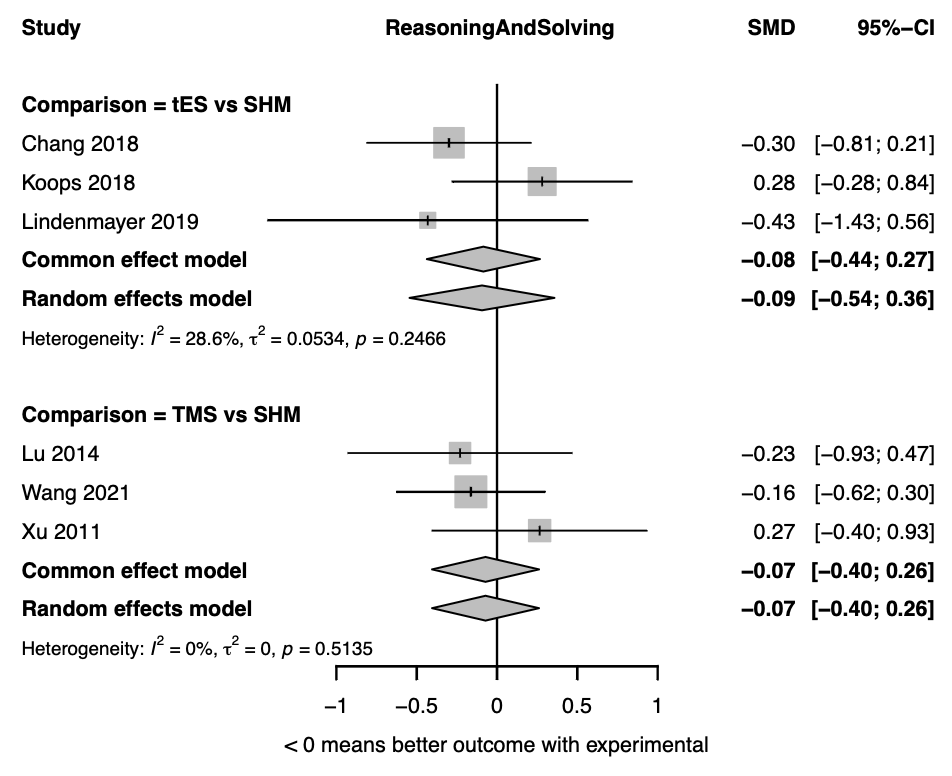


Forest-plot of results of pairwise meta-analyses including studies from Chinese mainland.


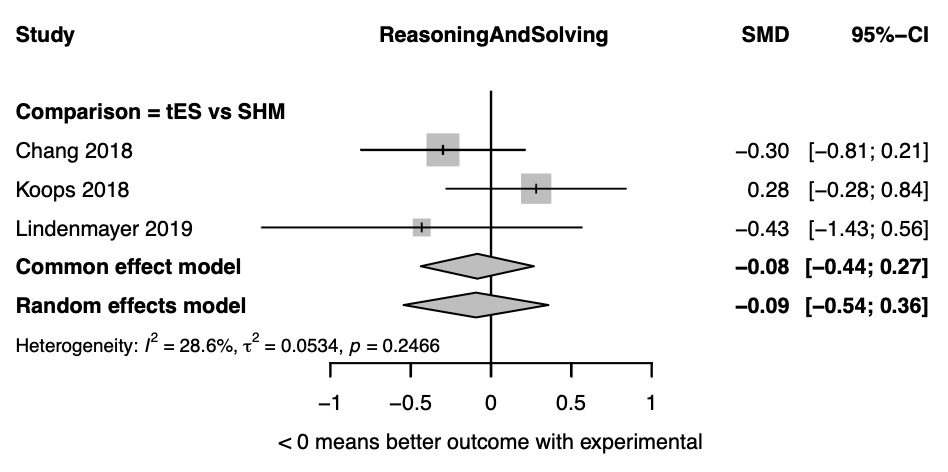


Forest-plot of results of pairwise meta-analyses excluding studies from Chinese mainland.

### **
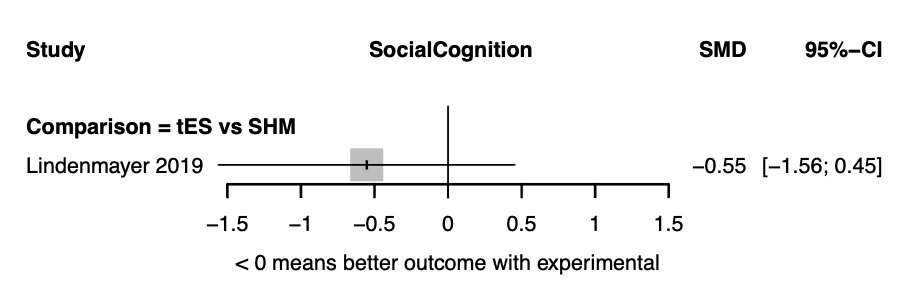
9.9.4 Social cognition**

Due to the limited number of included studies, the findings presented here should be interpreted with caution.

###
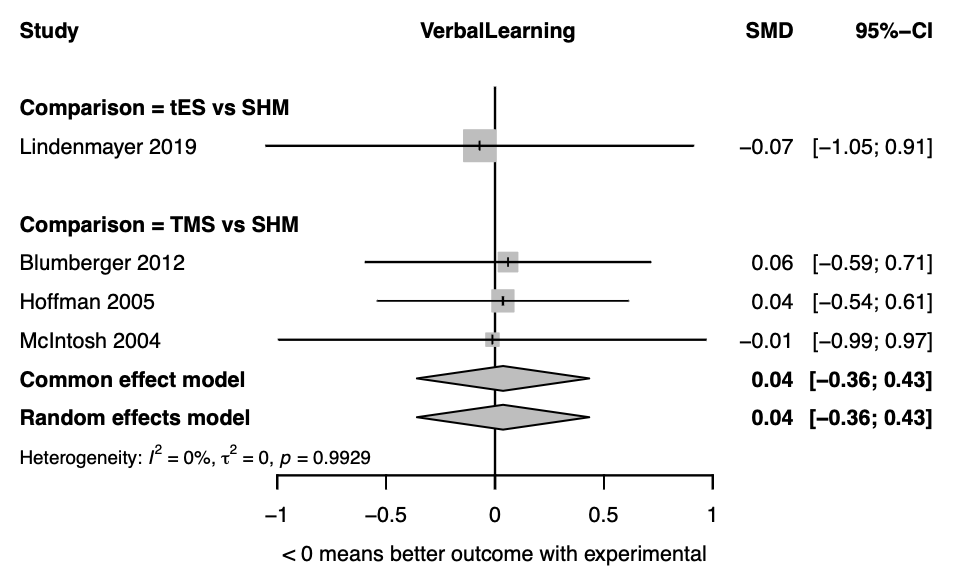
**9.9.5 Verbal learning**

Due to the limited number of included studies, the findings presented here should be interpreted with caution.

### **9.9.6 Visual learning**


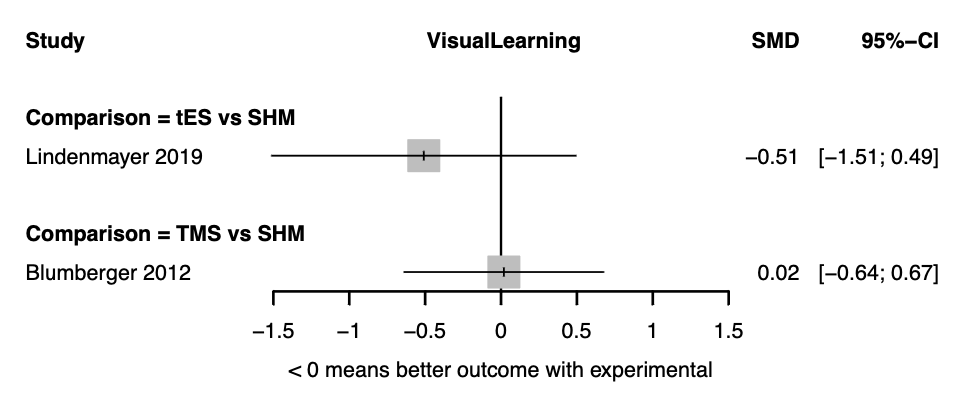


Due to the limited number of included studies, the findings presented here should be interpreted with caution.

### **9.9.7 Speed of processing**


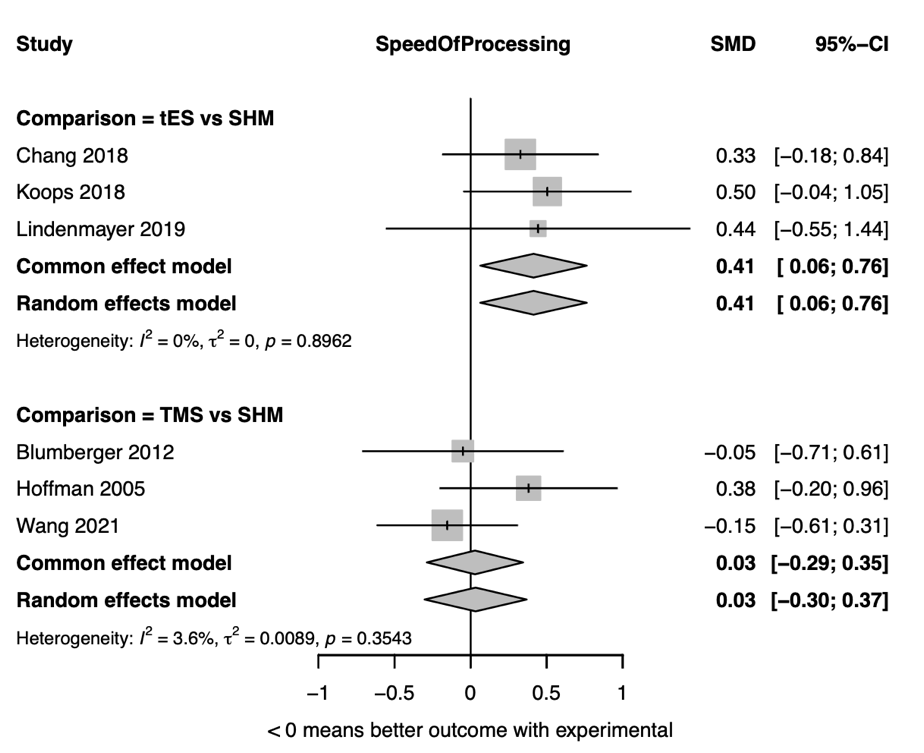
In the study by Koops 2018, there was a baseline imbalance between the two arms, which may have contributed to the observed effect. As a result, the pooled SMD could be influenced by this study and should be interpreted with caution.

Forest-plot of results of pairwise meta-analyses including studies from Chinese mainland.


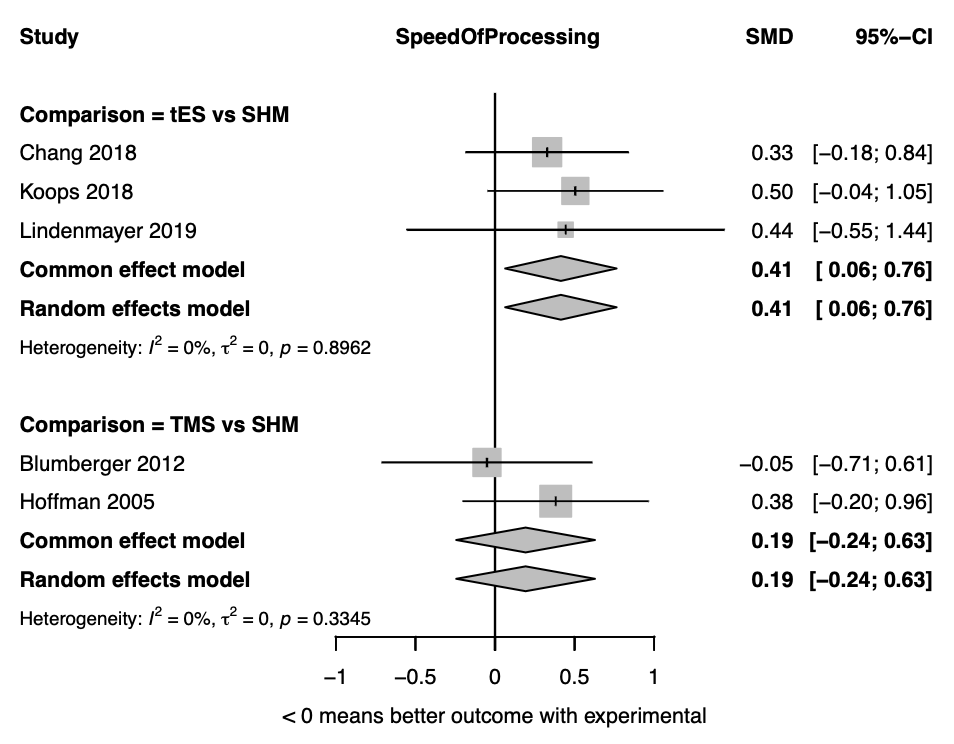


Forest-plot of results of pairwise meta-analyses excluding studies from Chinese mainland.

### **9.9.8 Working memory**

In the study by Lindenmayer 2019, there was a baseline imbalance between the two arms, which may have contributed to the observed effect. As a result, the pooled SMD could be influenced by this study and should be interpreted with caution.


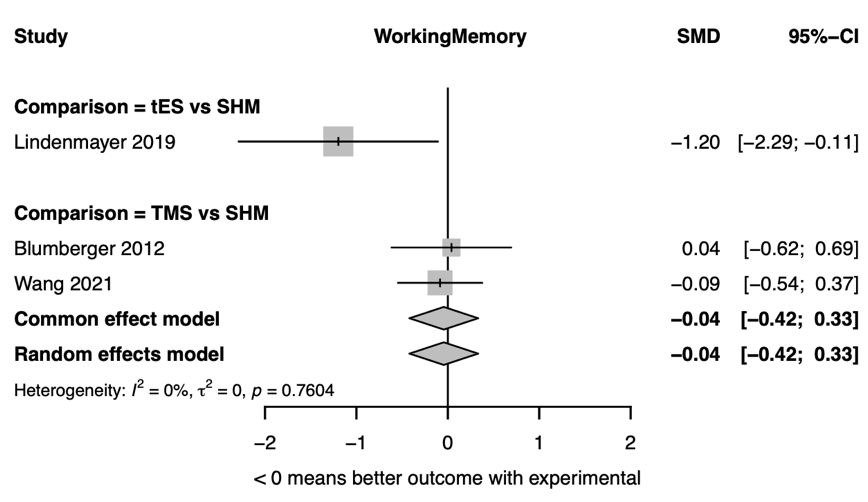


Forest-plot of results of pairwise meta-analyses including studies from Chinese mainland.


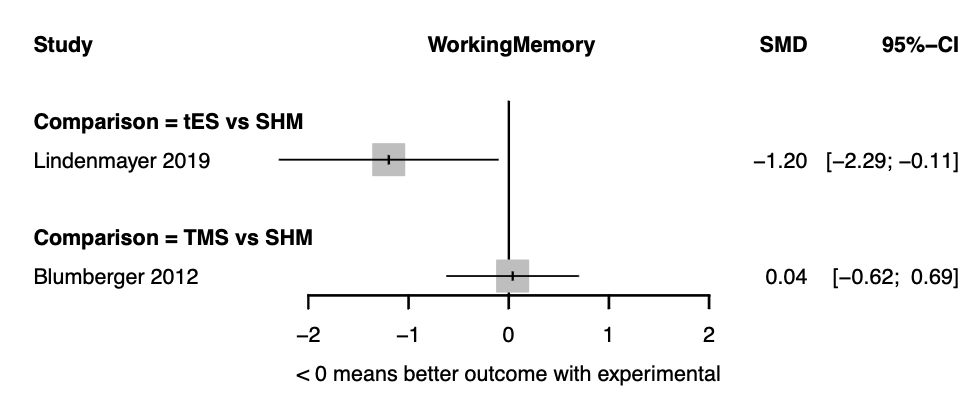


Forest-plot of results of pairwise meta-analyses excluding studies from Chinese mainland.

Due to the limited number of included studies, the findings presented here should be interpreted with caution.

## **9.10 Follow-up**

For follow-up, we extracted all available endpoint times, which were categorized into follow-ups at the 1st, 2nd, 3rd and 6th months. If a follow-up time occurred after an earlier endpoint (e.g., 1st month) but before the next (e.g., 2nd month), it was classified under the next endpoint. For example, if a study conducted a follow-up at 6 weeks, it was classified as the 2nd-month follow-up.

### **
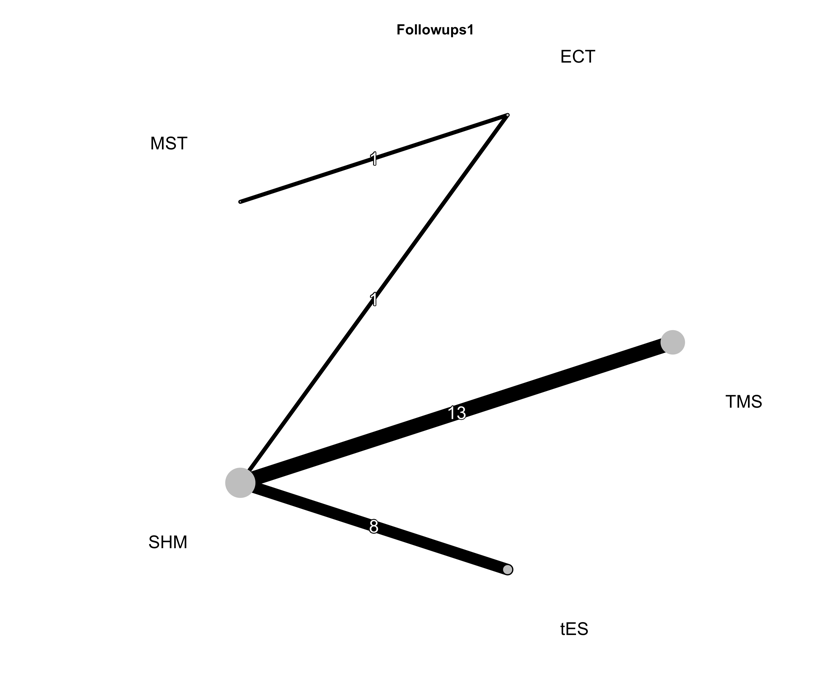
9.10.1 Follow-up in 1 month**

Network plot including studies from Chinese mainland.

**
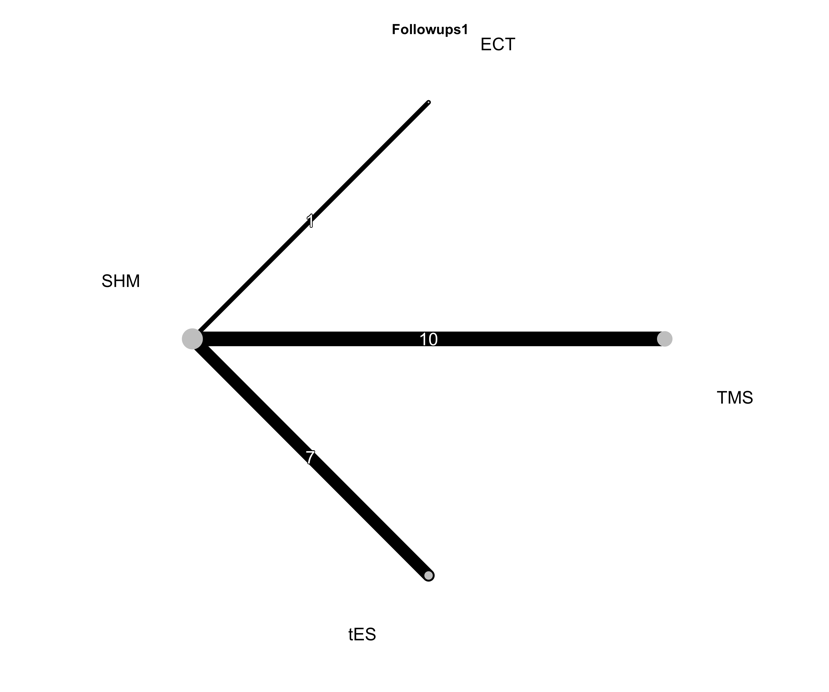
**

Network plot excluding studies from Chinese mainland.

**
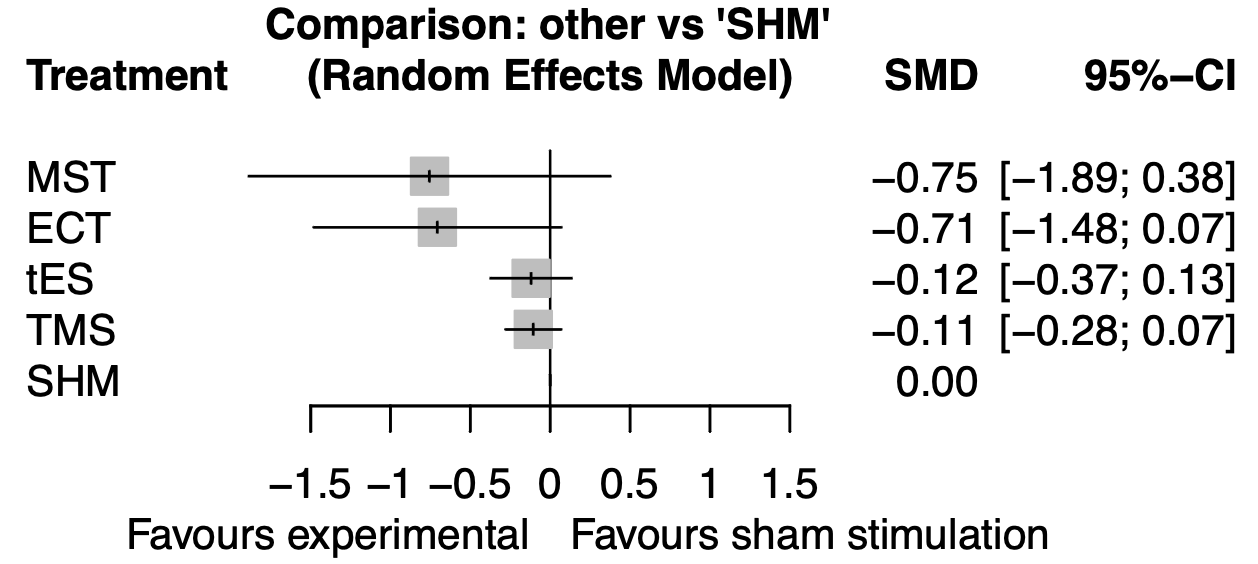
**

Forest-plot of results of network-meta-analysis including studies from Chinese mainland.

Quantifying heterogeneity / inconsistency:

tau^2 = 0.0513; tau = 0.2264; I^2 = 49.9% [16.0%; 70.1%]

**
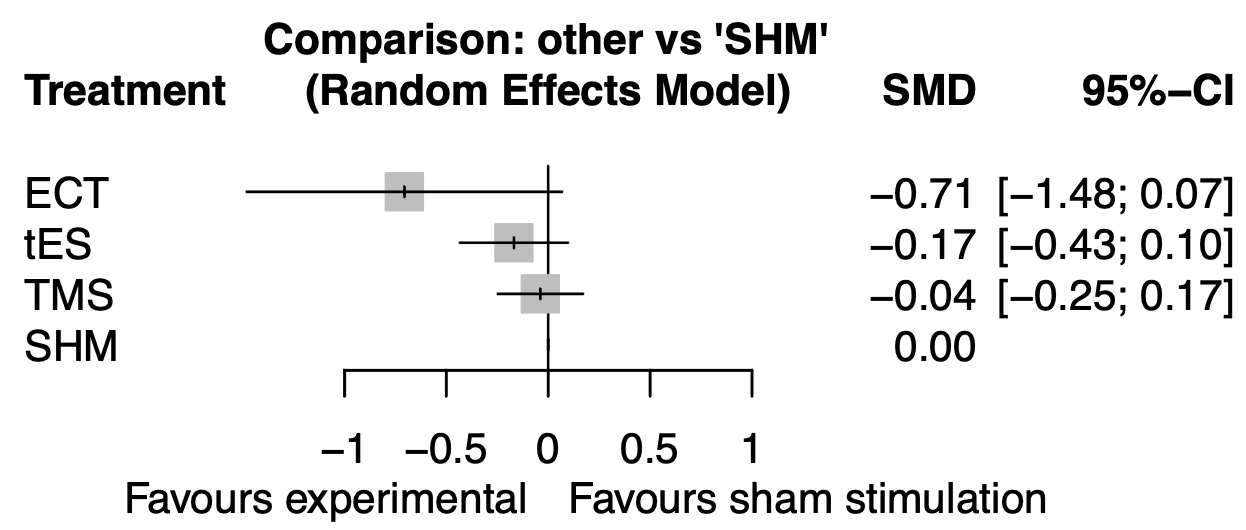
**

Forest-plot of results of network-meta-analysis excluding studies from Chinese mainland.

Quantifying heterogeneity / inconsistency:

tau^2 = 0.0501; tau = 0.2239; I^2 = 45% [1.3%; 69.4%]

| MST | -0.05 (-0.87,0.77) | . | . | . |
| --- | --- | --- | --- | --- |
| -0.05 (-0.87,0.77) | ECT | . | . | -0.71 (-1.48,0.07) |
| -0.64 (-1.79,0.52) | -0.59 (-1.40,0.23) | tES | . | -0.12 (-0.37,0.13) |
| -0.65 (-1.79,0.50) | -0.60 (-1.40,0.20) | -0.01 (-0.32,0.29) | TMS | -0.11 (-0.28,0.07) |
| -0.75 (-1.89,0.38) | -0.71 (-1.48,0.07) | -0.12 (-0.37,0.13) | -0.11 (-0.28,0.07) | SHM |

League-table of results of the network meta-analysis including studies from Chinese mainland.

| ECT | . | . | -0.71 (-1.48,0.07) |
| --- | --- | --- | --- |
| -0.54 (-1.35,0.28) | tES | . | -0.17 (-0.43,0.10) |
| -0.67 (-1.47,0.13) | -0.13 (-0.47,0.21) | TMS | -0.04 (-0.25,0.17) |
| -0.71 (-1.48,0.07) | -0.17 (-0.43,0.10) | -0.04 (-0.25,0.17) | SHM |

League-table of results of the network meta-analysis excluding studies from Chinese mainland.

**
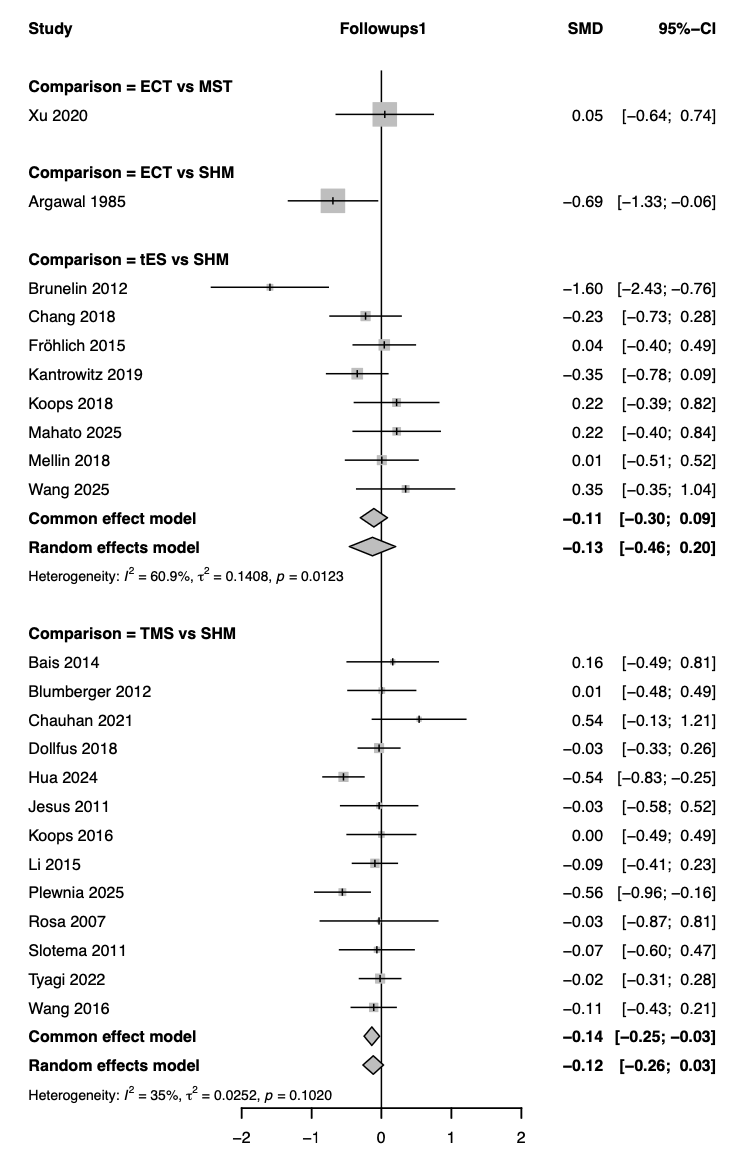
**

Forest-plot of results of pairwise meta-analyses including studies from Chinese mainland.

**
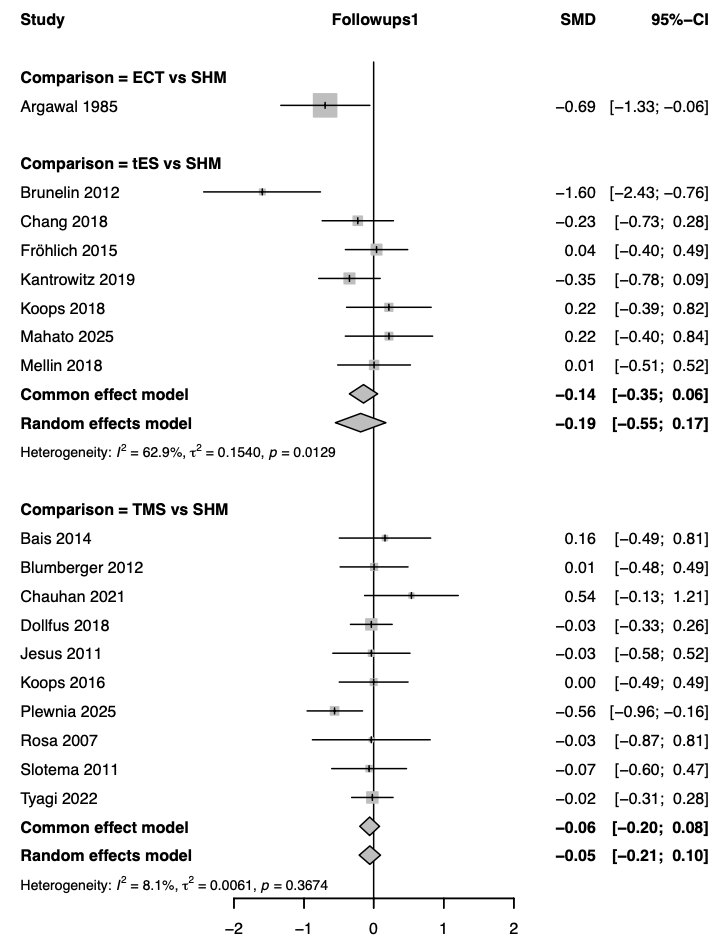
**

Forest-plot of results of pairwise meta-analyses excluding studies from Chinese mainland.

### **9.10.2 Follow-up in 2 months**

**
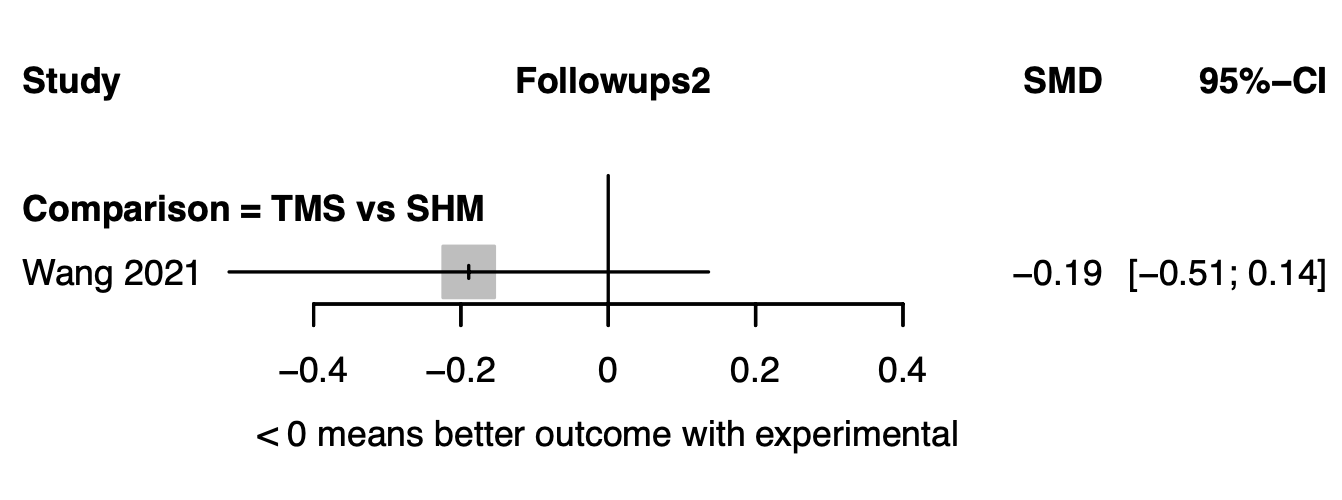
**

### **9.10.3 Follow-up in 3 months**


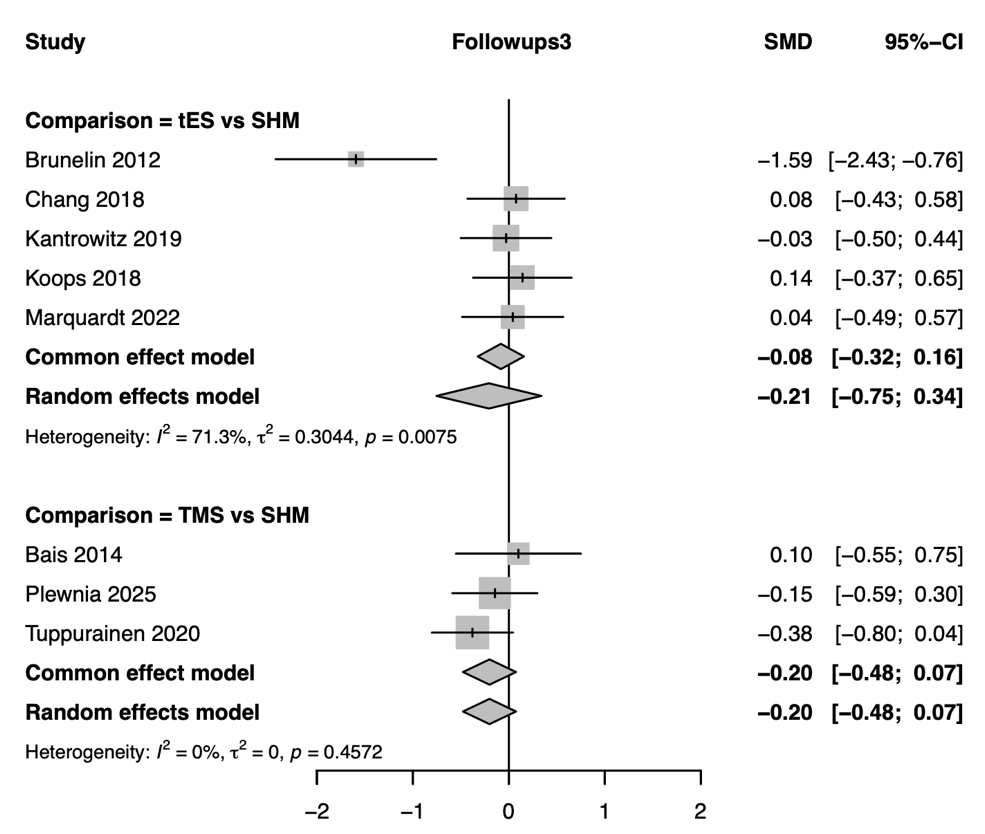


### **9.10.4 Follow-up in 6 months**


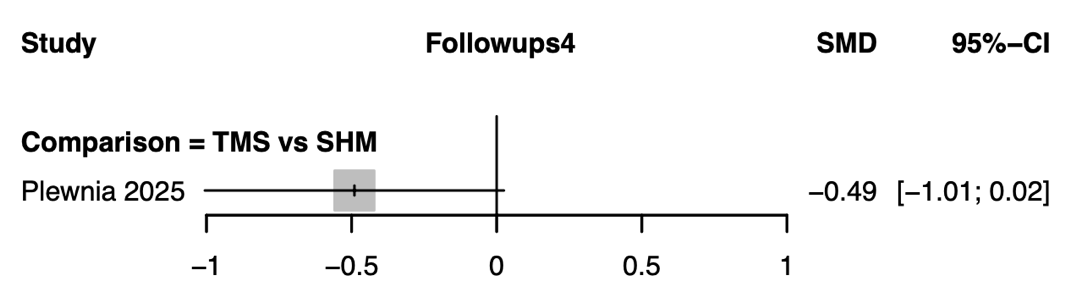


## **9.11 Side effects**

We assessed side effects which are known to occur with NIBS, including neurological, cognitive, cardiovascular, and musculoskeletal events. In the original studies, side effects were assessed by open interviews, not by specific rating scales. We analysed them based on whether each specific side effect occurred at least once during the trial, irrespective of severity. As with all dichotomous variables, we use odds ratios in the meta-analytic calculations.

### **
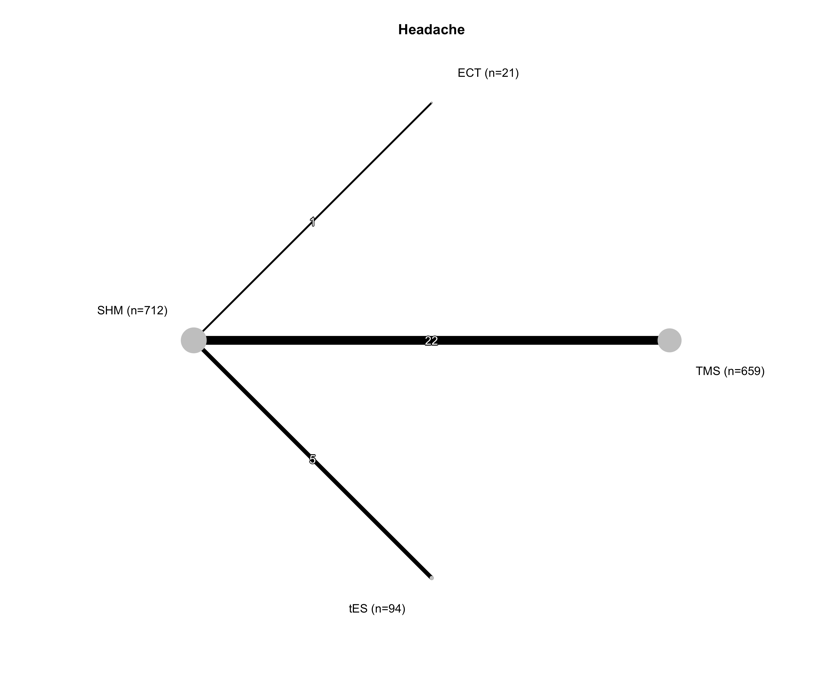
9.11.1 Headache**

Network plot including studies from Chinese mainland.

**
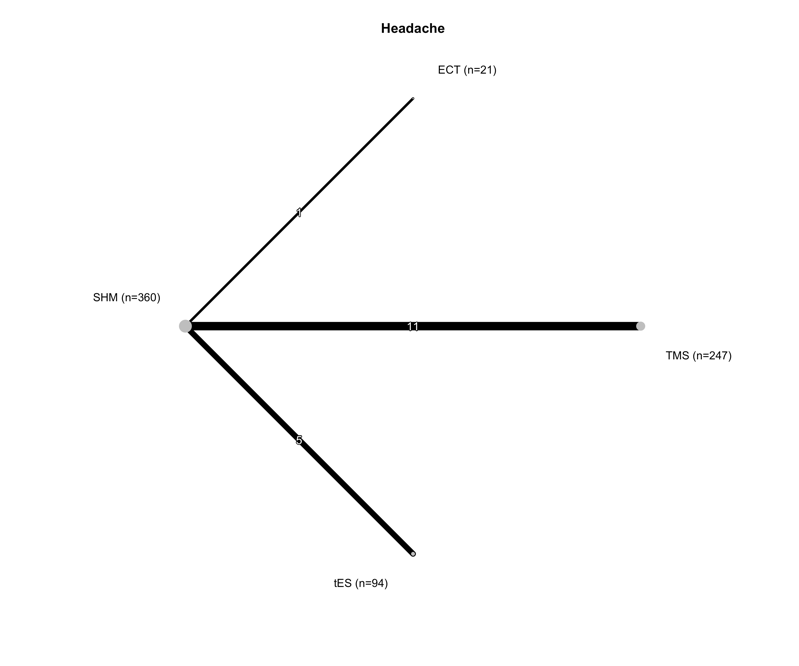
**

Network plot excluding studies from Chinese mainland.

**
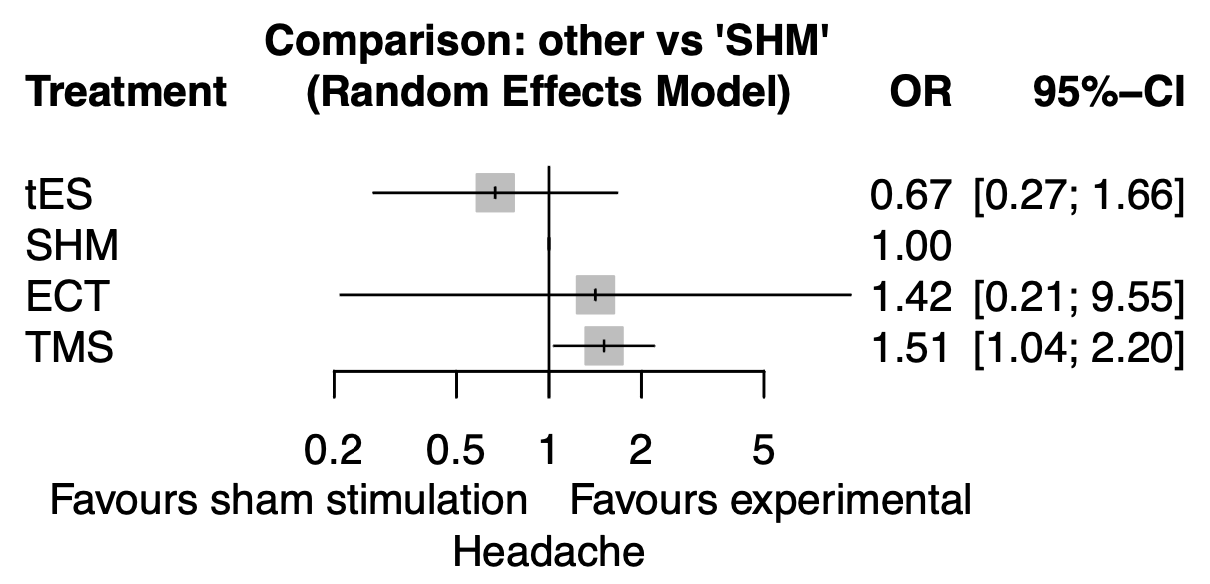
**

Forest-plot of results of network-meta-analysis including studies from Chinese mainland.

Quantifying heterogeneity / inconsistency:

tau^2 = 0; tau = 0; I^2 = 0% [0.0%; 43.2%]

**
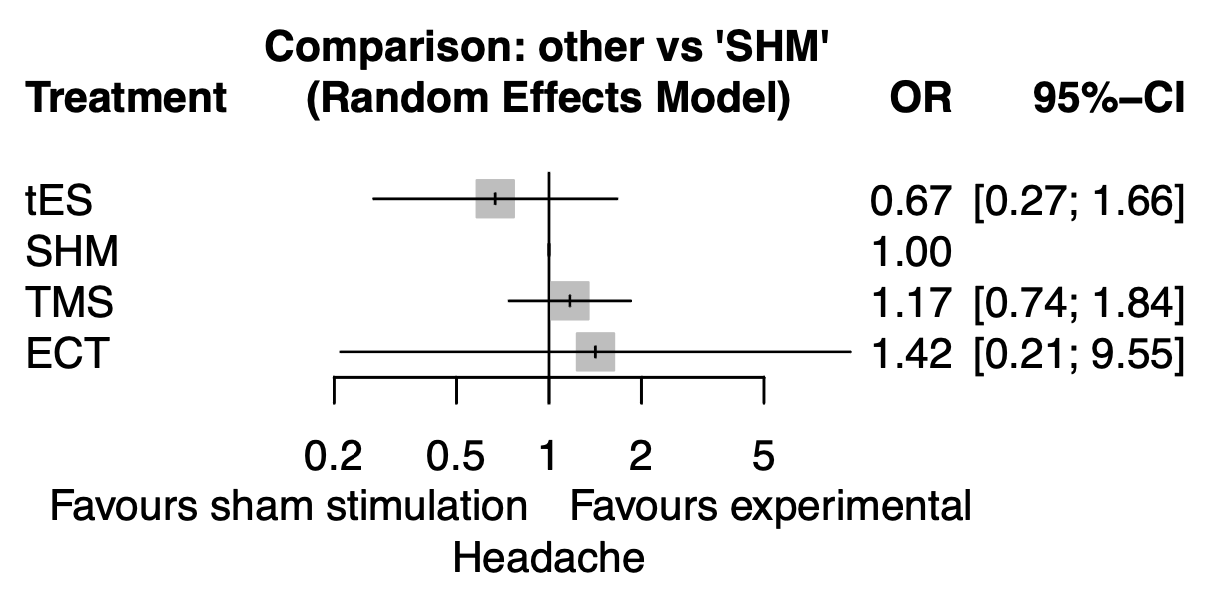
**

Forest-plot of results of network-meta-analysis e xcluding studies from Chinese mainland. Quantifying heterogeneity / inconsistency:

tau^2 = 0; tau = 0; I^2 = 0% [0.0%; 53.6%]

| tES | 0.67 (0.27,1.66) | . | . |
| --- | --- | --- | --- |
| 0.67 (0.27, 1.66) | SHM | 0.71 (0.10,4.76) | 0.66 (0.46,0.96) |
| 0.47 (0.06, 3.91) | 0.71 (0.10, 4.76) | ECT | . |
| 0.44 (0.17, 1.18) | 0.66 (0.46, 0.96) | 0.94 (0.13, 6.55) | TMS |

League-table of results of the network meta-analysis including studies from Chinese mainland.

| tES | 0.67 (0.27,1.66) | . | . |
| --- | --- | --- | --- |
| 0.67 (0.27, 1.66) | SHM | 0.85 (0.54,1.35) | 0.71 (0.10,4.76) |
| 0.57 (0.21, 1.58) | 0.85 (0.54, 1.35) | TMS | . |
| 0.47 (0.06, 3.91) | 0.71 (0.10, 4.76) | 0.83 (0.12, 5.87) | ECT |

League-table of results of the network meta-analysis excluding studies from Chinese mainland.


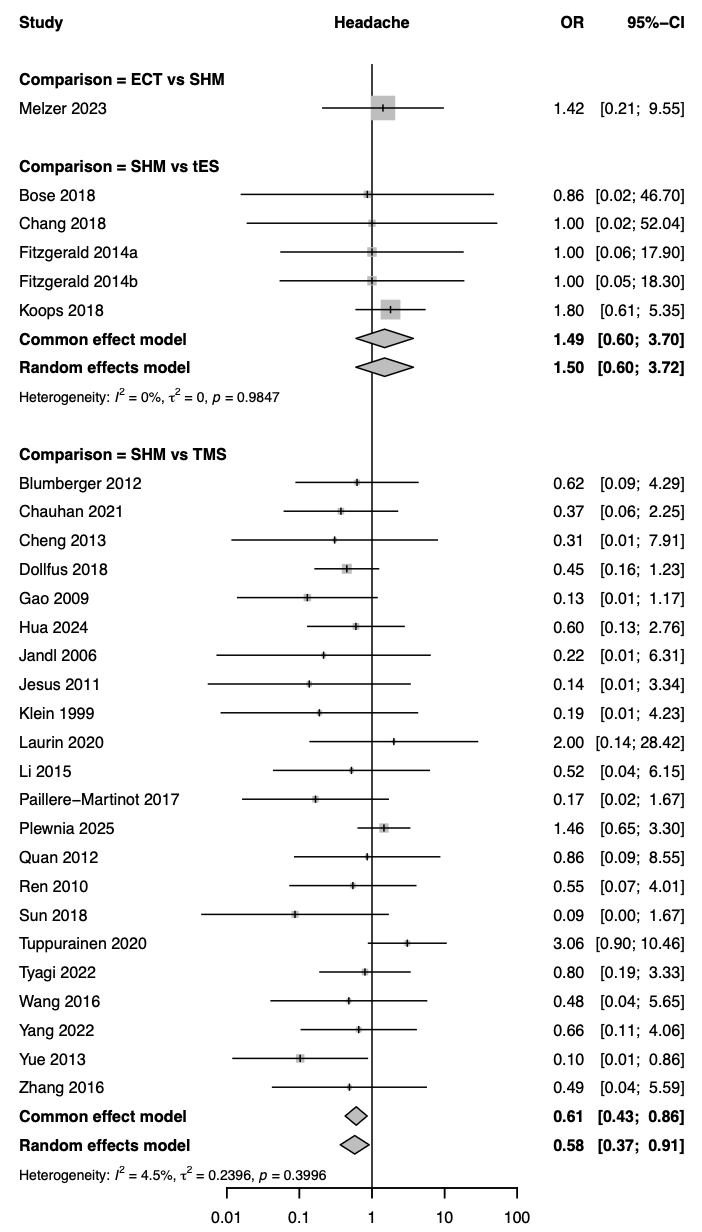


Forest-plot of results of pairwise meta-analyses including studies from Chinese mainland.

**
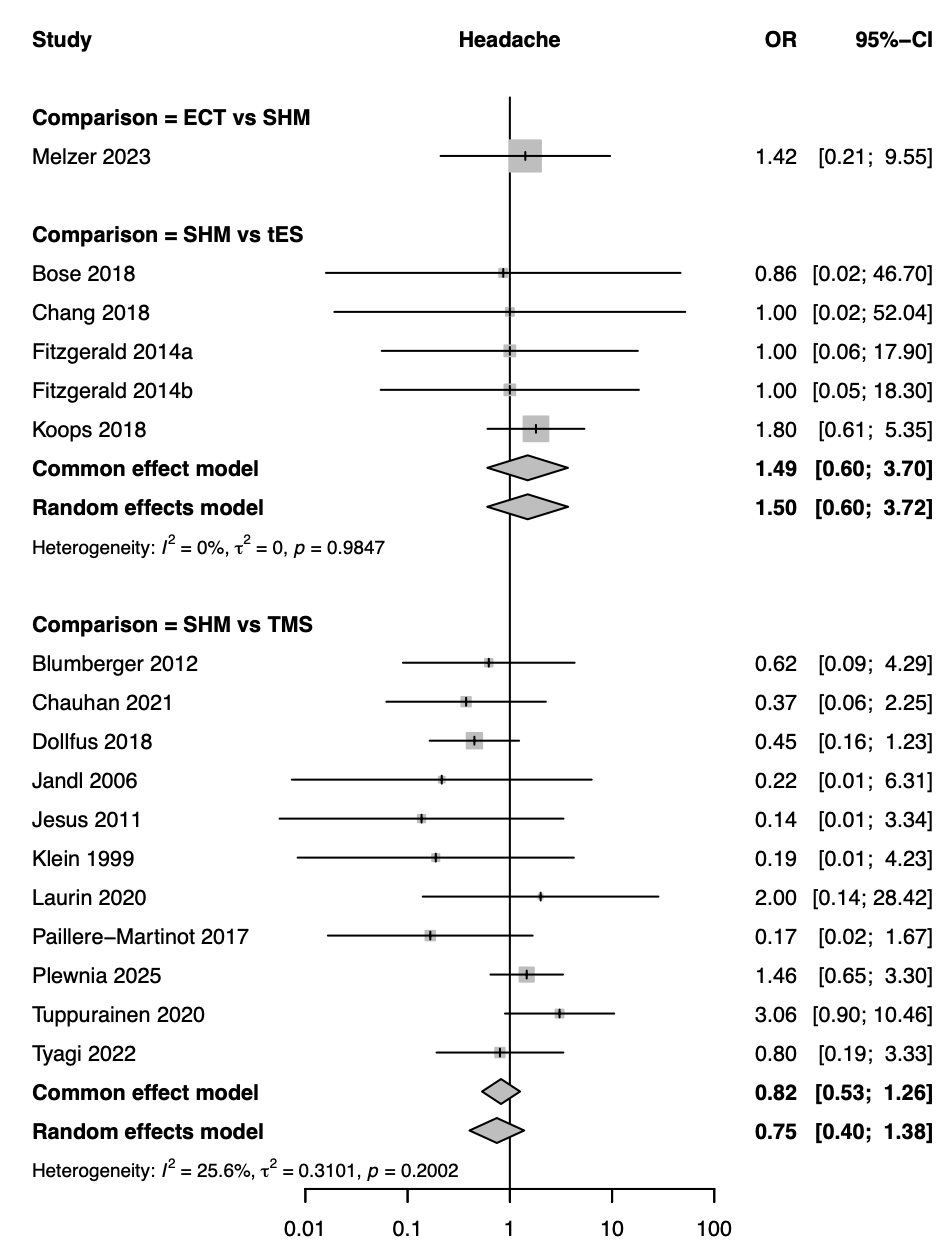
**

Forest-plot of results of pairwise meta-analyses excluding studies from Chinese mainland.

###
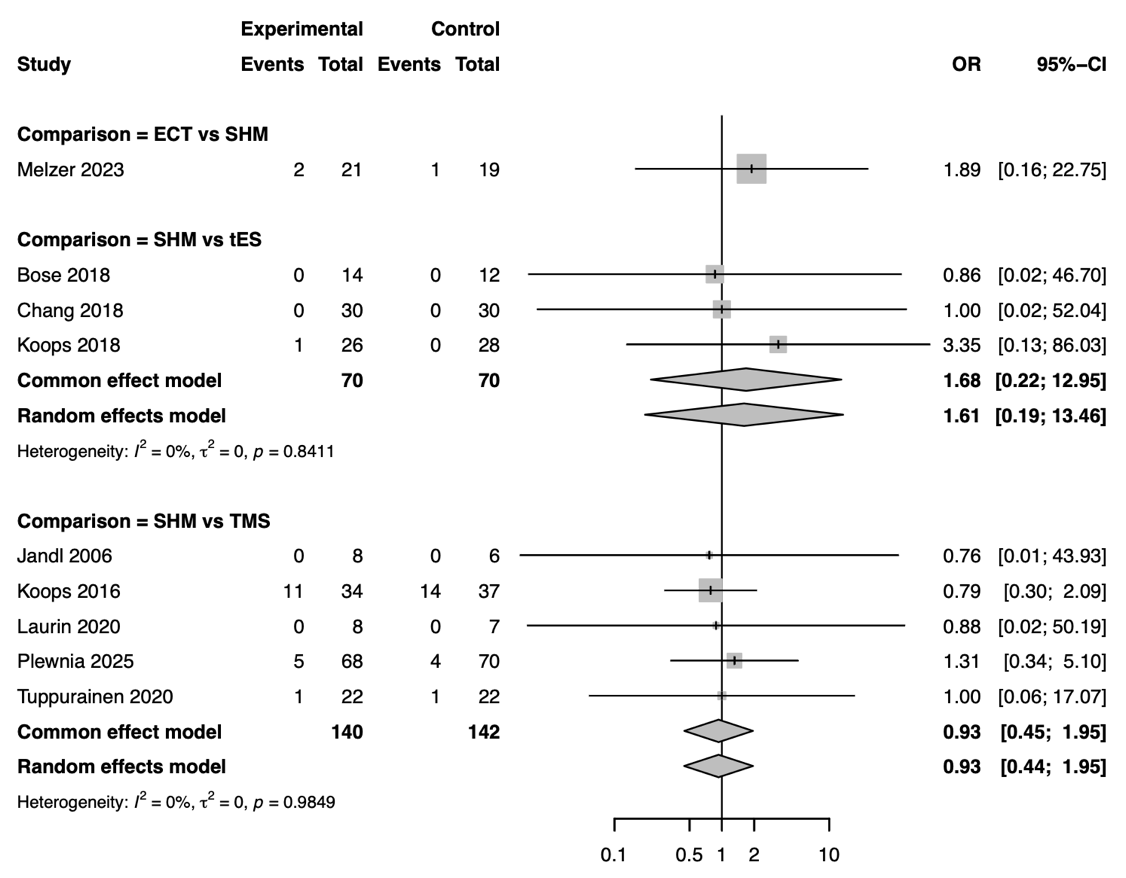
**9.11.2 Nausea**

### **9.11.3 Dizziness**

**
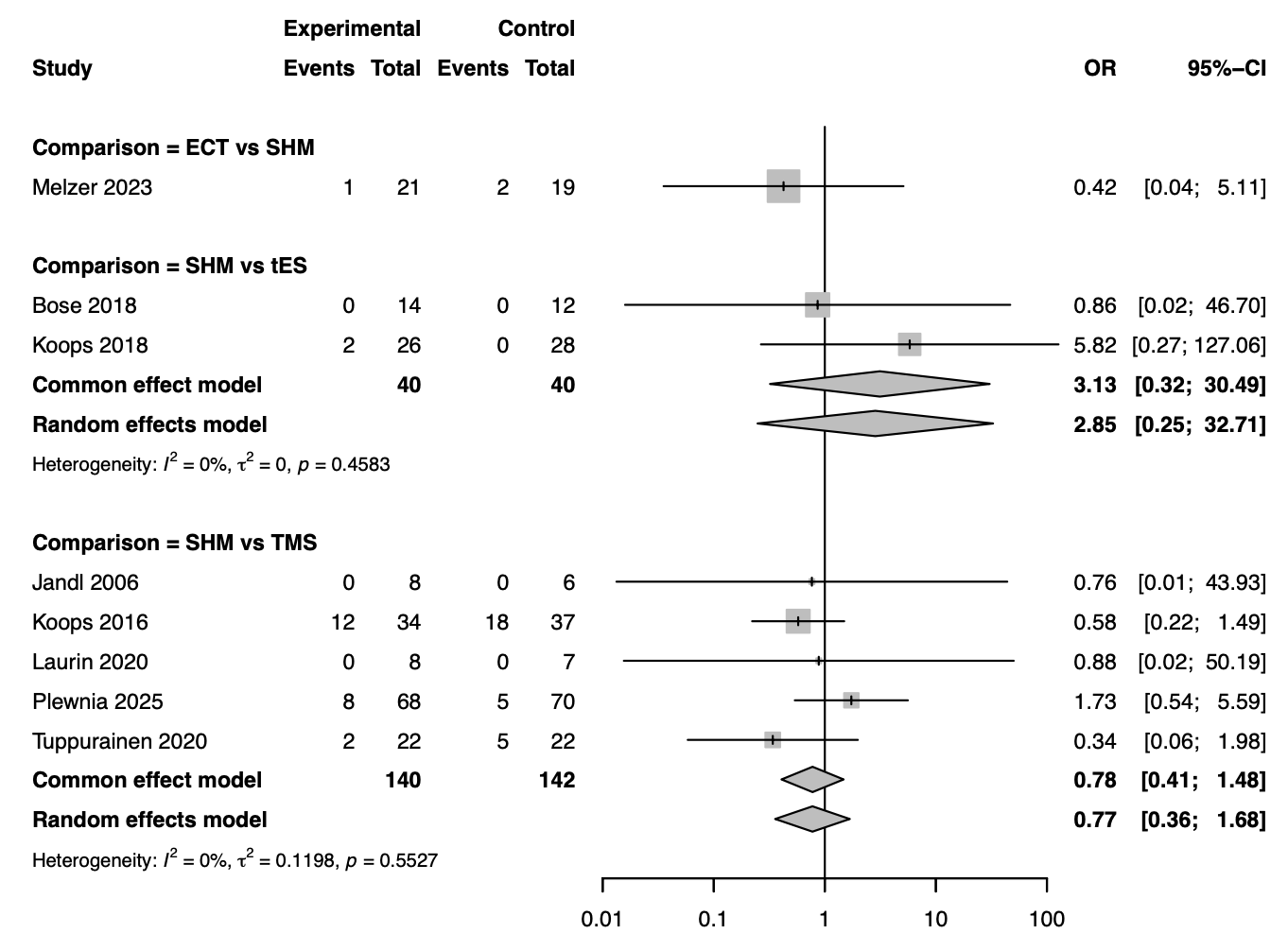
**

### **9.11.4 Sedation**


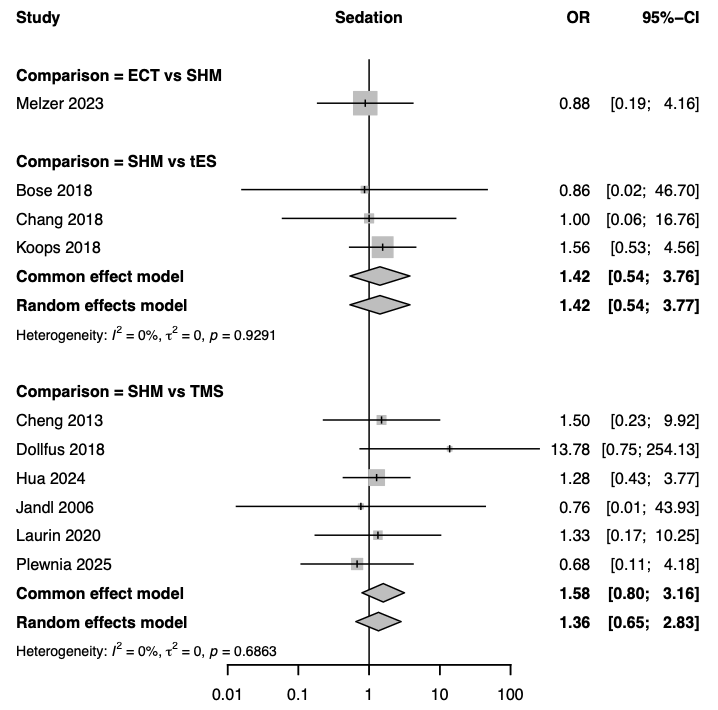


Forest-plot of results of pairwise meta-analyses including studies from Chinese mainland.

**
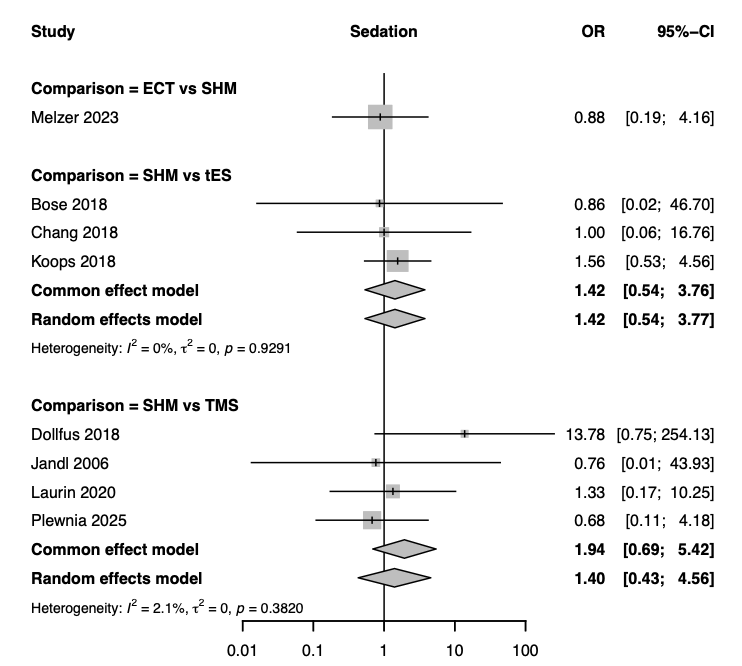
**

Forest-plot of results of pairwise meta-analyses excluding studies from Chinese mainland.

### **9.11.5 Depression**


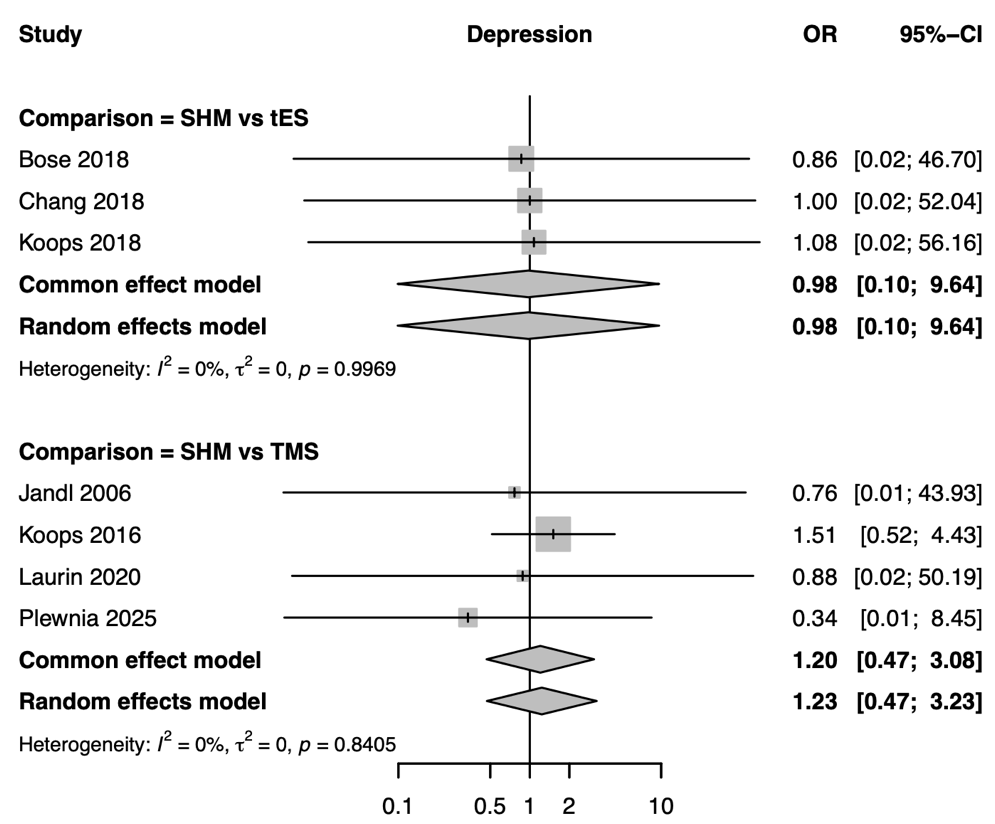


### **9.11.6 Dystonia**


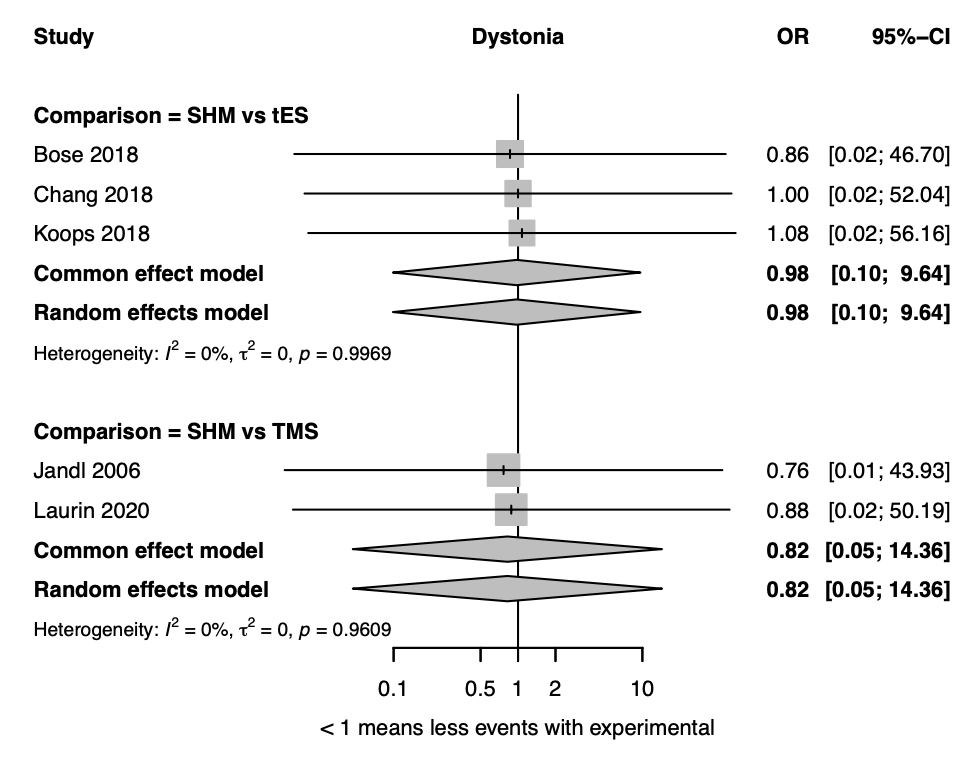


### **9.11.7 Mania**


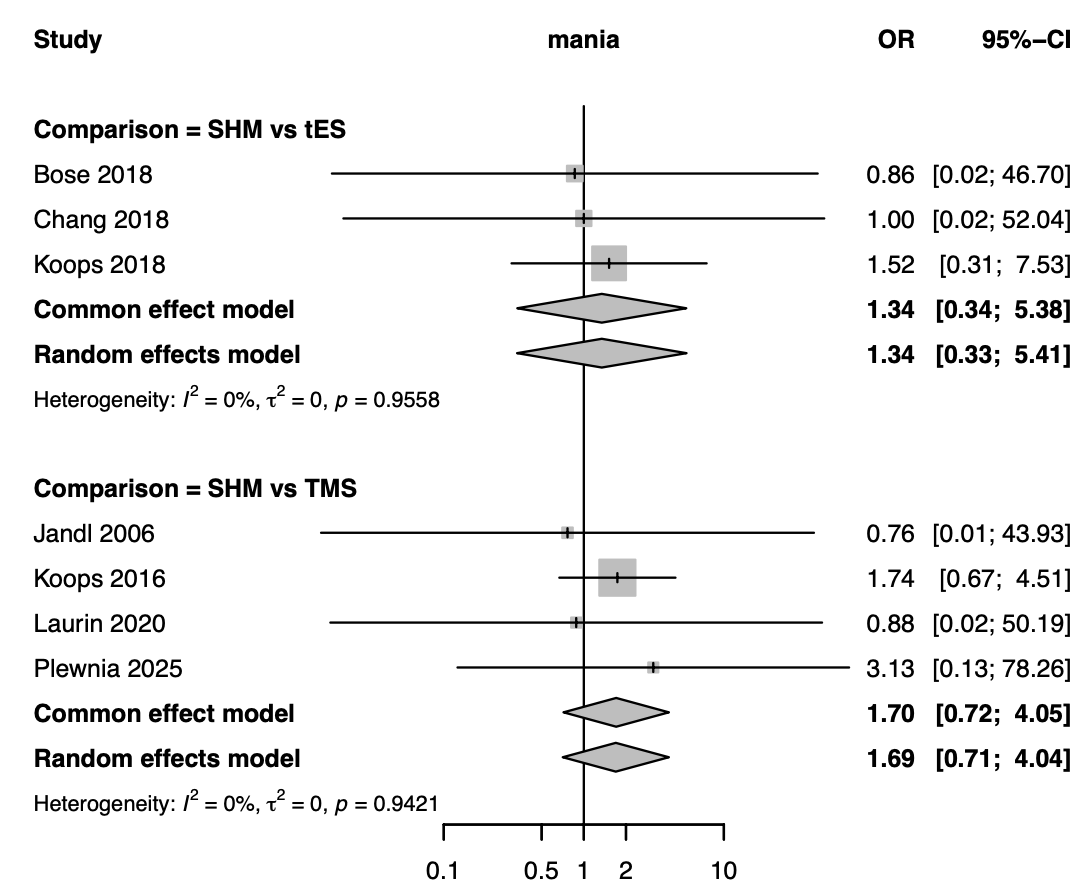


### **
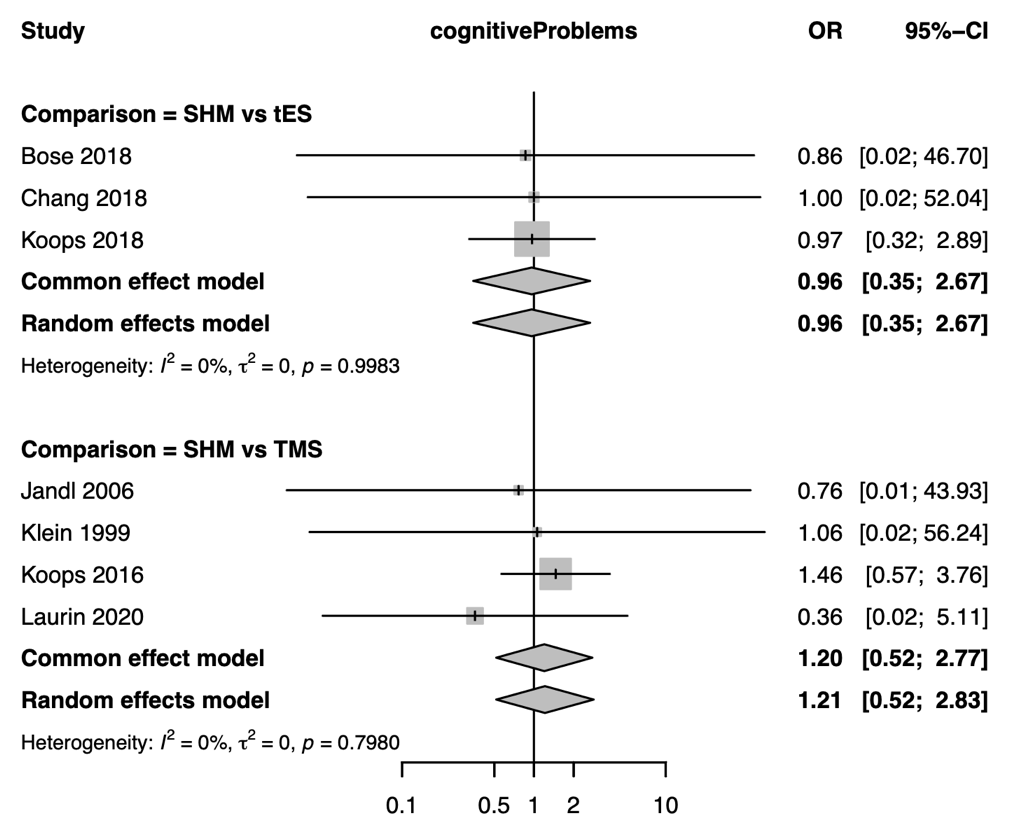
9.11.8 Cognitive problems**

### **
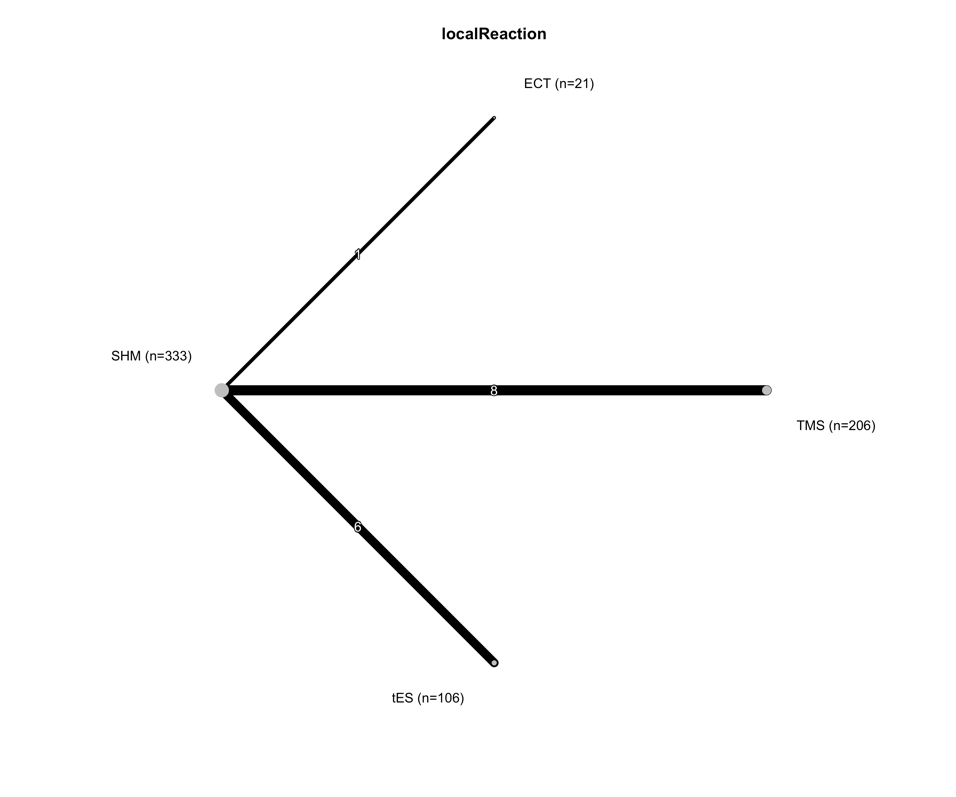
9.11.9 Local reaction**

Network plot including studies from Chinese mainland.

**
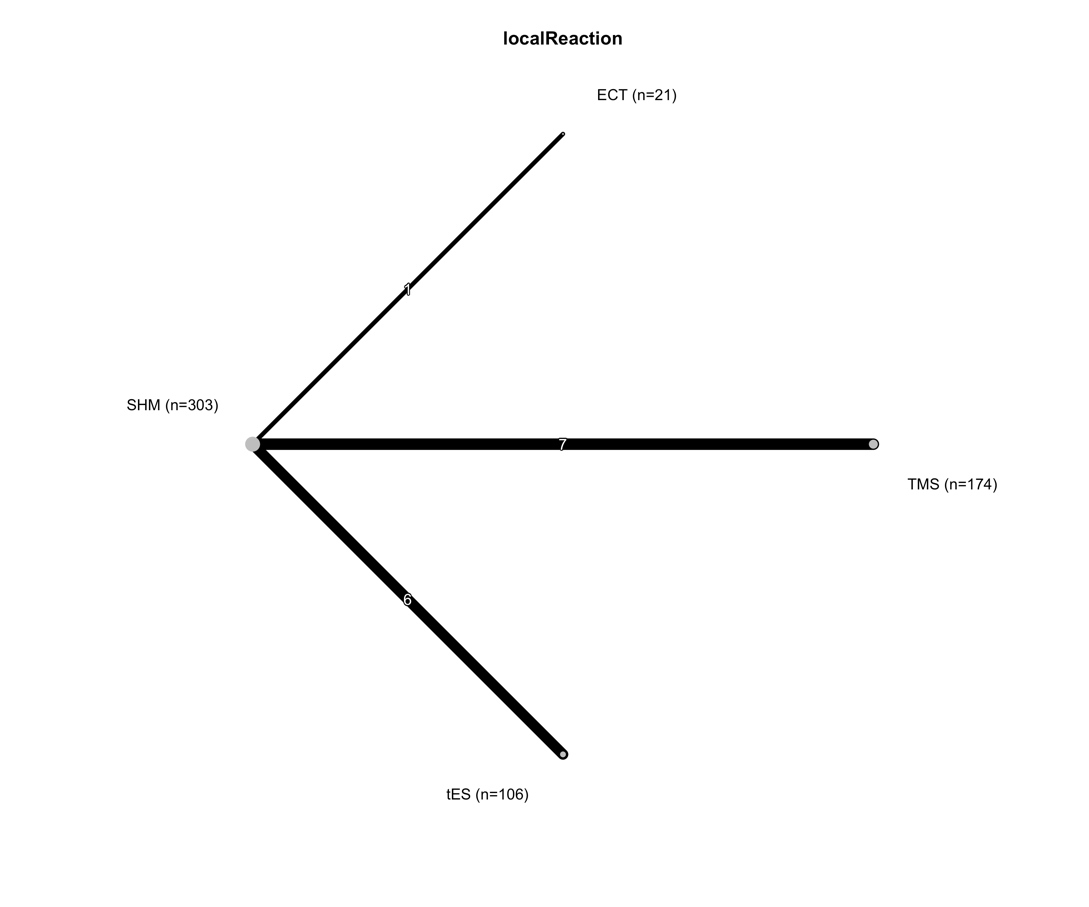
**

Network plot excluding studies from Chinese mainland.


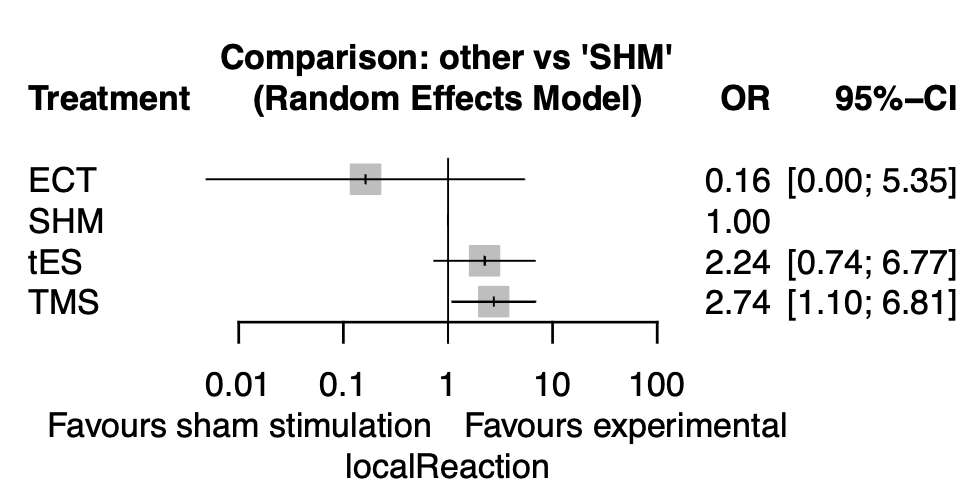


Forest-plot of results of network-meta-analysis including studies from Chinese mainland.

Quantifying heterogeneity / inconsistency:

tau^2 = 0.6722; tau = 0.8199; I^2 = 43.7% [0.0%; 70.6%]


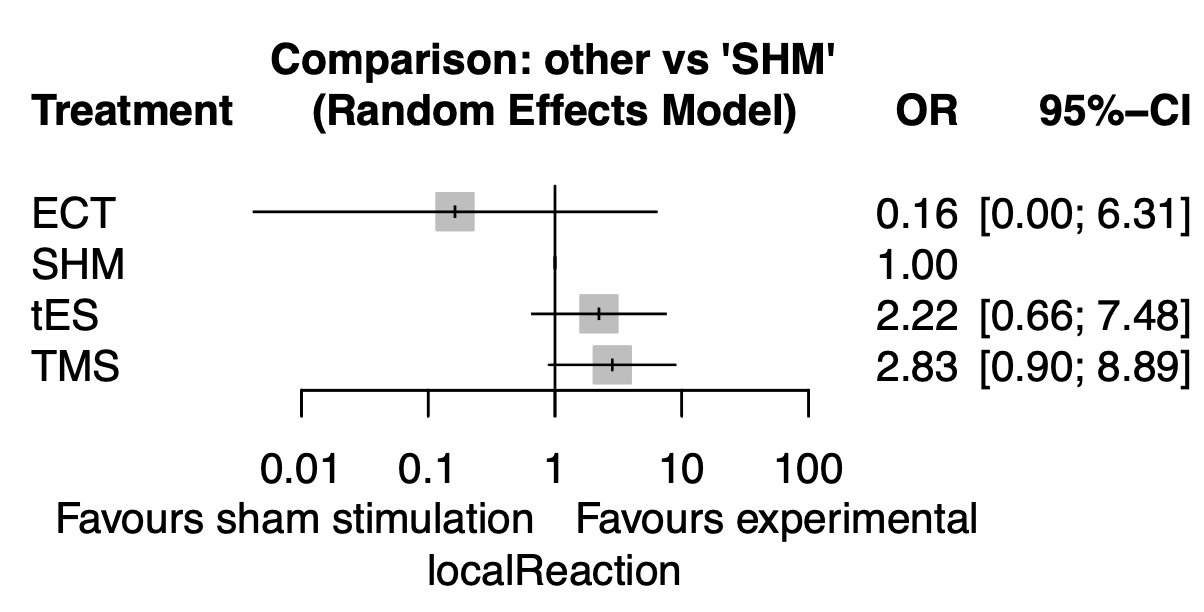


Forest-plot of results of network-meta-analysis excluding studies from Chinese mainland.

Quantifying heterogeneity / inconsistency:

tau^2 = 0.9791; tau = 0.9895; I^2 = 48.2% [0.0%; 73.4%]

| ECT | 0.16 (0.00, 5.35) | . | . |
| --- | --- | --- | --- |
| 0.16 (0.00, 5.35) | SHM | 0.45 (0.15, 1.35) | 0.37 (0.15, 0.91) |
| 0.07 (0.00, 2.84) | 0.45 (0.15, 1.35) | tES | . |
| 0.06 (0.00, 2.20) | 0.37 (0.15, 0.91) | 0.82 (0.19, 3.43) | TMS |

League-table of results of the network meta-analysis including studies from Chinese mainland.

| ECT | 0.16 (0.00, 6.31) | . | . |
| --- | --- | --- | --- |
| 0.16 (0.00, 6.31) | SHM | 0.45 (0.13, 1.51) | 0.35 (0.11, 1.11) |
| 0.07 (0.00, 3.46) | 0.45 (0.13, 1.51) | tES | . |
| 0.06 (0.00, 2.66) | 0.35 (0.11, 1.11) | 0.79 (0.15, 4.16) | TMS |

League-table of results of the network meta-analysis excluding studies from Chinese mainland.

**
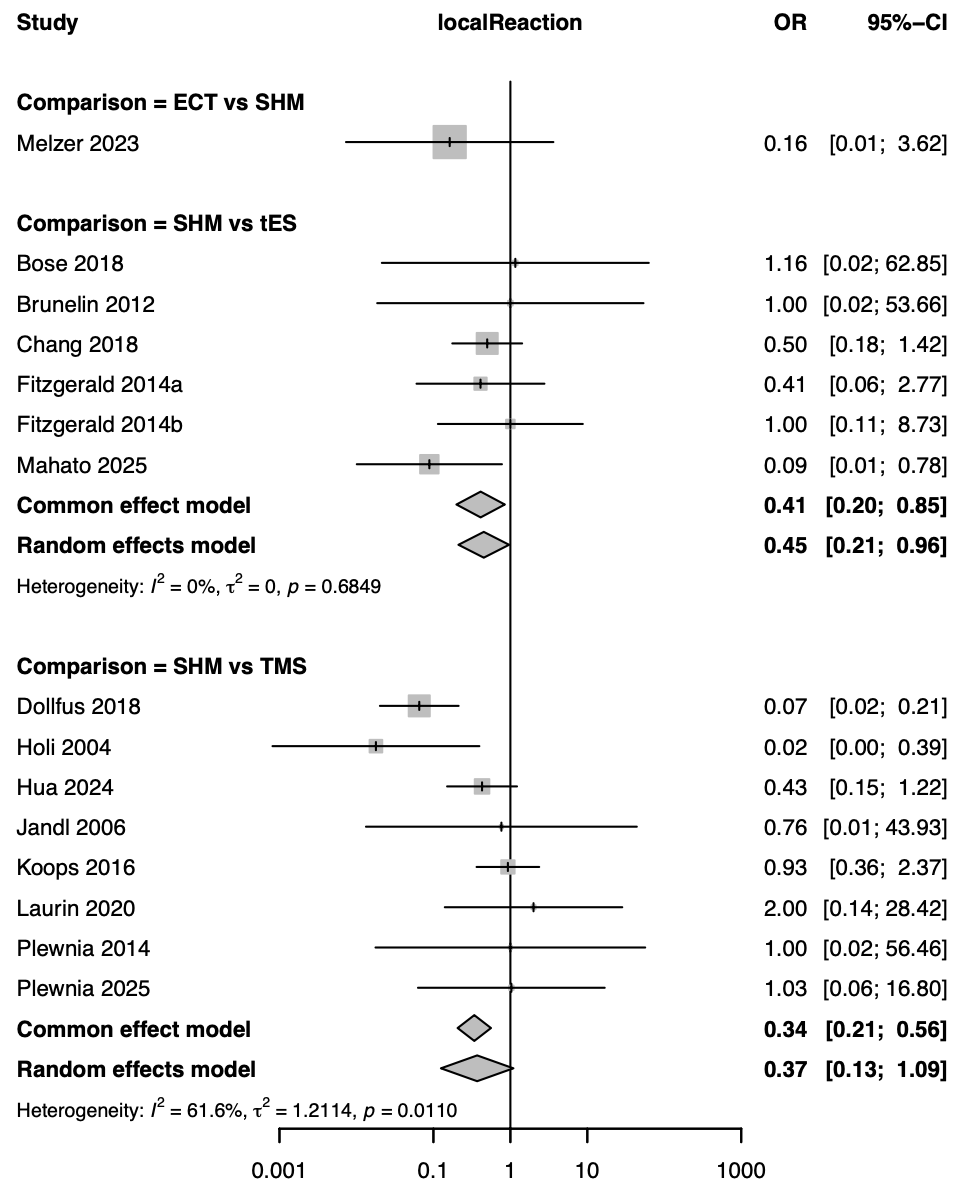
**

Forest-plot of results of pairwise meta-analyses including studies from Chinese mainland.

**
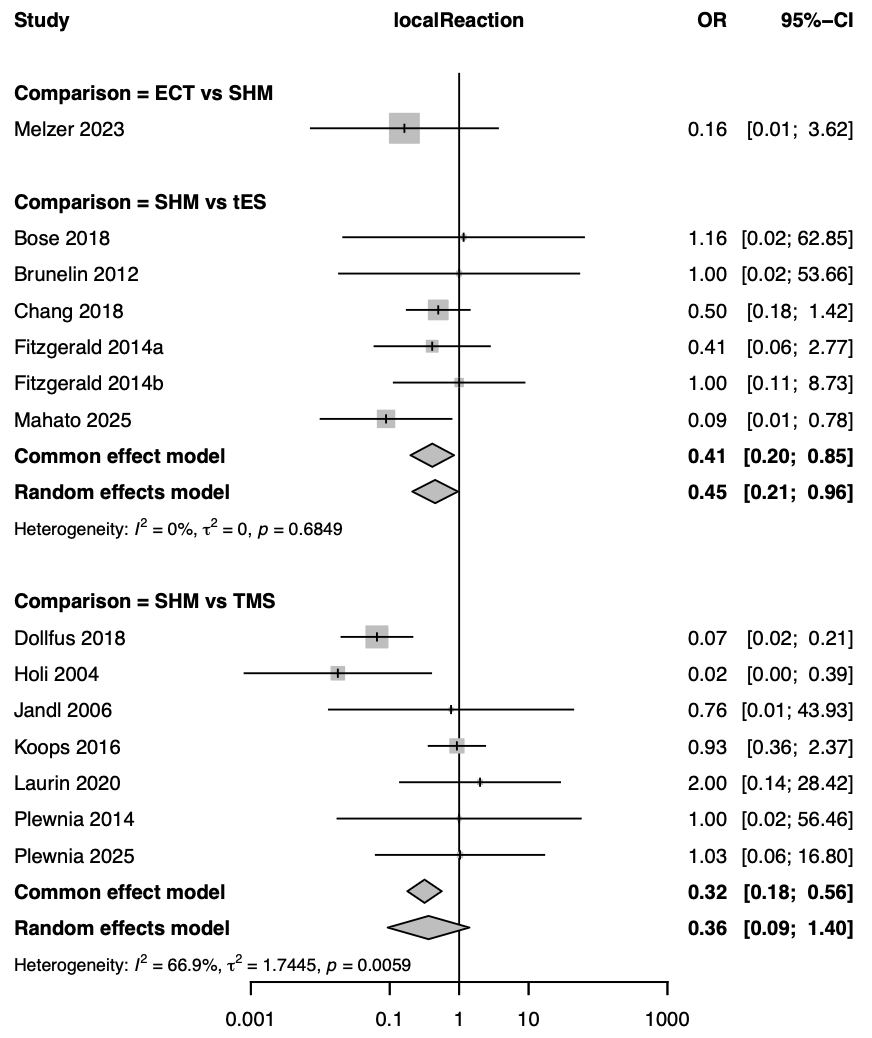
**

Forest-plot of results of pairwise meta-analyses excluding studies from Chinese mainland.

### **9.11.10 Hearing Problems**

**
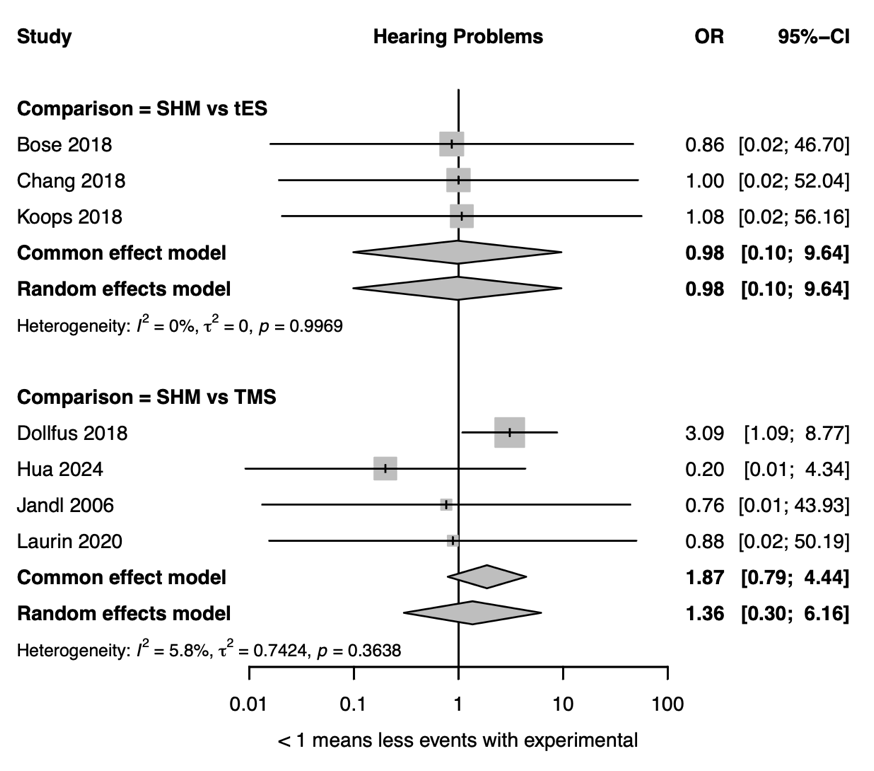
**

Forest-plot of results of pairwise meta-analyses including studies from Chinese mainland.


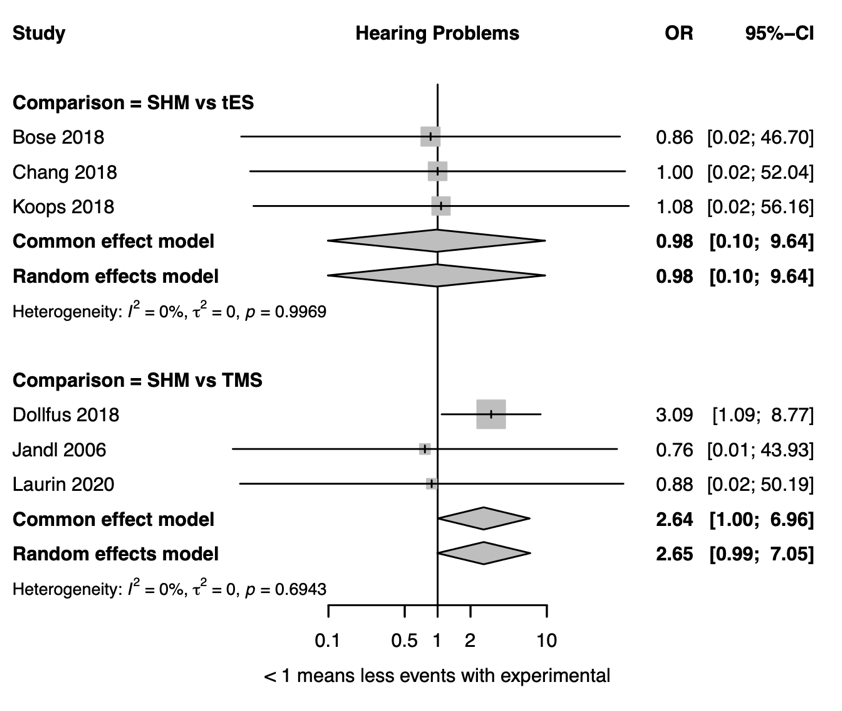


Forest-plot of results of pairwise meta-analyses excluding studies from Chinese mainland.

### **9.11.11 Myalgia**

**
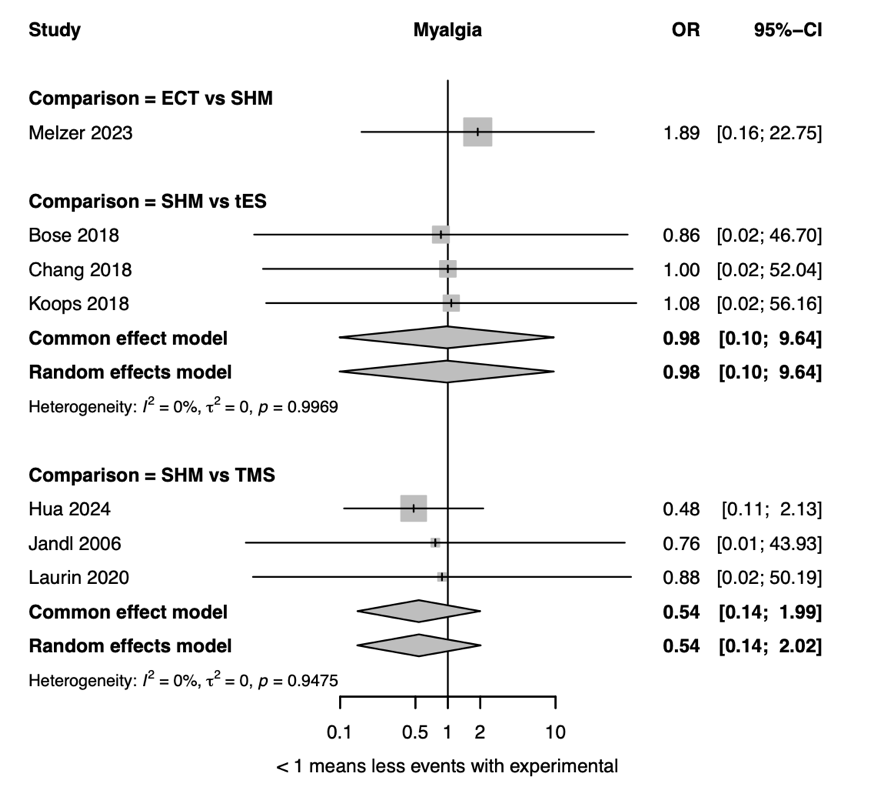
**

Forest-plot of results of pairwise meta-analyses including studies from Chinese mainland.

**
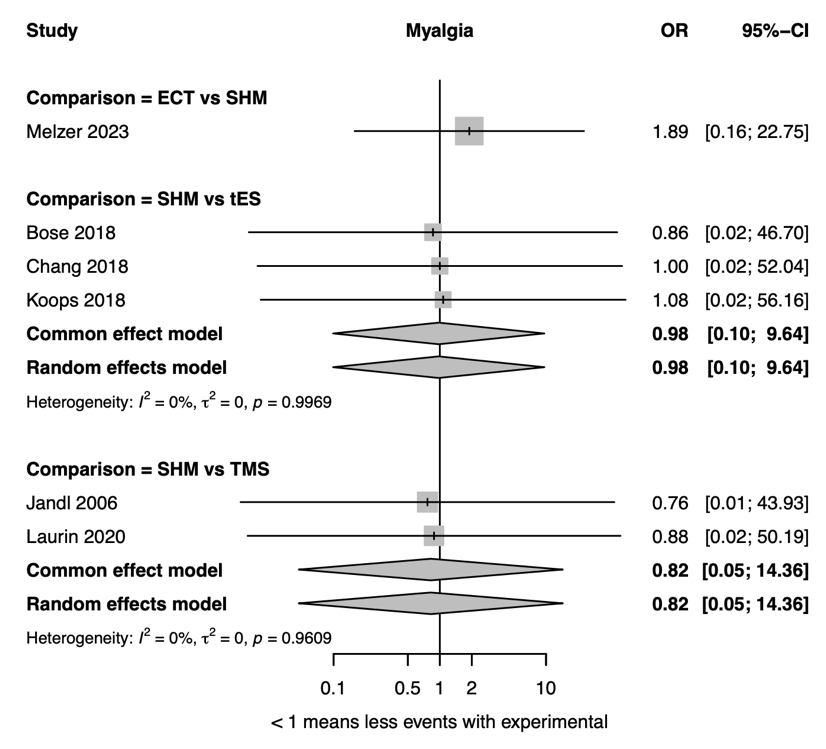
**

Forest-plot of results of pairwise meta-analyses excluding studies from Chinese mainland.

### **9.11.12 Seizures**

For this outcome, although 12 studies provided the data, 10 of them observed zero events in both arms. One study reported one seizure in the TMS group, while another reported one seizure in the sham stimulation group. Given this distribution, a network meta-analysis using the Mantel–Haenszel method is recommended for rare events, but the data is not feasible.

Forest-plot of results of pairwise meta-analyses including studies from Chinese mainland.

Forest-plot of results of pairwise meta-analyses excluding studies from Chinese mainland.

# **10. Subgroup analyses and meta-regressions**

In our protocol, we stated: *If network meta-regression is deemed appropriate and feasible for examining these potential effect modifiers, we will fit regressions in a Bayesian setting and assess the influence of modifiers by examining the credible intervals of the regression coefficients and evaluating changes in both heterogeneity and inconsistency between the unadjusted and adjusted models.*

In this review, most included trials had small sample sizes and compared NIBS with sham stimulation, with few studies examining head-to-head comparisons between active interventions. Therefore, the network had a star-like shape, and we did not conduct network meta-regression. Instead, we did pairwise analysis of the comparison of any NIBS with sham stimulation and examined the following potential effect modifiers with subgroup analyses and meta-regressions: (a) baseline severity, (b) definition of treatment-resistance, (c) duration of illness, (d) publication year, (e) sample size, (f) treatment duration, (g) number of sessions, and (h) clozapine resistance.

Legends for bubble plots: the size of the bubbles reflects the weight of each study, and different colors indicate different treatment comparisons (orange: TMS vs SHM, blue: ECT vs SHM, green: tES vs SHM).

## **10.1 Baseline severity**

## **10.2** **Definition of treatment-resistance**

## **10.3** **Illness Duration**

## **10.4 Publication year**

## **10.5 Sample size**

## **10.6 Treatment duration**

## **10.7 Sessions number**

## **10.8 Clozapine resistance**

# **11. Sensitivity analyses**

For each sensitivity analysis we present below:

-  Network plot

-  Forest-plot of results of network-meta-analysis (reference sham stimulation)

-  League-table of results of the network meta-analysis

-  Forest-plot of results of pairwise meta-analyses

When fewer than 10 studies reported on the secondary outcomes, we presented only the forest plots from the pairwise meta-analyses.

Legend for network plots: lines link treatments with direct comparisons in trials; thickness of lines corresponds to the number of trials evaluating the comparison; size of the nodes corresponds to the number of trials investigating the treatment.

Legend for forest-plots of results of network-meta-analysis: effect sizes are from the network-meta-analysis. The type of effect size measure is standardised mean difference (SMD). Order of treatments is according to the mean effect size. Reference is sham stimulation. The direction of the effect is indicated below the x-axis. In general, effect estimates to the left are in favor of NIBS treatment.

Legend for league tables: Order of treatments is according to the mean effect size. Results of the network-meta-analysis are presented in the left lower half and results of pairwise meta-analyses in the right upper half. Results in bold indicate 95% CI excluding no effect.

Legend for forest-plots of results of pairwise meta-analysis: Studies are ordered by comparison investigated (in alphabetical order) and a summary effect size is calculated by pairwise meta-analyses of all studies of a specific comparison. The type of effect size measure is standardised mean difference (SMD). Both fixed- and random-effects estimates were presented for sensitivity comparison. < 0 means better outcome with the first intervention in the comparison.

Abbreviations: ECT: Electroconvulsive therapy; MST: Magnetic seizure therapy; SHM: Sham therapy; TAU: Treatment as Usual; tES: Transcranial electrical stimulation; TMS: transcranial magnetic stimulation.

## **11.1 Different nodes for precise NIBS protocols**

In this sensitivity analysis, we explored the differences among various NIBS protocols, with consideration of stimulation frequency, target regions, and their combination (see Appendix 13 ‘Description of NIBS treatment’ below for a detailed description of the categories).

Each NIBS treatment was compared only to its corresponding sham stimulation; no comparisons were made between different sham stimulation modalities. Therefore, it was not possible to perform sensitivity analyses on different nodes for precise sham stimulations.

To assess the potential impact of including studies from Chinese mainland, we presented the sensitivity analysis results both with and without data from these studies.

### **11.1.1 Splitting NIBS into precise protocols, with consideration of frequency**

In the consideration of frequency, the NIBS treatments were classified as follows.

**ECT**

SC ECT, standard-charge ECT

LC ECT, low-charge ECT

**tES**

tDCS, transcranial direct current stimulation

tACS, transcranial alternating current stimulation

tRNS, transcranial random noise stimulation

**MST**

MST, magnetic seizure therapy

**TMS**

iTBS, intermittent theta burst stimulation

cTBS, continuous theta burst stimulation

αTMS, α-peak-frequency–guided transcranial magnetic stimulation

dTMS, deep transcranial magnetic stimulation

LF rTMS, low frequency rTMS

HF rTMS, high frequency rTMS

**TAU**

TAU, treatment as usual

**SHM**

SHM, sham therapy

Network plot including studies from Chinese mainland.

Network plot excluding studies from Chinese mainland.

Forest-plot of results of network-meta-analysis including studies from Chinese mainland.

Quantifying heterogeneity / inconsistency:

tau^2 = 0.2220; tau = 0.4712; I^2 = 68.5% [59.5%; 75.5%]

Forest-plot of results of network-meta-analysis excluding studies from Chinese mainland.

Quantifying heterogeneity / inconsistency:

tau^2 = 0.1336; tau = 0.3655; I^2 = 51% [29.8%; 65.8%]

| LC ECT | . | . | . | . | . | . | -0.61 (-2.09, 0.87) | . | . | . | . | . | . |
| --- | --- | --- | --- | --- | --- | --- | --- | --- | --- | --- | --- | --- | --- |
| -0.12 (-2.06, 1.82) | aTMS | . | . | . | . | . | . | . | . | . | -0.69 (-1.80, 0.42) | . | . |
| -0.29 (-1.93, 1.35) | -0.17 (-1.34, 1.00) | cTBS | . | -0.36 (-0.99, 0.27) | . | . | . | . | . | . | -0.32 (-0.76, 0.13) | . | . |
| -0.30 (-1.95, 1.35) | -0.18 (-1.36, 1.00) | -0.01 (-0.57, 0.55) | HF rTMS | -0.37 (-1.13, 0.38) | . | . | . | . | . | . | -0.41 (-0.86, 0.04) | . | . |
| -0.36 (-1.96, 1.25) | -0.23 (-1.36, 0.89) | -0.07 (-0.46, 0.33) | -0.06 (-0.49, 0.38) | LF rTMS | . | . | . | . | . | . | -0.49 (-0.69,-0.30) | . | . |
| -0.44 (-2.67, 1.79) | -0.32 (-2.22, 1.59) | -0.15 (-1.75, 1.45) | -0.14 (-1.75, 1.47) | -0.08 (-1.65, 1.48) | tRNS | . | . | . | . | . | -0.37 (-1.93, 1.18) | . | . |
| -0.51 (-2.51, 1.49) | -0.39 (-2.23, 1.45) | -0.22 (-1.74, 1.30) | -0.21 (-1.74, 1.32) | -0.15 (-1.64, 1.33) | -0.07 (-2.21, 2.07) | MST | -0.10 (-1.44, 1.25) | . | . | . | . | . | . |
| -0.61 (-2.09, 0.87) | -0.48 (-1.74, 0.77) | -0.32 (-1.03, 0.39) | -0.31 (-1.03, 0.42) | -0.25 (-0.88, 0.38) | -0.17 (-1.83, 1.50) | -0.10 (-1.44, 1.25) | SC ECT | . | . | . | -0.21 (-0.80, 0.39) | . | -1.33 (-2.49,-0.18) |
| -0.61 (-2.39, 1.16) | -0.49 (-1.84, 0.85) | -0.32 (-1.18, 0.53) | -0.31 (-1.19, 0.56) | -0.26 (-1.05, 0.53) | -0.18 (-1.91, 1.56) | -0.10 (-1.76, 1.56) | -0.01 (-0.98, 0.96) | iTBS | . | . | -0.20 (-0.96, 0.57) | . | . |
| -0.73 (-2.36, 0.90) | -0.61 (-1.76, 0.55) | -0.44 (-0.95, 0.07) | -0.43 (-0.96, 0.10) | -0.37 (-0.75, 0.01) | -0.29 (-1.88, 1.30) | -0.22 (-1.73, 1.29) | -0.12 (-0.81, 0.56) | -0.11 (-0.95, 0.72) | tDCS | . | -0.10 (-0.44, 0.23) | 0.34 (-1.04, 1.71) | . |
| -0.75 (-2.97, 1.48) | -0.62 (-2.52, 1.28) | -0.45 (-2.05, 1.14) | -0.45 (-2.05, 1.15) | -0.39 (-1.95, 1.17) | -0.31 (-2.50, 1.88) | -0.24 (-2.37, 1.90) | -0.14 (-1.80, 1.52) | -0.13 (-1.86, 1.59) | -0.02 (-1.60, 1.56) | dTMS | -0.07 (-1.61, 1.48) | . | . |
| -0.81 (-2.41, 0.78) | -0.69 (-1.80, 0.42) | -0.52 (-0.90,-0.14) | -0.51 (-0.93,-0.10) | -0.46 (-0.64,-0.27) | -0.37 (-1.93, 1.18) | -0.30 (-1.78, 1.17) | -0.21 (-0.80, 0.39) | -0.20 (-0.96, 0.57) | -0.08 (-0.42, 0.25) | -0.07 (-1.61, 1.48) | SHM | -0.13 (-1.02, 0.76) | . |
| -0.87 (-2.67, 0.93) | -0.75 (-2.13, 0.64) | -0.58 (-1.50, 0.34) | -0.57 (-1.50, 0.36) | -0.51 (-1.37, 0.34) | -0.43 (-2.20, 1.33) | -0.36 (-2.05, 1.33) | -0.26 (-1.29, 0.76) | -0.26 (-1.39, 0.88) | -0.14 (-1.01, 0.72) | -0.13 (-1.88, 1.63) | -0.06 (-0.89, 0.77) | tACS | . |
| -1.94 (-3.82,-0.06) | -1.82 (-3.53,-0.11) | -1.65 (-3.01,-0.29) | -1.64 (-3.01,-0.28) | -1.58 (-2.90,-0.27) | -1.50 (-3.53, 0.52) | -1.43 (-3.20, 0.34) | -1.33 (-2.49,-0.18) | -1.33 (-2.84, 0.18) | -1.21 (-2.55, 0.13) | -1.19 (-3.22, 0.83) | -1.13 (-2.43, 0.17) | -1.07 (-2.61, 0.48) | TAU |

League-table of results of the network meta-analysis including studies from Chinese mainland.

| aTMS | . | . | . | . | . | . | . | . | -0.69 (-1.63, 0.25) | . | . |
| --- | --- | --- | --- | --- | --- | --- | --- | --- | --- | --- | --- |
| -0.27 (-1.71, 1.16) | tACS | . | . | . | . | . | -0.34 (-1.58, 0.91) | . | -0.39 (-1.64, 0.86) | . | . |
| -0.31 (-1.35, 0.72) | -0.04 (-1.21, 1.13) | HF rTMS | . | . | . | -0.55 (-1.43, 0.32) | . | . | -0.29 (-0.76, 0.19) | . | . |
| -0.32 (-2.04, 1.40) | -0.04 (-1.84, 1.76) | -0.00 (-1.51, 1.50) | tRNS | . | . | . | . | . | -0.37 (-1.82, 1.07) | . | . |
| -0.48 (-1.50, 0.53) | -0.21 (-1.35, 0.94) | -0.17 (-0.74, 0.40) | -0.17 (-1.66, 1.32) | cTBS | . | -0.13 (-0.82, 0.56) | . | . | -0.15 (-0.55, 0.26) | . | . |
| -0.49 (-1.56, 0.59) | -0.21 (-1.42, 0.99) | -0.18 (-0.86, 0.51) | -0.17 (-1.70, 1.36) | -0.00 (-0.65, 0.64) | SC ECT | . | . | . | -0.20 (-0.72, 0.32) | . | -1.33 (-2.33,-0.34) |
| -0.58 (-1.55, 0.39) | -0.31 (-1.42, 0.80) | -0.27 (-0.75, 0.21) | -0.27 (-1.73, 1.20) | -0.10 (-0.51, 0.32) | -0.09 (-0.67, 0.49) | LF rTMS | . | . | -0.13 (-0.40, 0.13) | . | . |
| -0.59 (-1.57, 0.40) | -0.31 (-1.39, 0.77) | -0.27 (-0.80, 0.25) | -0.27 (-1.74, 1.20) | -0.10 (-0.57, 0.37) | -0.10 (-0.69, 0.50) | -0.00 (-0.39, 0.38) | tDCS | . | -0.10 (-0.39, 0.18) | . | . |
| -0.62 (-2.34, 1.09) | -0.35 (-2.14, 1.45) | -0.31 (-1.81, 1.19) | -0.31 (-2.34, 1.72) | -0.14 (-1.62, 1.34) | -0.14 (-1.66, 1.39) | -0.04 (-1.50, 1.41) | -0.04 (-1.50, 1.42) | dTMS | -0.07 (-1.50, 1.37) | . | . |
| -0.69 (-1.63, 0.25) | -0.42 (-1.50, 0.67) | -0.38 (-0.82, 0.06) | -0.37 (-1.82, 1.07) | -0.21 (-0.58, 0.17) | -0.20 (-0.72, 0.32) | -0.11 (-0.36, 0.14) | -0.10 (-0.39, 0.18) | -0.07 (-1.50, 1.37) | SHM | -0.42 (-1.39, 0.56) | . |
| -1.11 (-2.46, 0.24) | -0.84 (-2.29, 0.62) | -0.80 (-1.87, 0.27) | -0.79 (-2.53, 0.95) | -0.63 (-1.67, 0.42) | -0.62 (-1.73, 0.48) | -0.53 (-1.53, 0.48) | -0.52 (-1.54, 0.49) | -0.49 (-2.22, 1.25) | -0.42 (-1.39, 0.56) | iTBS | . |
| -1.82 (-3.29,-0.36) | -1.55 (-3.11, 0.01) | -1.51 (-2.72,-0.30) | -1.51 (-3.33, 0.32) | -1.34 (-2.52,-0.15) | -1.33 (-2.33,-0.34) | -1.24 (-2.39,-0.09) | -1.24 (-2.40,-0.07) | -1.20 (-3.02, 0.62) | -1.13 (-2.26,-0.01) | -0.71 (-2.20, 0.78) | TAU |

League-table of results of the network meta-analysis excluding studies from Chinese mainland.

Forest-plot of results of pairwise meta-analyses including studies from Chinese mainland.

Forest-plot of results of pairwise meta-analyses excluding studies from Chinese mainland.

### **11.1.2 Splitting NIBS into precise protocols, with consideration of targets**

In the consideration of targets, the NIBS treatments were classified as follows.

**ECT**

SC ECT-BT, standard-charge ECT, bitemporal

LC ECT-BT, low-charge ECT, bitemporal

**tES**

tDCS-FT, transcranial direct current stimulation, frontal-temporal

tDCS-TT, transcranial direct current stimulation, temporal-temporal

tACS-FT, alpha transcranial alternating current stimulation, frontal-temporal

tRNS-FT, transcranial random noise stimulation, frontal-temporal

**MST**

MST, magnetic seizure therapy

**TMS**

TBS-F theta burst stimulation, frontal

TBS-C, theta burst stimulation, cerebellum

TBS-T, theta burst stimulation, tempoparietal

dTMS-T, deep transcranial magnetic stimulation, tempoparietal

rTMS-T, low frequency rTMS, tempoparietal

rTMS-F, low frequency rTMS, frontal

**TAU**

TAU, treatment as usual

**SHM**

SHM, sham therapy

Network plot including studies from Chinese mainland.

Network plot excluding studies from Chinese mainland.

Forest-plot of results of network-meta-analysis including studies from Chinese mainland.

Quantifying heterogeneity / inconsistency:

tau^2 = 0.2126; tau = 0.4611; I^2 = 67.4% [57.9%; 74.8%]

Forest-plot of results of network-meta-analysis excluding studies from Chinese mainland.

Quantifying heterogeneity / inconsistency:

tau^2 = 0.1431; tau = 0.3783; I^2 = 53.1% [32.8%; 67.3%]

| LC ECT-BT | . | . | . | . | . | . | . | -0.61 (-2.08, 0.86) | . | . | . | . | . | . |
| --- | --- | --- | --- | --- | --- | --- | --- | --- | --- | --- | --- | --- | --- | --- |
| -0.10 (-1.98, 1.78) | TBS-F | . | . | . | . | . | . | . | . | . | -0.71 (-1.73, 0.31) | . | . | . |
| -0.28 (-1.91, 1.34) | -0.19 (-1.27, 0.90) | TBS-T | -0.36 (-0.98, 0.26) | . | . | . | . | . | . | . | -0.32 (-0.75, 0.12) | . | . | . |
| -0.34 (-1.93, 1.26) | -0.24 (-1.28, 0.80) | -0.05 (-0.45, 0.34) | rTMS-T | 0.03 (-0.69, 0.74) | . | . | . | . | . | . | -0.52 (-0.73,-0.31) | . | . | . |
| -0.35 (-1.95, 1.26) | -0.25 (-1.31, 0.81) | -0.06 (-0.53, 0.41) | -0.01 (-0.35, 0.33) | rTMS-F | . | . | . | . | . | . | -0.49 (-0.78,-0.20) | . | . | . |
| -0.44 (-2.65, 1.77) | -0.34 (-2.19, 1.51) | -0.15 (-1.74, 1.44) | -0.10 (-1.66, 1.45) | -0.09 (-1.66, 1.48) | tRNS-FT | . | . | . | . | . | -0.37 (-1.92, 1.17) | . | . | . |
| -0.51 (-2.49, 1.47) | -0.41 (-2.19, 1.37) | -0.23 (-1.73, 1.28) | -0.17 (-1.65, 1.30) | -0.17 (-1.65, 1.32) | -0.07 (-2.20, 2.05) | MST | . | -0.10 (-1.43, 1.24) | . | . | . | . | . | . |
| -0.56 (-2.49, 1.36) | -0.46 (-1.96, 1.03) | -0.28 (-1.44, 0.88) | -0.23 (-1.34, 0.89) | -0.22 (-1.35, 0.92) | -0.13 (-2.02, 1.77) | -0.05 (-1.88, 1.77) | tDCS-TT | . | . | . | -0.25 (-1.35, 0.85) | . | . | . |
| -0.61 (-2.08, 0.86) | -0.51 (-1.69, 0.67) | -0.32 (-1.02, 0.38) | -0.27 (-0.90, 0.35) | -0.26 (-0.92, 0.39) | -0.17 (-1.82, 1.48) | -0.10 (-1.43, 1.24) | -0.04 (-1.29, 1.20) | SC ECT-BT | . | . | -0.21 (-0.80, 0.39) | . | . | -1.33 (-2.47,-0.19) |
| -0.75 (-2.37, 0.87) | -0.65 (-1.72, 0.43) | -0.46 (-0.97, 0.05) | -0.41 (-0.81,-0.01) | -0.40 (-0.85, 0.05) | -0.31 (-1.89, 1.27) | -0.23 (-1.73, 1.26) | -0.18 (-1.33, 0.97) | -0.14 (-0.82, 0.55) | tDCS-FT | . | -0.09 (-0.44, 0.26) | 0.34 (-1.03, 1.70) | . | . |
| -0.75 (-2.95, 1.46) | -0.65 (-2.49, 1.20) | -0.46 (-2.04, 1.12) | -0.41 (-1.96, 1.14) | -0.40 (-1.96, 1.16) | -0.31 (-2.48, 1.87) | -0.24 (-2.35, 1.88) | -0.18 (-2.07, 1.70) | -0.14 (-1.78, 1.51) | -0.00 (-1.57, 1.57) | dTMS-T | -0.07 (-1.60, 1.47) | . | . | . |
| -0.81 (-2.40, 0.77) | -0.71 (-1.73, 0.31) | -0.53 (-0.90,-0.15) | -0.48 (-0.68,-0.27) | -0.47 (-0.75,-0.18) | -0.37 (-1.92, 1.17) | -0.30 (-1.76, 1.16) | -0.25 (-1.35, 0.85) | -0.21 (-0.80, 0.39) | -0.07 (-0.41, 0.28) | -0.07 (-1.60, 1.47) | SHM | -0.13 (-1.01, 0.74) | -0.42 (-1.54, 0.70) | . |
| -0.88 (-2.66, 0.91) | -0.78 (-2.09, 0.53) | -0.59 (-1.50, 0.31) | -0.54 (-1.39, 0.31) | -0.53 (-1.40, 0.34) | -0.44 (-2.19, 1.31) | -0.37 (-2.04, 1.31) | -0.31 (-1.69, 1.06) | -0.27 (-1.28, 0.74) | -0.13 (-0.99, 0.73) | -0.13 (-1.87, 1.61) | -0.06 (-0.89, 0.76) | tACS-FT | . | . |
| -1.23 (-3.17, 0.71) | -1.13 (-2.65, 0.38) | -0.95 (-2.13, 0.23) | -0.90 (-2.03, 0.24) | -0.89 (-2.04, 0.27) | -0.79 (-2.70, 1.11) | -0.72 (-2.56, 1.12) | -0.67 (-2.24, 0.90) | -0.62 (-1.89, 0.64) | -0.49 (-1.66, 0.69) | -0.49 (-2.39, 1.41) | -0.42 (-1.54, 0.70) | -0.35 (-1.75, 1.04) | TBS-C | . |
| -1.94 (-3.80,-0.08) | -1.84 (-3.48,-0.20) | -1.66 (-2.99,-0.32) | -1.60 (-2.90,-0.31) | -1.60 (-2.91,-0.28) | -1.50 (-3.51, 0.50) | -1.43 (-3.18, 0.32) | -1.38 (-3.07, 0.31) | -1.33 (-2.47,-0.19) | -1.20 (-2.52, 0.13) | -1.19 (-3.20, 0.81) | -1.13 (-2.41, 0.16) | -1.06 (-2.59, 0.46) | -0.71 (-2.41, 0.99) | TAU |

League-table of results of the network meta-analysis including studies from Chinese mainland.

| tACS-FT | . | . | . | . | . | . | -0.34 (-1.60, 0.92) | . | -0.39 (-1.65, 0.87) | . | . |
| --- | --- | --- | --- | --- | --- | --- | --- | --- | --- | --- | --- |
| -0.03 (-1.85, 1.79) | tRNS-FT | . | . | . | . | . | . | . | -0.37 (-1.83, 1.08) | . | . |
| -0.16 (-1.62, 1.30) | -0.13 (-1.87, 1.62) | tDCS-TT | . | . | . | . | . | . | -0.25 (-1.22, 0.72) | . | . |
| -0.18 (-1.34, 0.98) | -0.15 (-1.65, 1.36) | -0.02 (-1.06, 1.02) | TBS-T | . | -0.13 (-0.83, 0.57) | . | . | . | -0.15 (-0.56, 0.27) | . | . |
| -0.21 (-1.42, 1.01) | -0.17 (-1.72, 1.38) | -0.05 (-1.15, 1.06) | -0.03 (-0.68, 0.63) | SC ECT-BT | . | . | . | . | -0.20 (-0.73, 0.33) | . | -1.33 (-2.35,-0.32) |
| -0.21 (-1.33, 0.92) | -0.17 (-1.65, 1.30) | -0.05 (-1.05, 0.95) | -0.03 (-0.45, 0.39) | -0.00 (-0.59, 0.58) | rTMS-T | 0.22 (-1.07, 1.52) | . | . | -0.21 (-0.47, 0.05) | . | . |
| -0.22 (-1.42, 0.99) | -0.18 (-1.72, 1.35) | -0.06 (-1.14, 1.03) | -0.04 (-0.66, 0.59) | -0.01 (-0.73, 0.71) | -0.01 (-0.55, 0.53) | rTMS-F | . | . | -0.17 (-0.67, 0.34) | . | . |
| -0.32 (-1.42, 0.78) | -0.29 (-1.77, 1.20) | -0.16 (-1.18, 0.86) | -0.14 (-0.63, 0.35) | -0.11 (-0.73, 0.50) | -0.11 (-0.51, 0.29) | -0.10 (-0.69, 0.48) | tDCS-FT | . | -0.09 (-0.40, 0.22) | . | . |
| -0.34 (-2.16, 1.47) | -0.31 (-2.36, 1.74) | -0.18 (-1.92, 1.56) | -0.16 (-1.66, 1.33) | -0.14 (-1.68, 1.40) | -0.13 (-1.60, 1.33) | -0.13 (-1.65, 1.40) | -0.02 (-1.50, 1.45) | dTMS-T | -0.07 (-1.51, 1.38) | . | . |
| -0.41 (-1.51, 0.69) | -0.37 (-1.83, 1.08) | -0.25 (-1.22, 0.72) | -0.23 (-0.61, 0.15) | -0.20 (-0.73, 0.33) | -0.20 (-0.45, 0.05) | -0.19 (-0.69, 0.30) | -0.09 (-0.40, 0.22) | -0.07 (-1.51, 1.38) | SHM | -0.42 (-1.41, 0.57) | . |
| -0.83 (-2.31, 0.65) | -0.79 (-2.55, 0.97) | -0.67 (-2.05, 0.72) | -0.65 (-1.71, 0.42) | -0.62 (-1.75, 0.50) | -0.62 (-1.64, 0.41) | -0.61 (-1.72, 0.50) | -0.51 (-1.55, 0.53) | -0.49 (-2.24, 1.27) | -0.42 (-1.41, 0.57) | TBS-C | . |
| -1.54 (-3.13, 0.05) | -1.51 (-3.36, 0.35) | -1.38 (-2.88, 0.12) | -1.36 (-2.57,-0.15) | -1.33 (-2.35,-0.32) | -1.33 (-2.50,-0.16) | -1.32 (-2.57,-0.08) | -1.22 (-2.41,-0.03) | -1.20 (-3.04, 0.65) | -1.13 (-2.28, 0.02) | -0.71 (-2.23, 0.80) | TAU |

League-table of results of the network meta-analysis excluding studies from Chinese mainland.

Forest-plot of results of pairwise meta-analyses including studies from Chinese mainland.

Forest-plot of results of pairwise meta-analyses excluding studies from Chinese mainland.

### **11.1.3 Splitting NIBS into precise protocols, with consideration of both frequency and targets**

In the consideration of both frequency and targets, the NIBS treatments were classified as follows.

**ECT**

SC ECT-BT, standard-charge ECT, bitemporal

LC ECT-BT, low-charge ECT, bitemporal

**tES**

tDCS-FT, transcranial direct current stimulation, frontal-temporal

tDCS-TT, transcranial direct current stimulation, temporal-temporal

tACS-FT, alpha transcranial alternating current stimulation, frontal-temporal

tRNS-FT, transcranial random noise stimulation, frontal-temporal

**MST**

MST, magnetic seizure therapy

**TMS**

iTBS-F, intermittent theta burst stimulation, frontal

iTBS-C, intermittent theta burst stimulation, cerebellum

cTBS-T, continuous theta burst stimulation, tempoparietal

dTMS-T, deep transcranial magnetic stimulation, tempoparietal

aTMS-F, α-peak-frequency–guided transcranial magnetic stimulation, frontal

LF rTMS-T, low frequency rTMS, tempoparietal

LF rTMS-F, low frequency rTMS, frontal

HF rTMS-T, high frequency rTMS, tempoparietal

HF rTMS-F, high frequency rTMS, frontal

**TAU**

TAU, treatment as usual

**SHM**

SHM, sham therapy

Network plot including studies from Chinese mainland.

Network plot excluding studies from Chinese mainland.

Forest-plot of results of network-meta-analysis including studies from Chinese mainland.

Quantifying heterogeneity / inconsistency:

tau^2 = 0.2281; tau = 0.4776; I^2 = 68.4% [59.2%; 75.6%]

Forest-plot of results of network-meta-analysis excluding studies from Chinese mainland.

Quantifying heterogeneity / inconsistency:

tau^2 = 0.1318; tau = 0.3630; I^2 = 50.2% [27.9%; 65.6%]

| LC ECT-BT | . | . | . | . | . | . | . | . | . | . | -0.61 (-2.10, 0.88) | . | . | . | . | . | . |
| --- | --- | --- | --- | --- | --- | --- | --- | --- | --- | --- | --- | --- | --- | --- | --- | --- | --- |
| -0.10 (-2.02, 1.82) | iTBS-F | . | . | . | . | . | . | . | . | . | . | . | . | -0.71 (-1.76, 0.34) | . | . | . |
| -0.12 (-2.08, 1.83) | -0.02 (-1.55, 1.51) | aTMS-F | . | . | . | . | . | . | . | . | . | . | . | -0.69 (-1.81, 0.43) | . | . | . |
| -0.23 (-1.92, 1.46) | -0.13 (-1.30, 1.05) | -0.11 (-1.34, 1.13) | HF rTMS-F | . | -0.33 (-1.84, 1.19) | -0.37 (-1.33, 0.58) | . | . | . | . | . | . | . | -0.53 (-1.10, 0.03) | . | . | . |
| -0.29 (-1.94, 1.36) | -0.19 (-1.31, 0.93) | -0.17 (-1.35, 1.02) | -0.06 (-0.72, 0.59) | cTBS-T | . | -0.36 (-1.00, 0.28) | . | . | . | . | . | . | . | -0.32 (-0.76, 0.13) | . | . | . |
| -0.30 (-2.02, 1.41) | -0.20 (-1.41, 1.00) | -0.18 (-1.45, 1.09) | -0.08 (-0.85, 0.70) | -0.02 (-0.72, 0.69) | HF rTMS-T | -0.66 (-1.66, 0.35) | . | . | . | . | . | . | . | -0.25 (-0.95, 0.44) | . | . | . |
| -0.35 (-1.97, 1.27) | -0.25 (-1.32, 0.82) | -0.22 (-1.36, 0.91) | -0.12 (-0.68, 0.44) | -0.06 (-0.47, 0.35) | -0.04 (-0.66, 0.57) | LF rTMS-T | -0.20 (-1.42, 1.01) | . | . | . | . | . | . | -0.53 (-0.76,-0.31) | . | . | . |
| -0.40 (-2.04, 1.25) | -0.30 (-1.41, 0.81) | -0.27 (-1.45, 0.90) | -0.17 (-0.81, 0.48) | -0.11 (-0.64, 0.42) | -0.09 (-0.79, 0.61) | -0.05 (-0.46, 0.37) | LF rTMS-F | . | . | . | . | . | . | -0.47 (-0.84,-0.09) | . | . | . |
| -0.44 (-2.68, 1.80) | -0.34 (-2.22, 1.54) | -0.32 (-2.24, 1.60) | -0.21 (-1.86, 1.44) | -0.15 (-1.76, 1.46) | -0.13 (-1.81, 1.54) | -0.09 (-1.67, 1.48) | -0.04 (-1.65, 1.56) | tRNS-FT | . | . | . | . | . | -0.37 (-1.94, 1.19) | . | . | . |
| -0.51 (-2.52, 1.50) | -0.41 (-2.23, 1.41) | -0.39 (-2.25, 1.47) | -0.28 (-1.86, 1.29) | -0.22 (-1.76, 1.31) | -0.21 (-1.81, 1.39) | -0.16 (-1.66, 1.33) | -0.12 (-1.64, 1.41) | -0.07 (-2.23, 2.08) | MST | . | -0.10 (-1.45, 1.26) | . | . | . | . | . | . |
| -0.56 (-2.52, 1.40) | -0.46 (-2.00, 1.07) | -0.44 (-2.03, 1.14) | -0.34 (-1.58, 0.91) | -0.28 (-1.46, 0.91) | -0.26 (-1.53, 1.01) | -0.22 (-1.36, 0.93) | -0.17 (-1.35, 1.01) | -0.13 (-2.05, 1.80) | -0.05 (-1.91, 1.81) | tDCS-TT | . | . | . | -0.25 (-1.37, 0.87) | . | . | . |
| -0.61 (-2.10, 0.88) | -0.51 (-1.72, 0.70) | -0.48 (-1.75, 0.78) | -0.38 (-1.19, 0.43) | -0.32 (-1.04, 0.40) | -0.30 (-1.15, 0.55) | -0.26 (-0.90, 0.38) | -0.21 (-0.92, 0.49) | -0.17 (-1.84, 1.51) | -0.10 (-1.45, 1.26) | -0.04 (-1.32, 1.23) | SC ECT-BT | . | . | -0.21 (-0.81, 0.40) | . | . | -1.33 (-2.50,-0.17) |
| -0.75 (-2.39, 0.90) | -0.65 (-1.75, 0.46) | -0.62 (-1.79, 0.55) | -0.52 (-1.16, 0.12) | -0.46 (-0.98, 0.07) | -0.44 (-1.14, 0.25) | -0.40 (-0.81, 0.01) | -0.35 (-0.86, 0.16) | -0.31 (-1.91, 1.29) | -0.24 (-1.76, 1.29) | -0.18 (-1.36, 1.00) | -0.14 (-0.84, 0.56) | tDCS-FT | . | -0.09 (-0.44, 0.27) | 0.34 (-1.05, 1.72) | . | . |
| -0.75 (-2.98, 1.49) | -0.65 (-2.52, 1.23) | -0.62 (-2.54, 1.29) | -0.52 (-2.16, 1.12) | -0.46 (-2.06, 1.14) | -0.44 (-2.11, 1.22) | -0.40 (-1.97, 1.17) | -0.35 (-1.95, 1.24) | -0.31 (-2.51, 1.89) | -0.24 (-2.38, 1.91) | -0.18 (-2.10, 1.74) | -0.14 (-1.81, 1.53) | -0.00 (-1.59, 1.59) | dTMS-T | -0.07 (-1.62, 1.49) | . | . | . |
| -0.81 (-2.42, 0.79) | -0.71 (-1.76, 0.34) | -0.69 (-1.81, 0.43) | -0.59 (-1.12,-0.05) | -0.53 (-0.91,-0.14) | -0.51 (-1.11, 0.09) | -0.47 (-0.68,-0.25) | -0.42 (-0.78,-0.05) | -0.37 (-1.94, 1.19) | -0.30 (-1.79, 1.18) | -0.25 (-1.37, 0.87) | -0.21 (-0.81, 0.40) | -0.07 (-0.42, 0.29) | -0.07 (-1.62, 1.49) | SHM | -0.13 (-1.02, 0.76) | -0.42 (-1.56, 0.73) | . |
| -0.87 (-2.69, 0.94) | -0.77 (-2.12, 0.57) | -0.75 (-2.15, 0.65) | -0.65 (-1.64, 0.35) | -0.59 (-1.51, 0.34) | -0.57 (-1.60, 0.46) | -0.53 (-1.40, 0.34) | -0.48 (-1.39, 0.44) | -0.44 (-2.21, 1.34) | -0.36 (-2.07, 1.34) | -0.31 (-1.71, 1.09) | -0.27 (-1.30, 0.77) | -0.13 (-1.01, 0.75) | -0.13 (-1.89, 1.64) | -0.06 (-0.90, 0.78) | tACS-FT | . | . |
| -1.23 (-3.20, 0.74) | -1.13 (-2.69, 0.42) | -1.11 (-2.71, 0.49) | -1.00 (-2.27, 0.26) | -0.94 (-2.15, 0.27) | -0.93 (-2.22, 0.36) | -0.89 (-2.05, 0.28) | -0.84 (-2.04, 0.37) | -0.79 (-2.73, 1.14) | -0.72 (-2.60, 1.15) | -0.67 (-2.27, 0.94) | -0.62 (-1.92, 0.67) | -0.49 (-1.69, 0.71) | -0.49 (-2.42, 1.45) | -0.42 (-1.56, 0.73) | -0.36 (-1.78, 1.06) | iTBS-C | . |
| -1.94 (-3.83,-0.05) | -1.84 (-3.52,-0.16) | -1.82 (-3.54,-0.10) | -1.71 (-3.13,-0.30) | -1.65 (-3.02,-0.29) | -1.64 (-3.08,-0.20) | -1.59 (-2.92,-0.27) | -1.55 (-2.91,-0.18) | -1.50 (-3.54, 0.54) | -1.43 (-3.22, 0.36) | -1.38 (-3.10, 0.35) | -1.33 (-2.50,-0.17) | -1.19 (-2.55, 0.16) | -1.19 (-3.23, 0.84) | -1.13 (-2.44, 0.18) | -1.07 (-2.62, 0.49) | -0.71 (-2.45, 1.03) | TAU |

League-table of results of the network meta-analysis including studies from Chinese mainland.

| aTMS-F | . | . | . | . | . | . | . | . | . | . | -0.69 (-1.63, 0.25) | . | . | . |
| --- | --- | --- | --- | --- | --- | --- | --- | --- | --- | --- | --- | --- | --- | --- |
| -0.20 (-1.36, 0.95) | HF rTMS-F | . | -0.33 (-1.71, 1.06) | . | . | . | . | -0.82 (-2.27, 0.62) | . | . | -0.40 (-1.12, 0.31) | . | . | . |
| -0.28 (-1.71, 1.15) | -0.08 (-1.36, 1.20) | tACS-FT | . | . | . | . | . | . | -0.34 (-1.58, 0.91) | . | -0.39 (-1.64, 0.85) | . | . | . |
| -0.28 (-1.36, 0.79) | -0.08 (-0.90, 0.73) | -0.00 (-1.20, 1.20) | HF rTMS-T | . | . | . | . | -0.66 (-1.57, 0.25) | . | . | -0.25 (-0.85, 0.34) | . | . | . |
| -0.32 (-2.03, 1.40) | -0.11 (-1.70, 1.48) | -0.03 (-1.83, 1.77) | -0.03 (-1.56, 1.50) | tRNS-FT | . | . | . | . | . | . | -0.37 (-1.81, 1.06) | . | . | . |
| -0.44 (-1.77, 0.89) | -0.24 (-1.40, 0.93) | -0.16 (-1.60, 1.28) | -0.16 (-1.24, 0.92) | -0.13 (-1.85, 1.60) | tDCS-TT | . | . | . | . | . | -0.25 (-1.19, 0.70) | . | . | . |
| -0.47 (-1.48, 0.53) | -0.27 (-1.04, 0.50) | -0.19 (-1.34, 0.95) | -0.19 (-0.82, 0.44) | -0.16 (-1.64, 1.33) | -0.03 (-1.05, 0.98) | cTBS-T | . | -0.13 (-0.81, 0.56) | . | . | -0.15 (-0.55, 0.26) | . | . | . |
| -0.49 (-1.56, 0.58) | -0.28 (-1.14, 0.57) | -0.21 (-1.41, 0.99) | -0.20 (-0.94, 0.53) | -0.17 (-1.70, 1.36) | -0.05 (-1.13, 1.03) | -0.01 (-0.65, 0.63) | SC ECT-BT | . | . | . | -0.20 (-0.72, 0.32) | . | . | -1.33 (-2.33,-0.34) |
| -0.54 (-1.51, 0.43) | -0.33 (-1.05, 0.38) | -0.25 (-1.37, 0.86) | -0.25 (-0.80, 0.30) | -0.22 (-1.68, 1.24) | -0.09 (-1.07, 0.89) | -0.06 (-0.48, 0.35) | -0.05 (-0.63, 0.53) | LF rTMS-T | . | . | -0.19 (-0.46, 0.08) | . | . | . |
| -0.60 (-1.58, 0.38) | -0.40 (-1.14, 0.35) | -0.32 (-1.40, 0.76) | -0.32 (-0.92, 0.29) | -0.28 (-1.75, 1.19) | -0.16 (-1.15, 0.83) | -0.13 (-0.61, 0.35) | -0.11 (-0.71, 0.49) | -0.06 (-0.46, 0.33) | tDCS-FT | . | -0.09 (-0.39, 0.21) | . | . | . |
| -0.62 (-2.33, 1.08) | -0.42 (-2.00, 1.16) | -0.34 (-2.13, 1.45) | -0.34 (-1.86, 1.18) | -0.31 (-2.34, 1.72) | -0.18 (-1.90, 1.53) | -0.15 (-1.63, 1.33) | -0.14 (-1.66, 1.39) | -0.09 (-1.54, 1.37) | -0.02 (-1.48, 1.44) | dTMS-T | -0.07 (-1.50, 1.36) | . | . | . |
| -0.69 (-1.63, 0.25) | -0.49 (-1.17, 0.19) | -0.41 (-1.49, 0.67) | -0.41 (-0.93, 0.11) | -0.37 (-1.81, 1.06) | -0.25 (-1.19, 0.70) | -0.22 (-0.59, 0.16) | -0.20 (-0.72, 0.32) | -0.15 (-0.41, 0.10) | -0.09 (-0.39, 0.21) | -0.07 (-1.50, 1.36) | SHM | -0.42 (-1.39, 0.55) | -0.77 (-1.79, 0.25) | . |
| -1.11 (-2.46, 0.24) | -0.91 (-2.09, 0.28) | -0.83 (-2.28, 0.63) | -0.83 (-1.93, 0.28) | -0.79 (-2.53, 0.94) | -0.67 (-2.02, 0.69) | -0.64 (-1.68, 0.40) | -0.62 (-1.72, 0.48) | -0.57 (-1.58, 0.43) | -0.51 (-1.53, 0.51) | -0.49 (-2.21, 1.24) | -0.42 (-1.39, 0.55) | iTBS-C | . | . |
| -1.46 (-2.85,-0.08) | -1.26 (-2.48,-0.03) | -1.18 (-2.67, 0.31) | -1.18 (-2.32,-0.03) | -1.15 (-2.91, 0.62) | -1.02 (-2.41, 0.37) | -0.99 (-2.07, 0.10) | -0.97 (-2.12, 0.17) | -0.93 (-1.98, 0.13) | -0.86 (-1.92, 0.20) | -0.84 (-2.59, 0.92) | -0.77 (-1.79, 0.25) | -0.35 (-1.76, 1.06) | LF rTMS-F | . |
| -1.82 (-3.28,-0.36) | -1.62 (-2.93,-0.31) | -1.54 (-3.10, 0.02) | -1.54 (-2.77,-0.30) | -1.51 (-3.33, 0.32) | -1.38 (-2.85, 0.09) | -1.35 (-2.53,-0.17) | -1.33 (-2.33,-0.34) | -1.29 (-2.44,-0.13) | -1.22 (-2.38,-0.06) | -1.20 (-3.01, 0.62) | -1.13 (-2.25,-0.01) | -0.71 (-2.20, 0.77) | -0.36 (-1.88, 1.16) | TAU |

League-table of results of the network meta-analysis excluding studies from Chinese mainland.

Forest-plot of results of pairwise meta-analyses including studies from Chinese mainland.

Forest-plot of results of pairwise meta-analyses excluding studies from Chinese mainland.

## **11.2 Different nodes for precise NIBS protocols (excluding atypical protocols)**

There are four studies from the Chinese mainland that applied 1 Hz rTMS targeting the left dorsolateral prefrontal cortex (Jiang 2017; Quan 2012; Yue 2013; Zhang 2016). However, apart from these trials, very few studies have investigated this specific stimulation protocol. Therefore, we conducted an additional sensitivity analysis excluding these studies to assess differences among various NIBS protocols, with consideration of stimulation frequency, target regions, and their combination.

To assess the potential impact of including studies from Chinese mainland, we presented the sensitivity analysis results both with and without data from these studies.

### **11.2.1 Splitting NIBS into precise protocols, with consideration of frequency**

In the consideration of frequency, the NIBS treatments were classified as follows.

**ECT**

SC ECT, standard-charge ECT

LC ECT, low-charge ECT

**tES**

tDCS, transcranial direct current stimulation

tACS, transcranial alternating current stimulation

tRNS, transcranial random noise stimulation

**MST**

MST, magnetic seizure therapy

**TMS**

iTBS, intermittent theta burst stimulation

cTBS, continuous theta burst stimulation

αTMS, α-peak-frequency–guided transcranial magnetic stimulation

dTMS, deep transcranial magnetic stimulation

LF rTMS, low frequency rTMS

HF rTMS, high frequency rTMS

**TAU**

TAU, treatment as usual

**SHM**

SHM, sham therapy

Network plot including studies from Chinese mainland.

Network plot excluding studies from Chinese mainland.

Forest-plot of results of network-meta-analysis including studies from Chinese mainland.

Quantifying heterogeneity / inconsistency:

tau^2 = 0.2186; tau = 0.4676; I^2 = 66.5% [55.5%; 74.8%]

Forest-plot of results of network-meta-analysis excluding studies from Chinese mainland.

Quantifying heterogeneity / inconsistency:

tau^2 = 0.1336; tau = 0.3655; I^2 = 51% [29.8%; 65.8%]

| LC ECT | . | . | . | . | . | . | -0.61 (-2.10, 0.88) | . | . | . | . | . | . |
| --- | --- | --- | --- | --- | --- | --- | --- | --- | --- | --- | --- | --- | --- |
| -0.12 (-2.08, 1.83) | aTMS | . | . | . | . | . | . | . | . | . | -0.69 (-1.81, 0.43) | . | . |
| -0.29 (-1.95, 1.36) | -0.17 (-1.35, 1.01) | cTBS | . | -0.36 (-1.00, 0.28) | . | . | . | . | . | . | -0.32 (-0.76, 0.13) | . | . |
| -0.30 (-1.96, 1.36) | -0.18 (-1.37, 1.01) | -0.01 (-0.57, 0.56) | HF rTMS | -0.37 (-1.13, 0.38) | . | . | . | . | . | . | -0.41 (-0.86, 0.04) | . | . |
| -0.36 (-1.98, 1.26) | -0.24 (-1.37, 0.90) | -0.07 (-0.47, 0.34) | -0.06 (-0.50, 0.38) | LF rTMS | . | . | . | . | . | . | -0.50 (-0.71,-0.29) | . | . |
| -0.44 (-2.68, 1.80) | -0.32 (-2.24, 1.61) | -0.15 (-1.76, 1.46) | -0.14 (-1.76, 1.48) | -0.08 (-1.65, 1.50) | tRNS | . | . | . | . | . | -0.37 (-1.94, 1.19) | . | . |
| -0.51 (-2.53, 1.50) | -0.39 (-2.25, 1.47) | -0.22 (-1.75, 1.32) | -0.21 (-1.75, 1.33) | -0.15 (-1.65, 1.35) | -0.07 (-2.23, 2.08) | MST | -0.10 (-1.45, 1.26) | . | . | . | . | . | . |
| -0.61 (-2.10, 0.88) | -0.48 (-1.75, 0.79) | -0.31 (-1.03, 0.40) | -0.31 (-1.04, 0.43) | -0.25 (-0.88, 0.39) | -0.17 (-1.84, 1.51) | -0.10 (-1.45, 1.26) | SC ECT | . | . | . | -0.21 (-0.81, 0.40) | . | -1.33 (-2.50,-0.17) |
| -0.62 (-2.40, 1.17) | -0.49 (-1.85, 0.87) | -0.32 (-1.19, 0.54) | -0.32 (-1.20, 0.57) | -0.25 (-1.06, 0.55) | -0.18 (-1.92, 1.57) | -0.11 (-1.78, 1.57) | -0.01 (-0.99, 0.97) | iTBS | . | . | -0.20 (-0.97, 0.58) | . | . |
| -0.73 (-2.37, 0.91) | -0.61 (-1.77, 0.56) | -0.44 (-0.95, 0.08) | -0.43 (-0.97, 0.11) | -0.37 (-0.76, 0.02) | -0.29 (-1.89, 1.31) | -0.22 (-1.74, 1.30) | -0.12 (-0.81, 0.57) | -0.11 (-0.96, 0.73) | tDCS | . | -0.10 (-0.44, 0.24) | 0.34 (-1.05, 1.72) | . |
| -0.75 (-2.98, 1.49) | -0.62 (-2.54, 1.29) | -0.45 (-2.06, 1.15) | -0.45 (-2.06, 1.16) | -0.39 (-1.95, 1.18) | -0.31 (-2.51, 1.90) | -0.24 (-2.38, 1.91) | -0.14 (-1.81, 1.53) | -0.13 (-1.87, 1.61) | -0.02 (-1.61, 1.57) | dTMS | -0.07 (-1.62, 1.49) | . | . |
| -0.81 (-2.42, 0.79) | -0.69 (-1.81, 0.43) | -0.52 (-0.91,-0.13) | -0.51 (-0.93,-0.09) | -0.45 (-0.65,-0.25) | -0.37 (-1.94, 1.19) | -0.30 (-1.79, 1.18) | -0.21 (-0.81, 0.40) | -0.20 (-0.97, 0.58) | -0.08 (-0.42, 0.25) | -0.07 (-1.62, 1.49) | SHM | -0.13 (-1.03, 0.76) | . |
| -0.87 (-2.68, 0.94) | -0.75 (-2.15, 0.65) | -0.58 (-1.50, 0.35) | -0.57 (-1.51, 0.37) | -0.51 (-1.37, 0.35) | -0.43 (-2.21, 1.34) | -0.36 (-2.07, 1.35) | -0.26 (-1.30, 0.77) | -0.25 (-1.40, 0.89) | -0.14 (-1.02, 0.73) | -0.12 (-1.89, 1.64) | -0.06 (-0.90, 0.78) | tACS | . |
| -1.94 (-3.83,-0.05) | -1.82 (-3.54,-0.09) | -1.65 (-3.02,-0.28) | -1.64 (-3.02,-0.26) | -1.58 (-2.91,-0.25) | -1.50 (-3.54, 0.54) | -1.43 (-3.22, 0.36) | -1.33 (-2.50,-0.17) | -1.32 (-2.85, 0.20) | -1.21 (-2.57, 0.14) | -1.19 (-3.23, 0.84) | -1.13 (-2.44, 0.19) | -1.07 (-2.63, 0.49) | TAU |

League-table of results of the network meta-analysis including studies from Chinese mainland.

| aTMS | . | . | . | . | . | . | . | . | -0.69 (-1.63, 0.25) | . | . |
| --- | --- | --- | --- | --- | --- | --- | --- | --- | --- | --- | --- |
| -0.27 (-1.71, 1.16) | tACS | . | . | . | . | . | -0.34 (-1.58, 0.91) | . | -0.39 (-1.64, 0.86) | . | . |
| -0.31 (-1.35, 0.72) | -0.04 (-1.21, 1.13) | HF rTMS | . | . | . | -0.55 (-1.43, 0.32) | . | . | -0.29 (-0.76, 0.19) | . | . |
| -0.32 (-2.04, 1.40) | -0.04 (-1.84, 1.76) | -0.00 (-1.51, 1.50) | tRNS | . | . | . | . | . | -0.37 (-1.82, 1.07) | . | . |
| -0.48 (-1.50, 0.53) | -0.21 (-1.35, 0.94) | -0.17 (-0.74, 0.40) | -0.17 (-1.66, 1.32) | cTBS | . | -0.13 (-0.82, 0.56) | . | . | -0.15 (-0.55, 0.26) | . | . |
| -0.49 (-1.56, 0.59) | -0.21 (-1.42, 0.99) | -0.18 (-0.86, 0.51) | -0.17 (-1.70, 1.36) | -0.00 (-0.65, 0.64) | SC ECT | . | . | . | -0.20 (-0.72, 0.32) | . | -1.33 (-2.33,-0.34) |
| -0.58 (-1.55, 0.39) | -0.31 (-1.42, 0.80) | -0.27 (-0.75, 0.21) | -0.27 (-1.73, 1.20) | -0.10 (-0.51, 0.32) | -0.09 (-0.67, 0.49) | LF rTMS | . | . | -0.13 (-0.40, 0.13) | . | . |
| -0.59 (-1.57, 0.40) | -0.31 (-1.39, 0.77) | -0.27 (-0.80, 0.25) | -0.27 (-1.74, 1.20) | -0.10 (-0.57, 0.37) | -0.10 (-0.69, 0.50) | -0.00 (-0.39, 0.38) | tDCS | . | -0.10 (-0.39, 0.18) | . | . |
| -0.62 (-2.34, 1.09) | -0.35 (-2.14, 1.45) | -0.31 (-1.81, 1.19) | -0.31 (-2.34, 1.72) | -0.14 (-1.62, 1.34) | -0.14 (-1.66, 1.39) | -0.04 (-1.50, 1.41) | -0.04 (-1.50, 1.42) | dTMS | -0.07 (-1.50, 1.37) | . | . |
| -0.69 (-1.63, 0.25) | -0.42 (-1.50, 0.67) | -0.38 (-0.82, 0.06) | -0.37 (-1.82, 1.07) | -0.21 (-0.58, 0.17) | -0.20 (-0.72, 0.32) | -0.11 (-0.36, 0.14) | -0.10 (-0.39, 0.18) | -0.07 (-1.50, 1.37) | SHM | -0.42 (-1.39, 0.56) | . |
| -1.11 (-2.46, 0.24) | -0.84 (-2.29, 0.62) | -0.80 (-1.87, 0.27) | -0.79 (-2.53, 0.95) | -0.63 (-1.67, 0.42) | -0.62 (-1.73, 0.48) | -0.53 (-1.53, 0.48) | -0.52 (-1.54, 0.49) | -0.49 (-2.22, 1.25) | -0.42 (-1.39, 0.56) | iTBS | . |
| -1.82 (-3.29,-0.36) | -1.55 (-3.11, 0.01) | -1.51 (-2.72,-0.30) | -1.51 (-3.33, 0.32) | -1.34 (-2.52,-0.15) | -1.33 (-2.33,-0.34) | -1.24 (-2.39,-0.09) | -1.24 (-2.40,-0.07) | -1.20 (-3.02, 0.62) | -1.13 (-2.26,-0.01) | -0.71 (-2.20, 0.78) | TAU |

League-table of results of the network meta-analysis excluding studies from Chinese mainland.

Forest-plot of results of pairwise meta-analyses including studies from Chinese mainland.

Forest-plot of results of pairwise meta-analyses excluding studies from Chinese mainland.

### **11.2.2 Splitting NIBS into precise protocols, with consideration of targets**

In the consideration of targets, the NIBS treatments were classified as follows.

**ECT**

SC ECT-BT, standard-charge ECT, bitemporal

LC ECT-BT, low-charge ECT, bitemporal

**tES**

tDCS-FT, transcranial direct current stimulation, frontal-temporal

tACS-FT, alpha transcranial alternating current stimulation, frontal-temporal

tDCS-TT, transcranial direct current stimulation, temporal-temporal

tRNS-FT, transcranial random noise stimulation, frontal-temporal

**MST**

MST, magnetic seizure therapy

**TMS**

TBS-F theta burst stimulation, frontal

TBS-C, theta burst stimulation, cerebellum

TBS-T, theta burst stimulation, tempoparietal

dTMS-T, deep transcranial magnetic stimulation, tempoparietal

rTMS-T, low frequency rTMS, tempoparietal

rTMS-F, low frequency rTMS, frontal

**TAU**

TAU, treatment as usual

**SHM**

SHM, sham therapy

Network plot including studies from Chinese mainland.

Network plot excluding studies from Chinese mainland.

Forest-plot of results of network-meta-analysis including studies from Chinese mainland.

Quantifying heterogeneity / inconsistency:

tau^2 = 0.2168; tau = 0.4656; I^2 = 66.6% [56.4%; 74.5%]

Forest-plot of results of network-meta-analysis excluding studies from Chinese mainland.

Quantifying heterogeneity / inconsistency:

tau^2 = 0.1431; tau = 0.3783; I^2 = 53.1% [32.8%; 67.3%]

| LC ECT-BT | . | . | . | . | . | . | . | -0.61 (-2.08, 0.87) | . | . | . | . | . | . |
| --- | --- | --- | --- | --- | --- | --- | --- | --- | --- | --- | --- | --- | --- | --- |
| -0.10 (-1.99, 1.79) | TBS-F | . | . | . | . | . | . | . | . | . | -0.71 (-1.74, 0.32) | . | . | . |
| -0.28 (-1.92, 1.35) | -0.19 (-1.28, 0.91) | TBS-T | -0.36 (-0.98, 0.26) | . | . | . | . | . | . | . | -0.32 (-0.76, 0.12) | . | . | . |
| -0.34 (-1.94, 1.27) | -0.24 (-1.29, 0.81) | -0.05 (-0.45, 0.35) | rTMS-T | 0.03 (-0.70, 0.75) | . | . | . | . | . | . | -0.52 (-0.73,-0.31) | . | . | . |
| -0.35 (-1.98, 1.28) | -0.25 (-1.34, 0.83) | -0.07 (-0.58, 0.44) | -0.01 (-0.40, 0.37) | rTMS-F | . | . | . | . | . | . | -0.49 (-0.85,-0.13) | . | . | . |
| -0.44 (-2.66, 1.78) | -0.34 (-2.20, 1.52) | -0.15 (-1.75, 1.44) | -0.10 (-1.66, 1.46) | -0.09 (-1.67, 1.50) | tRNS-FT | . | . | . | . | . | -0.37 (-1.92, 1.17) | . | . | . |
| -0.51 (-2.50, 1.48) | -0.41 (-2.20, 1.38) | -0.23 (-1.74, 1.29) | -0.17 (-1.65, 1.31) | -0.16 (-1.67, 1.35) | -0.07 (-2.20, 2.06) | MST | . | -0.10 (-1.44, 1.24) | . | . | . | . | . | . |
| -0.56 (-2.50, 1.37) | -0.46 (-1.97, 1.04) | -0.28 (-1.45, 0.89) | -0.23 (-1.35, 0.90) | -0.21 (-1.37, 0.94) | -0.13 (-2.03, 1.78) | -0.05 (-1.89, 1.78) | tDCS-TT | . | . | . | -0.25 (-1.35, 0.86) | . | . | . |
| -0.61 (-2.08, 0.87) | -0.51 (-1.70, 0.68) | -0.32 (-1.03, 0.38) | -0.27 (-0.90, 0.36) | -0.26 (-0.94, 0.43) | -0.17 (-1.83, 1.49) | -0.10 (-1.44, 1.24) | -0.04 (-1.30, 1.21) | SC ECT-BT | . | . | -0.21 (-0.80, 0.39) | . | . | -1.33 (-2.48,-0.19) |
| -0.75 (-2.37, 0.88) | -0.65 (-1.73, 0.44) | -0.46 (-0.98, 0.05) | -0.41 (-0.81,-0.01) | -0.39 (-0.88, 0.10) | -0.31 (-1.89, 1.28) | -0.23 (-1.74, 1.27) | -0.18 (-1.34, 0.98) | -0.14 (-0.83, 0.55) | tDCS-FT | . | -0.09 (-0.44, 0.26) | 0.34 (-1.03, 1.70) | . | . |
| -0.75 (-2.96, 1.47) | -0.65 (-2.50, 1.20) | -0.46 (-2.05, 1.12) | -0.41 (-1.96, 1.14) | -0.40 (-1.97, 1.18) | -0.31 (-2.49, 1.88) | -0.24 (-2.36, 1.89) | -0.18 (-2.08, 1.71) | -0.14 (-1.79, 1.51) | -0.00 (-1.58, 1.58) | dTMS-T | -0.07 (-1.61, 1.47) | . | . | . |
| -0.81 (-2.40, 0.78) | -0.71 (-1.74, 0.32) | -0.53 (-0.91,-0.15) | -0.48 (-0.68,-0.27) | -0.46 (-0.81,-0.12) | -0.37 (-1.92, 1.17) | -0.30 (-1.77, 1.16) | -0.25 (-1.35, 0.86) | -0.21 (-0.80, 0.39) | -0.07 (-0.42, 0.28) | -0.07 (-1.61, 1.47) | SHM | -0.13 (-1.01, 0.75) | -0.42 (-1.55, 0.71) | . |
| -0.88 (-2.67, 0.92) | -0.78 (-2.10, 0.54) | -0.59 (-1.50, 0.32) | -0.54 (-1.39, 0.31) | -0.53 (-1.42, 0.37) | -0.44 (-2.19, 1.32) | -0.37 (-2.05, 1.32) | -0.31 (-1.69, 1.07) | -0.27 (-1.29, 0.75) | -0.13 (-1.00, 0.73) | -0.13 (-1.88, 1.62) | -0.06 (-0.89, 0.77) | tACS-FT | . | . |
| -1.23 (-3.18, 0.72) | -1.13 (-2.66, 0.39) | -0.95 (-2.14, 0.24) | -0.89 (-2.04, 0.25) | -0.88 (-2.06, 0.30) | -0.79 (-2.71, 1.12) | -0.72 (-2.57, 1.13) | -0.67 (-2.25, 0.91) | -0.62 (-1.90, 0.65) | -0.49 (-1.67, 0.69) | -0.49 (-2.39, 1.42) | -0.42 (-1.55, 0.71) | -0.36 (-1.75, 1.04) | TBS-C | . |
| -1.94 (-3.81,-0.07) | -1.84 (-3.49,-0.19) | -1.66 (-3.00,-0.31) | -1.60 (-2.91,-0.30) | -1.59 (-2.93,-0.25) | -1.50 (-3.52, 0.51) | -1.43 (-3.19, 0.33) | -1.38 (-3.08, 0.32) | -1.33 (-2.48,-0.19) | -1.20 (-2.53, 0.14) | -1.19 (-3.20, 0.81) | -1.13 (-2.42, 0.16) | -1.06 (-2.60, 0.47) | -0.71 (-2.42, 1.00) | TAU |

League-table of results of the network meta-analysis including studies from Chinese mainland.

| tACS-FT | . | . | . | . | . | . | -0.34 (-1.60, 0.92) | . | -0.39 (-1.65, 0.87) | . | . |
| --- | --- | --- | --- | --- | --- | --- | --- | --- | --- | --- | --- |
| -0.03 (-1.85, 1.79) | tRNS-FT | . | . | . | . | . | . | . | -0.37 (-1.83, 1.08) | . | . |
| -0.16 (-1.62, 1.30) | -0.13 (-1.87, 1.62) | tDCS-TT | . | . | . | . | . | . | -0.25 (-1.22, 0.72) | . | . |
| -0.18 (-1.34, 0.98) | -0.15 (-1.65, 1.36) | -0.02 (-1.06, 1.02) | TBS-T | . | -0.13 (-0.83, 0.57) | . | . | . | -0.15 (-0.56, 0.27) | . | . |
| -0.21 (-1.42, 1.01) | -0.17 (-1.72, 1.38) | -0.05 (-1.15, 1.06) | -0.03 (-0.68, 0.63) | SC ECT-BT | . | . | . | . | -0.20 (-0.73, 0.33) | . | -1.33 (-2.35,-0.32) |
| -0.21 (-1.33, 0.92) | -0.17 (-1.65, 1.30) | -0.05 (-1.05, 0.95) | -0.03 (-0.45, 0.39) | -0.00 (-0.59, 0.58) | rTMS-T | 0.22 (-1.07, 1.52) | . | . | -0.21 (-0.47, 0.05) | . | . |
| -0.22 (-1.42, 0.99) | -0.18 (-1.72, 1.35) | -0.06 (-1.14, 1.03) | -0.04 (-0.66, 0.59) | -0.01 (-0.73, 0.71) | -0.01 (-0.55, 0.53) | rTMS-F | . | . | -0.17 (-0.67, 0.34) | . | . |
| -0.32 (-1.42, 0.78) | -0.29 (-1.77, 1.20) | -0.16 (-1.18, 0.86) | -0.14 (-0.63, 0.35) | -0.11 (-0.73, 0.50) | -0.11 (-0.51, 0.29) | -0.10 (-0.69, 0.48) | tDCS-FT | . | -0.09 (-0.40, 0.22) | . | . |
| -0.34 (-2.16, 1.47) | -0.31 (-2.36, 1.74) | -0.18 (-1.92, 1.56) | -0.16 (-1.66, 1.33) | -0.14 (-1.68, 1.40) | -0.13 (-1.60, 1.33) | -0.13 (-1.65, 1.40) | -0.02 (-1.50, 1.45) | dTMS-T | -0.07 (-1.51, 1.38) | . | . |
| -0.41 (-1.51, 0.69) | -0.37 (-1.83, 1.08) | -0.25 (-1.22, 0.72) | -0.23 (-0.61, 0.15) | -0.20 (-0.73, 0.33) | -0.20 (-0.45, 0.05) | -0.19 (-0.69, 0.30) | -0.09 (-0.40, 0.22) | -0.07 (-1.51, 1.38) | SHM | -0.42 (-1.41, 0.57) | . |
| -0.83 (-2.31, 0.65) | -0.79 (-2.55, 0.97) | -0.67 (-2.05, 0.72) | -0.65 (-1.71, 0.42) | -0.62 (-1.75, 0.50) | -0.62 (-1.64, 0.41) | -0.61 (-1.72, 0.50) | -0.51 (-1.55, 0.53) | -0.49 (-2.24, 1.27) | -0.42 (-1.41, 0.57) | TBS-C | . |
| -1.54 (-3.13, 0.05) | -1.51 (-3.36, 0.35) | -1.38 (-2.88, 0.12) | -1.36 (-2.57,-0.15) | -1.33 (-2.35,-0.32) | -1.33 (-2.50,-0.16) | -1.32 (-2.57,-0.08) | -1.22 (-2.41,-0.03) | -1.20 (-3.04, 0.65) | -1.13 (-2.28, 0.02) | -0.71 (-2.23, 0.80) | TAU |

League-table of results of the network meta-analysis excluding studies from Chinese mainland.

Forest-plot of results of pairwise meta-analyses including studies from Chinese mainland.

Forest-plot of results of pairwise meta-analyses excluding studies from Chinese mainland.

### **11.2.3 Splitting NIBS into precise protocols, with consideration of both frequency and targets**

In the consideration of both frequency and targets, the NIBS treatments were classified as follows.

**ECT**

SC ECT-BT, standard-charge ECT, bitemporal

LC ECT-BT, low-charge ECT, bitemporal

**tES**

tDCS-FT, transcranial direct current stimulation, frontal-temporal

tDCS-TT, transcranial direct current stimulation, temporal-temporal

tACS-FT, alpha transcranial alternating current stimulation, frontal-temporal

tRNS-FT, transcranial random noise stimulation, frontal-temporal

**MST**

MST, magnetic seizure therapy

**TMS**

iTBS-F, intermittent theta burst stimulation, frontal

iTBS-C, intermittent theta burst stimulation, cerebellum

cTBS-T, continuous theta burst stimulation, tempoparietal

dTMS-T, deep transcranial magnetic stimulation, tempoparietal

aTMS-F, α-peak-frequency–guided transcranial magnetic stimulation, frontal

LF rTMS-T, low frequency rTMS, tempoparietal

LF rTMS-F, low frequency rTMS, frontal

HF rTMS-T, high frequency rTMS, tempoparietal

HF rTMS-F, high frequency rTMS, frontal

**TAU**

TAU, treatment as usual

**SHM**

SHM, sham therapy

Network plot including studies from Chinese mainland.

Network plot excluding studies from Chinese mainland.

Forest-plot of results of network-meta-analysis including studies from Chinese mainland.

Quantifying heterogeneity / inconsistency:

tau^2 = 0.2327; tau = 0.4824; I^2 = 67.6% [57.7%; 75.2%]

Forest-plot of results of network-meta-analysis excluding studies from Chinese mainland.

Quantifying heterogeneity / inconsistency:

tau^2 = 0.1318; tau = 0.3630; I^2 = 50.2% [27.9%; 65.6%]

| LC ECT-BT | . | . | . | . | . | . | . | . | . | . | -0.61 (-2.10, 0.89) | . | . | . | . | . | . |
| --- | --- | --- | --- | --- | --- | --- | --- | --- | --- | --- | --- | --- | --- | --- | --- | --- | --- |
| -0.10 (-2.03, 1.83) | iTBS-F | . | . | . | . | . | . | . | . | . | . | . | . | -0.71 (-1.77, 0.34) | . | . | . |
| -0.12 (-2.09, 1.84) | -0.02 (-1.57, 1.52) | aTMS-F | . | . | . | . | . | . | . | . | . | . | . | -0.69 (-1.81, 0.43) | . | . | . |
| -0.23 (-1.93, 1.47) | -0.13 (-1.31, 1.06) | -0.10 (-1.35, 1.14) | HF rTMS-F | . | -0.33 (-1.85, 1.19) | -0.37 (-1.33, 0.59) | . | . | . | . | . | . | . | -0.53 (-1.10, 0.03) | . | . | . |
| -0.29 (-1.95, 1.37) | -0.19 (-1.32, 0.94) | -0.17 (-1.36, 1.02) | -0.06 (-0.72, 0.60) | cTBS-T | . | -0.36 (-1.00, 0.28) | . | . | . | . | . | . | . | -0.32 (-0.77, 0.13) | . | . | . |
| -0.30 (-2.02, 1.42) | -0.20 (-1.42, 1.01) | -0.18 (-1.46, 1.10) | -0.08 (-0.85, 0.70) | -0.01 (-0.73, 0.70) | HF rTMS-T | -0.66 (-1.67, 0.35) | . | . | . | . | . | . | . | -0.25 (-0.96, 0.45) | . | . | . |
| -0.35 (-1.97, 1.28) | -0.25 (-1.33, 0.83) | -0.23 (-1.37, 0.92) | -0.12 (-0.68, 0.44) | -0.06 (-0.47, 0.35) | -0.04 (-0.66, 0.57) | LF rTMS-T | . | -0.20 (-1.42, 1.02) | . | . | . | . | . | -0.53 (-0.76,-0.31) | . | . | . |
| -0.44 (-2.69, 1.81) | -0.34 (-2.23, 1.55) | -0.32 (-2.24, 1.61) | -0.21 (-1.87, 1.45) | -0.15 (-1.77, 1.47) | -0.14 (-1.81, 1.54) | -0.09 (-1.67, 1.49) | tRNS-FT | . | . | . | . | . | . | -0.37 (-1.94, 1.19) | . | . | . |
| -0.45 (-2.15, 1.24) | -0.35 (-1.53, 0.82) | -0.33 (-1.57, 0.90) | -0.23 (-0.97, 0.51) | -0.17 (-0.81, 0.48) | -0.15 (-0.94, 0.64) | -0.11 (-0.65, 0.44) | -0.02 (-1.67, 1.63) | LF rTMS-F | . | . | . | . | . | -0.45 (-0.98, 0.08) | . | . | . |
| -0.51 (-2.53, 1.51) | -0.41 (-2.24, 1.42) | -0.39 (-2.26, 1.48) | -0.28 (-1.87, 1.30) | -0.22 (-1.76, 1.32) | -0.21 (-1.82, 1.40) | -0.16 (-1.67, 1.34) | -0.07 (-2.24, 2.09) | -0.06 (-1.63, 1.52) | MST | . | -0.10 (-1.46, 1.27) | . | . | . | . | . | . |
| -0.56 (-2.53, 1.41) | -0.46 (-2.01, 1.08) | -0.44 (-2.04, 1.15) | -0.34 (-1.59, 0.92) | -0.28 (-1.47, 0.92) | -0.26 (-1.54, 1.02) | -0.22 (-1.37, 0.94) | -0.13 (-2.06, 1.81) | -0.11 (-1.35, 1.13) | -0.05 (-1.93, 1.82) | tDCS-TT | . | . | . | -0.25 (-1.38, 0.88) | . | . | . |
| -0.61 (-2.10, 0.89) | -0.51 (-1.73, 0.71) | -0.48 (-1.76, 0.79) | -0.38 (-1.19, 0.43) | -0.32 (-1.04, 0.40) | -0.30 (-1.16, 0.55) | -0.26 (-0.90, 0.38) | -0.17 (-1.85, 1.51) | -0.15 (-0.95, 0.64) | -0.10 (-1.46, 1.27) | -0.04 (-1.33, 1.24) | SC ECT-BT | . | . | -0.21 (-0.81, 0.40) | . | . | -1.33 (-2.51,-0.16) |
| -0.75 (-2.40, 0.91) | -0.65 (-1.76, 0.47) | -0.62 (-1.80, 0.56) | -0.52 (-1.16, 0.13) | -0.46 (-0.99, 0.07) | -0.44 (-1.14, 0.26) | -0.40 (-0.81, 0.02) | -0.31 (-1.92, 1.30) | -0.29 (-0.92, 0.33) | -0.24 (-1.77, 1.30) | -0.18 (-1.37, 1.00) | -0.14 (-0.84, 0.56) | tDCS-FT | . | -0.09 (-0.45, 0.27) | 0.34 (-1.05, 1.73) | . | . |
| -0.75 (-2.99, 1.50) | -0.65 (-2.53, 1.24) | -0.62 (-2.55, 1.30) | -0.52 (-2.17, 1.13) | -0.46 (-2.07, 1.15) | -0.44 (-2.12, 1.23) | -0.40 (-1.97, 1.18) | -0.31 (-2.52, 1.90) | -0.29 (-1.93, 1.35) | -0.24 (-2.39, 1.92) | -0.18 (-2.11, 1.74) | -0.14 (-1.81, 1.53) | -0.00 (-1.60, 1.60) | dTMS-T | -0.07 (-1.63, 1.49) | . | . | . |
| -0.81 (-2.43, 0.80) | -0.71 (-1.77, 0.34) | -0.69 (-1.81, 0.43) | -0.59 (-1.12,-0.05) | -0.52 (-0.91,-0.14) | -0.51 (-1.11, 0.09) | -0.47 (-0.68,-0.25) | -0.37 (-1.94, 1.19) | -0.36 (-0.87, 0.15) | -0.30 (-1.79, 1.19) | -0.25 (-1.38, 0.88) | -0.21 (-0.81, 0.40) | -0.07 (-0.42, 0.29) | -0.07 (-1.63, 1.49) | SHM | -0.13 (-1.03, 0.77) | -0.42 (-1.57, 0.74) | . |
| -0.87 (-2.69, 0.95) | -0.77 (-2.13, 0.58) | -0.75 (-2.16, 0.65) | -0.65 (-1.65, 0.35) | -0.59 (-1.52, 0.34) | -0.57 (-1.61, 0.47) | -0.53 (-1.40, 0.35) | -0.44 (-2.22, 1.35) | -0.42 (-1.41, 0.57) | -0.36 (-2.08, 1.35) | -0.31 (-1.72, 1.10) | -0.27 (-1.31, 0.77) | -0.13 (-1.01, 0.75) | -0.13 (-1.90, 1.65) | -0.06 (-0.91, 0.78) | tACS-FT | . | . |
| -1.23 (-3.22, 0.75) | -1.13 (-2.70, 0.43) | -1.11 (-2.72, 0.50) | -1.00 (-2.28, 0.27) | -0.94 (-2.16, 0.27) | -0.93 (-2.23, 0.37) | -0.88 (-2.06, 0.29) | -0.79 (-2.74, 1.15) | -0.78 (-2.04, 0.49) | -0.72 (-2.61, 1.16) | -0.67 (-2.28, 0.95) | -0.62 (-1.93, 0.68) | -0.49 (-1.69, 0.72) | -0.49 (-2.43, 1.45) | -0.42 (-1.57, 0.74) | -0.36 (-1.79, 1.07) | iTBS-C | . |
| -1.94 (-3.84,-0.04) | -1.84 (-3.53,-0.15) | -1.82 (-3.55,-0.08) | -1.71 (-3.14,-0.29) | -1.65 (-3.03,-0.28) | -1.64 (-3.09,-0.19) | -1.59 (-2.93,-0.26) | -1.50 (-3.55, 0.55) | -1.49 (-2.90,-0.07) | -1.43 (-3.23, 0.37) | -1.38 (-3.12, 0.36) | -1.33 (-2.51,-0.16) | -1.19 (-2.56, 0.17) | -1.19 (-3.24, 0.85) | -1.13 (-2.45, 0.19) | -1.07 (-2.63, 0.50) | -0.71 (-2.46, 1.04) | TAU |

League-table of results of the network meta-analysis including studies from Chinese mainland.

| aTMS-F | . | . | . | . | . | . | . | . | . | . | -0.69 (-1.63, 0.25) | . | . | . |
| --- | --- | --- | --- | --- | --- | --- | --- | --- | --- | --- | --- | --- | --- | --- |
| -0.20 (-1.36, 0.95) | HF rTMS-F | . | -0.33 (-1.71, 1.06) | . | . | . | . | -0.82 (-2.27, 0.62) | . | . | -0.40 (-1.12, 0.31) | . | . | . |
| -0.28 (-1.71, 1.15) | -0.08 (-1.36, 1.20) | tACS-FT | . | . | . | . | . | . | -0.34 (-1.58, 0.91) | . | -0.39 (-1.64, 0.85) | . | . | . |
| -0.28 (-1.36, 0.79) | -0.08 (-0.90, 0.73) | -0.00 (-1.20, 1.20) | HF rTMS-T | . | . | . | . | -0.66 (-1.57, 0.25) | . | . | -0.25 (-0.85, 0.34) | . | . | . |
| -0.32 (-2.03, 1.40) | -0.11 (-1.70, 1.48) | -0.03 (-1.83, 1.77) | -0.03 (-1.56, 1.50) | tRNS-FT | . | . | . | . | . | . | -0.37 (-1.81, 1.06) | . | . | . |
| -0.44 (-1.77, 0.89) | -0.24 (-1.40, 0.93) | -0.16 (-1.60, 1.28) | -0.16 (-1.24, 0.92) | -0.13 (-1.85, 1.60) | tDCS-TT | . | . | . | . | . | -0.25 (-1.19, 0.70) | . | . | . |
| -0.47 (-1.48, 0.53) | -0.27 (-1.04, 0.50) | -0.19 (-1.34, 0.95) | -0.19 (-0.82, 0.44) | -0.16 (-1.64, 1.33) | -0.03 (-1.05, 0.98) | cTBS-T | . | -0.13 (-0.81, 0.56) | . | . | -0.15 (-0.55, 0.26) | . | . | . |
| -0.49 (-1.56, 0.58) | -0.28 (-1.14, 0.57) | -0.21 (-1.41, 0.99) | -0.20 (-0.94, 0.53) | -0.17 (-1.70, 1.36) | -0.05 (-1.13, 1.03) | -0.01 (-0.65, 0.63) | SC ECT-BT | . | . | . | -0.20 (-0.72, 0.32) | . | . | -1.33 (-2.33,-0.34) |
| -0.54 (-1.51, 0.43) | -0.33 (-1.05, 0.38) | -0.25 (-1.37, 0.86) | -0.25 (-0.80, 0.30) | -0.22 (-1.68, 1.24) | -0.09 (-1.07, 0.89) | -0.06 (-0.48, 0.35) | -0.05 (-0.63, 0.53) | LF rTMS-T | . | . | -0.19 (-0.46, 0.08) | . | . | . |
| -0.60 (-1.58, 0.38) | -0.40 (-1.14, 0.35) | -0.32 (-1.40, 0.76) | -0.32 (-0.92, 0.29) | -0.28 (-1.75, 1.19) | -0.16 (-1.15, 0.83) | -0.13 (-0.61, 0.35) | -0.11 (-0.71, 0.49) | -0.06 (-0.46, 0.33) | tDCS-FT | . | -0.09 (-0.39, 0.21) | . | . | . |
| -0.62 (-2.33, 1.08) | -0.42 (-2.00, 1.16) | -0.34 (-2.13, 1.45) | -0.34 (-1.86, 1.18) | -0.31 (-2.34, 1.72) | -0.18 (-1.90, 1.53) | -0.15 (-1.63, 1.33) | -0.14 (-1.66, 1.39) | -0.09 (-1.54, 1.37) | -0.02 (-1.48, 1.44) | dTMS-T | -0.07 (-1.50, 1.36) | . | . | . |
| -0.69 (-1.63, 0.25) | -0.49 (-1.17, 0.19) | -0.41 (-1.49, 0.67) | -0.41 (-0.93, 0.11) | -0.37 (-1.81, 1.06) | -0.25 (-1.19, 0.70) | -0.22 (-0.59, 0.16) | -0.20 (-0.72, 0.32) | -0.15 (-0.41, 0.10) | -0.09 (-0.39, 0.21) | -0.07 (-1.50, 1.36) | SHM | -0.42 (-1.39, 0.55) | -0.77 (-1.79, 0.25) | . |
| -1.11 (-2.46, 0.24) | -0.91 (-2.09, 0.28) | -0.83 (-2.28, 0.63) | -0.83 (-1.93, 0.28) | -0.79 (-2.53, 0.94) | -0.67 (-2.02, 0.69) | -0.64 (-1.68, 0.40) | -0.62 (-1.72, 0.48) | -0.57 (-1.58, 0.43) | -0.51 (-1.53, 0.51) | -0.49 (-2.21, 1.24) | -0.42 (-1.39, 0.55) | iTBS-C | . | . |
| -1.46 (-2.85,-0.08) | -1.26 (-2.48,-0.03) | -1.18 (-2.67, 0.31) | -1.18 (-2.32,-0.03) | -1.15 (-2.91, 0.62) | -1.02 (-2.41, 0.37) | -0.99 (-2.07, 0.10) | -0.97 (-2.12, 0.17) | -0.93 (-1.98, 0.13) | -0.86 (-1.92, 0.20) | -0.84 (-2.59, 0.92) | -0.77 (-1.79, 0.25) | -0.35 (-1.76, 1.06) | LF rTMS-F | . |
| -1.82 (-3.28,-0.36) | -1.62 (-2.93,-0.31) | -1.54 (-3.10, 0.02) | -1.54 (-2.77,-0.30) | -1.51 (-3.33, 0.32) | -1.38 (-2.85, 0.09) | -1.35 (-2.53,-0.17) | -1.33 (-2.33,-0.34) | -1.29 (-2.44,-0.13) | -1.22 (-2.38,-0.06) | -1.20 (-3.01, 0.62) | -1.13 (-2.25,-0.01) | -0.71 (-2.20, 0.77) | -0.36 (-1.88, 1.16) | TAU |

League-table of results of the network meta-analysis excluding studies from Chinese mainland.

Forest-plot of results of pairwise meta-analyses including studies from Chinese mainland.

Forest-plot of results of pairwise meta-analyses excluding studies from Chinese mainland.

## **11.3 Double blind studies only**

Quantifying heterogeneity / inconsistency:

tau^2 = 0.1657; tau = 0.4071; I^2 = 63% [51.1%; 72.0%]

| TMS | . | -0.44 (-0.60,-0.29) | . |
| --- | --- | --- | --- |
| -0.43 (-0.77,-0.10) | tES | -0.01 (-0.31, 0.29) | . |
| -0.44 (-0.60,-0.29) | -0.01 (-0.31, 0.29) | SHM | -0.25 (-1.01, 0.51) |
| -0.69 (-1.47, 0.09) | -0.26 (-1.08, 0.56) | -0.25 (-1.01, 0.51) | ECT |

## **11.4 Excluding studies at high risk of bias**

Quantifying heterogeneity / inconsistency:

tau^2 = 0.2058; tau = 0.4537; I^2 = 68.2% [54.9%; 77.6%]

| tES | . | -0.47 (-0.92,-0.01) | . | . | . |
| --- | --- | --- | --- | --- | --- |
| -0.04 (-0.54, 0.46) | TMS | -0.43 (-0.63,-0.23) | . | . | . |
| -0.47 (-0.92,-0.01) | -0.43 (-0.63,-0.23) | SHM | . | -0.11 (-1.21, 0.99) | . |
| -0.48 (-2.27, 1.30) | -0.44 (-2.18, 1.29) | -0.01 (-1.74, 1.71) | MST | -0.10 (-1.42, 1.23) | . |
| -0.58 (-1.77, 0.61) | -0.54 (-1.66, 0.58) | -0.11 (-1.21, 0.99) | -0.10 (-1.42, 1.23) | ECT | -1.33 (-2.46,-0.21) |
| -1.91 (-3.56,-0.27) | -1.87 (-3.46,-0.28) | -1.44 (-3.02, 0.13) | -1.43 (-3.17, 0.31) | -1.33 (-2.46,-0.21) | TAU |

## **11.5 Excluding studies with implied randomization**

Quantifying heterogeneity / inconsistency:

tau^2 = 0.1958; tau = 0.4425; I^2 = 66.3% [56.3%; 74.0%]

| TMS | . | . | . | -0.48 (-0.64,-0.32) | . |
| --- | --- | --- | --- | --- | --- |
| -0.18 (-1.62, 1.26) | MST | -0.10 (-1.41, 1.21) | . | . | . |
| -0.28 (-0.87, 0.32) | -0.10 (-1.41, 1.21) | ECT | . | -0.20 (-0.78, 0.37) | -1.33 (-2.44,-0.22) |
| -0.40 (-0.74,-0.06) | -0.22 (-1.68, 1.24) | -0.13 (-0.78, 0.52) | tES | -0.08 (-0.38, 0.22) | . |
| -0.48 (-0.64,-0.32) | -0.30 (-1.73, 1.13) | -0.20 (-0.78, 0.37) | -0.08 (-0.38, 0.22) | SHM | . |
| -1.61 (-2.87,-0.35) | -1.43 (-3.15, 0.29) | -1.33 (-2.44,-0.22) | -1.21 (-2.49, 0.08) | -1.13 (-2.38, 0.12) | TAU |

## **11.6 Excluding studies that did not use operationalized diagnostic criteria**

Quantifying heterogeneity / inconsistency:

tau^2 = 0.1920; tau = 0.4382; I^2 = 65.5% [54.8%; 73.7%]

| TMS | . | . | . | -0.49 (-0.65,-0.32) | . |
| --- | --- | --- | --- | --- | --- |
| -0.19 (-1.62, 1.25) | MST | -0.10 (-1.40, 1.21) | . | . | . |
| -0.28 (-0.88, 0.31) | -0.10 (-1.40, 1.21) | ECT | . | -0.20 (-0.78, 0.37) | -1.33 (-2.44,-0.23) |
| -0.41 (-0.75,-0.07) | -0.22 (-1.68, 1.23) | -0.13 (-0.77, 0.52) | tES | -0.08 (-0.38, 0.22) | . |
| -0.49 (-0.65,-0.32) | -0.30 (-1.73, 1.12) | -0.20 (-0.78, 0.37) | -0.08 (-0.38, 0.22) | SHM | . |
| -1.62 (-2.87,-0.36) | -1.43 (-3.14, 0.28) | -1.33 (-2.44,-0.23) | -1.21 (-2.49, 0.07) | -1.13 (-2.37, 0.12) | TAU |

## **11.7 Excluding studies that assumed the administration of antipsychotics**

Quantifying heterogeneity / inconsistency:

tau^2 = 0.1828; tau = 0.4276; I^2 = 64.8% [54.0%; 73.0%]

| TMS | . | . | . | -0.50 (-0.66,-0.35) | . |
| --- | --- | --- | --- | --- | --- |
| -0.20 (-1.62, 1.21) | MST | -0.10 (-1.39, 1.19) | . | . | . |
| -0.30 (-0.89, 0.29) | -0.10 (-1.39, 1.19) | ECT | . | -0.20 (-0.77, 0.36) | -1.33 (-2.42,-0.25) |
| -0.38 (-0.72,-0.03) | -0.17 (-1.61, 1.27) | -0.08 (-0.72, 0.57) | tES | -0.13 (-0.44, 0.18) | . |
| -0.50 (-0.66,-0.35) | -0.30 (-1.71, 1.11) | -0.20 (-0.77, 0.36) | -0.13 (-0.44, 0.18) | SHM | . |
| -1.63 (-2.87,-0.40) | -1.43 (-3.12, 0.26) | -1.33 (-2.42,-0.25) | -1.26 (-2.52, 0.01) | -1.13 (-2.36, 0.10) | TAU |

## **11.8 Studies in treatment-resistant positive symptom domains**

Quantifying heterogeneity / inconsistency:

tau^2 = 0.2766; tau = 0.5259; I^2 = 77.1% [66.4%; 84.5%]

| TMS | . | . | . | -0.58 (-0.83,-0.33) | . |
| --- | --- | --- | --- | --- | --- |
| -0.21 (-1.85, 1.43) | tES | . | . | -0.37 (-2.00, 1.25) | . |
| -0.32 (-2.03, 1.38) | -0.12 (-2.46, 2.22) | MST | -0.10 (-1.52, 1.33) | . | . |
| -0.42 (-1.37, 0.52) | -0.21 (-2.07, 1.65) | -0.10 (-1.52, 1.33) | ECT | -0.16 (-1.07, 0.75) | -1.33 (-2.58,-0.09) |
| -0.58 (-0.83,-0.33) | -0.37 (-2.00, 1.25) | -0.26 (-1.95, 1.43) | -0.16 (-1.07, 0.75) | SHM | . |
| -1.75 (-3.32,-0.19) | -1.55 (-3.78, 0.69) | -1.43 (-3.32, 0.46) | -1.33 (-2.58,-0.09) | -1.17 (-2.71, 0.37) | TAU |

## **11.9 Studies with rating scales of positive symptoms used for the primary outcome**

Quantifying heterogeneity / inconsistency:

tau^2 = 0.1917; tau = 0.4378; I^2 = 65.6% [53.0%; 74.9%]

| TMS | . | . | . | -0.49 (-0.67,-0.30) |
| --- | --- | --- | --- | --- |
| -0.19 (-1.62, 1.25) | MST | -0.10 (-1.40, 1.21) | . | . |
| -0.28 (-0.88, 0.32) | -0.10 (-1.40, 1.21) | ECT | . | -0.20 (-0.78, 0.37) |
| -0.36 (-0.77, 0.04) | -0.18 (-1.65, 1.29) | -0.08 (-0.76, 0.59) | tES | -0.12 (-0.48, 0.24) |
| -0.49 (-0.67,-0.30) | -0.30 (-1.72, 1.12) | -0.20 (-0.78, 0.37) | -0.12 (-0.48, 0.24) | SHM |

## **11.10 Studies excluding imputed values**

Quantifying heterogeneity / inconsistency:

tau^2 = 0.2100; tau = 0.4582; I^2 = 67.7% [55.4%; 76.7%]

| TMS | . | . | . | -0.47 (-0.67,-0.27) |
| --- | --- | --- | --- | --- |
| -0.17 (-1.64, 1.30) | MST | -0.10 (-1.43, 1.23) | . | . |
| -0.26 (-0.89, 0.36) | -0.10 (-1.43, 1.23) | ECT | . | -0.21 (-0.79, 0.38) |
| -0.27 (-0.71, 0.16) | -0.11 (-1.61, 1.40) | -0.01 (-0.71, 0.69) | tES | -0.19 (-0.58, 0.19) |
| -0.47 (-0.67,-0.27) | -0.30 (-1.76, 1.15) | -0.21 (-0.79, 0.38) | -0.19 (-0.58, 0.19) | SHM |

## **11.11 Excluding studies from mainland China**

Quantifying heterogeneity / inconsistency:

tau^2 = 0.1303; tau = 0.3610; I^2 = 51.2% [30.8%; 65.7%]

| ECT | . | . | -0.20 (-0.72, 0.32) | -1.33 (-2.32,-0.34) |
| --- | --- | --- | --- | --- |
| -0.01 (-0.57, 0.54) | TMS | . | -0.19 (-0.38, 0.01) | . |
| -0.08 (-0.67, 0.51) | -0.06 (-0.41, 0.28) | tES | -0.12 (-0.40, 0.16) | . |
| -0.20 (-0.72, 0.32) | -0.19 (-0.38, 0.01) | -0.12 (-0.40, 0.16) | SHM | . |
| -1.33 (-2.32,-0.34) | -1.32 (-2.46,-0.18) | -1.26 (-2.41,-0.10) | -1.13 (-2.25,-0.01) | TAU |

## **11.12 Excluding studies in treatment-resistant positive symptom domains**

Quantifying heterogeneity / inconsistency:

tau^2 = 0.2766; tau = 0.5259; I^2 = 77.1% [66.4%; 84.5%]

| TMS | . | . | . | -0.58 (-0.83,-0.33) | . |
| --- | --- | --- | --- | --- | --- |
| -0.21 (-1.85, 1.43) | tES | . | . | -0.37 (-2.00, 1.25) | . |
| -0.32 (-2.03, 1.38) | -0.12 (-2.46, 2.22) | MST | -0.10 (-1.52, 1.33) | . | . |
| -0.42 (-1.37, 0.52) | -0.21 (-2.07, 1.65) | -0.10 (-1.52, 1.33) | ECT | -0.16 (-1.07, 0.75) | -1.33 (-2.58,-0.09) |
| -0.58 (-0.83,-0.33) | -0.37 (-2.00, 1.25) | -0.26 (-1.95, 1.43) | -0.16 (-1.07, 0.75) | SHM | . |
| -1.75 (-3.32,-0.19) | -1.55 (-3.78, 0.69) | -1.43 (-3.32, 0.46) | -1.33 (-2.58,-0.09) | -1.17 (-2.71, 0.37) | TAU |

# **12. Investigation of small study effect and publication bias**

Due to the small sample sizes, limited head-to-head comparisons, and the star-shaped network of NIBS studies, we focused on the small-study effects and publication bias for the primary outcome (i.e., overall symptoms) rather than on dropouts.

Contour-enhanced funnel plots did not indicate small-study effects for either comparison (Egger’s test: SHM vs TMS p=0.71, SHM vs tES p=0.94; Begg’s test p=0.50 and 0.78, respectively).

# **13. Evaluating the Confidence in NMA: CINeMA**

We used the official webtool at https://cinema.ispm.unibe.ch/ and followed the CINeMA-guidance-document .

The CINeMA-tool provides a framework for evaluating the confidence in the estimates of a network-meta-analysis in six different domains. It uses the original data to run a network-meta-analysis in order to evaluate the results for each comparison and to calculate a contribution matrix. Therefore, some settings and judgements need to be given, which we report in the following.

Due to the small sample sizes, limited head-to-head comparisons, and the star-shaped network of NIBS studies, we focused on the confidence in the evidence for the primary outcome (i.e., overall symptoms) rather than on dropouts.

**Domain 1: WITHIN-STUDY BIAS**

We used the overall risk of bias rating from the Cochrane risk of bias tool 2 (eAppendix 5).

For studies with multiple comparisons, we used the worst overall risk of bias rating of all comparisons as the overall risk of bias rating of the study (i.e. when one comparison was judged at high risk, then the whole study was considered as at high risk).

**Domain 2: REPORTING BIAS**

We accessed reporting bias using ROB-MEN (Risk of Bias due to Missing Evidence in Network meta-analysis). ROB-MEN, as part of the CINeMA framework, is a web application (https://cinema.ispm.unibe.ch/rob-men/) that guides the evaluation of risk of bias due to missing evidence in the estimates from network meta-analysis.

**Domain 3: INDIRECTNESS**

Regarding population, we only included trials involving individuals with treatment-resistant schizophrenia or related disorders. We excluded trials in specific populations, such as those with comorbidities, cognitive impairment, or predominantly negative symptoms. Therefore, we consider the study populations as directly relevant for the research question.

Regarding interventions, all investigated interventions were NIBS treatments (or sham stimulation) and thus directly relevant for the research question.

Regarding outcomes, no indirect measurements of the outcome were used. Therefore, we consider the study outcomes as directly relevant for the research question.

Regarding settings, we included studies conducted with out-patients, in-patients or both. All these settings can be relevant to the research question.

**Domain 4/5/6: IMPRECISION/** **HETEROGENEITY/ INCOHERENCE**

These three domains require to set thresholds for clinically important differences between interventions. We considered SMDs outside the interval of -0.1 to 0.1 as potentially clinically important. Comparisons could be classified according to heterogeneity evaluation as ‘no concerns’, ‘some concerns’, ‘major concerns’.

**Summarizing judgements across the six domains: OVERALL CINeMA LEVEL**

In the CINeMA framework, the overall levels of confidence in the estimates are:

1. high

2. moderate

3. low

4. very low

The CINeMA-guidance-document suggests for each comparison to start at the first level (i.e. high) and to downgrade for one level for a rating of “some concerns”, and by two levels for a rating of “major concerns”. In case, several domains are rated at some concerns or major concerns, it is recommended to consider judgements on different domains jointly rather than in isolation. The reason is that domains are interconnected and downgrading more than once for related concerns should be avoided. (The following examples are given in the guidance document: Heterogeneity will increase imprecision in treatment effects and may be related to variability in within- study bias or the presence of reporting bias. Indirectness includes considerations on intransitivity, which manifests itself in the data as statistical incoherence. In the worked example there is ‘some concerns’ for imprecision and heterogeneity and ‘major concerns’ for incoherence. Downgrading by two levels is considered to be sufficient in this situation, because imprecision, heterogeneity, and incoherence are interconnected.)

Based on these recommendations, we used the following approach to reach an overall level of confidence for each comparison and outcome:

1 judgement of “some concerns” leads to downgrading by 1 level.

1 judgement of “major concerns” leads to downgrading by 2 levels.

2 judgements of “some concerns” could be interconnected and do not justify downgrading more than by 1 level.

1 judgement of “major concerns” and up to 2 judgements of “some concerns” or 1 additional judgement of “major concerns” could be interconnected and do not justify downgrading by more than 2 levels.

2 judgements of “major concerns” and any additional judgements of “some concerns” or “major concerns” (or more than 4 judgements of some concerns) lead to downgrading by three levels (the maximum).

| Comparison | Number of studies | Within-study bias | Reporting bias | Indirectness | Imprecision | Heterogeneity | Incoherence |  | Confidence rating |
| --- | --- | --- | --- | --- | --- | --- | --- | --- | --- |
| ECT:MST | 1 | Some concerns | Some concerns | No concerns | Major concerns | No concerns | Major concerns |  | Very low |
| ECT:SHM | 4 | Major concerns | Some concerns | No concerns | Major concerns | No concerns | Major concerns |  | Very low |
| ECT:TAU | 1 | Some concerns | Some concerns | No concerns | No concerns | Some concerns | Major concerns |  | Very low |
| SHM:TMS | 50 | Some concerns | Some concerns | No concerns | No concerns | Major concerns | Major concerns |  | Very low |
| SHM:tES | 13 | Some concerns | Some concerns | No concerns | Some concerns | Some concerns | Major concerns |  | Very low |
| ECT:TMS | 0 | Major concerns | Low risk | No concerns | Major concerns | No concerns | Major concerns |  | Very low |
| ECT:tES | 0 | Major concerns | Low risk | No concerns | Major concerns | No concerns | Major concerns |  | Very low |
| MST:SHM | 0 | Some concerns | High risk | No concerns | Major concerns | No concerns | Major concerns |  | Very low |
| MST:TAU | 0 | Some concerns | High risk | No concerns | Major concerns | No concerns | Major concerns |  | Very low |
| MST:TMS | 0 | Some concerns | Low risk | No concerns | Major concerns | No concerns | Major concerns |  | Very low |
| MST:tES | 0 | Some concerns | Low risk | No concerns | Major concerns | No concerns | Major concerns |  | Very low |
| SHM:TAU | 0 | Some concerns | Low risk | No concerns | Major concerns | No concerns | Major concerns |  | Very low |
| TAU:TMS | 0 | Some concerns | High risk | No concerns | No concerns | No concerns | Major concerns |  | Very low |
| TAU:tES | 0 | Some concerns | High risk | No concerns | No concerns | Major concerns | Major concerns |  | Very low |
| tES:TMS | 0 | Some concerns | Low risk | No concerns | Major concerns | No concerns | Major concerns |  | Very low |

# **14. Description of NIBS treatment**

## **14.1 Convulsive NIBS treatment**

**Electroconvulsive therapy**

Electroconvulsive therapy (ECT) involves delivering an electrical stimulus through electrodes to induce a generalized cerebral seizure, with the electrodes often placed on the bitemporal areas.

**Magnetic seizure therapy**

Magnetic seizure therapy (MST) induces a seizure using magnetic stimulation that is more focal and less intense compared to the electrical stimulation used in ECT.

## **14.2 Non-convulsive NIBS treatment**

**Transcranial magnetic stimulation**

- Repetitive transcranial magnetic stimulation (rTMS) is the intervention form of transcranial magnetic stimulation (TMS) where a rapidly changing magnetic field induces a changing electrical field, e.g., in cortex regions and can depolarize cortical neurons.

In rTMS protocols, single TMS pulses are applied in a repetitive sequence at constant rates, e.g., high frequency (>5 Hz, HF rTMS) or low frequency (≤1 Hz, LF rTMS) or as patterned stimulation, e.g., as theta burst stimulation (TBS), where just the intervals between trains of bursts define intermittent TBS (iTBS) or continuous TBS (cTBS) protocols. TBS protocols mimic burst patterns from basic neurophysiology, which are applied for inducing long-term potentiation or long-term depression.

A less common rTMS protocol is the alpha rTMS (αTMS), an HF rTMS approach, where the rTMS frequency is adjusted to the individual alpha EEG frequency.

As rTMS is a focal NIBS method, the targets are crucial for its action. Most rTMS studies in TRS target the tempoparietal region (rTMS-T), especially for persistent auditory hallucinations, whereas other target regions include the prefrontal cortex (rTMS-F), commonly in dorsolateral prefrontal cortex sites. fMRI-guided rTMS (rTMS-fMRI) is developed to deliver the rTMS treatment precisely to the identified brain network.

**Transcranial electrical stimulation**

Transcranial electrical stimulation (tES) involves a bipolar electrode in the scalp, allowing the electrical current to flow between the electrodes to alter brain function. Anode was mostly placed over the prefrontal cortex, and the cathode over the temporoparietal cortex. Current treatment protocols include:

- Transcranial direct current stimulation (tDCS) is the most studied tES which uses constant, low direct current to modulate cortical excitability.

- Transcranial alternating current stimulation (tACS) applies a low-intensity sinusoidal and biphasic electrical current to stimulate cortical neurons.

- Transcranial random noise stimulation (tRNS) is a variant form of tACS where alternating current is applied while both intensity and frequency of the current randomly change.
